# Ontogeny of the elemental composition and the biomechanics of radular teeth in the chiton *Lepidochitona cinerea*

**DOI:** 10.1186/s12983-022-00465-w

**Published:** 2022-06-11

**Authors:** Wencke Krings, Jan-Ole Brütt, Stanislav N. Gorb

**Affiliations:** 1 Department of Behavioral Biology, Institute of Cell and Systems Biology of Animals, Universität Hamburg, Martin-Luther-King-Platz 3, 20146 Hamburg, Germany; 2 Department of Mammalogy and Palaeoanthropology, Leibniz Institute for the Analysis of Biodiversity Change, Martin-Luther-King-Platz 3, 20146 Hamburg, Germany; 3 Department of Functional Morphology and Biomechanics, Zoological Institute, Christian-Albrechts-Universität zu Kiel, Am Botanischen Garten 9, 24118 Kiel, Germany

**Keywords:** Elemental composition, Nanoindentation, Functional gradients, Material properties, Mollusca, Biomechanics, Functional morphology, Feeding

## Abstract

**Background:**

The radula, a chitinous membrane with embedded teeth, is one important molluscan autapomorphy. In some taxa (Polyplacophora and Patellogastropoda) one tooth type (the dominant lateral tooth) was studied intensively in the last decades with regard to its mechanical properties, chemical and structural composition, and the relationship between these parameters. As the dominant lateral tooth is probably one of the best studied biological materials, it is surprising, that data on elements and mechanical properties of the other tooth types, present on a chiton radula, is lacking.

**Results:**

We provide data on the elemental distribution and mechanical properties (hardness and elasticity, i.e. Young’s modulus) of all teeth from the Polyplacophora *Lepidochitona cinerea* (Linnaeus, 1767) [Chitonidae: Ischnochitonidae]. The ontogeny of elements, studied by energy-dispersive X-ray spectroscopy, and of the mechanical properties, determined by nanoindentation, was analysed in every individual tooth type. Additionally, we performed breaking stress experiments with teeth under dry and wet condition, highlighting the high influence of the water content on the mechanical behaviour of the radula. We thereby could determine the forces and stresses, teeth can resist, which were previously not studied in representatives of Polyplacophora. Overall, we were able to relate the mineral (iron, calcium) content with the mechanical parameters (hardness and Young’s modulus) and the breaking force and stress in every tooth type. This led to a better understanding of the relationship between structure, material, and function in radular teeth. Further, we aimed at determining the role of calcium for the mechanical behaviour of the teeth: we decalcified radulae by ethylene diamine tetra acetic acid and performed afterwards elemental analyses, breaking stress experiments, and nanoindentation. Among other things, we detected that wet and decalcified radular teeth could resist highest forces, since teeth have a higher range of bending motion leading to a higher capability of teeth to gain mechanical support from the adjacent tooth row. This indicates, that the tooth material is the result of a compromise between failure reduction and the ability to transfer forces onto the ingesta.

**Conclusion:**

We present novel data on the elemental composition, mechanical properties, and the mechanical behaviour of chiton teeth, which allows conclusions about tooth function. We could also relate the parameters mentioned, which contributes to our understanding on the origins of mechanical property gradients and the processes reducing structural failure in radular teeth. Additionally, we add more evidence, that the elemental composition of radular is probably species-specific and could be used as taxonomic character.

**Supplementary Information:**

The online version contains supplementary material available at 10.1186/s12983-022-00465-w.

## Background

### The molluscan autapomorphy for feeding

The food processing and gathering structure, the radula, is an important autapomorphy of the molluscan phylum. Its diversity in morphology and structural organization has probably enabled the establishment of distinct ecological niches, rendering the Mollusca the second largest taxa next to Hexapoda.

The radula together with (1) the underlain odontophoral cartilages, (2) the surrounding radular muscles, (3) the alary processus, and (4), in some taxa, the jaw forms the feeding apparatus. The radula itself is a fibrous and chitinous membrane with small, embedded teeth (alpha chitin matrix with associated proteins) arranged serially in transversal and longitudinal rows [1, 2]. It is continuously renewed by the secretion of new teeth and membrane in the posterior ‘radular sac’ or 'building zone' and becomes maturated in the ‘mineralization zone’ by overlain epithelia [3–12]. Only the anterior tooth rows (‘working zone’) are actually used for feeding and come into contact with the ingesta (food, minerals, feeding substrate, etc.).

### The diversity of organism-ingesta interfaces

As the molluscan species are highly distinct, radular morphologies, including tooth shape, arrangement, quantity, mechanical properties, and chemical composition, are diverse as well. The morphological diversity in tooth arrangement and quantity led to the categorization of radulae into five to seven basic radular types (e.g. docogloss, toxogloss, taeniogloss, isodont, rhipidogloss). The precise shape of teeth is often used as taxonomic character, but as an interface to the ingesta, it reflects trophic preferences as well [e.g. 13, 14].

To gain deep insight into trophic adaptations, mechanical properties (e.g. Young’s modulus, hardness, and breaking stress), which have a high influence on the tooth’s biomechanics, and the tooth’s chemical composition were previously investigated in addition to morphology [15–29]. However, shape together with mechanical properties can also reflect functional specializations of the distinct tooth types, i.e. some teeth are rather used for scratching, and others for food gathering, together forming a multifunctional radula [14, 29–34]. Besides, in some taxa, some teeth do not necessarily interact with the ingesta; here their involvement either depends on the feeding substrate roughness or the teeth contribute to foraging by e.g. reinforcing the membrane [35–37]. Thus, in general, functional loads and thus forces on teeth can vary, even within one single radula.

Many of the past studies relating chemical composition, mechanical properties, morphology, functional loads, and ecology focus on Polyplacophora and patelliform Gastropoda with docoglossan and rhipidoglossan radulae [e.g. 19, 38–40]. The dominant lateral teeth of these taxa, used for loosening algae from rocky feeding substrates, were very well studied in the last decades due to their immense hardness and stiffness [for throughout reviews see 41–43]. These mechanical properties are based on substantial proportions of iron-based incorporations, which increase the cusps’ wear-resistance [17, 20, 44–49], and at least one non-iron element incorporated in teeth (e.g. silicon, calcium), probably serving as mechanical reinforcement [42].

However, many more species await deep investigations with regard to tooth mineralization and mechanical properties, as e.g. in Polyplacophora ~ 20 of ~ 940 species were examined so far. In addition, even in the well investigated taxa, usually the heavy mineralized dominant lateral tooth (termed here lateral tooth II) was studied, whereas for all other tooth types (centrals, lateral teeth I, and marginals) analyses on the chemical composition and mechanical properties are lacking.

### Aim of the study

Here we provide data on the elemental proportions, analysed by semi-quantitative energy-dispersive X-ray spectroscopy (EDX, EDS), and the mechanical properties hardness and elasticity (Young’s modulus), determined by nanoindentation, of teeth from *Lepidochitona cinerea* (Linnaeus, 1767) [Polyplacophora: Chitonida: Ischnochitonidae]. We studied the radular ontogeny with regard to elemental composition and mechanical parameters, not only in for the dominant lateral teeth, but for every tooth type, which was not done before. Additionally, we focused on the mechanical behaviour of teeth when hitting against an obstacle and their ability to resist structural failure. This was done by measuring the force needed to break teeth and calculating the breaking stress (breaking force divided by cross-sectional area of the tooth), which is also new set of data for Polyplacophora. We were able to relate such parameters as (1) mineral (iron, calcium) content, (2) hardness, (3) Young’s modulus, (4) breaking force, and (5) breaking stress for every tooth type, leading to hypotheses on tooth function. Further, we aimed at determining the role of calcium for the mechanical behaviour of the teeth: for this purpose, we decalcified radulae by ethylene diamine tetra acetic acid (EDTA) and performed afterwards semi-quantitative EDX (EDS) analyses, nanoindentation, and breaking stress experiments. These results were compared to those obtained on the untreated (native) radulae.

## Results

### Radular morphology

**Fig. 1:**
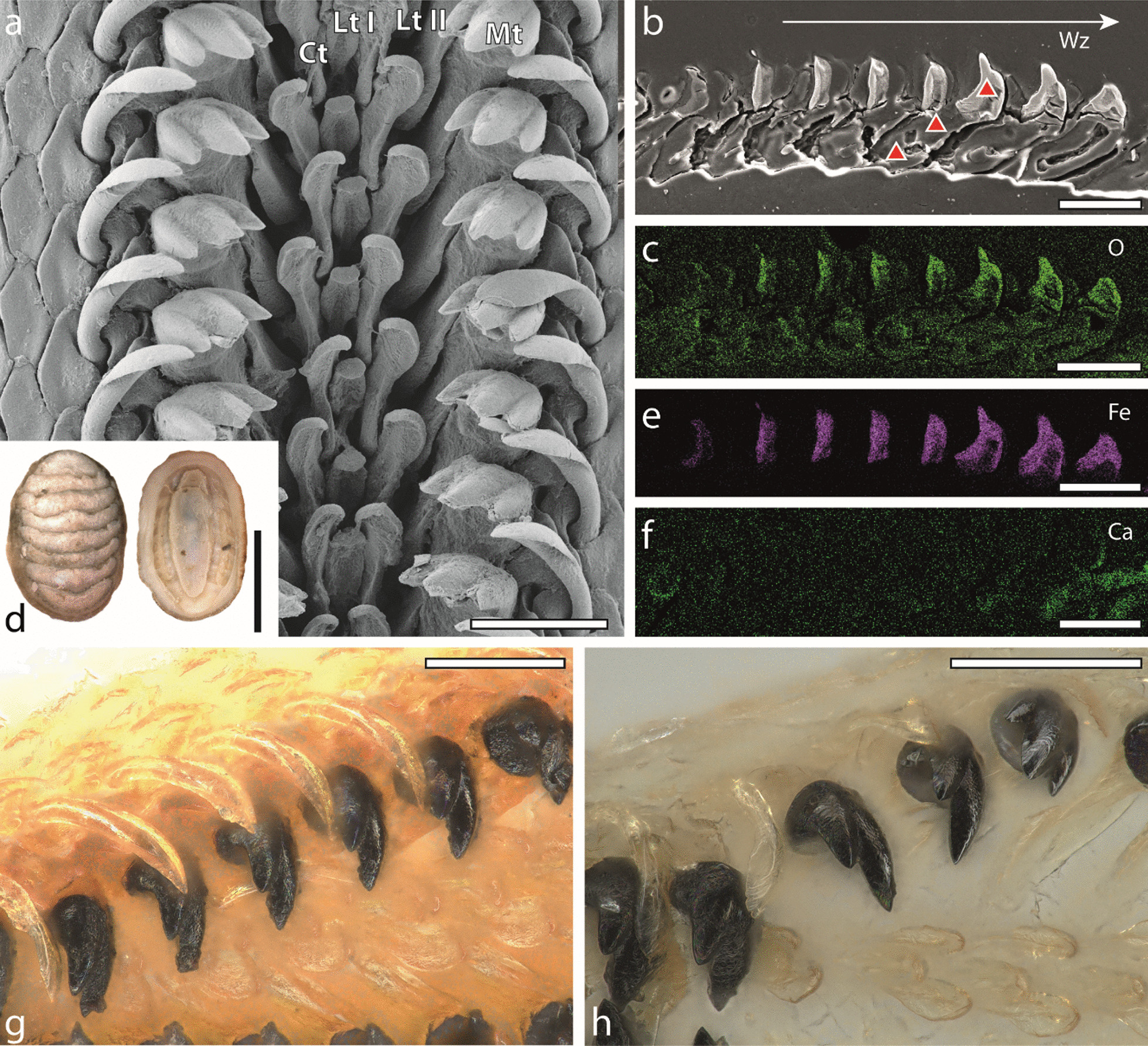
**a** SEM image of one representative radula of *Lepidochitona cinerea*, working zone (Wz), taken from [109]. **b**, **c**, **e**, **f** SEM image and EDX mapping of the embedded, native radula (working zone) from specimen number 1. **c** Oxygen (O) distribution. **e** Iron (Fe) distribution. **f** Calcium (Ca) distribution. **b** Red triangles represent the areas of nanoindentation for lateral tooth II (basis, stylus, cusp). **d** Habitus from one representative specimen, taken from [109]. **g** Light microscopy image of one native radula, working zone. Central teeth, lateral teeth I, marginal teeth are almost translucent, but slightly yellowish. The cusps of the lateral teeth II are clearly visible due to the high proportions of Fe. **h** Light microscopy image of one treated radula, working zone. Central teeth, lateral teeth I, and marginal teeth are more translucent. Scale bars: **a**–**c**, **e**–**h** = 100 μm, **d** = 5 mm. Ct, central tooth; Lt I, lateral tooth I; Lt II, lateral tooth II; Mt, marginal tooth

The radula of *Lepidochitona cinerea* is docogloss with seven teeth per row: the central tooth (Ct) is median, flanked by one small lateral tooth (Lt I), one dominant lateral tooth (Lt II), and one marginal tooth (Mt) on every side (Fig. 1a). The central tooth is small and slender with a small spoon-like cusp. The lateral tooth I is of rather similar shape as the central tooth but exceed it in size. The lateral tooth II is the largest and most prominent tooth on the radula, its cusps is of paw-like shape possessing three denticles. The marginal teeth are slender, their styli are of a roundish shape and their cusps have a spoon-like morphology.

### Radular colour

**Fig. 2:**
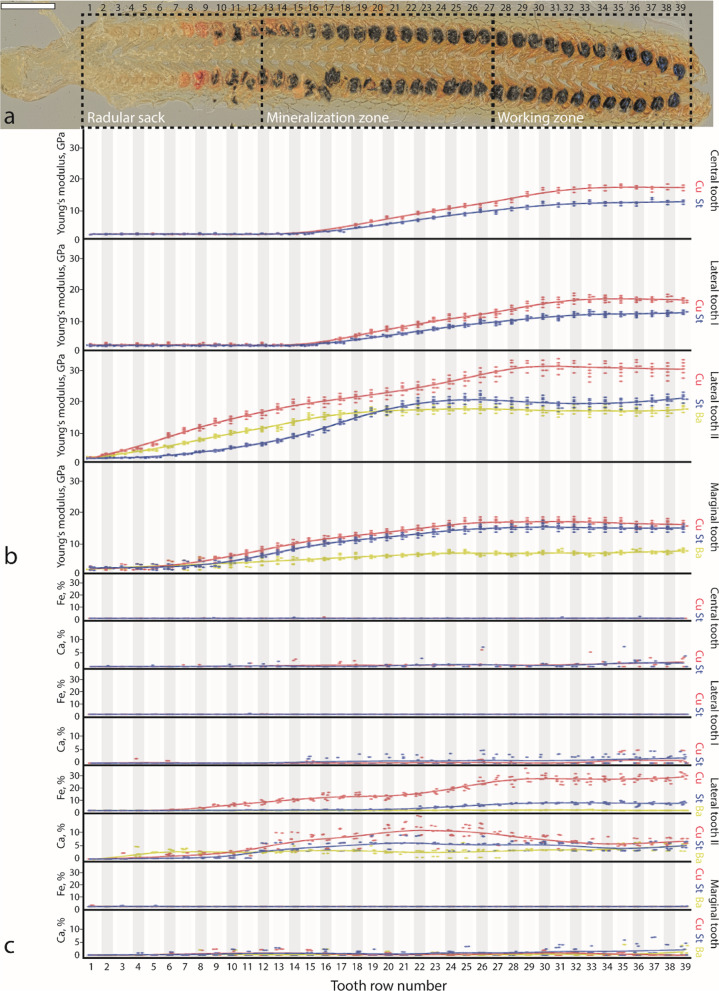
EDX and nanoindentation data obtained from native radulae (*n* = 3). **a** Light microscopy image of one radula, tooth rows are numbered, and the zones (radular sac, mineralization zone, working zone) are defined. **b** Changes in Young’s modulus, GPa, during ontogeny for each tooth type and tooth part (Ba, basis; Cu, cusp; St, stylus). **c** Changes in Fe and Ca proportions (atomic %) during ontogeny for each tooth type and tooth part. Dots represent values measured and solid lines trend lines based on the individual measurements. Scale bar: **a** = 250 μm

Overall, the radula has a yellowish colour gradually shifting into orange from the radular sac to the working zone, while remaining somewhat transparent (Fig. 2a). The most prominent colour change during maturation can be observed in the lateral teeth II. In the radular sac they are of a rather uniformly distributed white-yellow colour. Within three rows, starting in tooth row 8, their cusps change colour from pale yellow to a deeper yellow. From row 10 on, cusps become red and turn black and no transparency is left after row 17. The shift in colour from the black cusps to the yellow colour of the stylus in abrupt and not gradual (Figs. 1g and 2a, Additional file 1: Fig. 7a). This area was termed previously as junction zone [see e.g. 50, 51].

The iron dominated lateral II tooth cusps of the treated specimens are similar in colour to the native ones; however, the styli and bases of the lateral teeth II as well as the centrals, lateral teeth I, marginals are rather whitish (Fig. 1h, Additional file 1: Fig. 7b, Additional file 1: Fig. 5a).

### Tooth failure during breaking stress experiments

Certain mechanical behaviours are observed during breaking stress experiments (see Additional file 1: Fig. 3 and Additional file 1: Table 9): some teeth break close to the membrane at their basis or their styli, some are completely crushed, break underneath their junction zone or at their junction zone. In some cases, it is impossible to break teeth due to bending or twisting.

Native and treated radulae exhibit similar mechanical behaviours: wet marginal teeth, lateral teeth I, and central teeth bent in most experiments and thus do not fail; especially the younger teeth of the radular sac and mineralization zone. If breaking occurs, teeth either break at their basis or stylus (Additional file 1: Fig. 2b and c). Wet lateral teeth II of the working and mineralization zone fail underneath their junction zone. In the radular sac, these teeth are, however, capable of bending (in 36% of cases). Dry marginal teeth fail in all cases at their basis, dry lateral teeth I, and central teeth are always completely crushed. Dry lateral teeth II of the working and mineralization zones always fail at their junction zone (Additional file 1: Fig. 2b and c). In the radular sac, these teeth fail in 24 cases at their junction zone and are crushed in 23 cases.

### Variability between the radulae, sorted to each treatment group (native or treated)

#### Elements

In all radulae of *Lepidochitona cinerea* (native and treated) we detect Na, K, Si, Mg, P, S, Cl, Ca, and Fe. These elements are found in all three zones of every individual. Proportions of every element in every radula are non-normally distributed (when the data from all teeth is pooled together). Kruskal–Wallis test detects no significant difference between the radulae of either the native or the treated group in the proportions of the individual element; only for Si we detect significant differences between radulae from the native group (see Additional file 1: Table 1).

#### Young’s modulus and hardness

In all individual radulae of *Lepidochitona cinerea* (native and treated), the data on E and H are normally distributed (when the data of all teeth is pooled together). ANOVA detects significant differences between the individuals of the native group for E and H and between the individuals of the treated group for H (see Additional file 1: Table 1).

#### Breaking force and stress

Breaking force and stress of every individual radula are normally distributed (when the data from all teeth is pooled together). T test detects no significant differences for both parameters between the individuals of the following groups: native dry, native wet, treated dry, treated wet (see Additional file 1: Table 1).

### Parameters of the whole radulae (data from all zones is pooled together)

#### Elements

Proportions of all elements (Ae) are non-normal distributed in native and treated group. The native radulae possess more elements and Wilcoxon test detects significant difference between native and treated group for proportions of Ae (see Additional file 1: Table 2).

For the native radulae, we determine that Fe occurs with the highest proportion, followed by Ca, P, Na, Mg, S, Cl, K, and finally Si (see Additional file 1: Table 2). In the treated radulae, Fe is also present with the highest proportion, followed by Ca, Cl, P, K, S, Mg, Na, and finally Si.

Fe, Ca, Na, K, Si, Mg, P, S, and Cl proportions of native and treated radulae are non-normal distributed. Between treated and native radulae, Wilcoxon test detects no significant difference in Fe and K, but significant difference in Na, Mg, Si, P, Cl, and Ca (see Additional file 1: Table 2).

#### Young’s modulus and hardness

Both E and H values of the treated and the native group are non-normally distributed. The native radulae have higher E and H values than the treated radulae. Wilcoxon test detects significant difference for E and H between treated and native radulae (see Additional file 1: Table 2).

#### Breaking force and stress

Breaking force and breaking stress values at different testing condition (dry or wet) are normally distributed. Between dry and wet condition, t test detects significant differences for force and stress. Breaking force and stress are higher for wet teeth than for dry ones (see Additional file 1: Table 2).

Breaking force and stress sorted to the treated dry, treated wet, native dry, or the native wet group are normally distributed. Highest force must be exerted to break teeth of the treated wet radulae, followed by native wet, native dry, and finally treated dry ones. ANOVA detects significant differences between these groups (see Additional file 1: Table 2).

Highest breaking stress is calculated for the treated wet radulae, followed by native wet, treated dry, and finally native dry ones. ANOVA detects significant differences between these groups (see Additional file 1: Table 2).

### Parameters of each ontogenetic zone (data from tooth types is pooled together)

#### Elements

*Native*. For native radulae, the highest proportion of Ae is found in the working zone, followed by the mineralization zone, and finally radular sac (see Additional file 1: Table 3). The same pattern is detected for Fe, P, Si, and Mg proportions. For Na, K, and Ca, the highest proportions are determined for the working zone, followed by the radular sac, and finally mineralization zone. For S and Cl, the highest proportions are found in the mineralization zone, followed by the radular sac, and finally working zone. Proportions of each element, sorted to each zone (radular sac, mineralization zone, working zone), are non-normally distributed and Kruskal–Wallis test detects significant differences between radular sac, mineralization zone, and working zone (see Additional file 1: Table 3).

*Treated*. For treated radulae, the highest proportion of Ae is determined for the working zone, followed by the mineralization zone, and finally radular sac (see Additional file 1: Table 3). The same pattern is found for Fe and Ca. For K, the highest proportion is detected in the working zone, followed by the radular sac, and finally mineralization zone. For Cl and S, the highest proportion is found in the mineralization zone, followed by the radular sac, and finally working zone. For Mg, the highest proportion is detected in the working zone, followed by the radular sac, and finally mineralization zone. Na, Si, and P are only found in one zone. Proportions of each element, sorted to each zone (radular sac, mineralization zone, working zone), are non-normally distributed. Kruskal–Wallis test determines no significant difference between proportions of the radular sac, mineralization zone, and working zone for Na, Mg, Si, P, and S (see Additional file 1: Table 3). Significant differences are found for Cl, K, Ca, Fe, and Ae.

#### Young’s modulus and hardness

For native and treated radulae, the highest E and H are found in the working zone, followed by the mineralization zone, and finally the radular sac (see Additional file 1: Table 3). The E and H for every zone are normally distributed and ANOVA detects significant differences between radular sac, mineralization zone, and working zone of either treated or native radulae (see Additional file 1: Table 3).

#### Breaking force and stress

Breaking force and stress, sorted to zone (radular sac, mineralization zone, working zone) and condition (treated dry, treated wet, native dry, or the native wet), are non-normally distributed. For every parameter, Kruskal–Wallis test detects significant differences (see Additional file 1: Table 3). The highest forces and the highest stress are calculated for breaking teeth of the working zone, followed by the mineralization zone, and finally the radular sac for every condition (see Additional file 1: Table 3).

### Parameters of each tooth type (data from all zones is pooled together)

#### Elements

*Native*. The highest proportions of Ae, Ca, and Si are detected in the lateral teeth II, followed by the marginal teeth, lateral teeth I, and finally central teeth (see Additional file 1: Table 4). For Fe, the highest proportions are found in the lateral teeth II, followed by marginal teeth, central teeth, and finally lateral teeth I. For K, lateral teeth II have the highest proportions, followed by central teeth, marginal teeth, and finally lateral teeth I. Cl is mostly present in lateral teeth II, followed by lateral teeth I, marginal teeth, and finally central teeth. The highest proportions of S are measured in the marginal teeth, followed by lateral teeth II, lateral teeth I, and finally central teeth. Na and P are only detected in the lateral teeth II, here with the highest proportions, and in the marginal teeth. Mg is found only in the lateral teeth II.

Proportions of each element, sorted to each tooth type (central tooth, lateral tooth I, lateral tooth II, marginal tooth), are non-normal distributed and Kruskal–Wallis test detects in most cases significant differences (except for Si, S, and Cl) (see Additional file 1: Table 4).

*Treated*. For the treated radulae, the highest proportions of Ae, Fe, and Ca are detected in the lateral teeth II, followed by the marginal teeth, central teeth, and finally lateral teeth I (see Additional file 1: Table 4). K is determined in the lateral teeth II with the highest proportion, followed by marginal teeth, and central teeth, but not in the lateral teeth I. S is not detected in the marginal teeth; its highest proportion is found in the lateral teeth I, followed by lateral teeth II, and central teeth. P is not detected in marginals and centrals; its highest proportion is found in lateral teeth I followed by lateral teeth II. Si is only determined in marginal teeth, Mg in central teeth, and Na in lateral teeth II.

Proportions of each element, sorted to each tooth type (central tooth, lateral tooth I, lateral tooth II, marginal tooth), are non-normally distributed and Kruskal–Wallis test detects in most cases no significant differences (except for Mg, Ca, Fe, and Ae) (see Additional file 1: Table 4). Wilcoxon test (see Additional file 1: Table 5) detects significant differences between proportions of Ae, Ca, and S in treated and native radulae, sorted to each tooth type, and no significant differences between Fe and K proportions in treated and native radulae, sorted to each tooth type. For all other elements, the picture is rather puzzling.

#### Young’s modulus and hardness

For treated and native radulae, E and H values, sorted to each tooth type, are normally distributed. The hardest and stiffest teeth are always the lateral teeth II, followed by marginal teeth, lateral teeth I, and finally central teeth (see Additional file 1: Table 4). ANOVA detects significant differences for E and H between the tooth types within the either treated or native group (see Additional file 1: Table 4). Tukey–Kramer test reveals significant differences between E and H of treated and native radulae, sorted to each tooth type (see Additional file 1: Table 5).

#### Breaking force and stress

Breaking force and breaking stress, sorted to tooth type and condition (treated dry, treated wet, native dry, or the native wet), are normally distributed. For breaking force, ANOVA detects significant differences. For breaking stress, t test also detects significant differences (see Additional file 1: Table 4).

For each condition (treated dry, treated wet, native dry, or the native wet), the highest forces are needed for breaking lateral teeth II, followed by central teeth, lateral teeth I, and finally marginal teeth. The highest breaking stresses are calculated for lateral teeth II and the lowest one for marginal teeth (see Additional file 1: Table 4).

Tukey–Kramer test reveals significant differences in most cases for breaking force and breaking stress between dry or wet, treated or native condition (see Additional file 1: Table 5).

### Parameters of each tooth type (sorted to each ontogenetic zone)

#### Elements

Elements are non-normal distributed. Regarding Ae, the Wilcoxon method detects significant differences between treated and native marginal teeth, lateral teeth II, lateral teeth I, and central teeth of both working and mineralization zones, but only between treated and native lateral teeth II and marginal teeth of the radular sac (see Additional file 1: Table 8). For Fe and K, no significant differences between treated and native teeth are detected. For Ca, all teeth of both working and mineralization zones are significantly different. Only the central teeth or radular sac are not different. For Cl, significant differences are detected in the mineralization zone for the lateral tooth I, lateral tooth II, and marginal tooth. For S, significant differences are detected for each tooth type of the mineralization zone. For the radular sac, only the lateral tooth I and marginal teeth differ significantly. For the working zone, only the lateral teeth II differ significantly. With regard to P and Mg, lateral teeth II from the working zone and mineralization zone differ significantly. For Si, differences are detected for lateral teeth II of the working zone and for marginal teeth of the mineralization zone. For Na, treated and native lateral teeth II are significantly different in each zone. In the radular sac, marginal teeth differ significantly.

*Native*. Whole element content is always highest in lateral tooth II in every zone (see Additional file 1: Table 6). In the radular sac, it is followed by marginal tooth, central tooth, and finally lateral tooth I. In the mineralization and working zones, it is followed by the central tooth, lateral tooth I, and finally marginal tooth.

When analyzing the individual elements, Fe is present in the highest proportions, however, only in lateral tooth II (Additional file 1: Fig. 1). Here proportions increase during ontogeny. For all other teeth, Fe is detected in very small proportions and the distribution does not seem to follow a clear pattern. In the central teeth, the proportions increase during ontogeny. In the lateral tooth I, they decrease from the radular sac to the working zone. For the marginal teeth, Fe proportions first increase from the radular sac to the mineralization zone, but then decrease to the working zone.

The second most abundant element is Ca. In every zone, the lateral tooth II always contains the highest proportions of Ca (Additional file 1: Fig. 1). In the radular sac, it is followed by the marginal tooth, central tooth, and finally lateral tooth I. In the mineralization and working zones it is followed by the central tooth, lateral tooth I, and finally marginal tooth. For the central tooth, lateral tooth I, and marginal tooth, Ca proportions increased during ontogeny. For the lateral tooth II, Ca proportions first increase from the radular sac to the mineralization zone, but then decrease to the working zone.

However, for many elements, especially those that are rarely detected as trace elements, (Na, Si, K, Mg, P, S, Cl), the elemental distributions are rather puzzling and no clear gradients during ontogeny can be detected for the individual tooth types (Additional file 1: Fig. 1; here Na, Mg, Si, P, S, Cl, and K are summarized as Te ‘trace elements ‘). Mg is only detected in the lateral tooth II, its proportions slightly increases during ontogeny. P is detected in the lateral tooth II and marginal tooth, increasing during ontogeny. S and Cl proportions first increase from the radular sac to the mineralization zone, but then decrease in the working zone in each tooth type.

*Treated*. The highest proportions of Ae are found in the lateral tooth II, in every zone (see Additional file 1: Table 6). In every zone, the content decreases in the series central tooth—> marginal tooth—> lateral tooth I.

Fe is highly present only in the lateral tooth II (Additional file 1: Fig. 1). Highest Fe proportions are detected in the working zone, followed by the mineralization zone, and finally the radular sac. For all other teeth, Fe is detected in very small proportions and its distribution does not seem to follow a clear pattern. However, for central teeth, proportions increase during ontogeny. For the lateral tooth I, the amount of Fe decreases from the radular sac to the working zone. For the marginal teeth, Fe proportions first increase from the radular sac to the mineralization zone, but then decrease to the working zone.

Ca is less abundant than in native radulae (Additional file 1: Fig. 1). However, in each zone, the lateral tooth II contains the highest proportions of Ca. The distribution pattern differs however from the native radulae. In the radular sac and working zone, it is followed by the central tooth, marginal tooth, and finally lateral tooth I. In the mineralization zone, it is followed by the marginal tooth, central tooth, and finally lateral tooth I. For the central tooth, lateral tooth II, and marginal tooth, the highest Ca proportions are detected in the working zone, followed by mineralization zone, and finally radular sac. For the lateral tooth I, Ca is only detected in the mineralization zone.

Na, Mg, Si, P, S, Cl, K are only very occasionally detected in treated radulae (Additional file 1: Fig. 1, here Na, Mg, Si, P, S, Cl, and K are summarized as Te, ‘trace elements ‘).

#### Young’s modulus and hardness

Both E and H data are normally distributed. The Tukey–Kramer method detects significant differences between treated and native marginal teeth, lateral teeth II, lateral teeth I, and central teeth of the working zone (see Additional file 1: Table 8). For the mineralization zone, significant differences are detected between native and treated lateral teeth II, lateral teeth I, and marginal teeth. For the radular sac, significant differences are detected between native and treated lateral teeth II, and marginal teeth.

*Native*. Each tooth type becomes stiffer and harder during ontogeny (see Additional file 1: Fig. 1 and Additional file 1: Table 6). In each zone, the lateral tooth II is always the hardest and stiffest one. In the working zone, it is followed by the lateral tooth I, central tooth, and finally marginal tooth. In the mineralization zone and radular sac, it is followed by marginal tooth, lateral tooth I, and finally central tooth.

*Treated*. Each tooth type is the stiffest and hardest in the working zone, followed by the mineralization zone, and finally radular sac (see Additional file 1: Fig. 1 and Additional file 1: Table 6). In each zone, the lateral tooth II is always the hardest and stiffest one. It is followed by the central tooth, lateral tooth I, and marginal tooth in the working zone; by the marginal tooth, lateral tooth I, and central tooth in the mineralization zone and radular sac.

#### Breaking force and stress

Sorted to each condition (treated dry, treated wet, native dry, or the native wet), breaking force and breaking stress are non-normally distributed. In most cases, significant differences between the treated and native tooth types in every zone are detected (see Additional file 1: Table 8).

**Fig. 3:**
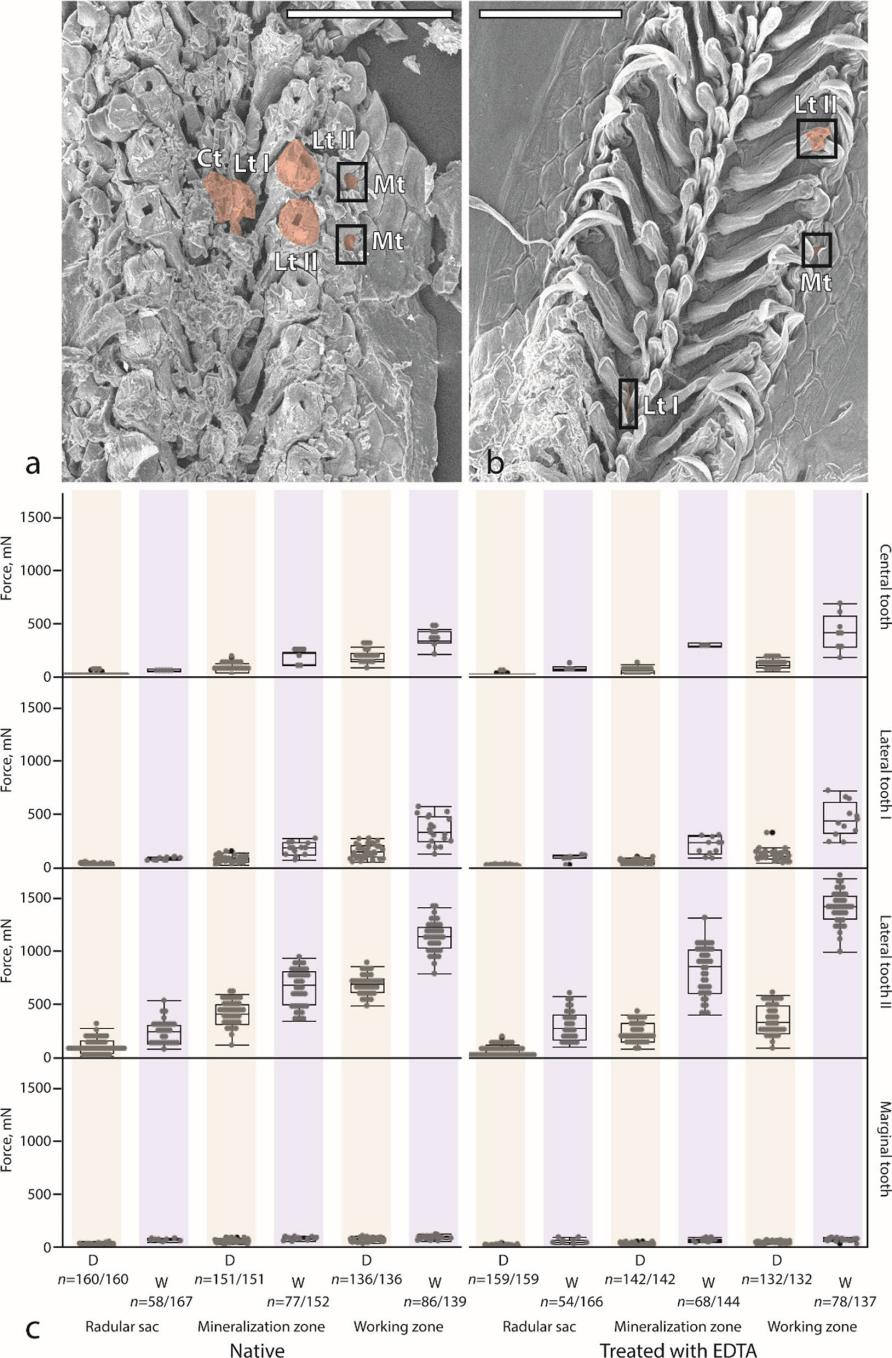
**a**, **b** Broken teeth (Ct, central tooth; Lt I, lateral tooth I; Lt II, lateral tooth II; Mt, marginal tooth) of **a** one dry, native radula and **b** one wet, treated radula. **c** Breaking force, mN, for each radular zone, tooth type, and condition (native dry, native wet, treated dry, treated wet) with quantity (*n*) of broken teeth. Brown colour highlight the results obtained by testing dry teeth and blue the results obtained from testing wet teeth. Scale bars: **a**–**b** =  200 μm. D, dry; W, wet

*Native*. Tested under dry condition, the highest force (see Fig. 3c and Additional file 1: Table 7) must be exerted to break the lateral teeth II, followed by central teeth, lateral teeth I, and finally marginal teeth in each zone. Tested under wet condition, the same pattern is found for teeth of the mineralization and working zones. For the wet radular sac, the lateral teeth II are, however, followed by the lateral teeth I, central teeth, and finally marginal teeth. The breaking stress does not fully relate to this pattern (see Additional file 1: Fig. 2a and Additional file 1: Table 7). For every dry radular zone, the highest breaking stress is calculated for the marginal teeth and the lowest one for the lateral teeth II. Tested under wet condition, the highest stress is found for the lateral teeth II and the lowest one for the marginal teeth for each zone.

*Treated*. Tested under dry condition, the highest force must be exerted to break lateral teeth II, followed again by central teeth, lateral teeth I, and finally marginal teeth of each zone (see Fig. 3c and Additional file 1: Table 7). Tested under wet condition, the same pattern is found for teeth of the radular sac and mineralization zone. For the wet working zone, the lateral teeth II are followed by lateral teeth I, central teeth, and finally marginal teeth. The breaking stress does not fully relate to this pattern (Additional file 1: Fig. 2a). For each zone, the highest breaking stress is calculated for the marginal teeth and the lowest one for the lateral teeth II. Tested under wet condition, the highest stress is found for lateral teeth II and the lowest one for the marginal teeth for each zone.

### Ontogenetic changes of each tooth type (focus on gradients within each tooth)

#### Elements

**Fig. 4:**
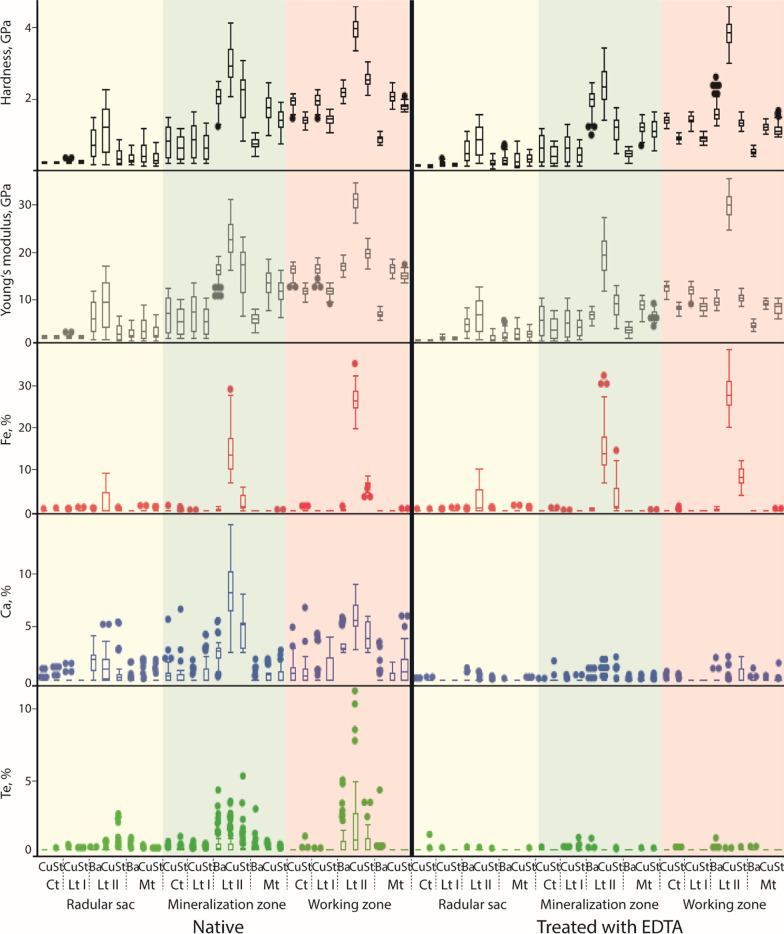
For each tooth type (Ct, central tooth; Lt I, lateral tooth I; Lt II, lateral tooth II; Mt, marginal tooth), part (Ba, basis; Cu, cusp; St, stylus), and each zone: Hardness, given in GPa, Young’s moduli, given in GPa; Fe, Ca, and Te (‘trace elements’) proportions, given in atomic %. Left side: native radulae, right side: treated radulae

Overall, no gradients within teeth are determined for Na, Mg, Si, P, S, Cl, and K, since these elements are only occasionally detected in native and treated radulae (see Fig. 4, here these elements are summarized as Te ‘trace elements ‘, and Additional file 1: Table 10). We therefore focus on Ca, Fe, and Ae. Sorted to the tooth type, tooth area (basis, stylus, cusp), and zone, these elements are normally distributed.

*Native*. For Ca, no clear gradients are detected within central teeth and lateral tooth I (see Fig. 4 and Additional file 1: Table 10). However, the lateral tooth II and marginal tooth exhibit gradients. For the lateral tooth II, gradients are already present in the radular sac (here the basis contains the highest proportion of Ca, followed by the cusp, and finally the stylus). In the mineralization and working zone, the cusps contain most Ca, followed by the stylus, and finally the basis. For the marginal teeth, gradients are detected in the mineralization and working zones. In both zones, the stylus contains the most Ca, followed by the cusp, and finally the basis. For Fe, a gradient is detected in only lateral teeth II (see Fig. 4); the cusps contain the highest Fe proportions, followed by the stylus and finally by the basis in each zone.

*Treated*. With the one exception (lateral tooth II of the working zone), all teeth are homogenous in their Ca distribution (see Fig. 4 and Additional file 1: Table 10). For Fe, the same pattern as in native radulae is detected (see Fig. 4).

#### Young’s modulus and hardness

Sorted to the tooth type, tooth area (basis, stylus, cusp), and zone, E and H are normally distributed. Within each tooth type, with some exceptions in the radular sac, t test (for the central and lateral tooth I) or ANOVA (for the lateral tooth II and marginal tooth) detects significant differences between basis, stylus, and cusps for treated and native radulae (see Additional file 1: Table 10).

*Native*. In the radular sac, no gradients within the central tooth are detected (see Fig. 4 and Additional file 1: Table 10). They are detectable for the lateral tooth I and marginal tooth and pronounced in the lateral tooth II. The cusp of the tooth are always the stiffest and hardest areas and the stylus was much softer. For the lateral tooth II and marginal tooth E and H of the tooth basis are also measured. In the marginal teeth, the basis is softer and more flexible than the stylus, but for the lateral teeth II, the stylus is softer than the basis. In the mineralization and working zones, all teeth possess pronounced gradients from the cusp, as the stiffest and hardest part, across the stylus to the basis, as the softest and most flexible part.

*Treated*. In the radular sac, no gradients within the tooth are detected for the lateral tooth I and the marginal tooth (see Fig. 4 and Additional file 1: Table 10). Gradients are present in the central tooth and are pronounced in the lateral tooth II. Here the cusp is again the stiffest and hardest part and the stylus is much softer. In the lateral teeth II, the stylus is again softer than the basis. In the mineralization and working zones, gradients are similar to those of the native group.

### Ontogenetic changes between the individual tooth rows

#### Elements

*Native and treated*. Proportions of Fe, Ae, and Te in each tooth part gradually increase (see Additional file 1: Fig. 4, Additional file 1: Fig. 5, Additional file 1: Fig. 6, and Additional file 1: Table 12). Ca proportions in the native lateral teeth II first increase during ontogeny and then decrease after row 25 (see Fig. 2 and Additional file 1: Table 12). The Ca content of the treated lateral teeth II fluctuates highly between tooth rows. Proportions of Ae and the Te are significantly lower in each treated tooth row (see Additional file 1: Figs. 4 and 6, and Additional file 1: Table 12).

#### Young’s modulus and hardness

*Native*. E and H values of all central tooth parts, all lateral tooth I parts, lateral tooth II cusp and basis, and all marginal tooth parts rather increase gradually during ontogeny (see Fig. 2, Additional file 1: Table 11, and Additional file 1: Table 12). E and H values of the lateral tooth II stylus increase strongly from row 17 to row 22.

*Treated*. In the treated radulae, the E and H values of all parts of the central and lateral teeth I, the stylus and bases of the lateral teeth II, and all parts of the marginals do not increase much (see Additional file 1: Fig. 5 and Additional file 1: Table 12). The E and H values of the lateral teeth II cusps seem to be, however, not affected by treatment.

### Relationship between elements, mechanical properties, and mechanical behaviour

#### Data from treated and native radulae pooled together

H and E are highly correlated (r = 0.99; see Additional file 1: Table 13 and Additional file 1: Figs. 8 and 9). H correlates with Fe proportion by a correlation coefficient of 0.76 and with Ca with the one of 0.68. For E, a row-wise correlation with Fe of 0.70 and with Ca of 0.70 is obtained. For Na, Mg, Si, P, S, Cl, and K, low correlation coefficients are determined. When relating the mean values of measured mechanical properties (H and E), element proportions (Ca and Fe) to the mean values of breaking force and breaking stress (see Additional file 1: Table 24 and Additional file 1: Figs. 8 and 9), we detect, that H, E, Fe, Ca, dry breaking force, wet breaking force, and wet breaking stress correlate with each other. Dry breaking stress correlates with wet breaking stress to 0.74 and to Fe with 0.34, but to low correlation coefficients with H, E, Ca, dry breaking stress, and wet breaking stress.

#### Sorted to treatment

For all native teeth together, H and E highly correlate (r = 0.99; see Additional file 1: Table 14). H correlates with Fe proportion by a correlation coefficient of 0.70 and with Ca with the one of 0.71. For E, correlation factors with Fe and Ca of 0.69 are calculated. For Na, Mg, Si, P, S, Cl, and K, small correlation coefficients are determined. For all treated teeth pooled together, H correlates with E (r = 0.94; see Additional file 1: Table 15). H correlates with the Fe proportion by a correlation coefficient of 0.77, but with Ca only with the one of 0.09. E and Fe correlate with the factor of 0.85. Ca and H are related by a correlation factor of 0.10. For Na, Mg, Si, P, S, Cl, and K, very small correlation coefficients are determined.

#### Sorted to treatment and tooth type

*Central tooth, native*. H and E highly correlate (r = 1.00; see Additional file 1: Table 16). H and E correlate less with Ca (r = 0.32). All other determined correlation coefficients are very small. H, E, Fe, Ca, dry breaking force, and wet breaking force highly correlate: the highest correlation coefficient (1.00) is detected between H and E and the lowest one (0.97) between Ca and Fe (see Additional file 1: Table 25).

*Lateral tooth I, native*. H highly correlates with E (r = 1.00; see Additional file 1: Table 17). H and E correlate less with Ca (r = 0.28). All other determined correlation coefficients are very small. H, E, Ca, dry breaking force, and wet breaking force highly correlate with each other (see Additional file 1: Table 26). Fe correlates with all other parameters with relatively low correlation coefficients (0.38–0.49).

*Lateral tooth II, native*. H and E are highly correlated with each other (r = 0.99; see Additional file 1: Table 18). Both H and E are also highly correlated with Fe (r of H = 0.79 and of E = 0.80) and Ca (r of H or E = 0.73). They are less correlated with P (r of H = 0.29 and of E = 0.30). H, E, Fe, Ca, dry breaking force, and wet breaking force highly correlate; the highest coefficient (1.00) is detected for H and E and the lowest ones between Ca and wet breaking force (0.76) and between Ca and Fe (0.76) (see Additional file 1: Table 27). All other determined correlation coefficients are very small.

*Marginal tooth, native.* H and E are highly correlated with each other (r = 0.99; see Additional file 1: Table 19). Both H and E are only slightly correlated with Ca (r of H = 0.23 and E = 0.24). H, E, Ca, dry breaking force, wet breaking force, dry breaking stress, and wet breaking stress highly correlate; the highest coefficient (1.00) is detected for H and E and the lowest ones between Ca and wet breaking force (0.72) and between H and wet breaking stress (0.76) (see Additional file 1: Table 28). Fe negatively correlates with each parameter. All other determined correlation coefficients are very small.

*Central tooth, treated*. H and E are highly correlated with each other (r = 0.99; see Additional file 1: Table 20). H, E, Ca, dry breaking force, and wet breaking force are highly correlated (see Additional file 1: Table 29). Fe negatively correlates with each parameter. All other correlation coefficients are very small.

*Lateral tooth I, treated*. H and E are highly correlated with each other (r = 0.99; see Additional file 1: Table 21). H, E, dry breaking force, and wet breaking force highly are correlated (see Additional file 1: Table 30). Ca negatively correlates with each parameter: with H, E, dry breaking force, and wet breaking force at low negative coefficients (-0.17 – -0.21) and with Fe with a high negative coefficient (-0.94). Fe correlates with all parameters with a low positive coefficient (0.48–0.52). All other correlation coefficients are very small.

*Lateral tooth II, treated*. H and E are highly correlated with each other (r = 0.92; see Additional file 1: Table 22). Both E and H are highly correlated with Fe (r of H = 0.82 and of E = 0.94). H, E, Fe, Ca, dry breaking force, and wet breaking force are highly correlated: the highest coefficient (1.00) is detected for H and dry breaking stress and the lowest one between Ca and H (0.86) (see Additional file 1: Table 31). All other correlation coefficients are very small.

*Marginal tooth, treated*. H and E are highly correlated with each other (r = 0.92; see Additional file 1: Table 23). H, E, Ca, dry breaking force, wet breaking force, dry breaking stress, and wet breaking stress are highly correlated: the highest coefficients (1.00) are detected between wet breaking force and E as well as between Ca and E; the lowest ones between dry breaking force and wet breaking stress (0.81) as well as between wet and dry breaking stress (0.81) (see Additional file 1: Table 32). Fe correlates negatively with each parameter. All other correlation coefficients are very small.

3-way ANOVA reveals that state (native or treated), tooth type, and radular zone all have significant effects on E and H values (p < 0.0001 for each, see Additional file 1: Fig. 10 and Additional file 1: Tables 33–34). 4-ANOVA detects that state (native or treated), tooth type, condition (dry or wet), and radular zone all have the same significant effect on E and H values (p < 0.0001 for each, see Additional file 1: Fig. 11 and Additional file 1: Tables 35–36). By PCA for marginals, centrals, and lateral teeth I no clusters are detected (see Additional file 1: Fig. 12).

## Discussion

### Elements detected

The mineralization and transformations during maturation in the dominant lateral tooth (termed lateral tooth II in this study) of iron and non-iron minerals were very well investigated in the last decades [see e.g. 12, 17, 41–43, 52–58]. It was previously determined that after the secretion and formation of the three-dimensional alpha chitin matrix with the associated proteins, which are possibly involved in the biomineralization process [59], various iron oxide phases are incorporated into the matrix [see also 60]. First ferrihydrite (Fe^3+^_10_O_14_(OH)_2_) is deposited in the junction zone and the margins of the tooth, before it also occurs in the leading part [12, 17, 39, 53, 55, 61]. During maturation it is replaced by iron oxides and iron oxyhydroxide minerals, usually by magnetite [62, 63], in the cusps’ leading part [8, 53, 56, 64, 65]. But in some taxa small proportions of lepidocrocite (γ-FeOOH) [11, 12, 39, 55, 66–71], goethite (alpha-FeOOH) [54, 72], or hydrated iron oxides (limonite) [73] were detected.

The distribution of magnetite seems to be taxon specific, in *Chiton* and *Acanthopleura* the posterior part of the dominant lateral tooth cusp is composed of magnetite whereas the anterior part is composed of apatite [11, 12, 55, 66, 70, 74–76]. In *Cryptochiton*, *Cryptoplax*, and *Chaetopleura* magnetite covers both posterior and anterior cusps’ surfaces [50, 56, 74, 77] whereas the core is either composed of amorphous iron phosphate [78, 79], apatite [8, 69, 72], or iron and/or magnesium phases [39, 71].

For *Lepidochitona,* due to our methodology, we are not capable of differentiating between the different iron oxide phases, since we were only able to determine the elemental distribution. But, [8] described that ferrihydrite deposition in the matrix is accompanied by colour change to red/brown and magnetite deposition by a change to glossy black. For *Lepidochitona*, we would carefully suggest that from row 8 to 9 ferrihydrite is present and from row 10 on magnetite is dominant (see Fig. 2a).

In *Lepidochitona,* the distribution of iron, observed in the dominant lateral tooth cusps, is rather similar to the pattern observed in *Cryptochiton*, *Cryptoplax*, and *Chaetopleura* [50, 56, 74, 77], as iron covers both the anterior and posterior parts of the cusp (see Additional file 1: Fig. 7a). Potentially, the occurrence of iron only in the posterior part is an apomorphic character of the Chitonida (for systematics of Polyplacophora, see [80]) and on anterior and posterior part is plesiomorphic. However, this awaits further investigations and a broader taxon-sampling.

In past studies about the radular tooth ontogeny, the dominant lateral tooth was in focus. As described by [81], ferritin is present in the haemolymph and delivered to the superior epithelia cells of the radular sac, which covers the dominant lateral tooth cusps. Afterwards a pore is penetrated into the cusp and stylus [76, 82]. Thus, the stylus canal may potentially serve as a biomineralization delivery pathway [51]. The first depositions of iron were detected in the ‘junction zone’, the part between cusp and basis [50, 51], and therefore it was postulated that this region serves as repository for ions, which migrate during ontogeny to the cusp [55, 69, 71, 76, 83]. For *Lepidochitona* (see Fig. 2)*,* we observed a slightly different pattern: in native radulae we found that the cusps are mineralized with iron first (0.38 ± 0.59 atomic % in row 6). In row 7, the cusps still contain the highest iron proportions (0.52 ± 0.38 atomic %), followed by the stylus (0.15 ± 0.05 atomic %), and finally the basis (0.14 ± 0.13 atomic %). These gradients in the iron distribution with the cusps, containing the highest proportions, followed by the stylus, and finally the basis continued until the tooth is mature in row 28 or 29 (see Fig. 2).

The ontogenetic changes of the iron proportions were previously documented for *Acanthopleura* [55, 69, 70]*, Ischnochiton, Onithochiton, Plaxiphora* [70], *Cryptoplax* [50], and *Clavarizona* [84]. In all of these taxa, iron proportions increase dramatically within very few rows in the magnetite region of the cusps and the junction zone. The junction zone sometimes loses its iron content during ontogeny again, whereas the anterior core region, the central/posterior core region, and the tooth basis rather increase their iron content gradually. For *Lepidochitona,* due to the small dimensions of these teeth and our EDX methodology, which involved rather larger areas, we were not capable of clearly differentiating between the magnetite region, the anterior core region, and the central/posterior core region [terms from 70]. Our points of measurements rather cover areas of all three zones in the cusp altogether. During ontogeny we determined a steady increase of iron content in the cusps (see Fig. 2). The stylus [which is equivalent to the ‘tooth basis’ in 70] and the basis also show a steady increase of iron, which is for the stylus in congruence with the previous results of [50] and [70].

In previous studies, iron contents of mature dominant lateral teeth were determined for (1) *Acanthopleura* with 59.2 weight % [11] or 62 weight % [55, 70] or few weight % [69], (2) *Plaxiphora* with 86.6 weight % [11] and 17–27 weight % or 66 weight % [70], (3) *Cryptochiton* 51.8 weight % [52] or up to 69 weight % [17], (4) *Ischnochiton* with 62 weight % [70], (5) *Onithochiton* with 66 weight % [70], (6) *Cryptoplax* with ~ 90 weight % in the cap, ~ 30 weight % in the core, junction zone, and basis [50], and (7) *Chiton* with 97 weight % [61]. We detected for mature *Lepidochitona* teeth iron proportions of ~ 30 atomic % in the cusps (see Fig. 2). However, it is rather difficult to compare percentages between studies, since in some publications weight percentages were studied, whereas here atomic ratios were determined. Besides, methodology, sample preparation, and the analysed sample itself (whole radula or individual radular parts) strongly vary between individual studies.

The cores of the dominant lateral tooth, the region underneath the iron-containing tooth caps, can contain, besides of the organic matrix being present in the mineralized parts of teeth [12], some amount of phosphorus [50, 52, 70, 71, 85] as iron phosphate [39, 78, 79] or as apatitic calcium phosphate [8, 69, 71, 72, 74, 86], magnesium [70, 71], and fluorine related to calcium [66, 86, 87]. It was reported that this tooth part is the least mineralized one in ontogeny [41]. In the posterior core region, calcium (max. ~ 30 weight %) and phosphorus proportions (max. ~ 18 weight %) increase dramatically within few rows, whereas calcium (max. ~ 30 weight %) and phosphorus (max. ~ 20 weight %) content in the anterior core region increases gradually during ontogeny in *Acanthopleura* [69, 70] and *Onithochiton* [70]. In *Acanthopleura* and *Onithochiton,* calcium and phosphorus content also increase dramatically within few rows in the anterior core region, whereas magnesium content of all tooth areas, calcium and phosphorus content of the basis (termed here ‘stylus’) of *Acanthopleura*, the anterior core region, the central/posterior core region, and the basis (termed here ‘stylus’) of *Ischnochiton* and *Plaxiphora* increase their content gradually [70]. Additionally, silicon was previously detected in the core, where the tooth also contains iron and phosphorus [8, 50, 70, 74]. In the junction zone, iron, phosphorus, and calcium were detected in higher proportions for *Acanthopleura*, *Ischnochiton*, *Onithochiton*, and *Plaxiphora* early in ontogeny (from row 8 on) [70] and at very low proportions before teeth become orange in colour [50]. Sulphur, deposited in ontogeny earlier than iron (before the onset of mineralization) in the junction zone and probably responsible for the yellow colour of teeth, was also previously detected [50]. It seems to be associated with the appearance of proteins and the tanning of the organic matrix [88]. Additionally, [84] detected zinc, potassium, fluorine, sulphur, sodium, and chlorine in radular segments of *Clavarizona*, [11] – calcium, phosphorus, magnesium, sulphur, sodium, zinc, potassium, aluminium, copper, and silicon in radulae of *Acanthopleura* and *Plaxiphora,* and [50] – magnesium (with max. ~ 5.5 weight % in the basis), potassium (with max. ~ 1.0 weight % in the basis), sodium (with max. ~ 2.0 weight % in the basis), silicon (with max. ~ 1.0 weight % in the basis), aluminium (with max. ~ 0.5 weight % in the basis), and sulphur (with max. ~ 0.8 weight % in the junction zone) in *Cryptoplax*. Calcium of ~ 5 atomic % was detected in the cusps of mature dominant lateral teeth of *Lepidochitona* (see Fig. 2): proportions decrease gradually from cusp across stylus to basis. All other elements occurred only in very small proportions. This altogether highlights that species can differ in their radular tooth elemental composition which could potentially be used as tool for taxonomy [70].

For the central, lateral teeth I, marginal teeth, no gradients in iron, calcium, or any other inorganic components could be detected (see Fig. 2), thus these teeth are regarded as non-mineralized, similar to most gastropods and limpets [89–91].

By comparing treated and native radulae, we found that iron seems to be tightly bonded within *Lepidochitona* radular teeth, as it was not washed out by EDTA, whereas calcium and all trace elements (Te) do not seem to be tightly bonded (see Fig. 4 and Additional file 1: Fig. 1). In the native specimens, reduction in the calcium proportion from row 25 on was detected (see Fig. 2). Potentially, under native conditions, calcium is washed out by surrounding fluids, either by passive diffusion or possibly by the salivary fluids. However, this awaits further investigations.

### Young’s modulus and hardness

Biological materials are generally composites displaying material heterogeneities or property gradients, which are important for the function of particular structure due to improved load bearing capacity or contact damage resistance [for comprehensive reviews, see 92, 93]. The parameter Young’s modulus (E) indicates the stiffness of a solid material and describes the relationship between tensile stress and axial strain. It correlates with the ability of the material (and structure) to transmit force, which is important to understand the puncturing behaviour and failure resistance [for comprehensive review on puncture mechanics, see 94]. The hardness (H) is the measure of the resistance to local plastic deformation induced by indentation or abrasion.

In molluscs, the ontogenetic change in these parameters (E and H) was well investigated for the dominant teeth of *Patella* (Gastropoda). Here, the hardness increases dramatically from row 60 to 110 (~ 150 rows in total) [44, 45]. For *Lepidochitona,* we observed a rather gradual increase in hardness and Young’s modulus for most tooth parts. Only in the styli of the lateral teeth II, their E and H values increased rather exponentially from row 15 to row 22.

Overall, hardness and Young’s modulus values varied between studies and molluscan taxa. [19] analysed the mature dominant lateral tooth regions by nanoindentation in *Cryptochiton* and revealed that the leading edge has a hardness of 10.2–10.4 GPa and a Young’s modulus of 128.5–130.8 GPa, the trailing edge: H = 7.5–8.2 GPa and E = 97.6–114.8 GPa, and the core: H = 1.5–1.6 GPa and E = 28.6–29.4 GPa. [95] determined H = 3.6 GPa and E = 86 GPa for the core, comparable to vertebrate enamel [96]. For mature limpet tooth cusps (Gastropoda), E = 16 GPa in dry state and E = 8 GPa in wet state was measured by nanoindentation for *Megathura* [22]. However, for *Patella* cusps, E = 120 (up to E = 140 GPa) and H = 4.9 GPa was reported [18, 20]. For paludomid gastropods, E = 8 GPa and H = 0.4 GPa were measured for the tooth cusps [23, 26, 29]. For the stylus of polyplacophoran teeth, H = 0.2–1.8 GPa and E = 7–30 GPa [95] or E values from 18 GPa (for upper stylus) to 12 GPa (basis) [97] were previously determined. Here the walls of the stylus canal and the core of the upper stylus were rather soft and flexible, whereas the outer parts were stiffer and harder [95, 97]. [98] identified a hardness of 0.6 GPa and a Young’s modulus of 10.5 GPa for the stylus leading edge. The stylus became first softer (H: 0.55 GPa, E: 9.5 GPa) and then again harder and stiffer (H: 0.60–0.65 GPa, E: 11–12 GPa) towards the stylus canal. The stylus was hardest and stiffest at its outer layer of the trailing edge (H: 0.6–0.7 GPa, E: 12–14 GPa); both parameters decreased around the stylus canal (H: 0.55 GPa, E: 11 GPa) [98].

For *Lepidochitona,* we detected graded values of hardness and Young’s modulus for every individual tooth type (see Fig. 4), which is in congruence with the previous studies on Polyplacophora, but also with analyses of radular teeth from paludomid gastropod species that forage on algae attached to rocks [23, 26, 29]. The cusps were always the stiffest and hardest elements, followed by the stylus, and finally the basis (see Fig. 4). This probably enables the cusps to puncture or interact with the ingesta with possible formation of local stress. The tooth styli and bases enable the avoidance of structural failure or heavy abrasive wear, as it was observed for paludomid gastropods [23, 26, 29]. Due to the smallness of the analyzed structures we were not able to differentiate between the core and the cusp edges in *Lepidochitona*, but values of the mature cusps of the dominant lateral teeth are comparable to cores of *Cryptochiton* [19] and the values of the styli are comparable to the values detected in other Polyplacophora [95, 97, 98]*.* The H and E values of the central, lateral teeth I, and marginal teeth (see Fig. 4 and Additional file 1: Fig. 1) are comparable to the values detected for the limpet *Megathura* [22].

### Breaking force and stress

For proper functioning, failures of biological structures must be avoided or reduced and studies on various biological structures depict the multiple origins of failure prevention [for comprehensive review, see 93]. As biological structures are adapted to certain functional loads, the analysis of forces leading to the structural failure could lead to the detection of e.g. functional specialisations.

For radulae, mechanisms contributing to the avoidance of structural failure were already in focus of research [e.g. 25, 35, 36, 99, 100]. A proper stress distribution, reducing high local stress, is probably enabled by the inner structure of the tooth, i.e. fibre orientation [48, 100], and the radular membrane itself [2, 25, 100, 101], as the wet membrane enables a higher bending capability of the tooth [25, 27].

Teeth also contribute to the prevention of failure by their mechanical behaviour, which is based on their individual morphology, arrangement in particular arrays, mechanical properties, and water content. Tooth morphology can enable the tooth to rely on adjacent teeth of the adjacent rows or to bend and slip away leading to the resistance of higher forces, which were previously termed ‘collective effect’ [25, 27]. The strong ability to bend, enabling a higher range of motion for tooth cusps, including the ability to deform and twist, when shear force is applied, was observed for long, slender, and thin teeth of paludomid gastropods [24, 25, 27, 34] and was also observed for the marginals, lateral teeth I, and centrals of *Lepidochitona* (see Additional file 1: Fig. 3)*.* However, this capability was only possible, when teeth were loaded under wet condition (see Additional file 1: Fig. 3). In dry condition, the thin teeth broke at their bases or stylus (see Additional file 1: Fig. 3). The wet lateral teeth II of *Lepidochitona* were also capable of bending [see also 95]; here the basis and stylus bent in contrast to the stiff and hard iron-containing cusp, leading to structural failure underneath the junction zone (see Additional file 1: Fig. 2b and Additional file 1: Fig. 3). The lateral teeth II in *Lepidochitona* were the only teeth that were capable of relying on the stylus of the adjacent lateral tooth II, resulting in the resistance to high forces (see Fig. 3). This ability was, however, not as pronounced as in paludomid gastropods [25, 27]. Under dry condition, lateral teeth II were not capable of bending and they break, when loaded, directly at the junction zone (see Additional file 1: Fig. 2b).

A stiffer part (higher E) of the tooth rather transfers forces, when e.g. interacting with the ingesta, whereas more flexible regions (lower E) enable the structure to bend. Previous breaking stress experiments on paludomid gastropods revealed that tooth failure usually occurred at the softest and most flexible part of the tooth (stylus and basis), whereas the hardest and stiffest parts (cusps) were not as prone to failure under applied shear force. The same pattern was also observed for *Lepidochitona* as teeth usually broke at their soft and flexible parts (see Additional file 1: Fig. 2 and Additional file 1: Fig. 3).

The H and E values of wet biological materials are lower than those of dry ones and additionally dry materials have lower fracture toughness. This was also previously reported for chiton radular teeth with wet teeth having 15% reduction in Young’s modulus and hardness [17]. However, previous breaking stress experiments on teeth of paludomid gastropods showed that wet teeth were capable of resisting higher forces and stresses than dry ones due to the increased flexibility of teeth and membrane [25, 27]. This distributed the stress from the tooth cusps to the radular membrane [2, 13, 21, 31–33, 98, 102]. The same pattern was observed in *Lepidochitona,* as wet radular structures were capable of resisting higher forces and stresses than the dry ones (see Fig. 3 and Additional file 1: Fig. 2).

*Lepidochitona* teeth (see Fig. 3) were capable of resisting higher forces than teeth of paludomid gastropods [25, 27]. Here the wet and mature central teeth of the species, foraging on algae attached to stone, showed the highest degree of collective effect, allowing the teeth to resist 754 ± 406.62 mN. The wet and mature lateral teeth II of *Lepidochitona* resisted to 1150 ± 143 mN, whereas the wet and mature marginals, centrals, and lateral teeth I resisted to much lower forces (Ct: 379 ± 75 mN; Lt I: 360 ± 134 mN; Mt: 94 ± 18 mN). These latter values were comparable to the breaking forces of wet and mature teeth of paludomid species foraging on mixed feeding substrate (plant surface, sandy surfaces, and occasionally on rocky surfaces) [27]. We would, thus, assume that centrals, lateral teeth I, and marginals of *Lepidochitona* are capable of occasional, but not regular interaction with the solid feeding substrate (rock).

### Origins of the mechanical property gradients and the behaviour

In biological materials, functional gradients and heterogeneities can have their origin in geometry, elemental composition, and/or structure [for comprehensive review on the origins of gradients and heterogeneities, see 92]. In the dominant lateral teeth of chitons and limpets the distribution of the inorganic components and the architecture of the organic components were found to determine material property gradients [17, 19, 56, 95, 97, 98, 103].

The here detected values of E and H in the dominant lateral teeth (lateral teeth II) of *Lepidochitona* highly correlated to the iron and the calcium proportions (see Additional file 1: Table 18 and Additional file 1: Fig. 8). The relationship between hardness and iron content was previously described for limpet teeth [20, 44–46] and for chiton teeth [17, 19, 56, 98], but here for *Lepidochitona* we were here able to determine correlation coefficients. But we however think, that organic components and degree of tanning had a high influence on the mechanical properties as well. Thus, the property gradients of the lateral teeth II probably had their origin in the combination of iron, calcium, and organic substances, as it was described for *Cryptochiton* teeth [19, 56, 97]. In limpets, hardness seems to depend additionally on silicon content [44–46], but in *Lepidochitona* only small proportions of silicon were detected.

For the centrals, lateral teeth I, and marginal teeth, we detected smaller correlation coefficients between hardness, Young’s modulus, and the amount of calcium (r = 0.23–0.32) and no correlations between hardness, Young’s modulus, and the amount of iron (see Additional file 1: Tables 16, 17, 19). Thus, the measured gradients in H and E and the resulting mechanical behaviour of the teeth of *Lepidochitona* could be rather based on the radular organic components, as distinct folding or bounding conditions of the chitin due to tanning [4], a mixture of both, or/and fibre size, arrangement, distribution, and/or density [18, 19, 22, 67, 77, 95, 103–105]. As the fibre orientation contributes highly to the mechanical behaviour of teeth and to the self-sharpening effect [46, 103, 105–107], this should be investigated for *Lepidochitona* in the future.

For *Lepidochitona,* we demonstrated high correlation coefficients between the breaking force of the central teeth (and the lateral teeth II) and the mean iron and the mean calcium contents (see Additional file 1: Tables 25 and 27). The breaking force of the lateral teeth I strongly correlated with the mean calcium content and less strong with mean iron content (see Additional file 1: Table 26). The breaking force of the marginal teeth strongly correlated with the mean calcium content (see Additional file 1: Table 28). For the breaking stress similar patterns were observed. Thus, calcium content seemed to have a high influence on the biomechanics of all teeth. In breaking stress experiments, we observed that wet and treated radulae resisted to higher forces and stresses (see Fig. 3 and Additional file 1: Fig. 2). This indicates that the degree of mineralization with calcium hindered the tooth from bending, but the reduction of this element enabled a higher collective effect under wet conditions. A very high bending amplitude was also detected for the unmineralized teeth of paludomid gastropods [25, 27].

This indicates, for the lateral teeth II, that the tooth material is a compromise between the ability to bend (and to initiate a collective effect) and the ability to transfer forces while puncturing the ingesta or scratching across it (which is probably enabled by the E and H values originated from the calcium content).

### Function of radular structures

During foraging, a rotatory scraping action of the dominant lateral teeth, followed by inward sweeping action, enabled by the bending of the radula, can be observed in chitons [1, 30, 97, 108, 109]. For this purpose, the membrane must be flexible and capable of changing its shape, but it must also be strong and tough, to avoid failure during this action [see 43, 97]. This is in congruence with our results from the breaking stress experiments in *Lepidochitona* and paludomid gastropods [25, 27], where we observed that the wet membrane is flexible enough to enable the bending of embedded teeth and additionally contributes to stress distribution. The dominant lateral tooth cusps possess caps of exceptional hardness, reducing wear and contributing to a self-sharpening effect [17, 19, 47, 105, 107; see also 103 for limpets]. This, together with their high ability to resist forces (documented here) and with the previously documented foraging behaviour and observed rotating interaction of the dominant lateral teeth with the ingesta surface [1, 30, 109], depicts that these teeth loosen the food from the hard surface and transport them towards the mouth [43, 97]. The underlain softer core of the tooth seems to serve as a shock absorption and toughening mechanism [105].

It was previously reported that due to the specific shape of the cusp and the gradients in hardness and Young’s modulus across the tooth, tensile stress is concentrated on the leading edge and reduced in the trailing edge, reducing the ability of the tooth to bend and thus reduce the failure of the sharp tip of the tooth [19, 47]. It was also reported that the stylus enables the sweeping action during feeding [95, 97], orients the tooth to the ingesta [47], but also transfers force from the basis to the tooth cusp [98] leading to the reduction of structural failure [97]. These mechanical behaviours, which are based on the graded mechanical property gradients, were also confirmed by breaking stress experiments in *Lepidochitona*.

The centrals of *Lepidochitona* can resist highest forces and stresses (see Additional file 1: Table 4 and Additional file 1: Table 7), followed by lateral teeth I, and finally marginals, even though marginal teeth have (slightly) higher E and H values than lateral teeth I and marginals (see Additional file 1: Fig. 1 and Additional file 1: Table 10). This leads to the conclusion, that centrals potentially interact more frequently with the ingesta surface, followed by the lateral teeth I. Since the marginals can resist to significantly lesser forces and stresses (see Additional file 1: Table 4 and Additional file 1: Table 7), these teeth may be potentially responsible for collecting the loosened particles in the sweeping motion. We also detected material property gradients in every type of teeth, with the cusps always the hardest and stiffest tooth parts, followed by the styli, and finally the bases (see Fig. 4). This may lead to the conclusion that the cusps of these teeth probably also interact, or even puncture, the ingesta with the possible formation of local stress at the cusps, whereas the softer and flexible tooth styli and bases allow the avoidance of structural failure or heavy tooth wear during scratching on the hard substrate.

## Conclusion

We here provide comprehensive data on the mechanical properties (hardness, Young’s modulus) of all radular teeth from the chiton *Lepidochitona cinerea*. By employing energy-dispersive X-ray spectroscopy we could analyse the radular tooth elemental composition and correlate the proportions of calcium and iron to the mechanical properties. We also tested the mechanical behaviour of the teeth in dry and wet condition, which altogether allowed conclusions about tooth function. Knowledge about the relationship of all parameters mentioned contributed to our understanding on the origins of mechanical property gradients and the processes reducing structural failure in radular teeth.

## Methods

### Specimen studied and dissection

Adult individuals were collected at the coast of the North Sea, at Husum in Germany, in summer 2019, shortly boiled, and preserved in 70% ethanol. They were identified as *Lepidochitona cinerea* (Linnaeus, 1767) [Polyplacophora: Chitonida: Ischnochitonidae] by employing the relevant literature [110]. The nomenclature and systematic position were checked using *molluscabase.org*; specimens were inventoried (collection number: ZMH 154.653) in the malacological collection of the Zoologisches Museum Hamburg (ZMH), Germany, which is part of the Leibniz Institute for the Analysis of Biodiversity Change (LIB).

Overall, 20 adult specimens were used for this study. To receive the radula from the body, each specimen was placed on its dorsal side, a paramedian cut was performed on the ventral side of the head and the radula was carefully extracted by tweezers. Radulae were then manually freed from surrounding tissue always grabbing the radula by the alary processus to avoid tooth and membrane fractures.

### Treatment

To test the influence of calcium, silicon, etc. on the mechanical properties, such as hardness, elasticity (Young’s modulus), and breaking stress, 10 radulae were treated for seven days with ethylene diamine tetra acetic acid (EDTA). We followed the protocol of [111], using ethylene diamine tetra acetic acid disodium salt dehydrate (Carl Roth GmbH + Co. KG, Karlsruhe, Germany). EDTA is known for binding ions of e.g. magnesium and calcium and is thus used for e.g. decalcification of bones in histology. Radulae were washed with distilled water afterwards.

### Scanning electron microscopy (SEM) and light microscopy

Scanning electron microscopy (SEM) images of the working zone (Fig. 1a) and habitus images (Fig. 1d) were taken from literature [109], since they studied specimens from the same lot. The teeth were categorized into different tooth types according to their morphology and arrangement on the radula (central tooth, lateral tooth I, lateral tooth II [= dominant lateral tooth], and marginal tooth). On both lateral tooth II and marginal tooth, three distinct regions (cusp, stylus, and basis) and for the smaller lateral tooth I and central tooth two regions were defined (cusp and stylus).

For light microscopy imaging, six radulae (three native, see Figs. 1g and 2a, and three treated ones = treated with EDTA, see Fig. 1h and Additional file 1: Fig. 5a) were cleaned in an ultrasonic bath for a few seconds, placed on a glass object slide, and left to dry under room conditions. Images were taken with magnifications between × 50 and × 700, employing the Keyence Digital Microscope VHX-7000 (KEYENCE, Neu-Isenburg, Germany) equipped with automatic stacking software. To achieve the highest possible resolution, radulae were documented in parts and later merged using Adobe Photoshop CS6 (Adobe Inc., San José, USA). Additionally, detailed images in specific depths of the lateral teeth were taken employing the optical microscope Leica DM2500 LED (Leica Camera AG, Wetzlar, Germany) to illustrate the irregularity and shift in tooth colour. Based on the general tooth colour and the presence of overlying epithelia, the tooth rows were either assigned to the radular sac (with overlain epithelia, cusps rather lighter), the mineralization zone (with overlain epithelia, cusps are rather darker due to more iron incrustations), or the working zone (without overlain epithelia).

### Elemental analysis and nanoindentation

Six radulae (three of native and three of treated condition) were arranged on glass object slides (Carl Roth, Karlsruhe, Germany) by double-sided adhesive tape along their longitudinal axis so that the marginal teeth of one side were directly attached to the bottom. The adjacent lateral teeth II were thus located above, followed by the lateral teeth I, central teeth, again lateral teeth I, lateral teeth II, and finally, on top, the marginal teeth from the other side. Each radula was then dried under ambient temperature for three days and afterwards surrounded by a small, metallic ring which ensured an almost parallel sample surface. Epoxy resin (RECKLI EPOXI WST, RECKLI GmbH, Herne, Germany) was filled into the metallic ring and left polymerizing at room temperature for three days (Young’s modulus of the polymerized epoxy is 1.2 ± 0.3 GPa; Young’s modulus received from testing five localities per sample). This specific epoxy was chosen since it does not infiltrate the teeth. Object slide and tape were removed and, to receive longitudinal sections of each tooth, the embedded radulae were polished until the marginal teeth were on display (controlled by examining the samples by microscope) using sandpapers of different roughness. Then samples were smoothed with aluminium oxide polishing powder suspension of 0.3 μm grainsize (PRESI GmbH, Hagen, Germany) on a polishing machine (Minitech 233/333, PRESI GmbH, Hagen, Germany).

After polishing, the samples were cleaned from the polishing powder by an ultrasonic bath lasting five minutes. Samples (Figs. 1b, Additional file 1: Fig. 7a and b) were then sputter-coated with platinum (5 nm layer). The elemental composition of specific areas of the embedded teeth was examined employing the SEM Zeiss LEO 1525 (One Zeiss Drive, Thornwood, New York, USA) equipped with an Octane Silicon Drift Detector (SDD) (micro analyses system TEAM, EDAX Inc., New Jersey, USA), always using an acceleration voltage of 20 keV and the same settings (e.g. lens opening, working distance, etc.). Before analyses, the detector was always calibrated with copper. Due to limitations of the used system, we could not quantify H (hydrogen); thus our results are semi-quantitative. We performed elemental mappings for test purposes for Fe (iron), O (oxygen), Ca (calcium), Mg (magnesium), P (phosphor), Si (silicon), S (sulphur), Cl (chlorine), K (potassium), F (fluorine), Na (sodium) (Fig. 1c, e, f), but for elements, rather present with lower proportions, this method is not sensitive enough (see e.g. calcium in Fig. 1f). We thus decided to focus here on elemental analyses of small areas (10–15 μm^2^) (Additional file 1: Fig. 7).

Overall, 4144 semi-quantitative EDX (EDS) measurements (for native radulae: 2074 and for treated radulae: 2070) were successfully performed. For every central and lateral tooth I, the two previously defined regions (stylus and cusp) and for every lateral tooth II and marginal tooth, the three areas (basis, stylus, and cusp) were investigated (Fig. 1b, Additional file 1: Fig. 7a and b). The proportion of the elements H (hydrogen), C (carbon), N (nitrogen), O, Pt (platinum), Al (aluminium), Ca, Na, Mg, Si, P, S, Cl, K, C, and Fe were detected and measured. We used the data of atomic ratio (atomic %) for this study. The values were received with two positions after the decimal point: lower proportions were not detectable with this method and given as 0.00. We did not analyse and discuss the following elements, as they are either the elemental basis of chitin (H, C, N, O), the coating (Pt), or of the polishing powder (Al, O). For some analyses or graphic depictions Na, Mg, Si, P, S, Cl, K, Ca, and Fe were summed to ‘all elements (Ae)’ and Na, Mg, Si, P, S, Cl, and K, as they were only sparsely detected, were summed to ‘trace elements (Te)’.

After EDX analysis the same samples were used for nanoindentation [for the detailed nanoindentation protocol see 23, 26, 29]. Here, hardness and Young’s modulus (E, elastic modulus) of the regions, which were previously analysed by EDX, were identified. A nanoindenter SA2 (MTS Nano Instrument, Oak Ridge, Tennessee, USA) equipped with a Berkovich indenter tip and a dynamic contact module (DCM) head, allowing to test materials with low contact stiffness as soft biological tissues, was employed. Hardness and the effective elastic modulus could be determined from force-distance curves by applying the continuous stiffness measurement technique [112]. As in past research on mechanical property testing by nanoindentation [22, 23, 26, 28, 29], a Poisson’s ratio of 0.3 was used. All tests were performed under normal room conditions (relative humidity 28–30%, temperature 22–24 °C). The position of every indent was controlled after each experiment under the microscopy as well as the run of every individual displacement into surface vs. Young’s modulus curve (to see if artefacts as e.g. surface roughness affected the results). E and H of the material was either determined at penetration depth of 600–700 nm (for lateral tooth II), or at 500–600 nm (for all other teeth), with about 30 values per indentation, which were collated to receive one H and one E mean value per indent. After this, every sample was again polished and smoothened until the next tooth type was on display. Then cleaning, EDX analyses, and nanoindentation were again performed. These steps were repeated until all teeth were measured. Overall, we received successfully data on E and H for 4139 tooth areas (for native radulae: 2072 and for treated radulae: 2067).

### Breaking force and breaking stress

For breaking force experiments overall eight radulae were used (four of native and four of treated condition). For testing in dry condition, we used two native and two treated radulae, and for testing under wet condition, we also used two native and two treated radulae. As described in detail in [25, 27], every radula was mounted on one microscope glass slide (Carl Roth, Karlsruhe, Germany) either with double sided adhesive tape (for experiments in dry condition) or by applying epoxy (RECKLI EPOXI WST, RECKLI GmbH, Herne, Germany) to both alary processus and both sides of the radular membrane (for experiments in wet condition). As mentioned above, this epoxy was chosen, because it does not infiltrate the teeth. Then teeth were carefully stroked into proposed feeding position. For experiment in dry condition the mounted radulae were left to dry for four hours at room temperature. For experiments in wet condition the epoxy was left for two days to polymerize; afterwards radulae were rehydrated by applying distilled water onto the radula. Water drops on teeth were removed by soaking it up with tissues before experiment.

Glass slides with the radulae were positioned under binocular microscope and a rounded steel needle (diameter: 0.4 mm), firmly attached to a force transducer FORT-1000 (World Precision Instruments, Sarasota, FL, USA), which was connected to an amplifier (Biopac System Inc., California, USA) and computer-based data acquisition and processing system (Acq Knowledge, Biopac Systems, Inc., version: 3.7.0.0, World Precision Instruments, Sarasota, Florida, USA), was pressed onto the individual tooth cusps by employing a remote-controlled micromanipulator (DC 3001R, World Precision Instruments Inc., Sarasota, Florida, USA). The needle was positioned on the concave part of every tooth cusps at 30° to the horizontal plane and moved onto the cusps until structural failure occurred. The forces needed to either crush or shear teeth were recorded and their maxima were calculated from the obtained force–time diagrams. Overall, we received data from 1301 individual breaking force curves (equals the quantity of broken teeth). Afterwards the broken radulae were documented with the Tabletop scanning electron microscope TM4000 Plus (Hitachi, Tokyo, Japan) and the types (e.g. crushing, rupture, breaking) and region of the structural failure (basis, stylus, cusps, etc.) were examined (Fig. 3a and b). Since the centrals and the lateral teeth I were either crushed or ripped, we were not able to measure their area of failure and to determine the breaking stress (breaking force divided by tooth cross sectional area). The lateral teeth II and the marginal teeth exhibited a rather plain breaking area, which could be measured. Here, the average breaking area was determined by documenting ten areas per tooth type and per radula using SEM. The obtained SEM images were imported into Adobe Illustrator CS 6 (Adobe Inc., San José, USA), here the breaking areas were outlined, and every outline was translated into an individual red area. By using the scale bar from SEM as reference a blue square area (in μm^2^) was also computed for every image. Then images were individually imported into Adobe Photoshop CS6 (Adobe Inc., San José, USA), here the quantity of blue and red pixels was read out. By accounting pixel quantity of the square with the pixel quantities for every broken tooth area, the area (in μm^2^) of failure and subsequently an average breaking area for every tooth type could be determined. Then breaking stress was calculated from the breaking force and the mean broken area for the corresponding tooth type.

### Statistical analyses

All statistical analyses were performed with JMP Pro, Version 14 (SAS Institute Inc., Cary, North Caroline, 1989–2007). Mean values and standard deviations were calculated and Shapiro–Wilk-W-tests for testing of normality were conducted. When data was non-normal distributed, a Kruskal–Wallis test was carried out. When the data was normal distributed, either a t test or ANOVA was performed to detect differences. Pairwise comparisons were conducted with Wilcoxon method (non-normal distribution) or Tukey–Kramer test (normal distribution). However, statistical analyses are difficult in some cases either as we do not have a high quantity of measurements or as the statistical test especially takes the variance into account while comparison. All correlations and 4-way ANOVA were also performed with JMP; relationships between parameters were here visualized. Gain or loss of E, H, Fe, Ca, Ae, Te from one row to the adjacent was calculated (e.g., Ca atomic % of tooth row 2 − Ca atomic % of tooth row 1 = gain or loss of Ca from row 1 to row 2). We set the highest proportion of e.g. Ca in whole radula to 100% to receive gain or loss of Ca in %. A PCA based on results from elemental analysis and mechanical tests was conducted for all teeth (without lateral teeth II), to detect potential similarities between native centrals, native lateral teeth I, native marginals, treated centrals, treated lateral teeth I, and treated marginals.

## Supplementary Information

**Additional file 1.** Additional figures, tables including raw data and statistics, correlation coefficients, relationships between parameters, and results from ANOVA and PCA are presented.

## Data Availability

All data is included in the supplementary file.
