# Supplementary material for: Ontogeny of the elemental composition and the biomechanics of radular teeth in the chiton Lepidochitona cinerea
Source: Front Zool. 2022 Jun 11;19:19. doi: 10.1186/s12983-022-00465-w (PMC9188181; doi:10.1186/s12983-022-00465-w)
Supplement: Supplementary file 1 — Additional file 1. Additional figures, tables including raw data and statistics, correlation coefficients, relationships between parameters, and results from ANOVA and PCA are presented. [file 12983_2022_465_MOESM1_ESM.pdf]

Supplementary data

Ontogeny of the elemental composition and the biomechanics of radular teeth in the chiton *Lepidochitona cinerea*

Wencke Krings[1,2,3*], Jan-Ole Brütt[1,2], Stanislav N. Gorb[3]

[1] Department of Behavioral Biology, Institute of Cell and Systems Biology of Animals, Universität Hamburg, Martin-Luther-King-Platz 3, 20146 Hamburg, Germany

[2] Department of Mammalogy and Palaeoanthropology, Leibniz Institute for the Analysis of Biodiversity Change, Martin-Luther-King-Platz 3, 20146 Hamburg, Germany

[3] Department of Functional Morphology and Biomechanics, Zoological Institute, Christian-Albrechts-Universität zu Kiel, Am Botanischen Garten 9, 24118 Kiel, Germany

*corresponding author: wencke.krings@uni-hamburg.de

1. Additional figures:

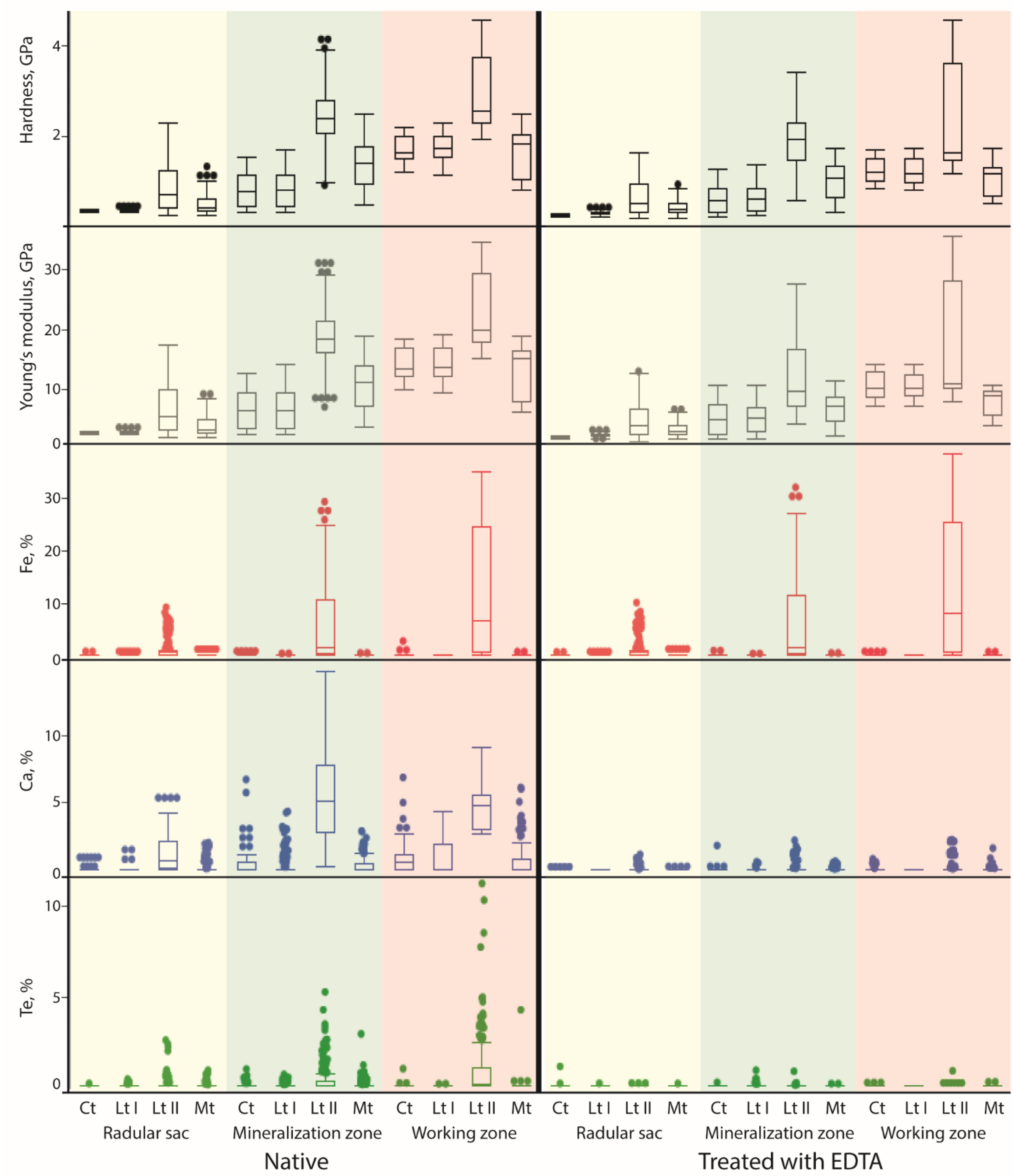

Supplementary Figure 1. For each tooth type (Ct, central tooth; Lt I, lateral tooth I; Lt II, lateral tooth II; Mt, marginal tooth) and each zone: Hardness, GPa, Young's moduli, GPa, and Fe, Ca, and Te ('trace elements') as proportion, %. Left side: native radulae, right side: treated radulae.

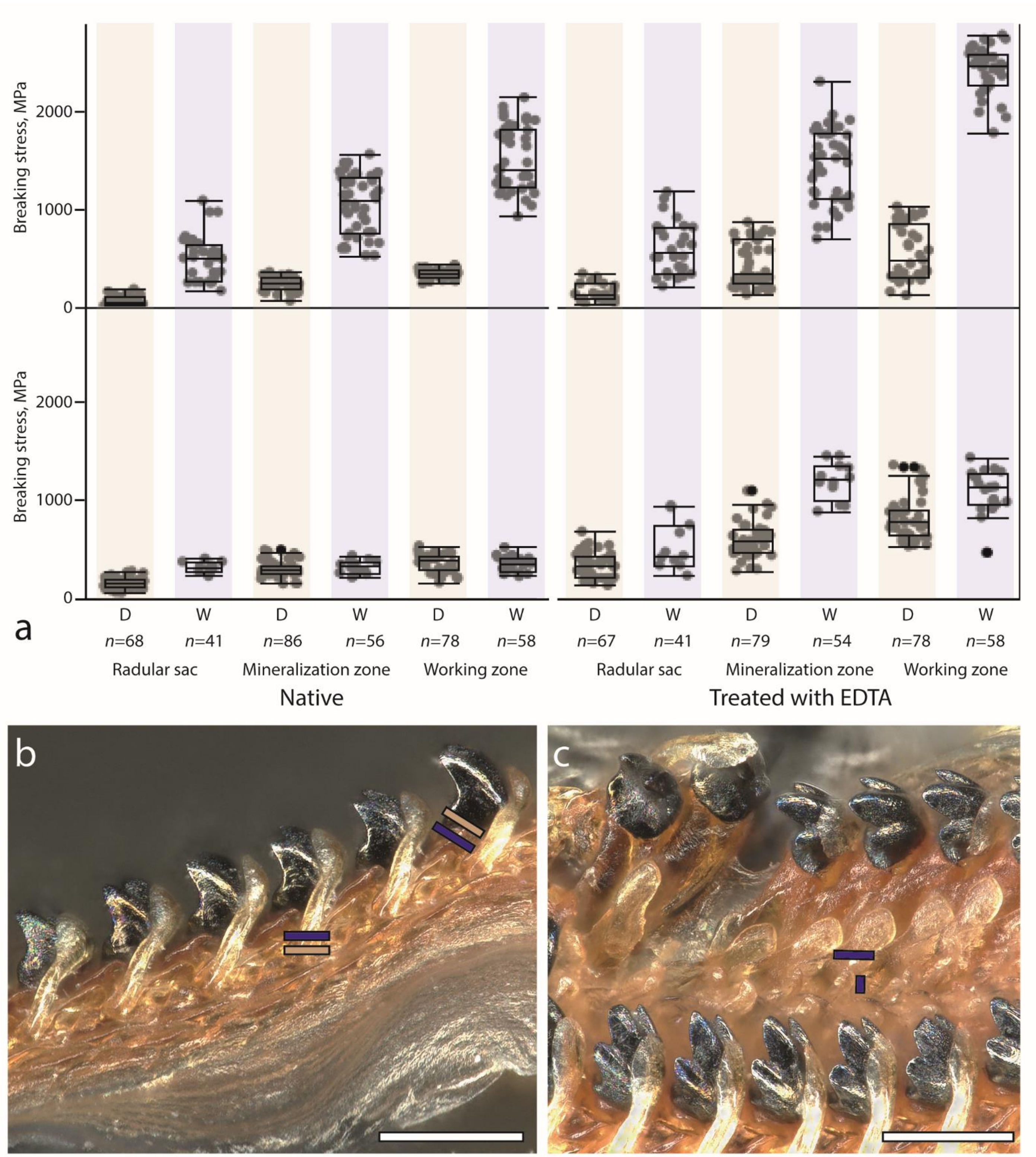

Supplementary Figure 2. a. Calculated breaking stress (breaking force/area), given in MPa, for each radular zone and condition (native dry, native wet, treated dry, treated wet) with quantity (*n*) of broken teeth that were used for calculation. Brown colour highlight the results obtained by testing dry teeth and blue the results obtained from testing wet teeth. b–c. Region of structural failure under dry (brown) or wet condition (blue). Scale bars: b–c = 100 µm. D, dry; W, wet.

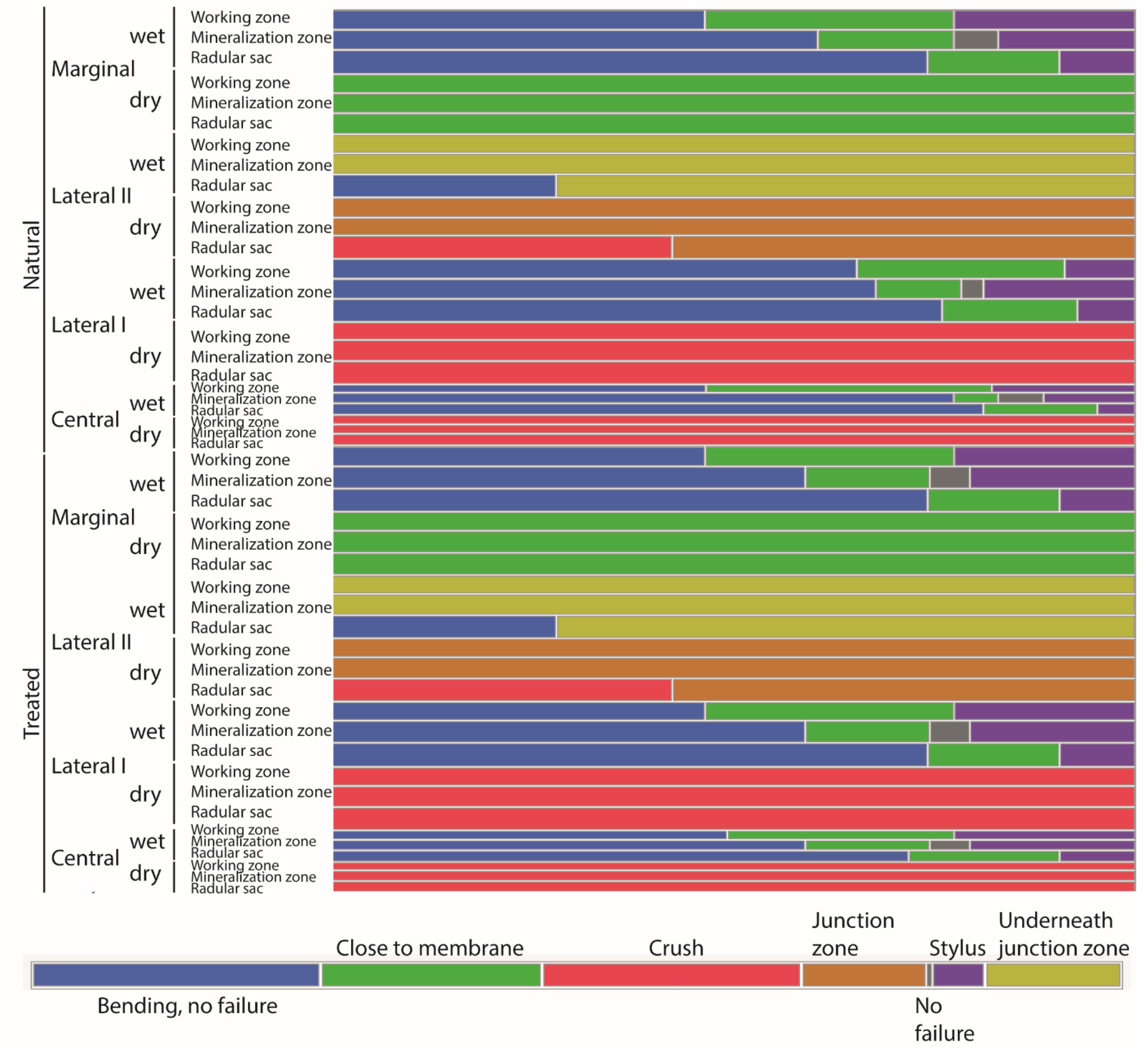

Supplementary Figure 3. Percentage of different failure types (sorted to the area, where failure occurred) of each tooth type during breaking stress experiments. Sorted to radular zone, native and treated, tested under dry or wet condition.

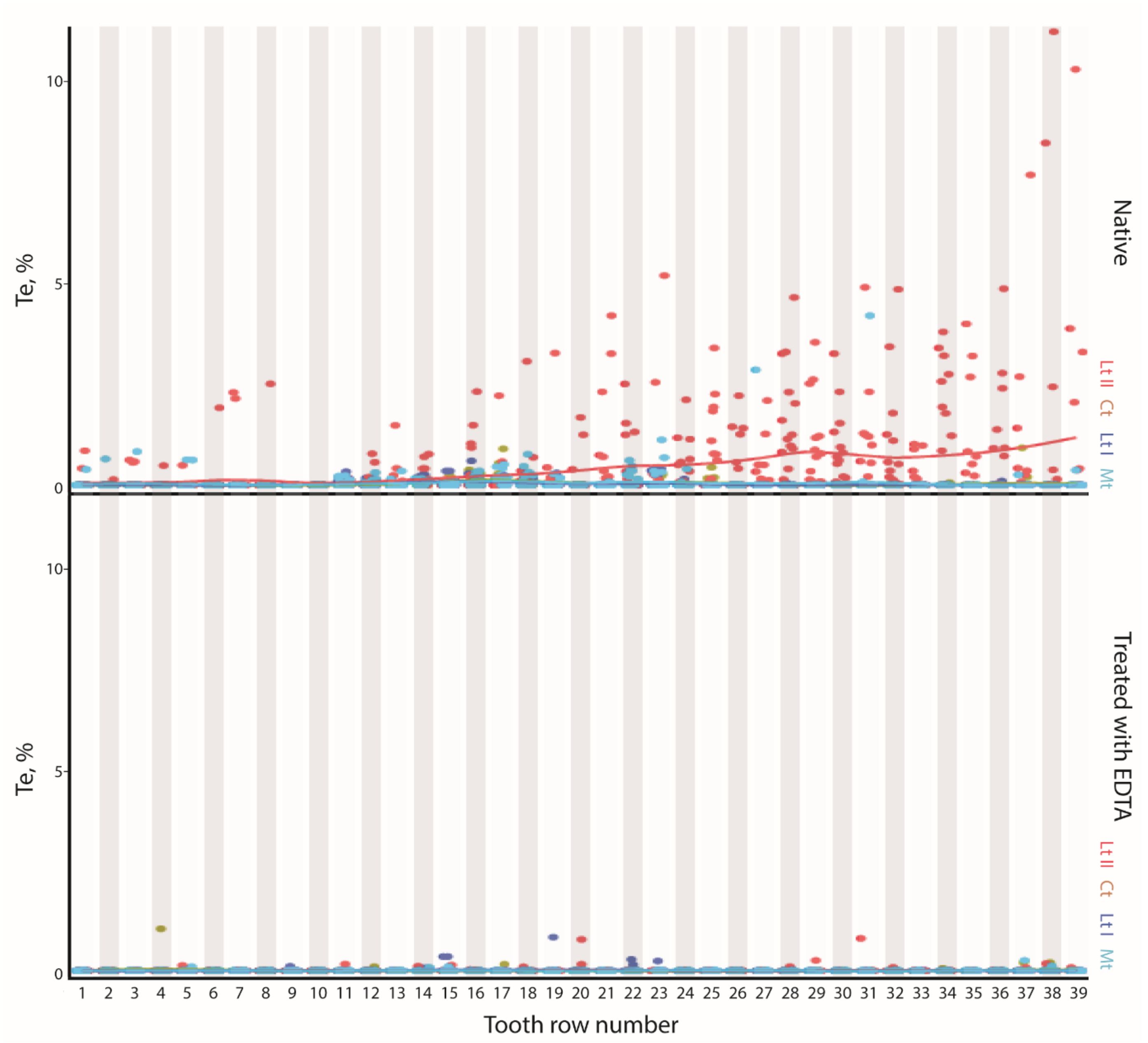

Supplementary Figure 4. Changes in proportion of Te ('trace elements'), %, during ontogeny for each tooth type (Ct, central tooth; Lt I, lateral tooth I; Lt II, lateral tooth II; Mt, marginal tooth); values and trend lines for native (above) and treated (below) radulae.

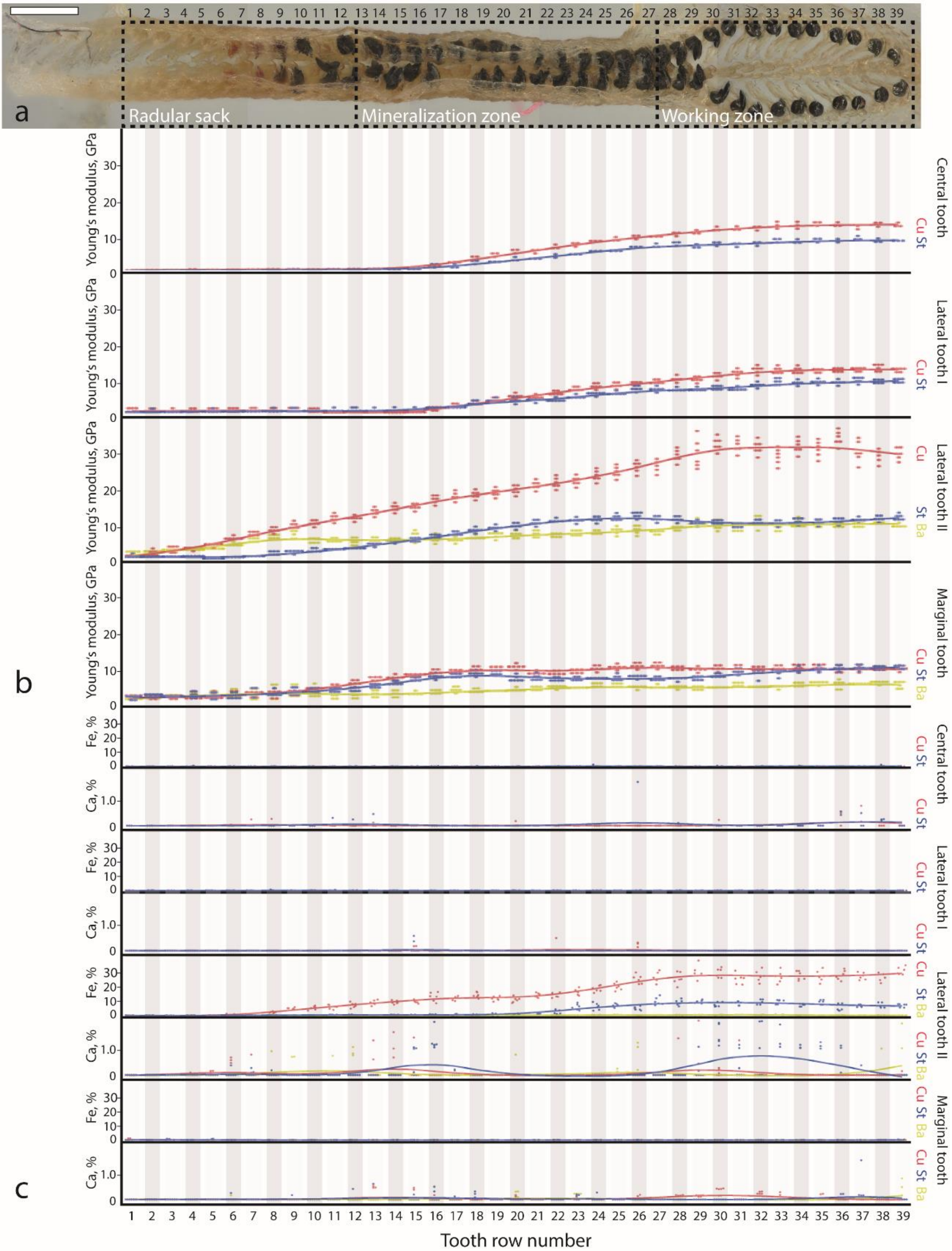

Supplementary Figure 5. EDX and nanoindentation data obtained from treated radulae (*n* = 3). a. Light microscopy images of one radula, tooth rows are numbered, and the zones (radular sac, mineralization zone, working zone) are defined. b. Changes in Young's modulus, GPa, during ontogeny for each tooth type and tooth part (Cu, cusp; St, stylus, Ba, basis). c. Changes in and Fe and Ca proportions (%) during ontogeny for each tooth type and tooth part. Dots show values measured and solid lines show trend lines based on the individual measurements. Scale bar: A = 200 µm.

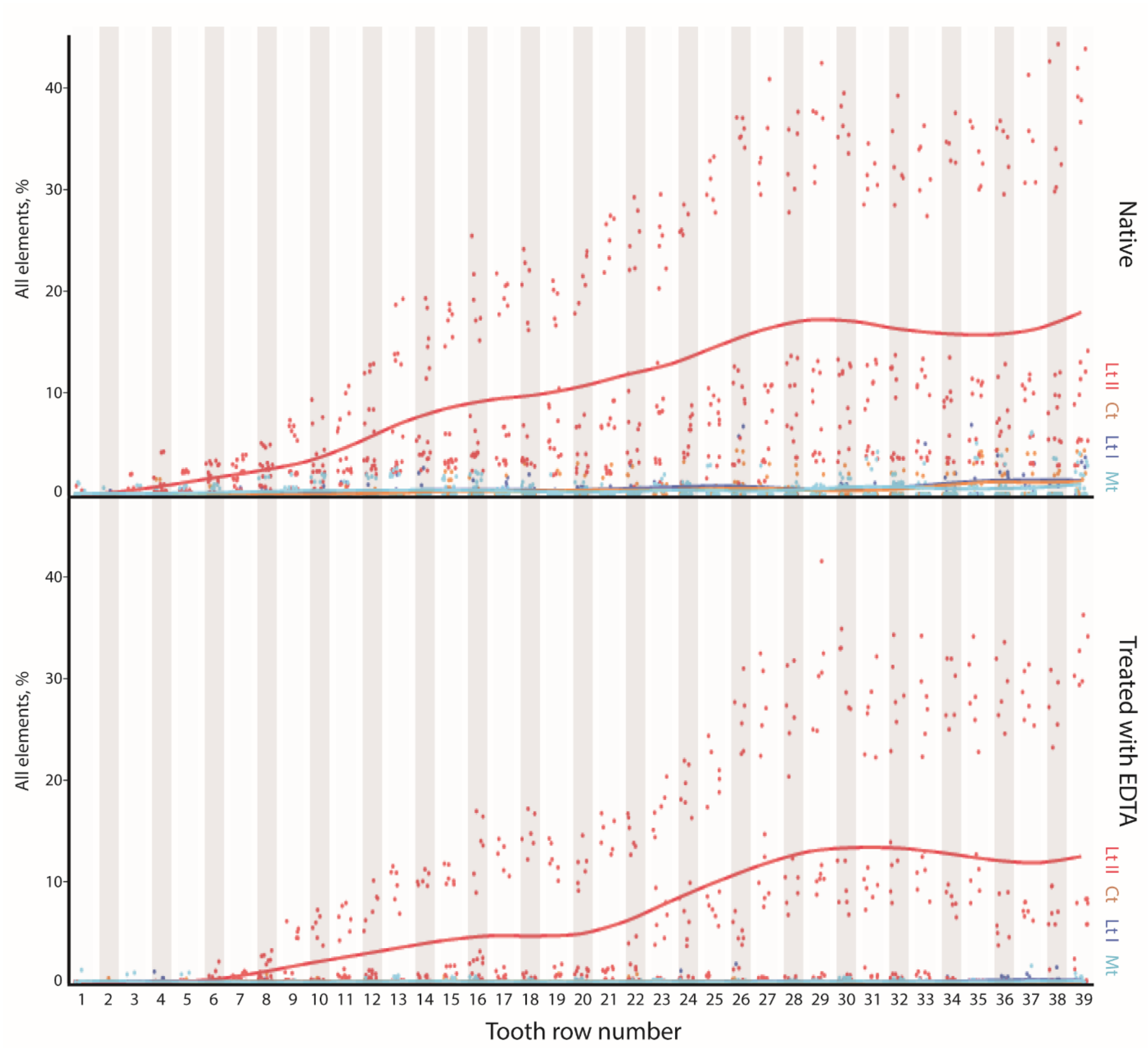

Supplementary Figure 6. Changes in proportion of all elements, %, during ontogeny for each tooth type (Ct, central tooth; Lt I, lateral tooth I; Lt II, lateral tooth II; Mt, marginal tooth); values and trend lines for native (above) and treated (below) radulae.

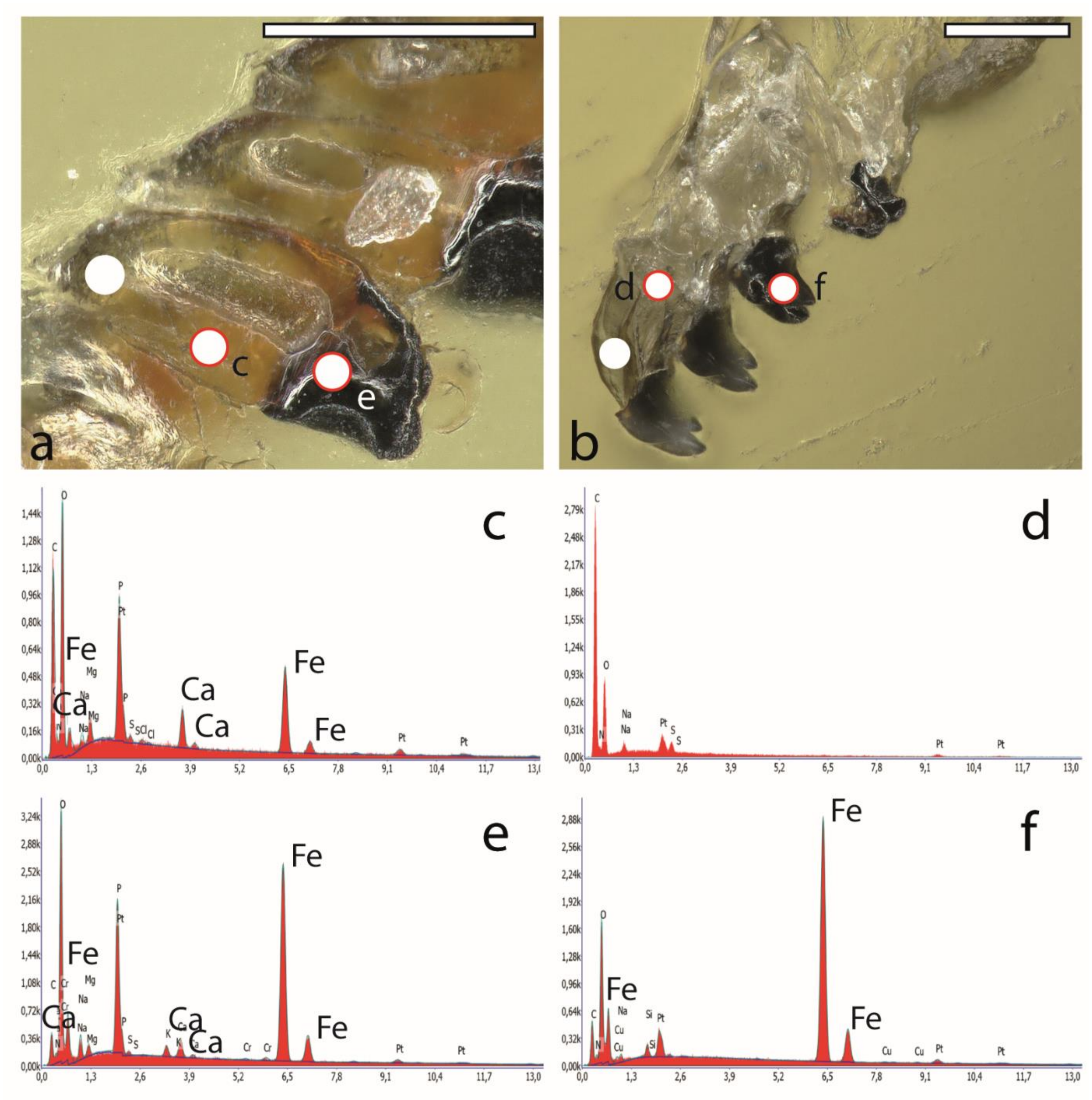

Supplementary Figure 7. Embedded and polished samples, images obtained by light microscopy, and corresponding EDX spectra (the circles indicate the area of EDX analysis: basis, stylus, cusp). a. Lateral teeth II, working zone of the native radula from specimen number 2. b. Lateral teeth II, working zone of the treated radula from specimen number 1. c. EDX spectrum of the native stylus. d. EDX spectrum of the treated basis. e. EDX spectrum of the native cusp. f. EDX spectrum of the treated cusp. Scale bars: a–b = 100 µm.

2. Tables including raw data and statistics:

Supplementary Table 1. For every individual whole radula. Mean, standard deviation (Sd), and quantity (*n*) of analysed tooth areas (by EDX and nanoindentation) or quantity of broken teeth (by breaking stress experiments) for the following parameters are given: (a) proportions, given in %, of Na, Mg, Si, P, S, Cl, K, Ca, Fe, and all elements. Differences between the individual radulae for each condition (native or treated) were tested by Kruskal-Wallis test (orange p-values = highly significant). (b) Young's modulus (E) and hardness (H), given in GPa. Differences between the individual radulae for each condition (native or treated) were tested by ANOVA (red p-values = significant). (c) Breaking force (Bf), given in mN, and breaking stress (Bs), given in MPa. Differences between the individual radulae for each condition (native dry, native wet, treated dry, or treated wet) were tested by t test (black p-values = not significant).

Parameter	Condition	Individual 1			Individual 2			Individual 3			Results from Kruskal-Wallis		
		Mean	Sd	*n*	Mean	Sd	*n*	Mean	Sd	*n*	ChiSquare	df	p-value
Na	Native	0.03	0.23	687	0.04	0.29	695	0.03	0.22	692	0.5871	2	0.7456
Mg	Native	0.02	0.14	687	0.02	0.15	695	0.02	0.15	692	0.9639	2	0.6176
Si	Native	0.00	0.01	687	0.00	0.01	695	0.00	0.00	692	9.5023	2	0.0086*
P	Native	0.09	0.47	687	0.07	0.44	695	0.09	0.09	692	1.1567	2	0.5608
S	Native	0.02	0.08	687	0.02	0.08	695	0.02	0.08	692	0.0997	2	0.9514
Cl	Native	0.01	0.05	687	0.00	0.02	695	0.00	0.03	692	0.6350	2	0.7280
K	Native	0.00	0.02	687	0.00	0.02	695	0.00	0.05	692	4.3273	2	0.1149
Ca	Native	1.55	2.33	687	1.53	2.40	695	1.51	2.39	692	0.1154	2	0.9439
Fe	Native	1.99	5.86	687	1.94	5.77	695	1.95	5.85	692	0.2894	2	0.8653
All elements	Native	3.69	7.93	687	3.63	7.95	695	3.62	7.91	692	0.0692	2	0.9660
Na	Treated	0.00	0.03	684	0.00	0.00	694	0.00	0.00	690	1.0093	2	0.6037
Mg	Treated	0.00	0.04	684	0.00	0.00	694	0.00	0.01	690	1.0093	2	0.6037
Si	Treated	0.00	0.00	684	0.00	0.00	694	0.00	0.00	690	1.9827	2	0.3711
P	Treated	0.00	0.04	684	0.00	0.00	694	0.00	0.00	690	4.0546	2	0.1317
S	Treated	0.00	0.01	684	0.00	0.00	694	0.00	0.01	690	4.7708	2	0.0921
Cl	Treated	0.00	0.01	684	0.00	0.01	694	0.00	0.02	690	0.4275	2	0.8076
K	Treated	0.00	0.01	684	0.00	0.02	694	0.00	0.01	690	0.6411	2	0.7258
Ca	Treated	0.05	0.22	684	0.05	0.22	694	0.06	0.25	690	0.5467	2	0.7608
Fe	Treated	2.13	6.17	684	2.17	6.25	694	2.20	6.39	690	0.5042	2	0.7772
All elements	Treated	2.18	6.20	684	2.23	6.27	694	2.26	6.42	690	0.8869	2	0.6418
											Results from ANOVA		
											F Ratio	df	p-value
H	Native	1.41	0.96	687	1.48	0.98	695	1.33	0.90	692	4.5207	2	0.0110*
E	Native	11.20	7.49	687	11.72	7.59	695	10.52	6.96	692	4.6125	2	0.0100*
H	Treated	1.05	0.80	684	1.11	0.83	694	1.00	0.77	690	3.5028	2	0.0303*
E	Treated	7.80	5.99	684	8.14	6.17	694	7.46	5.73	690	2.2726	2	0.1033
		Individual 4			Individual 5						Results from t test		
		Mean	Sd	*n*	Mean	Sd	*n*				t ratio	df	p-value
Bf	Native dry	174.51	207.49	223	160.68	196.00	224				-0.72472	443.3292	0.7655
Bf	Native wet	467.09	411.37	115	436.53	385.06	106				-0.5705	218.9456	0.7155
Bs	Native dry	277.64	116.07	112	240.35	110.21	120				-2.50584	226.6855	0.9935
Bs	Native wet	948.32	601.83	79	753.84	422.26	76				-2.33601	140.0989	0.9895
Bf	Treated dry	116.20	143.46	216	79.84	75.68	217				-3.29606	325.7227	0.9995
Bf	Treated wet	598.14	532.97	103	564.10	497.93	97				-0.46696	197.9883	0.6795
Bs	Treated dry	617.32	284.14	108	399.47	221.08	116				-6.37196	201.9238	1.0000
Bs	Treated wet	1387.87	735.62	78	1392.48	717.84	75				0.039224	150.966	0.4844

Supplementary Table 2. For each condition. Mean, standard deviation (Sd), and quantity (*n*) of analysed tooth areas (by EDX and nanoindentation) or quantity of broken teeth (by breaking stress experiments) for the following parameters are given: (a) proportions, given in %, of Na, Mg, Si, P, S, Cl, K, Ca, Fe, and all elements. Differences between the individual radulae for each condition (native or treated) were tested by Kruskal-Wallis test (orange p-values = highly significant, red = significant). (b) Young's modulus (E) and hardness (H), given in GPa. Differences between the individual radulae for each condition (native or treated) were tested by Wilcoxon test (orange p-values = highly significant). (c) Breaking force, given in mN, and breaking stress, given in MPa. Differences between the conditions (native, treated, native dry, native wet, treated dry, or treated wet) were tested by t test or ANOVA (orange p-values = highly significant).

Parameter	Mean ± Sd				Wilcoxon test		
	Native		Treated		ChiSquare	df	p-value
All elements	3.65 ± 7.93		2.22 ± 6.29		259.6057	1	< 0.0001
Fe	1.96 ± 5.82		2.16 ± 6.26		0.0101	1	0.9198
K	0.02 ± 0.03		0.00 ± 0.01		3.0432	1	0.0811
Na	0.03 ± 0.25		0.00 ± 0.02		46.9454	1	< 0.0001
Mg	0.02 ± 0.14		0.00 ± 0.02		70.4860	1	< 0.0001
Si	0.00 ± 0.01		0.00 ± 0.00		13.2788	1	< 0.0003
P	0.08 ± 0.46		0.00 ± 0.02		94.4597	1	< 0.0001
S	0.02 ± 0.08		0.00 ± 0.01		168.1426	1	< 0.0001
Cl	0.00 ± 0.03		0.00 ± 0.01		19.2699	1	< 0.0001
Ca	1.53 ± 2.37		0.05 ± 0.23		938.3499	1	< 0.0001
H	1.40 ± 0.95		1.05 ± 0.80		176.583	1	<0.0001
E	11.15 ± 7.36		7.80 ± 5.9		254.9487	1	<0.0001
					T test		
	Dry		Wet		t	df	p-value
Breaking force	133.33 ± 168.70<br>*n* = 880		513.81 ± 461.61<br>*n* = 421		16.39621	474.4609	<0.0001
Breaking stress	379.27 ± 242.88<br>*n* = 456		1119.80 ± 687.50<br>*n* = 308		18.15392	359.2179	<0.0001
					ANOVA		
	Native dry	Treated dry	Native wet	Treated wet	F	df	p-value
Breaking force	167.58 ± 201.70<br>*n* = 447	97.98 ± 115.92<br>*n* = 433	452.43 ± 398.35<br>*n* = 221	581.63 ± 515.26<br>*n* = 200	170.4726	3	<0.0001
Breaking stress	258.35 ± 114.36<br>*n* = 232	504.51 ± 275.40<br>*n* = 224	852.96 ± 528.96<br>*n* = 155	1390.13 ± 724.56<br>*n* = 153	230.4705	3	<0.0001

Supplementary Table 3. For each zone (radular sac, mineralization zone, and working zone) sorted to condition (native or treated). Mean, standard deviation (Sd), and quantity (*n*) of analysed tooth areas (by EDX and nanoindentation) or quantity of broken teeth (by breaking force experiments) for the following parameters are given: (a) proportions, given in %, of Na, Mg, Si, P, S, Cl, K, Ca, Fe, and all elements. Differences between the zones for each condition were tested by Kruskal-Wallis test (orange p-values = highly significant). (b) Young's modulus (E) and hardness (H), given in GPa. Differences between the zones for each condition were tested by ANOVA (orange p-values = highly significant). (c) Breaking force (Bf), given in mN, and breaking stress (Bs), given in MPa. Differences between the zones for each condition (native dry, native wet, treated dry, or treated wet) were tested by Kruskal-Wallis test (black p-values = not significant).

Parameter	Condition	Radular sac				Mineralization zone				Working zone				Results from Kruskal-Wallis		
		Sum	Mean	Sd	*n*	Sum	Mean	Sd	*n*	Sum	Mean	Sd	*n*	ChiSquare	df	p-value
Na	Native	16.09	0.03	0.19	637	5.31	0.01	0.12	797	43.62	0.07	0.38	640	27.6204	2	< 0.0001*
Mg	Native	0.00	0.00	0.00	637	7.92	0.01	0.09	797	39.18	0.06	0.24	640	80.0754	2	< 0.0001*
Si	Native	0.00	0.00	0.00	637	0.32	0.00	0.00	797	0.84	0.00	0.01	640	10.2193	2	0.0060*
P	Native	1.34	0.00	0.04	637	67.00	0.08	0.43	797	101.85	0.16	0.67	640	41.7880	2	< 0.0001*
S	Native	4.12	0.01	0.03	637	34.63	0.04	0.12	797	1.08	0.00	0.01	640	122.3041	2	< 0.0001*
Cl	Native	0.00	0.00	0.00	637	8.18	0.01	0.05	797	0.06	0.00	0.00	640	76.9271	2	< 0.0001*
K	Native	0.10	0.00	0.00	637	0.00	0.00	0.00	797	5.00	0.01	0.06	640	34.5237	2	< 0.0001*
Ca	Native	312.58	0.49	0.96	637	1562.26	1.96	2.93	797	1294.83	2.02	2.26	640	182.6653	2	< 0.0001*
Fe	Native	176.73	0.28	1.12	637	1492.93	1.87	4.94	797	2390.78	3.74	8.50	640	66.3440	2	< 0.0001*
All elements	Native	510.96	0.80	1.79	637	3178.55	3.99	7.59	797	3877.23	6.06	10.71	640	171.8470	2	< 0.0001*
Na	Treated	0.00	0.00	0.00	638	0.00	0.00	0.00	796	0.90	0.00	0.03	634	4.5322	2	0.1037
Mg	Treated	1.04	0.00	0.04	638	0.00	0.00	0.00	796	0.20	0.00	0.01	634	1.2553	2	0.5338
Si	Treated	0.10	0.00	0.00	638	0.00	0.00	0.00	796	0.00	0.00	0.00	634	2.2445	2	0.3255
P	Treated	0.00	0.00	0.00	638	1.61	0.00	0.04	796	0.00	0.00	0.00	634	3.1895	2	0.2030
S	Treated	0.36	0.00	0.01	638	0.93	0.00	0.02	796	0.00	0.00	0.00	634	3.7718	2	0.1517
Cl	Treated	0.11	0.00	0.00	638	1.61	0.00	0.02	796	0.06	0.00	0.00	634	14.7670	2	0.0006*
K	Treated	0.10	0.00	0.00	638	0.00	0.00	0.00	796	1.25	0.00	0.02	634	14.6396	2	0.0007*
Ca	Treated	12.83	0.02	0.11	638	38.60	0.05	0.21	796	57.94	0.09	0.33	634	28.0213	2	< 0.0001*
Fe	Treated	191.86	0.30	1.21	638	1640.56	2.06	5.28	796	2649.28	4.18	9.18	634	66.5291	2	< 0.0001*
All elements	Treated	206.41	0.32	1.21	638	1683.30	2.11	5.28	796	2709.64	4.27	9.23	634	85.4997	2	< 0.0001*
														Results from ANOVA		
														F Ratio	df	p-value
H	Native		0.55	0.40	637		1.53	0.84	797		2.10	0.81	640	749.4349	2	< 0.0001*
E	Native		4.39	3.07	637		12.03	6.36	797		16.77	6.17	640	827.3774	2	< 0.0001*
H	Treated		0.44	0.28	638		1.16	0.69	796		1.53	0.89	634	444.0553	2	< 0.0001*
E	Treated		3.49	2.12	638		8.05	4.92	796		11.82	6.81	634	443.8665	2	< 0.0001*
														Results from Kruskal-Wallis		
														ChiSquare	df	p-value
Bf	Native dry		55.72	48.75	160		170.25	165.09	151		296.21	264.24	136	195.3931	2	< 0.0001*
Bf	Native wet		166.68	116.03	58		433.48	294.42	77		662.12	472.92	86	51.3350	2	< 0.0001*
Bs	Native dry		130.76	62.38	68		272.30	78.68	86		354.21	71.53	78	142.8282	2	< 0.0001*
Bs	Native wet		452.78	216.64	41		863.75	411.53	56		1125.43	607.26	58	34.5874	2	< 0.0001*
Bf	Treated dry		35.12	29.71	159		98.17	96.87	142		173.48	150.37	132	187.9531	2	< 0.0001*
Bf	Treated wet		210.08	156.47	54		568.14	374.50	68		850.62	614.00	78	36.7029	2	< 0.0001*
Bs	Treated dry		273.77	142.42	67		522.58	215.57	79		684.40	275.23	78	84.3258	2	< 0.0001*
Bs	Treated wet		591.94	264.89	41		1382.89	361.75	54		1961.11	662.58	58	89.3619	2	< 0.0001*

Supplementary Table 4. For each tooth type (Ct, central tooth; Lt I, lateral tooth I; Lt II, lateral tooth II; Mt, marginal tooth) sorted to condition (native or treated). Sum, mean, standard deviation (Sd), and quantity (*n*) of analysed tooth areas (by EDX or nanoindentation) or quantity of broken teeth (by breaking stress experiments) for the following parameters: (a) proportions, in %, of Na, Mg, Si, P, S, Cl, K, Ca, Fe, and sum of all elements. Differences between the zones for each condition were tested by Kruskal-Wallis test (orange p-values = highly significant). (b) Young's modulus (E) and hardness (H) are given in GPa. Differences between the zones for each condition were tested by ANOVA (orange p-values = significant). (c) Breaking force (Bf) is given in mN and breaking stress (Bs) in MPa. Differences between the zones for each condition (native dry, native wet, treated dry, or treated wet) were tested by ANOVA (breaking force, orange p-values = highly significant) or by t test (breaking stress, orange p-values = highly significant).

Parameter	Condition	Ct				Lt I				Lt II				Mt				Results from Kruskal-Wallis		
		Sum	Mean	Sd	*n*	Sum	Mean	Sd	*n*	Sum	Mean	Sd	*n*	Sum	Mean	Sd	*n*	ChiSquare	df	p-value
Na	Native	0.00	0.00	0.00	229	0.00	0.00	0.00	463	61.91	0.09	0.42	690	3.11	0.00	0.05	692	79.2646	3	<0.0001*
Mg	Native	0.00	0.00	0.00	229	0.00	0.00	0.00	463	47.10	0.07	0.24	690	0.00	0.00	0.00	692	155.9332	3	<0.0001*
Si	Native	0.00	0.00	0.00	229	0.17	0.00	0.01	463	0.55	0.00	0.01	690	0.44	0.00	0.01	692	3.9068	3	0.2717
P	Native	0.00	0.00	0.00	229	0.00	0.00	0.00	463	163.14	0.24	0.76	690	7.04	0.01	0.19	692	193.2730	3	<0.0001*
S	Native	5.58	0.02	0.10	229	7.22	0.02	0.06	463	11.89	0.02	0.06	690	15.14	0.02	0.09	692	5.4994	3	0.1387
Cl	Native	0.52	0.00	0.02	229	2.26	0.00	0.03	463	4.07	0.01	0.05	690	1.39	0.00	0.02	692	4.4746	3	0.2146
K	Native	1.22	0.01	0.06	229	0.00	0.00	0.00	463	3.37	0.00	0.04	690	0.51	0.00	0.01	692	15.5432	3	0.0014*
Ca	Native	113.75	0.50	1.01	229	189.76	0.41	0.97	463	2608.96	3.78	2.78	690	257.20	0.37	0.77	692	1047.4882	3	<0.0001*
Fe	Native	7.05	0.03	0.17	229	3.21	0.01	0.06	463	4043.23	5.86	8.89	690	6.96	0.01	0.09	692	1296.9599	3	<0.0001*
All elements	Native	128.12	0.56	1.02	229	202.62	0.44	0.97	463	6944.22	10.06	11.21	690	291.77	0.42	0.79	692	1059.7455	3	<0.0001*
Na	Treated	0.00	0.00	0.00	230	0.00	0.00	0.00	459	0.90	0.00	0.03	691	0.00	0.00	0.00	688	3.9932	3	0.2622
Mg	Treated	1.24	0.01	0.07	230	0.00	0.00	0.00	459	0.00	0.00	0.00	691	0.00	0.00	0.00	688	16.0077	3	0.0011*
Si	Treated	0.00	0.00	0.00	230	0.00	0.00	0.00	459	0.00	0.00	0.00	691	0.10	0.00	0.00	688	2.0000	3	0.5724
P	Treated	0.00	0.00	0.00	230	0.83	0.00	0.04	459	0.78	0.00	0.03	691	0.00	0.00	0.00	688	1.7543	3	0.6249
S	Treated	0.16	0.00	0.01	230	0.67	0.00	0.02	459	0.46	0.00	0.01	691	0.00	0.00	0.00	688	4.2058	3	0.2401
Cl	Treated	0.06	0.00	0.00	230	1.06	0.00	0.02	459	0.48	0.00	0.01	691	0.18	0.00	0.00	688	4.4635	3	0.2156
K	Treated	0.30	0.00	0.01	230	0.00	0.00	0.00	459	0.67	0.00	0.01	691	0.38	0.00	0.01	688	4.6665	3	0.1979
Ca	Treated	8.51	0.04	0.16	230	3.13	0.01	0.05	459	77.31	0.11	0.37	691	20.43	0.03	0.11	688	40.0048	3	<0.0001*
Fe	Treated	4.14	0.02	0.11	230	2.99	0.01	0.06	459	4467.46	6.47	9.49	691	7.12	0.01	0.10	688	1295.7281	3	<0.0001*
All elements	Treated	14.41	0.06	0.22	230	8.67	0.02	0.09	459	4548.06	6.58	9.50	691	28.21	0.04	0.14	688	1073.3262	3	<0.0001*
																		Results from ANOVA		
																		F ratio	df	p-value
H	Native		0.93	0.62	229		0.96	0.63	463		2.08	1.08	690		1.18	0.64	692	251.9113	3	<0.0001*
E	Native		7.79	5.25	229		7.87	5.26	463		16.17	8.32	690		9.44	5.18	692	218.3512	3	<0.0001*
H	Treated		0.69	0.47	230		0.70	0.44	459		1.62	1.02	691		0.84	0.42	688	237.2871	3	<0.0001*
E	Treated		5.91	4.05	230		6.00	3.76	459		11.30	8.14	691		6.11	2.70	688	144.0745	3	<0.0001*
																		Results from ANOVA (Bf) or t test (Bs)		
																		F ratio or t ratio	df	p-value
Bf	Native dry		105.50	77.91	64		92.00	65.65	126		385.61	258.38	128		55.30	24.18	127	134.9219	3	<0.0001*
Bf	Native wet		245.42	137.91	22		237.84	147.18	44		723.43	388.08	111		86.88	17.46	44	68.6591	3	<0.0001*
Bs	Native dry										247.26	115.58	105		267.52	112.99	127	1.345512	230	<0.0001*
Bs	Native wet										1059.60	487.74	111		331.66	72.27	44	-9.83833	153	<0.0001*
Bf	Treated dry		72.22	53.80	62		64.75	50.77	124		208.47	158.03	124		33.06	15.46	123	87.9258	3	<0.0001*
Bf	Treated wet		326.40	193.25	15		276.83	191.40	32		897.57	478.05	111		70.03	18.04	42	63.4137	3	<0.0001*
Bs	Treated dry										423.79	274.30	101		570.78	259.12	123	4.114165	222	<0.0001*
Bs	Treated wet										1551.42	766.01	111		963.86	339.81	42	-4.78827	151	<0.0001*

Supplementary Table 5. For proportions of Na, Mg, Si, P, S, Cl, K, Ca, Fe, all elements, Young's modulus (E), hardness (H), breaking force (Bf), and breaking stress (Bs): pairwise comparison between treated and native tooth types (Ct, central tooth; Lt I, lateral tooth I; Lt II, lateral tooth II; Mt, marginal tooth) was performed by Wilcoxon method (orange p-values = highly significant, red = significant).

Parameter	Structure 1	Structure 1	1-Way Test, ChiSquare approximation			Wilcoxon method
			ChiSquare	df	p-value	p-value
Na	Treated Lt II	Native Lt II	197.7465	7	<.0001*	<.0001*
Na	Treated Lt I	Native Lt I				1.0000
Na	Treated Mt	Native Mt				0.0254
Na	Treated Ct	Native Ct				1.0000
Mg	Treated Lt II	Native Lt II	369.3892	7	<.0001*	<.0001*
Mg	Treated Lt I	Native Lt I				1.0000
Mg	Treated Mt	Native Mt				1.0000
Mg	Treated Ct	Native Ct				0.1587
Si	Treated Lt II	Native Lt II	20.8149	7	0.0041*	0.0046*
Si	Treated Lt I	Native Lt I				0.1593
Si	Treated Mt	Native Mt				0.0591
Si	Treated Ct	Native Ct				1.0000
P	Treated Lt II	Native Lt II	464.6647	7	<.0001*	<.0001*
P	Treated Lt	Native Lt I				0.3163
P	Treated Mt	Native Mt				0.1581
P	Treated Ct	Native Ct				1.0000
S	Treated Lt II	Native Lt II	179.2865	7	<.0001*	<.0001*
S	Treated Lt I	Native Lt I				<.0001*
S	Treated Mt	Native Mt				<.0001*
S	Treated Ct	Native Ct				<.0001*
Cl	Treated Lt II	Native Lt II	28.0896	7	0.0002*	0.0034*
Cl	Treated Lt I	Native Lt I				0.0732
Cl	Treated Mt	Native Mt				0.0072*
Cl	Treated Ct	Native Ct				0.3126
K	Treated Lt II	Native Lt II	26.8353	7	0.0004*	0.0559
K	Treated Lt I	Native Lt I				1.0000
K	Treated Mt	Native Mt				0.9988
K	Treated Ct	Native Ct				0.6484
Ca	Treated Lt II	Native Lt II	2177.0622	7	<.0001*	<.0001*
Ca	Treated Lt I	Native Lt I				<.0001*
Ca	Treated Mt	Native Mt				<.0001*
Ca	Treated Ct	Native Ct				<.0001*
Fe	Treated Lt II	Native Lt II	2593.3205	7	<.0001*	0.4500
Fe	Treated Lt I	Native Lt I				0.9891
Fe	Treated Mt	Native Mt				0.9943
Fe	Treated Ct	Native Ct				0.6103
All elements	Treated Lt II	Native Lt II	2146.2191	7	<.0001*	<.0001*
All elements	Treated Lt I	Native Lt I				<.0001*
All elements	Treated Mt	Native Mt				<.0001*
All elements	Treated Ct	Native Ct				<.0001*
			Results from ANOVA			Tukey-Kramer
			F ratio	df	p-value	p-value
H	Treated Lt II	Native Lt II	242.8407	7	<.0001*	<.0001*
H	Treated Lt I	Native Lt I				<.0001*
H	Treated Mt	Native Mt				<.0001*
H	Treated Ct	Native Ct				0.0117*
E	Treated Lt II	Native Lt II	207.5219	7	<.0001*	<.0001*
E	Treated Lt I	Native Lt I				<.0001*
E	Treated Mt	Native Mt				<.0001*
E	Treated Ct	Native Ct				0.0170*
Bf	Native dry Lt II	Native wet Lt II	145.1643	15	<.0001*	<.0001*
Bf	Native dry Lt I	Native wet Lt I				0.0086*
Bf	Native dry Mt	Native wet Mt				1.0000
Bf	Native dry Ct	Native wet Ct				0.3516
Bf	Treated dry Lt II	Treated wet Lt II				<.0001*
Bf	Treated dry Lt I	Treated wet Lt I				<.0001*
Bf	Treated dry Mt	Treated wet Mt				0.9999
Bf	Treated dry Ct	Treated wet Ct				0.0034*
Bf	Native dry Lt II	Treated dry Lt II				<.0001*
Bf	Native dry Lt I	Treated dry Lt I				0.9998
Bf	Native dry Mt	Treated dry Mt				1.0000
Bf	Native dry Ct	Treated dry Ct				1.0000
Bf	Native wet Lt II	Treated wet Lt II				<.0001*
Bf	Native wet Lt I	Treated wet Lt I				1.0000
Bf	Native wet Mt	Treated wet Mt				1.0000
Bf	Native wet Ct	Treated wet Ct				0.9991
Bs	Native dry Lt II	Native wet Lt II	149.5308	7	<.0001*	<.0001*
Bs	Native dry Mt	Native wet Mt				0.9817
Bs	Treated dry Lt II	Treated wet Lt II				<.0001*
Bs	Treated dry Mt	Treated wet Mt				<.0001*
Bs	Native dry Lt II	Treated dry Lt II				<.0001*
Bs	Native dry Mt	Treated dry Mt				<.0001*
Bs	Native wet Lt II	Treated wet Lt II				<.0001*
Bs	Native wet Mt	Treated wet Mt				<.0001*

Supplementary Table 6. For each tooth type sorted to condition (native or treated) and radular zone (Mz, mineralization zone; Rs, radular sac; Wz, working zone). Mean (M), standard deviation (Sd), and quantity of analysed tooth areas (*n*) are provided for the following parameters (P): proportions, given in %, of Na, Mg, Si, P, S, Cl, K, Ca, Fe, and all elements (Ae) Young's modulus (E) and hardness (H), given in GPa. U, unit of measure.

Condition	Zone	Parameter	U	Central tooth			Lateral tooth I			Lateral tooth II			Marginal tooth		
				M	Sd	*n*	M	Sd	*n*	M	Sd	*n*	M	Sd	*n*
Native	Rs	H	GPa	0.31	0.02	69	0.34	0.03	143	0.85	0.54	213	0.49	0.21	211
Native	Rs	E	GPa	2.62	0.17	69	2.76	0.28	143	6.62	4.16	213	3.83	1.65	211
Native	Rs	Fe	%	0.01	0.09	69	0.02	0.11	143	0.78	1.82	213	0.03	0.16	211
Native	Rs	Ca	%	0.10	0.28	69	0.03	0.20	143	1.23	1.27	213	0.18	0.48	211
Native	Rs	Na	%	0.00	0.00	69	0.00	0.00	143	0.06	0.32	213	0.01	0.10	211
Native	Rs	Mg	%	0.00	0.00	69	0.00	0.00	143	0.00	0.00	213	0.00	0.00	211
Native	Rs	Si	%	0.00	0.00	69	0.00	0.00	143	0.00	0.00	213	0.00	0.00	211
Native	Rs	P	%	0.00	0.00	69	0.00	0.00	143	0.01	0.07	213	0.00	0.00	211
Native	Rs	S	%	0.00	0.00	69	0.01	0.04	143	0.01	0.03	213	0.01	0.04	211
Native	Rs	Cl	%	0.00	0.00	69	0.00	0.00	143	0.00	0.00	213	0.00	0.00	211
Native	Rs	K	%	0.00	0.01	69	0.00	0.00	143	0.00	0.00	213	0.00	0.00	211
Native	Rs	Ae	%	0.11	0.28	69	0.06	0.23	143	2.09	2.60	213	0.23	0.50	211
Native	Mz	H	GPa	0.80	0.38	90	0.82	0.39	178	2.41	0.64	263	1.37	0.49	265
Native	Mz	E	GPa	6.64	3.13	90	6.75	3.23	178	18.64	4.83	263	10.86	3.84	265
Native	Mz	Fe	%	0.03	0.16	90	0.00	0.01	178	5.66	7.25	263	0.00	0.02	265
Native	Mz	Ca	%	0.52	1.05	90	0.39	0.88	178	5.20	3.03	263	0.30	0.57	265
Native	Mz	Na	%	0.00	0.00	90	0.00	0.00	178	0.02	0.20	263	0.00	0.00	265
Native	Mz	Mg	%	0.00	0.00	90	0.00	0.00	178	0.03	0.15	263	0.00	0.00	265
Native	Mz	Si	%	0.00	0.00	90	0.00	0.00	178	0.00	0.00	263	0.00	0.01	265
Native	Mz	P	%	0.00	0.00	90	0.00	0.00	178	0.24	0.70	263	0.01	0.17	265
Native	Mz	S	%	0.06	0.15	90	0.03	0.09	178	0.04	0.09	263	0.05	0.14	265
Native	Mz	Cl	%	0.01	0.03	90	0.01	0.05	178	0.02	0.08	263	0.01	0.02	265
Native	Mz	K	%	0.00	0.00	90	0.00	0.00	178	0.00	0.00	263	0.00	0.00	265
Native	Mz	Ae	%	0.62	1.03	90	0.44	0.87	178	11.21	9.79	263	0.37	0.60	265
Native	Wz	H	GPa	1.71	0.28	70	1.75	0.29	142	2.92	0.79	214	1.64	0.51	210
Native	Wz	E	GPa	14.37	2.46	70	14.41	2.52	142	22.65	6.11	214	13.24	4.21	210
Native	Wz	Fe	%	0.05	0.24	70	0.03	0.03	142	11.15	11.57	214	0.00	0.04	210
Native	Wz	Ca	%	0.87	1.26	70	0.82	1.34	142	4.58	1.61	214	0.65	1.09	210
Native	Wz	Na	%	0.00	0.00	70	0.00	0.00	142	0.20	0.64	214	0.00	0.00	210
Native	Wz	Mg	%	0.00	0.00	70	0.00	0.00	142	0.18	0.38	214	0.00	0.00	210
Native	Wz	Si	%	0.00	0.00	70	0.00	0.01	142	0.00	0.01	214	0.00	0.01	210
Native	Wz	P	%	0.00	0.00	70	0.00	0.00	142	0.46	1.07	214	0.02	0.29	210
Native	Wz	S	%	0.00	0.00	70	0.00	0.00	142	0.01	0.02	214	0.00	0.00	210
Native	Wz	Cl	%	0.00	0.01	70	0.00	0.00	142	0.00	0.00	214	0.00	0.00	210
Native	Wz	K	%	0.02	0.11	70	0.00	0.00	142	0.02	0.07	214	0.00	0.02	210
Native	Wz	Ae	%	0.93	1.29	70	0.86	1.35	142	16.60	13.18	214	0.67	1.12	210
Treated	Rs	H	GPa	0.23	0.02	71	0.29	0.04	140	0.62	0.37	212	0.42	0.16	209
Treated	Rs	E	GPa	1.95	0.15	71	2.38	0.27	140	4.86	2.94	212	3.35	1.10	209
Treated	Rs	Fe	%	0.01	0.07	71	0.02	0.10	140	0.85	1.97	212	0.03	0.16	209
Treated	Rs	Ca	%	0.02	0.07	71	0.00	0.00	140	0.05	0.18	212	0.00	0.04	209
Treated	Rs	Na	%	0.00	0.00	71	0.00	0.00	140	0.00	0.00	212	0.00	0.00	209
Treated	Rs	Mg	%	0.01	0.12	71	0.00	0.00	140	0.00	0.00	212	0.00	0.00	209
Treated	Rs	Si	%	0.00	0.00	71	0.00	0.00	140	0.00	0.00	212	0.00	0.01	209
Treated	Rs	P	%	0.00	0.00	71	0.00	0.00	140	0.00	0.00	212	0.00	0.00	209
Treated	Rs	S	%	0.00	0.00	71	0.00	0.00	140	0.00	0.02	212	0.00	0.00	209
Treated	Rs	Cl	%	0.00	0.00	71	0.00	0.01	140	0.00	0.00	212	0.00	0.00	209
Treated	Rs	K	%	0.00	0.01	71	0.00	0.00	140	0.00	0.00	212	0.00	0.00	209
Treated	Rs	Ae	%	0.04	0.15	71	0.02	0.10	140	0.90	1.96	212	0.03	0.17	209
Treated	Mz	H	GPa	0.61	0.31	89	0.62	0.29	176	1.87	0.61	264	1.01	0.38	267

Treated	Mz	E	GPa	5.29	2.70	89	5.21	2.38	176	12.03	6.05	264	6.94	2.27	267
Treated	Mz	Fe	%	0.01	0.12	89	0.00	0.01	176	6.21	7.66	264	0.00	0.02	267
Treated	Mz	Ca	%	0.03	0.20	89	0.02	0.08	176	0.09	0.32	264	0.04	0.11	267
Treated	Mz	Na	%	0.00	0.00	89	0.00	0.00	176	0.00	0.00	264	0.00	0.00	267
Treated	Mz	Mg	%	0.00	0.00	89	0.00	0.00	176	0.00	0.00	264	0.00	0.00	267
Treated	Mz	Si	%	0.00	0.00	89	0.00	0.00	176	0.00	0.00	264	0.00	0.00	267
Treated	Mz	P	%	0.00	0.00	89	0.00	0.06	176	0.00	0.05	264	0.00	0.00	267
Treated	Mz	S	%	0.00	0.02	89	0.00	0.03	176	0.00	0.01	264	0.00	0.00	267
Treated	Mz	Cl	%	0.00	0.00	89	0.01	0.04	176	0.00	0.01	264	0.00	0.01	267
Treated	Mz	K	%	0.00	0.00	89	0.00	0.00	176	0.00	0.00	264	0.00	0.00	267
Treated	Mz	Ae	%	0.05	0.23	89	0.03	0.12	176	6.30	7.63	264	0.04	0.04	267
Treated	Wz	H	GPa	1.24	0.26	65	1.22	0.28	145	2.31	1.12	223	1.05	0.33	214
Treated	Wz	E	GPa	10.73	2.18	65	10.59	1.97	145	16.88	9.36	223	7.84	2.14	214
Treated	Wz	Fe	%	0.00	0.00	65	0.03	0.14	145	12.42	12.19	223	0.00	0.04	214
Treated	Wz	Ca	%	0.07	0.17	65	0.00	0.00	145	0.20	0.52	223	0.05	0.15	214
Treated	Wz	Na	%	0.00	0.00	65	0.00	0.00	145	0.00	0.06	223	0.00	0.00	214
Treated	Wz	Mg	%	0.00	0.02	65	0.00	0.00	145	0.00	0.00	223	0.00	0.00	214
Treated	Wz	Si	%	0.00	0.00	65	0.00	0.00	145	0.00	0.00	223	0.00	0.00	214
Treated	Wz	P	%	0.00	0.00	65	0.00	0.00	145	0.00	0.00	223	0.00	0.00	214
Treated	Wz	S	%	0.00	0.00	65	0.00	0.00	145	0.00	0.00	223	0.00	0.00	214
Treated	Wz	Cl	%	0.00	0.01	65	0.00	0.00	145	0.00	0.00	223	0.00	0.00	214
Treated	Wz	K	%	0.00	0.02	65	0.00	0.00	145	0.00	0.02	223	0.00	0.02	214
Treated	Wz	Ae	%	0.08	0.18	65	0.09	0.25	145	12.63	12.18	223	0.05	0.16	214

Supplementary Table 7. For each tooth type sorted to condition (native dry, native wet, treated dry, treated wet) and radular zone (Mz, mineralization zone; Rs, radular sac; Wz, working zone). Mean (M), standard deviation (Sd), and quantity (*n*) of broken teeth for breaking force (Bf) and calculated breaking stress (Bs) are provided. D, dry; W, wet.

Condition	Zone	D or W	Central tooth Bf, mN			Lateral tooth I Bf, mN			Lateral tooth II Bf, mN			Lateral tooth II Bs, MPa			Marginal tooth Bf, mN			Marginal tooth Bs, MPa		
			M	Sd	*n*	M	Sd	*n*	M	Sd	*n*	M	Sd	*n*	M	Sd	*n*	M	Sd	*n*
Native	Rs	D	40.10	17.14	24	37.23	12.30	45	104.27	66.21	47	81.84	46.84	24	31.30	10.28	44	157.44	53.16	44
Native	Mz	D	91.28	38.85	21	84.23	28.52	43	414.82	111.87	42	244.91	66.65	42	60.51	16.17	43	298.45	81.05	43
Native	Wz	D	203.82	55.23	19	163.97	66.11	39	693.19	91.48	39	351.59	44.47	39	76.49	19.08	40	356.82	91.51	40
Native	Rs	W	81.14	12.67	6	90.60	13.97	10	245.56	113.77	30	503.37	232.16	30	74.30	11.03	11	314.81	53.11	11
Native	Mz	W	213.21	58.54	7	186.94	58.57	14	668.25	179.62	42	1046.01	299.33	42	85.87	14.63	14	316.96	68.12	14
Native	Wz	W	379.98	75.75	9	360.60	134.13	19	1150.45	143.07	39	1502.10	324.68	39	94.91	18.44	19	352.25	82.29	19
Treated	Rs	D	27.27	11.65	24	25.32	8.36	45	63.75	40.36	47	158.80	86.45	24	18.47	6.97	43	337.94	126.54	43
Treated	Mz	D	62.81	27.00	19	57.28	19.39	44	232.46	95.62	38	447.08	227.67	38	33.99	9.92	41	592.55	179.56	41
Treated	Wz	D	138.42	38.63	19	124.83	53.98	35	359.50	141.69	37	564.18	279.08	37	48.19	11.54	38	804.63	214.36	38
Treated	Rs	W	100.81	17.39	4	99.29	27.93	9	312.99	139.11	30	613.95	272.97	30	59.81	22.46	11	531.91	243.25	11
Treated	Mz	W	318.89	18.33	3	213.91	82.18	11	820.56	227.67	41	1440.57	379.79	41	71.70	16.34	12	1181.00	189.00	12
Treated	Wz	W	442.02	173.44	8	467.66	164.50	12	1430.19	152.83	39	2391.93	231.55	39	74.90	14.38	19	1076.80	222.24	19

Supplementary Table 8. For the following parameters: proportions of Na, Mg, Si, P, S, Cl, K, Ca, Fe, all elements, Young's modulus (E), hardness (H), breaking force (Bf), and breaking stress (Bs). Pairwise comparison between treated and native tooth types (Ct, central tooth; Lt I, lateral tooth I; Lt II, lateral tooth II; Mt, marginal tooth) was performed by Wilcoxon method (orange p-values = highly significant, red = significant).

Parameter	Zone	Structure 1	Structure 1	1-Way Test, ChiSquare approximation			Wilcoxon method
				ChiSquare	df	p-value	p-value
Na	Rs	Treated Lt II	Native Lt II	419.6903	7	<.0001*	0.0004*
Na	Rs	Treated Lt I	Native Lt I				1.0000
Na	Rs	Treated Mt	Native Mt				0.0245*
Na	Rs	Treated Ct	Native Ct				1.0000
Na	Mz	Treated Lt II	Native Lt II	20.2967	7	0.0050*	0.0447*
Na	Mz	Treated Lt I	Native Lt I				1.0000
Na	Mz	Treated Mt	Native Mt				1.0000
Na	Mz	Treated Ct	Native Ct				1.0000
Na	Wz	Treated Lt II	Native Lt II	145.0505	7	<.0001*	<.0001*
Na	Wz	Treated Lt I	Native Lt I				1.0000
Na	Wz	Treated Mt	Native Mt				1.0000
Na	Wz	Treated Ct	Native Ct				1.0000
Mg	Rs	Treated Lt II	Native Lt II	16.9577	7	0.0177*	1.0000
Mg	Rs	Treated Lt I	Native Lt I				1.0000
Mg	Rs	Treated Mt	Native Mt				1.0000
Mg	Rs	Treated Ct	Native Ct				0.3313
Mg	Mz	Treated Lt II	Native Lt II	92.1423	7	<.0001*	<.0001*
Mg	Mz	Treated Lt I	Native Lt I				1.0000
Mg	Mz	Treated Mt	Native Mt				1.0000
Mg	Mz	Treated Ct	Native Ct				1.0000
Mg	Wz	Treated Lt II	Native Lt II	288.8741	7	<.0001*	<.0001*
Mg	Wz	Treated Lt I	Native Lt I				1.0000
Mg	Wz	Treated Mt	Native Mt				1.0000
Mg	Wz	Treated Ct	Native Ct				0.3243
Si	Rs	Treated Lt II	Native Lt II	4.9859	7	0.6617	1.0000
Si	Rs	Treated Lt I	Native Lt I				1.0000
Si	Rs	Treated Mt	Native Mt				0.3207
Si	Rs	Treated Ct	Native Ct				1.0000
Si	Mz	Treated Lt II	Native Lt II	14.0921	7	0.0496*	0.1569
Si	Mz	Treated Lt I	Native Lt I				1.0000
Si	Mz	Treated Mt	Native Mt				0.0447*
Si	Mz	Treated Ct	Native Ct				1.0000
Si	Wz	Treated Lt II	Native Lt II	17.5084	7	0.0144*	0.0140*
Si	Wz	Treated Lt I	Native Lt I				0.1595
Si	Wz	Treated Mt	Native Mt				0.1617
Si	Wz	Treated Ct	Native Ct				1.0000
P	Rs	Treated Lt II	Native Lt II	9.9797	7	0.1897	0.1568
P	Rs	Treated Lt	Native Lt I				1.0000
P	Rs	Treated Mt	Native Mt				1.0000
P	Rs	Treated Ct	Native Ct				1.0000
P	Mz	Treated Lt II	Native Lt II	225.0170	7	<.0001*	<.0001*
P	Mz	Treated Lt	Native Lt I				0.3200
P	Mz	Treated Mt	Native Mt				0.3182
P	Mz	Treated Ct	Native Ct				1.0000
P	Wz	Treated Lt II	Native Lt II	234.2701	7	<.0001*	<.0001*
P	Wz	Treated Lt	Native Lt I				1.0000
P	Wz	Treated Mt	Native Mt				0.3242
P	Wz	Treated Ct	Native Ct				1.0000
S	Rs	Treated Lt II	Native Lt II	26.8493	7	0.0004*	0.1210
S	Rs	Treated Lt I	Native Lt I				0.0082*
S	Rs	Treated Mt	Native Mt				0.0008*
S	Rs	Treated Ct	Native Ct				1.0000
S	Mz	Treated Lt II	Native Lt II	140.8061	7	<.0001*	<.0001*
S	Mz	Treated Lt I	Native Lt I				<.0001*
S	Mz	Treated Mt	Native Mt				<.0001*
S	Mz	Treated Ct	Native Ct				<.0001*
S	Wz	Treated Lt II	Native Lt II	95.4550	7	<.0001*	<.0001*
S	Wz	Treated Lt I	Native Lt I				1.0000
S	Wz	Treated Mt	Native Mt				1.0000
S	Wz	Treated Ct	Native Ct				1.0000
Cl	Rs	Treated Lt II	Native Lt II	8.1071	7	0.3232	1.0000
Cl	Rs	Treated Lt I	Native Lt I				0.3156
Cl	Rs	Treated Mt	Native Mt				1.0000
Cl	Rs	Treated Ct	Native Ct				1.0000
Cl	Mz	Treated Lt II	Native Lt II	33.6396	7	<.0001*	0.0027*
Cl	Mz	Treated Lt I	Native Lt I				0.0338*
Cl	Mz	Treated Mt	Native Mt				0.0064*
Cl	Mz	Treated Ct	Native Ct				0.1608
Cl	Wz	Treated Lt II	Native Lt II	16.2127	7	0.0232*	1.0000
Cl	Wz	Treated Lt I	Native Lt I				1.0000
Cl	Wz	Treated Mt	Native Mt				1.0000
Cl	Wz	Treated Ct	Native Ct				1.0000

K	Rs	Treated Lt II	Native Lt II	16.2307	7	0.0231*	1.0000
K	Rs	Treated Lt I	Native Lt I				1.0000
K	Rs	Treated Mt	Native Mt				1.0000
K	Rs	Treated Ct	Native Ct				0.9919
K	Mz	Treated Lt II	Native Lt II	0.0000	7	1.0000	1.0000
K	Mz	Treated Lt I	Native Lt I				1.0000
K	Mz	Treated Mt	Native Mt				1.0000
K	Mz	Treated Ct	Native Ct				1.0000
K	Wz	Treated Lt II	Native Lt II	27.4261	7	0.0003*	0.0511
K	Wz	Treated Lt I	Native Lt I				1.0000
K	Wz	Treated Mt	Native Mt				0.9887
K	Wz	Treated Ct	Native Ct				0.5609
Ca	Rs	Treated Lt II	Native Lt II	593.3227	7	<.0001*	<.0001*
Ca	Rs	Treated Lt I	Native Lt I				0.0470*
Ca	Rs	Treated Mt	Native Mt				<.0001*
Ca	Rs	Treated Ct	Native Ct				0.1224
Ca	Mz	Treated Lt II	Native Lt II	1004.2683	7	<.0001*	<.0001*
Ca	Mz	Treated Lt I	Native Lt I				<.0001*
Ca	Mz	Treated Mt	Native Mt				<.0001*
Ca	Mz	Treated Ct	Native Ct				<.0001*
Ca	Wz	Treated Lt II	Native Lt II	754.4791	7	<.0001*	<.0001*
Ca	Wz	Treated Lt I	Native Lt I				<.0001*
Ca	Wz	Treated Mt	Native Mt				<.0001*
Ca	Wz	Treated Ct	Native Ct				<.0001*
Fe	Rs	Treated Lt II	Native Lt II	325.5845	7	<.0001*	0.8961
Fe	Rs	Treated Lt I	Native Lt I				0.9801
Fe	Rs	Treated Mt	Native Mt				0.9978
Fe	Rs	Treated Ct	Native Ct				0.9712
Fe	Mz	Treated Lt II	Native Lt II	1236.4760	7	<.0001*	0.7292
Fe	Mz	Treated Lt I	Native Lt I				1.0000
Fe	Mz	Treated Mt	Native Mt				0.9985
Fe	Mz	Treated Ct	Native Ct				0.2586
Fe	Wz	Treated Lt II	Native Lt II	1112.1097	7	<.0001*	0.0703
Fe	Wz	Treated Lt I	Native Lt I				1.0000
Fe	Wz	Treated Mt	Native Mt				0.9868
Fe	Wz	Treated Ct	Native Ct				0.7406
All elements	Rs	Treated Lt II	Native Lt II	481.9565	7	<.0001*	<.0001*
All elements	Rs	Treated Lt I	Native Lt I				0.0813
All elements	Rs	Treated Mt	Native Mt				<.0001*
All elements	Rs	Treated Ct	Native Ct				0.2509
All elements	Mz	Treated Lt II	Native Lt II	1012.9077	7	<.0001*	<.0001*
All elements	Mz	Treated Lt I	Native Lt I				<.0001*
All elements	Mz	Treated Mt	Native Mt				<.0001*
All elements	Mz	Treated Ct	Native Ct				<.0001*
All elements	Wz	Treated Lt II	Native Lt II	859.6170	7	<.0001*	<.0001*
All elements	Wz	Treated Lt I	Native Lt I				<.0001*
All elements	Wz	Treated Mt	Native Mt				<.0001*
All elements	Wz	Treated Ct	Native Ct				<.0001*
				Results from ANOVA			Tukey-Kramer
				F ratio	df	p-value	p-value
H	Rs	Treated Lt II	Native Lt II	81.7399	7	<.0001*	<.0001*
H	Rs	Treated Lt I	Native Lt I				0.8501
H	Rs	Treated Mt	Native Mt				<.0001*
H	Rs	Treated Ct	Native Ct				0.6828
E	Rs	Treated Lt II	Native Lt II	77.7588	7	<.0001*	<.0001*
E	Rs	Treated Lt I	Native Lt I				0.8421
E	Rs	Treated Mt	Native Mt				<.0001*
E	Rs	Treated Ct	Native Ct				0.6470
H	Mz	Treated Lt II	Native Lt II	376.0661	7	<.0001*	<.0001*
H	Mz	Treated Lt I	Native Lt I				0.0019*
H	Mz	Treated Mt	Native Mt				<.0001*
H	Mz	Treated Ct	Native Ct				0.1909
E	Mz	Treated Lt II	Native Lt II	289.1062	7	<.0001*	<.0001*
E	Mz	Treated Lt I	Native Lt I				0.0066*
E	Mz	Treated Mt	Native Mt				<.0001*
E	Mz	Treated Ct	Native Ct				0.3164
H	Wz	Treated Lt II	Native Lt II	184.4595	7	<.0001*	<.0001*
H	Wz	Treated Lt I	Native Lt I				<.0001*
H	Wz	Treated Mt	Native Mt				<.0001*
H	Wz	Treated Ct	Native Ct				0.0003*
E	Wz	Treated Lt II	Native Lt II	151.8233	7	<.0001*	<.0001*
E	Wz	Treated Lt I	Native Lt I				<.0001*
E	Wz	Treated Mt	Native Mt				<.0001*
E	Wz	Treated Ct	Native Ct				0.0008*
				1-Way Test, ChiSquare approximation			Wilcoxon method
				ChiSquare	df	p-value	p-value
Bf	Rs	Native dry Lt II	Native wet Lt II	318.5840	15	<.0001*	<.0001*
Bf	Rs	Native dry Lt I	Native wet Lt I				<.0001*
Bf	Rs	Native dry Mt	Native wet Mt				<.0001*
Bf	Rs	Native dry Ct	Native wet Ct				0.0010*
Bf	Rs	Treated dry Lt II	Treated wet Lt II				<.0001*
Bf	Rs	Treated dry Lt I	Treated wet Lt I				<.0001*

Bf	Rs	Treated dry Mt	Treated wet Mt				<.0001*
Bf	Rs	Treated dry Ct	Treated wet Ct				0.0018*
Bf	Rs	Native dry Lt II	Treated dry Lt II				0.0015*
Bf	Rs	Native dry Lt I	Treated dry Lt I				<.0001*
Bf	Rs	Native dry Mt	Treated dry Mt				<.0001*
Bf	Rs	Native dry Ct	Treated dry Ct				0.0006*
Bf	Rs	Native wet Lt II	Treated wet Lt II				0.0451*
Bf	Rs	Native wet Lt I	Treated wet Lt I				0.2231
Bf	Rs	Native wet Mt	Treated wet Mt				0.0657
Bf	Rs	Native wet Ct	Treated wet Ct				0.1658
Bf	Mz	Native dry Lt II	Native wet Lt II				<.0001*
Bf	Mz	Native dry Lt I	Native wet Lt I				<.0001*
Bf	Mz	Native dry Mt	Native wet Mt				<.0001*
Bf	Mz	Native dry Ct	Native wet Ct				0.0003*
Bf	Mz	Treated dry Lt II	Treated wet Lt II				<.0001*
Bf	Mz	Treated dry Lt I	Treated wet Lt I				<.0001*
Bf	Mz	Treated dry Mt	Treated wet Mt				<.0001*
Bf	Mz	Treated dry Ct	Treated wet Ct	386.1311	15	<.0001*	0.0074*
Bf	Mz	Native dry Lt II	Treated dry Lt II				<.0001*
Bf	Mz	Native dry Lt I	Treated dry Lt I				<.0001*
Bf	Mz	Native dry Mt	Treated dry Mt				<.0001*
Bf	Mz	Native dry Ct	Treated dry Ct				0.0101*
Bf	Mz	Native wet Lt II	Treated wet Lt II				0.0021*
Bf	Mz	Native wet Lt I	Treated wet Lt I				0.4597
Bf	Mz	Native wet Mt	Treated wet Mt				0.0366*
Bf	Mz	Native wet Ct	Treated wet Ct				0.0227*
Bf	Wz	Native dry Lt II	Native wet Lt II				<.0001*
Bf	Wz	Native dry Lt I	Native wet Lt I				<.0001*
Bf	Wz	Native dry Mt	Native wet Mt				0.0013*
Bf	Wz	Native dry Ct	Native wet Ct				<.0001*
Bf	Wz	Treated dry Lt II	Treated wet Lt II				<.0001*
Bf	Wz	Treated dry Lt I	Treated wet Lt I				<.0001*
Bf	Wz	Treated dry Mt	Treated wet Mt				<.0001*
Bf	Wz	Treated dry Ct	Treated wet Ct	398.6515	15	<.0001*	<.0001*
Bf	Wz	Native dry Lt II	Treated dry Lt II				<.0001*
Bf	Wz	Native dry Lt I	Treated dry Lt I				0.0097*
Bf	Wz	Native dry Mt	Treated dry Mt				<.0001*
Bf	Wz	Native dry Ct	Treated dry Ct				0.0004*
Bf	Wz	Native wet Lt II	Treated wet Lt II				<.0001*
Bf	Wz	Native wet Lt I	Treated wet Lt I				0.1092
Bf	Wz	Native wet Mt	Treated wet Mt				0.0015*
Bf	Wz	Native wet Ct	Treated wet Ct				0.5964
Bs	Rs	Native dry Lt II	Native wet Lt II				<.0001*
Bs	Rs	Native dry Mt	Native wet Mt				<.0001*
Bs	Rs	Treated dry Lt II	Treated wet Lt II				<.0001*
Bs	Rs	Treated dry Mt	Treated wet Mt	154.3249	7	<.0001*	0.0127*
Bs	Rs	Native dry Lt II	Treated dry Lt II				0.0004*
Bs	Rs	Native dry Mt	Treated dry Mt				<.0001*
Bs	Rs	Native wet Lt II	Treated wet Lt II				0.0962
Bs	Rs	Native wet Mt	Treated wet Mt				0.0047*
Bs	Mz	Native dry Lt II	Native wet Lt II				<.0001*
Bs	Mz	Native dry Mt	Native wet Mt				0.4187
Bs	Mz	Treated dry Lt II	Treated wet Lt II				<.0001*
Bs	Mz	Treated dry Mt	Treated wet Mt	212.2759	7	<.0001*	<.0001*
Bs	Mz	Native dry Lt II	Treated dry Lt II				0.0001*
Bs	Mz	Native dry Mt	Treated dry Mt				<.0001*
Bs	Mz	Native wet Lt II	Treated wet Lt II				<.0001*
Bs	Mz	Native wet Mt	Treated wet Mt				<.0001*
Bs	Wz	Native dry Lt II	Native wet Lt II				<.0001*
Bs	Wz	Native dry Mt	Native wet Mt				0.7909
Bs	Wz	Treated dry Lt II	Treated wet Lt II				<.0001*
Bs	Wz	Treated dry Mt	Treated wet Mt	223.2620	7	<.0001*	<.0001*
Bs	Wz	Native dry Lt II	Treated dry Lt II				0.0099*
Bs	Wz	Native dry Mt	Treated dry Mt				<.0001*
Bs	Wz	Native wet Lt II	Treated wet Lt II				<.0001*
Bs	Wz	Native wet Mt	Treated wet Mt				<.0001*

Supplementary Table 9. Percentage of different failure types (sorted to the area, where failure occurred) of each tooth type during breaking stress experiments. Sorted to radular zones, native and treated, tested under dry or wet condition. Quantity of experiments resulting in this specific failure type (*n*) and the percentage of this particular type are given.

Condition	Tooth type	Dry or wet	Zone		Failure type or area of failure						
					No failing, bending	Failure close to membrane	Failure, crush	Failure at junction zone	No failure	Failure at stylus	Failure underneath junction zone
Native	Marginal	Wet	Working zone	*n*	21	11	0	0	0	8	0
				%	52.50	27.50	0.00	0.00	0.00	20.00	0.00
Native	Marginal	Wet	Mineralization zone	*n*	28	6	0	0	2	8	0
				%	63.64	13.64	0.00	0.00	4.55	18.18	0.00
Native	Marginal	Wet	Radular sac	*n*	37	7	0	0	0	4	0
				%	77.08	14.58	0.00	0.00	0.00	8.33	0.00
Native	Marginal	Dry	Working zone	*n*	0	39	0	0	0	0	0
				%	0.00	100.00	0.00	0.00	0.00	0.00	0.00
Native	Marginal	Dry	Mineralization zone	*n*	0	44	0	0	0	0	0
				%	0.00	100.00	0.00	0.00	0.00	0.00	0.00
Native	Marginal	Dry	Radular sac	*n*	0	42	0	0	0	0	0
				%	0.00	100.00	0.00	0.00	0.00	0.00	0.00
Native	Lateral II	Wet	Working zone	*n*	0	0	0	0	0	0	39
				%	0.00	0.00	0.00	0.00	0.00	0.00	100.00
Native	Lateral II	Wet	Mineralization zone	*n*	0	0	0	0	0	0	42
				%	0.00	0.00	0.00	0.00	0.00	0.00	100.00
Native	Lateral II	Wet	Radular sac	*n*	17	0	0	0	0	0	30
				%	36.17	0.00	0.00	0.00	0.00	0.00	63.83
Native	Lateral II	Dry	Working zone	*n*	0	0	0	39	0	0	0
				%	0.00	0.00	0.00	100.00	0.00	0.00	0.00
Native	Lateral II	Dry	Mineralization zone	*n*	0	0	0	42	0	0	0
				%	0.00	0.00	0.00	100.00	0.00	0.00	0.00
Native	Lateral II	Dry	Radular sac	*n*	0	0	23	24	0	0	0
				%	0.00	0.00	48.94	51.06	0.00	0.00	0.00
Native	Lateral I	Wet	Working zone	*n*	21	11	0	0	0	8	0
				%	52.50	27.50	0.00	0.00	0.00	20.00	0.00
Native	Lateral I	Wet	Mineralization zone	*n*	28	6	0	0	2	8	0
				%	63.64	13.64	0.00	0.00	4.55	18.18	0.00
Native	Lateral I	Wet	Radular sac	*n*	37	7	0	0	0	4	0
				%	77.08	14.58	0.00	0.00	0.00	8.33	0.00
Native	Lateral I	Dry	Working zone	*n*	0	0	39	0	0	0	0
				%	0.00	0.00	100.00	0.00	0.00	0.00	0.00
Native	Lateral I	Dry	Mineralization zone	*n*	0	0	44	0	0	0	0
				%	0.00	0.00	100.00	0.00	0.00	0.00	0.00
Native	Lateral I	Dry	Radular sac	*n*	0	0	45	0	0	0	0
				%	0.00	0.00	100.00	0.00	0.00	0.00	0.00
Native	Central	Wet	Working zone	*n*	11	5	0	0	0	4	0
				%	55.00	25.00	0.00	0.00	0.00	20.00	0.00
Native	Central	Wet	Mineralization zone	*n*	14	3	0	0	1	4	0
				%	63.64	13.64	0.00	0.00	4.55	18.18	0.00
Native	Central	Wet	Radular sac	*n*	18	4	0	0	2	0	0
				%	75.00	16.67	0.00	0.00	8.33	0.00	0.00
Native	Central	Dry	Working zone	*n*	0	0	19	0	0	0	0
				%	0.00	0.00	100.00	0.00	0.00	0.00	0.00
Native	Central	Dry	Mineralization zone	*n*	0	0	21	0	0	0	0
				%	0.00	0.00	100.00	0.00	0.00	0.00	0.00
Native	Central	Dry	Radular sac	*n*	0	0	24	0	0	0	0
				%	0.00	0.00	100.00	0.00	0.00	0.00	0.00
Treated	Marginal	Wet	Working zone	*n*	20	11	0	0	0	8	0
				%	51.28	28.00	0.00	0.00	0.00	20.72	0.00
Treated	Marginal	Wet	Mineralization zone	*n*	26	6	0	0	2	6	0
				%	65.00	15.00	0.00	0.00	5.00	15.00	0.00
Treated	Marginal	Wet	Radular sac	*n*	39	7	0	0	0	4	0
				%	78.00	14.00	0.00	0.00	0.00	8.00	0.00
Treated	Marginal	Dry	Working zone	*n*	0	39	0	0	0	0	0
				%	0.00	100.00	0.00	0.00	0.00	0.00	0.00
Treated	Marginal	Dry	Mineralization zone	*n*	0	41	0	0	0	0	0
				%	0.00	100.00	0.00	0.00	0.00	0.00	0.00
Treated	Marginal	Dry	Radular sac	*n*	0	43	0	0	0	0	0
				%	0.00	100.00	0.00	0.00	0.00	0.00	0.00
Treated	Lateral II	Wet	Working zone	*n*	0	0	0	0	0	0	40
				%	0.00	0.00	0.00	0.00	0.00	0.00	100.00

Treated	Lateral II	Wet	Mineralization zone	*n*<br>%	0<br>0.00	0<br>0.00	0<br>0.00	0<br>0.00	0<br>0.00	0<br>0.00	41<br>100.00
Treated	Lateral II	Wet	Radular sac	*n*<br>%	17<br>36.17	0<br>0.00	0<br>0.00	0<br>0.00	0<br>0.00	0<br>0.00	30<br>63.83
Treated	Lateral II	Dry	Working zone	*n*<br>%	0<br>0.00	0<br>0.00	0<br>0.00	39<br>100.00	0<br>0.00	0<br>0.00	0<br>0.00
Treated	Lateral II	Dry	Mineralization zone	*n*<br>%	0<br>0.00	0<br>0.00	0<br>0.00	38<br>100.00	0<br>0.00	0<br>0.00	0<br>0.00
Treated	Lateral II	Dry	Radular sac	*n*<br>%	0<br>0.00	0<br>0.00	24<br>48.94	25<br>51.06	0<br>0.00	0<br>0.00	0<br>0.00
Treated	Lateral I	Wet	Working zone	*n*<br>%	27<br>69.23	9<br>23.08	0<br>0.00	0<br>0.00	0<br>0.00	3<br>7.69	0<br>0.00
Treated	Lateral I	Wet	Mineralization zone	*n*<br>%	30<br>71.43	4<br>9.52	0<br>0.00	0<br>0.00	1<br>2.38	7<br>16.67	0<br>0.00
Treated	Lateral I	Wet	Radular sac	*n*<br>%	37<br>78.72	7<br>14.89	0<br>0.00	0<br>0.00	0<br>0.00	3<br>6.38	0<br>0.00
Treated	Lateral I	Dry	Working zone	*n*<br>%	0<br>0.00	0<br>0.00	35<br>100.00	0<br>0.00	0<br>0.00	0<br>0.00	0<br>0.00
Treated	Lateral I	Dry	Mineralization zone	*n*<br>%	0<br>0.00	0<br>0.00	44<br>100.00	0<br>0.00	0<br>0.00	0<br>0.00	0<br>0.00
Treated	Lateral I	Dry	Radular sac	*n*<br>%	0<br>0.00	0<br>0.00	42<br>100.00	0<br>0.00	0<br>0.00	0<br>0.00	0<br>0.00
Treated	Central	Wet	Working zone	*n*<br>%	10<br>52.63	6<br>31.58	0<br>0.00	0<br>0.00	0<br>0.00	3<br>15.79	0<br>0.00
Treated	Central	Wet	Mineralization zone	*n*<br>%	16<br>80.00	1<br>5.00	0<br>0.00	0<br>0.00	1<br>5.00	2<br>10.00	0<br>0.00
Treated	Central	Wet	Radular sac	*n*<br>%	20<br>83.33	3<br>12.50	0<br>0.00	0<br>0.00	0<br>0.00	1<br>4.17	0<br>0.00
Treated	Central	Dry	Working zone	*n*<br>%	0<br>0.00	0<br>0.00	19<br>100.00	0<br>0.00	0<br>0.00	0<br>0.00	0<br>0.00
Treated	Central	Dry	Mineralization zone	*n*<br>%	0<br>0.00	0<br>0.00	17<br>100.00	0<br>0.00	0<br>0.00	0<br>0.00	0<br>0.00
Treated	Central	Dry	Radular sac	*n*<br>%	0<br>0.00	0<br>0.00	24<br>100.00	0<br>0.00	0<br>0.00	0<br>0.00	0<br>0.00

Supplementary Table 10. For each tooth type and tooth part sorted to condition (native or treated) and radular zone (Mz, mineralization zone; Rs, radular sac; Wz, working zone). Mean (M), standard deviation (Sd), and quantity of analysed tooth areas (*n*) for the following parameters (P) are shown: proportions, given in %, of Te ('trace elements' = sum of Na, Mg, Si, P, S, Cl, K), Ca, Fe; Young's modulus (E), and hardness (H), given in GPa. ANOVA or t test were carried out to detect differences between tooth areas of each tooth type in each radular zone (orange p-values = highly significant, red = significant). U, unit of measure.

Central tooth

Condition	Zone	Parameter	U	Cusp			Stylus			T test		
				M	Sd	*n*	M	Sd	*n*	t ratio	df	p-value
Native	Rs	H	GPa	0.31	0.02	34	0.32	0.02	35	0.573561	67	0.7159
Native	Rs	E	GPa	2.61	0.16	34	2.63	0.17	35	0.574001	66.96544	0.7161
Native	Rs	Fe	%	0.01	0.07	34	0.02	0.10	35	0.258671	59.8825	0.6016
Native	Rs	Ca	%	0.08	0.25	34	0.11	0.30	35	0.451055	65.96905	0.6733
Native	Rs	Te	%	0	0	34	0.003	0.02	35	1	34	0.8378
Native	Mz	H	GPa	0.88	0.42	45	0.71	0.31	45	-2.1626	88	0.0166*
Native	Mz	E	GPa	7.34	3.47	45	5.94	2.61	45	-2.1626	81.70919	0.0167*
Native	Mz	Fe	%	0.03	0.18	45	5.94	2.61	45	0.275853	85.08007	0.6083
Native	Mz	Ca	%	0.62	0.99	45	0.04	0.15	45	-0.90826	87.00615	0.1831
Native	Mz	Te	%	0.060	0.130	45	0.07	0.17	45	0.425258	83.33998	0.6641
Native	Wz	H	GPa	1.96	0.16	35	0.42	1.10	35	-15.234	68	<.0001*
Native	Wz	E	GPa	16.53	1.37	35	1.46	0.10	35	-15.8195	57.18146	<.0001*
Native	Wz	Fe	%	0.00	0.00	35	12.20	0.86	35	1.610529	34	0.9417
Native	Wz	Ca	%	0.89	1.17	35	0.09	0.34	35	-0.18267	66.56176	0.4278
Native	Wz	Te	%	0.00	0.00	35	0.03	0.001	35	1.261312	34	0.8921
Treated	Rs	H	GPa	0.24	0.02	35	0.84	1.36	36	-6.20012	68.77351	<.0001*
Treated	Rs	E	GPa	2.04	0.13	35	0.22	0.02	36	-5.58401	68.84251	<.0001*
Treated	Rs	Fe	%	0.01	0.07	35	1.87	0.13	36	-0.01992	68.87465	0.4921
Treated	Rs	Ca	%	0.02	0.06	35	0.01	0.07	36	-0.0295	68.92521	0.4883
Treated	Rs	Te	%	0.00	0.00	35	0.03	0.17	36	1.094104	35	0.8593
Treated	Mz	H	GPa	0.71	0.34	45	0.02	0.07	44	-3.15926	77.8655	0.0011*
Treated	Mz	E	GPa	6.10	2.99	45	0.52	0.23	44	-3.02377	79.04346	0.0017*
Treated	Mz	Fe	%	0.00	0.00	45	4.45	2.10	44	1.164916	43	0.8748
Treated	Mz	Ca	%	0.01	0.03	45	0.03	0.17	44	1.054511	44.24342	0.8513
Treated	Mz	Te	%	0.004	0.024	45	0.00	0.00	44	-1	44	0.1614
Treated	Wz	H	GPa	1.48	0.11	35	0.05	0.28	35	-23.1605	57.37051	<.0001*
Treated	Wz	E	GPa	12.76	0.92	35	0.99	0.07	35	-21.6352	59.16874	<.0001*
Treated	Wz	Fe	%	0.00	0.00	35	8.71	0.61	35	1.717077	34	0.9547

Lateral tooth I

Condition	Zone	Parameter	U	Cusp			Stylus			T test		
				M	Sd	*n*	M	Sd	*n*	t ratio	df	p-value
Native	Rs	H	GPa	0.34	0.04	71	0.33	0.03	72	-1.869	141	0.0324*
Native	Rs	E	GPa	2.84	0.33	71	2.68	0.18	72	-3.73703	107.4768	0.0002*
Native	Rs	Fe	%	0.02	0.09	71	0.02	0.13	72	-0.01635	127.3844	0.4935
Native	Rs	Ca	%	0.07	0.28	71	0.00	0.00	72	-1.94657	70	0.0278*
Native	Rs	Te	%	0.013	0.054	71	0.005	0.024	72	-1.14012	95.42337	0.1285
Native	Mz	H	GPa	0.91	0.43	88	0.74	0.33	90	-2.95202	162.6483	0.0018*
Native	Mz	E	GPa	7.56	3.58	88	5.97	2.63	90	-3.3824	159.6005	0.0005*
Native	Mz	Fe	%	0.00	0.01	88	0.00	0.00	90	-1.41995	87	0.0796
Native	Mz	Ca	%	0.12	0.38	88	0.65	1.11	90	4.334803	109.7063	1
Native	Mz	Te	%	0.05	0.110	88	0.042	0.11	90	-0.51407	175.1602	0.3039
Native	Wz	H	GPa	1.99	0.17	72	1.50	0.14	70	-18.8045	137.1585	<.0001*
Native	Wz	E	GPa	16.60	1.35	72	12.15	0.99	70	-22.4109	130.3124	<.0001*
Native	Wz	Fe	%	0.00	0.00	72	0.00	0.00	70	-	-	-
Native	Wz	Ca	%	0.52	1.31	72	1.13	1.30	70	2.775944	139.9236	0.9969
Native	Wz	Te	%	0.002	0.014	72	0.00	0.00	70	-1.39843	71	0.0832
Treated	Rs	H	GPa	0.31	0.05	69	0.27	0.02	71	-7.14212	99.10572	<.0001*
Treated	Rs	E	GPa	2.41	0.34	69	2.35	0.17	71	-1.46528	98.02379v	0.073
Treated	Rs	Fe	%	0.02	0.08	69	0.02	0.12	71	-0.03323	126.1276	0.4868
Treated	Rs	Ca	%	0.00	0.00	69	0.00	0.00	71	-	-	-
Treated	Rs	Te	%	0.002	0.01	69	0.00	0.00	71	-1	68	0.1604
Treated	Mz	H	GPa	0.70	0.35	89	0.54	0.20	87	-3.84605	140.1818	<.0001*
Treated	Mz	E	GPa	5.61	2.80	89	4.81	1.80	87	-2.28433	150.1567	0.0119*
Treated	Mz	Fe	%	0.00	0.01	89	0.00	0.00	87	-1.40854	88	0.0812
Treated	Mz	Ca	%	0.02	0.09	89	0.01	0.08	87	-1.04057	168.6772	0.1498
Treated	Mz	Te	%	0.006	0.04	89	0.021	0.10	87	1.316947	107.8504	0.9052
Treated	Wz	H	GPa	1.48	0.12	70	0.97	0.09	75	-28.739	127.3146	<.0001*
Treated	Wz	E	GPa	12.28	1.11	70	8.92	0.92	75	-19.5784	133.7228	<.0001*
Treated	Wz	Fe	%	0.00	0.00	70	0.00	0.00	75	-	-	-

Lateral tooth II

Condition	Zone	Parameter	U	Cusp			Stylus			Basis			ANOVA		
				M	Sd	*n*	M	Sd	*n*	M	Sd	*n*	F Ratio	df	p-value
Native	Rs	H	GPa	1.22	0.63	71	0.49	0.21	69	0.83	0.39	71	48.7105	2	<.0001*
Native	Rs	E	GPa	9.42	4.84	71	3.74	1.63	69	6.71	3.15	71	47.7558	2	<.0001*
Native	Rs	Fe	%	2.11	2.70	71	0.16	0.21	69	0.08	0.17	71	38.1495	2	<.0001*
Native	Rs	Ca	%	1.27	1.27	71	0.65	1.13	69	1.79	1.17	71	16.3364	2	<.0001*
Native	Rs	Te	%	0.031	0.116	71	0.184	0.532	69	0.005	0.027	71	6.7523	2	0.0014*
Native	Mz	H	GPa	3.01	0.48	89	2.11	0.61	86	2.08	0.29	88	109.1505	2	<.0001*
Native	Mz	E	GPa	23.22	3.58	89	16.22	4.64	86	16.38	1.93	88	111.7183	2	<.0001*
Native	Mz	Fe	%	14.64	5.40	89	1.95	1.73	86	0.22	0.23	88	505.2928	2	<.0001*
Native	Mz	Ca	%	8.29	2.63	89	4.71	1.55	86	2.54	1.08	88	211.9182	2	<.0001*
Native	Mz	Te	%	0.418	0.794	89	0.250	0.736	86	0.367	0.720	88	1.1412	2	0.321
Native	Wz	H	GPa	3.97	0.31	71	2.57	0.19	71	2.23	0.16	72	1159.652	2	<.0001*
Native	Wz	E	GPa	30.90	1.98	71	19.90	1.29	71	17.22	1.04	72	1692.459	2	<.0001*
Native	Wz	Fe	%	26.94	2.99	71	6.35	1.03	71	0.32	0.26	72	4153.577	2	<.0001*
Native	Wz	Ca	%	5.95	1.41	71	4.28	1.28	71	3.53	1.04	72	70.4898	2	<.0001*
Native	Wz	Te	%	1.582	2.374	71	0.433	0.726	71	0.588	1.127	72	11.1832	2	<.0001*
Treated	Rs	H	GPa	0.91	0.39	71	0.33	0.11	72	0.63	0.29	73	73.9073	2	<.0001*
Treated	Rs	E	GPa	7.13	3.45	71	2.48	0.83	72	5.01	1.64	73	76.3472	2	<.0001*
Treated	Rs	Fe	%	2.33	2.91	71	0.16	0.22	72	0.08	0.16	73	40.9484	2	<.0001*
Treated	Rs	Ca	%	0.05	0.16	71	0.02	0.07	72	0.09	0.27	73	2.6216	2	0.0751
Treated	Rs	Te	%	0.00	0.02	71	0.00	0.01	72	0.00	0.019	73	0.1431	2	0.8668
Treated	Mz	H	GPa	2.39	0.48	88	1.22	0.32	86	2.00	0.29	90	226.3026	2	<.0001*
Treated	Mz	E	GPa	19.59	3.83	88	9.29	2.45	86	7.20	1.04	90	534.0206	2	<.0001*
Treated	Mz	Fe	%	15.42	5.78	88	2.99	3.38	86	0.21	0.23	90	386.7115	2	<.0001*
Treated	Mz	Ca	%	0.08	0.32	88	0.14	0.41	86	0.05	0.21	90	1.8764	2	0.1552
Treated	Mz	Te	%	0.00	0.00	88	0.004	0.02	86	0.012	0.09	90	1.2598	2	0.2854
Treated	Wz	H	GPa	3.84	0.33	74	1.41	0.12	70	1.68	0.25	71	2027.849	2	<.0001*
Treated	Wz	E	GPa	29.88	2.49	74	10.80	0.77	70	9.95	0.97	71	3501.158	2	<.0001*
Treated	Wz	Fe	%	28.62	3.79	74	8.34	2.01	70	0.32	0.26	71	2454.757	2	<.0001*

Marginal tooth

Condition	Zone	Parameter	U	Cusp			Stylus			Basis			ANOVA		
				M	Sd	*n*	M	Sd	*n*	M	Sd	*n*	F Ratio	df	p-value
Native	Rs	H	GPa	0.57	0.27	71	0.44	0.16	71	0.45	0.16	70	8.298	2	0.0003*
Native	Rs	E	GPa	4.36	2.09	71	3.64	1.36	71	3.49	1.25	70	5.8346	2	0.0034*
Native	Rs	Fe	%	0.03	0.19	71	0.05	0.20	71	0.00	0.00	70	1.6688	2	0.191
Native	Rs	Ca	%	0.24	0.54	71	0.20	0.48	71	0.12	0.40	70	1.0621	2	0.3476
Native	Rs	Te	%	0.011	0.046	71	0.004	0.02	71	0.052	0.168	70	4.6504	2	0.0106*
Native	Mz	H	GPa	1.81	0.34	90	1.46	0.29	88	0.84	0.14	88	290.3843	2	<.0001*
Native	Mz	E	GPa	13.91	2.59	90	12.17	2.42	88	6.43	1.06	88	2553.45	2	<.0001*
Native	Mz	Fe	%	0.00	0.00	90	0.00	0.03	88	0.00	0.00	88	2.0394	2	0.1322
Native	Mz	Ca	%	0.27	0.53	90	0.49	0.69	88	0.14	0.39	88	9.0992	2	0.0002*
Native	Mz	Te	%	0.067	0.137	90	0.029	0.073	88	0.108	0.35	88	2.8637	2	0.0588
Native	Wz	H	GPa	2.11	0.18	72	1.85	0.10	70	0.97	0.09	72	1544.57	2	<.0001*
Native	Wz	E	GPa	16.88	1.00	72	15.38	0.86	70	7.52	0.62	72	2553.45	2	<.0001*
Native	Wz	Fe	%	0.00	0.00	72	0.01	0.08	70	0.00	0.00	72	2.0807	2	0.1274
Native	Wz	Ca	%	0.36	0.49	72	1.24	1.47	70	0.35	0.84	72	17.7966	2	<.0001*
Native	Wz	Te	%	0.00	0.00	72	0.00	0.00	70	0.068	0.495	72	1.337	2	0.2648
Treated	Rs	H	GPa	0.40	0.20	69	0.44	0.12	71	0.41	0.14	72	0.7896	2	0.4554
Treated	Rs	E	GPa	3.54	1.24	69	3.38	0.95	71	3.13	1.06	72	2.5337	2	0.0818
Treated	Rs	Fe	%	0.03	0.20	69	0.05	0.20	71	0.00	0.00	72	1.6058	2	0.2032
Treated	Rs	Ca	%	0.00	0.00	69	0.01	0.06	71	0.00	0.02	72	2.0946	2	0.1257
Treated	Rs	Te	%	0.00	0.00	69	0.00	0.01	71	0.00	0.00	72	1	2	0.3696
Treated	Mz	H	GPa	1.28	0.18	90	1.20	0.28	89	0.56	0.10	88	333.122	2	<.0001*
Treated	Mz	E	GPa	9.21	1.18	90	7.32	0.94	89	4.22	0.76	88	590.2058	2	<.0001*
Treated	Mz	Fe	%	0.00	0.00	90	0.00	0.03	89	0.00	0.00	88	2.0031	2	0.137
Treated	Mz	Ca	%	0.04	0.12	90	0.04	0.11	89	0.03	0.09	88	0.4424	2	0.643
Treated	Mz	Te	%	0.00	0.01	90	0.00	0.01	89	0.00	0.00	88	0.502	2	0.6059
Treated	Wz	H	GPa	1.29	0.10	70	1.24	0.19	71	0.62	0.06	69	589.485	2	<.0001*
Treated	Wz	E	GPa	9.62	0.54	70	8.84	1.13	71	5.11	0.59	69	629.7949	2	<.0001*
Treated	Wz	Fe	%	0.00	0.00	70	0.01	0.08	71	0.00	0.00	69	1.966	2	0.1426

Treated	Wz	Ca	%	0.07	0.19	35	0.06	0.20	35	0.047975	65.8488	0.5191	0.00	0.00	70	0.00	0.00	75	-	-	-	0.09	0.36	74	0.45	0.69	70	0.06	0.31	71	14.3309	2	<.0001*	0.08	0.13	70	0.017	0.20	71	0.02	0.12	69	3.0962	2	0.0473*
Treated	Wz	Te	%	0.00	0.00	35	0.01	0.05	35	1.628022	34	0.9436	0.00	0.00	70	0.00	0.00	75	-	-	-	0.00	0.02	74	0.00	0.02	70	0.02	0.01	71	1.4271	2	0.2423	0.00	0.00	70	0.00	0.00	71	0.01	0.03	69	1.8301	2	0.163

Supplementary Table 11. For each tooth type and tooth part sorted to condition (native or treated) and radular tooth row (Tr). Mean (M) and standard deviation (Sd) for the following parameters (P) are shown: proportions, given in %, of Te ('trace elements' = sum of Na, Mg, Si, P, S, Cl, K), Ca, Fe, and all elements (Ae), Young's modulus (E) and hardness (H), given in GPa. Mz, mineralization zone; Rs, radular sac; Rz, radular zone; U, unit of measure; Wz, working zone.

Condition	Rz	Tr	P	U	Central tooth				Lateral tooth I				Lateral tooth II						Marginal tooth					
					Stylus		Cusp		Stylus		Cusp		Basis		Stylus		Cusp		Basis		Stylus		Cusp	
					M	Sd	M	Sd	M	Sd	M	Sd	M	Sd	M	Sd	M	Sd	M	Sd	M	Sd	M	Sd
Native	Rs	1	Ae	%	0.00	0.00	0.00	0.00	0.00	0.00	0.00	0.00	0.00	1.47	0.21	0.35	0.00	0.00	0.06	0.16	0.00	0.00	0.38	0.59
Native	Rs	1	Ca	%	0.00	0.00	0.00	0.00	0.00	0.00	0.00	0.00	0.00	0.00	0.00	0.00	0.00	0.00	0.00	0.00	0.00	0.00	0.00	0.00
Native	Rs	1	E	GPa	2.50	0.13	2.50	0.13	2.66	0.20	2.77	0.28	2.43	0.12	2.10	0.12	2.31	0.13	2.36	0.29	2.55	0.48	2.62	0.50
Native	Rs	1	Fe	%	0.00	0.00	0.00	0.00	0.00	0.00	0.00	0.00	0.00	0.00	0.00	0.00	0.00	0.00	0.00	0.00	0.00	0.00	0.38	0.59
Native	Rs	1	H	GPa	0.30	0.02	0.30	0.02	0.33	0.03	0.33	0.03	0.30	0.02	0.27	0.02	0.30	0.02	0.31	0.04	0.31	0.06	0.34	0.07
Native	Rs	1	Te	%	0.00	0.00	0.00	0.00	0.00	0.00	0.00	0.00	0.00	0.00	0.21	0.35	0.00	0.00	0.06	0.16	0.00	0.00	0.00	0.00
Native	Rs	2	Ae	%	0.12	0.21	0.00	0.00	0.00	0.00	0.00	0.00	0.00	0.00	0.01	0.01	0.02	0.06	0.11	0.26	0.00	0.00	0.00	0.00
Native	Rs	2	Ca	%	0.12	0.21	0.00	0.00	0.00	0.00	0.00	0.00	0.00	0.00	0.00	0.00	0.00	0.00	0.00	0.00	0.00	0.00	0.00	0.00
Native	Rs	2	E	GPa	2.59	0.17	2.61	0.06	2.69	0.23	2.89	0.43	2.54	0.13	2.23	0.13	3.37	0.19	2.52	0.17	2.72	0.19	2.50	0.37
Native	Rs	2	Fe	%	0.00	0.00	0.00	0.00	0.00	0.00	0.00	0.00	0.00	0.00	0.01	0.01	0.00	0.00	0.00	0.00	0.00	0.00	0.00	0.00
Native	Rs	2	H	GPa	0.31	0.02	0.31	0.01	0.33	0.03	0.35	0.05	0.31	0.02	0.29	0.02	0.44	0.03	0.33	0.03	0.33	0.02	0.32	0.05
Native	Rs	2	Te	%	0.00	0.00	0.00	0.00	0.00	0.00	0.00	0.00	0.00	0.00	0.00	0.00	0.02	0.06	0.11	0.26	0.00	0.00	0.00	0.00
Native	Rs	3	Ae	%	0.00	0.00	0.03	0.06	0.00	0.00	0.00	0.00	0.00	0.00	0.19	0.29	0.77	0.98	0.17	0.37	0.28	0.44	0.00	0.00
Native	Rs	3	Ca	%	0.00	0.00	0.03	0.06	0.00	0.00	0.00	0.00	0.00	0.00	0.00	0.00	0.67	1.03	0.00	0.00	0.00	0.00	0.00	0.00
Native	Rs	3	E	GPa	2.65	0.22	2.59	0.17	2.62	0.15	2.73	0.22	3.58	0.19	2.25	0.13	4.43	0.25	2.59	0.28	2.85	0.24	2.81	0.82
Native	Rs	3	Fe	%	0.00	0.00	0.00	0.00	0.00	0.00	0.00	0.00	0.00	0.00	0.00	0.00	0.00	0.00	0.00	0.00	0.28	0.44	0.00	0.00
Native	Rs	3	H	GPa	0.32	0.03	0.31	0.02	0.32	0.02	0.33	0.03	0.44	0.03	0.29	0.02	0.58	0.04	0.34	0.05	0.34	0.03	0.37	0.11
Native	Rs	3	Te	%	0.00	0.00	0.00	0.00	0.00	0.00	0.00	0.00	0.00	0.00	0.19	0.29	0.10	0.25	0.17	0.37	0.00	0.00	0.00	0.00
Native	Rs	4	Ae	%	0.00	0.00	0.00	0.00	0.00	0.00	0.50	0.78	2.29	0.00	0.30	0.04	0.31	0.20	0.00	0.00	0.21	0.33	0.00	0.00
Native	Rs	4	Ca	%	0.00	0.00	0.00	0.00	0.00	0.00	0.50	0.78	2.29	1.50	0.30	0.04	0.23	0.02	0.00	0.00	0.21	0.33	0.00	0.00
Native	Rs	4	E	GPa	2.66	0.22	2.67	0.25	2.66	0.18	2.86	0.38	4.32	0.22	2.32	0.13	5.16	0.29	3.15	0.51	2.96	0.68	2.72	0.83
Native	Rs	4	Fe	%	0.00	0.00	0.00	0.00	0.00	0.00	0.00	0.00	0.00	0.00	0.00	0.00	0.00	0.00	0.00	0.00	0.00	0.00	0.00	0.00
Native	Rs	4	H	GPa	0.32	0.03	0.32	0.03	0.33	0.03	0.34	0.05	0.53	0.04	0.30	0.02	0.67	0.05	0.41	0.07	0.35	0.08	0.35	0.11
Native	Rs	4	Te	%	0.00	0.00	0.00	0.00	0.00	0.00	0.00	0.00	0.00	0.00	0.00	0.00	0.08	0.20	0.00	0.00	0.00	0.00	0.00	0.00
Native	Rs	5	Ae	%	0.17	0.30	0.00	0.00	0.00	0.00	0.13	0.20	2.25	1.50	0.24	0.19	0.38	0.10	0.21	0.32	0.28	0.44	0.04	0.06
Native	Rs	5	Ca	%	0.17	0.30	0.00	0.00	0.00	0.00	0.00	0.00	2.25	0.10	0.16	0.03	0.38	0.10	0.00	0.00	0.00	0.00	0.04	0.06
Native	Rs	5	E	GPa	2.52	0.13	2.65	0.25	2.69	0.21	2.81	0.30	4.67	0.23	2.32	0.13	6.56	0.37	3.12	0.61	2.81	0.82	2.81	0.78
Native	Rs	5	Fe	%	0.00	0.00	0.00	0.00	0.00	0.00	0.13	0.20	0.00	0.00	0.00	0.00	0.00	0.00	0.00	0.00	0.28	0.44	0.00	0.00
Native	Rs	5	H	GPa	0.30	0.02	0.32	0.03	0.33	0.03	0.34	0.04	0.58	0.04	0.30	0.02	0.85	0.06	0.41	0.08	0.34	0.10	0.36	0.10
Native	Rs	5	Te	%	0.00	0.00	0.00	0.00	0.00	0.00	0.00	0.00	0.00	0.00	0.08	0.20	0.00	0.00	0.21	0.32	0.00	0.00	0.00	0.00
Native	Rs	6	Ae	%	0.00	0.00	0.05	0.07	0.00	0.00	0.26	0.41	2.72	0.10	0.93	0.78	1.91	0.81	0.12	0.27	0.33	0.50	0.21	0.33
Native	Rs	6	Ca	%	0.00	0.00	0.05	0.07	0.00	0.00	0.26	0.41	2.72	0.67	0.55	0.41	1.53	0.25	0.12	0.27	0.33	0.50	0.21	0.33
Native	Rs	6	E	GPa	2.59	0.13	2.48	0.06	2.62	0.14	2.95	0.48	5.78	0.29	3.07	0.19	9.44	0.53	3.76	1.02	2.77	0.80	3.37	1.20
Native	Rs	6	Fe	%	0.00	0.00	0.00	0.00	0.00	0.00	0.00	0.00	0.00	0.00	0.00	0.00	0.38	0.59	0.00	0.00	0.00	0.00	0.00	0.00
Native	Rs	6	H	GPa	0.31	0.02	0.30	0.01	0.32	0.02	0.35	0.06	0.71	0.05	0.39	0.02	1.23	0.08	0.48	0.14	0.33	0.10	0.44	0.16
Native	Rs	6	Te	%	0.00	0.00	0.00	0.00	0.00	0.00	0.00	0.00	0.00	0.00	0.38	0.85	0.00	0.00	0.00	0.00	0.00	0.00	0.00	0.00
Native	Rs	7	Ae	%	0.00	0.00	0.21	0.37	0.00	0.00	0.00	0.00	3.19	0.67	1.32	1.19	1.75	0.34	0.15	0.12	0.00	0.00	0.09	0.14
Native	Rs	7	Ca	%	0.00	0.00	0.21	0.37	0.00	0.00	0.00	0.00	3.06	0.64	0.43	0.23	1.24	0.72	0.15	0.12	0.00	0.00	0.09	0.14
Native	Rs	7	E	GPa	2.61	0.18	2.65	0.18	2.67	0.17	2.76	0.23	6.97	0.35	3.39	0.19	10.94	0.62	3.33	1.30	3.40	1.16	3.75	1.13
Native	Rs	7	Fe	%	0.00	0.00	0.00	0.00	0.00	0.00	0.00	0.00	0.14	0.13	0.15	0.05	0.52	0.38	0.00	0.00	0.00	0.00	0.00	0.00
Native	Rs	7	H	GPa	0.31	0.02	0.32	0.02	0.33	0.03	0.33	0.03	0.86	0.06	0.44	0.03	1.42	0.10	0.43	0.17	0.41	0.14	0.49	0.15

Native	Rs	7	Te	%	0.00	0.00	0.00	0.00	0.00	0.00	0.00	0.00	0.00	0.00	0.74	1.15	0.00	0.00	0.00	0.00	0.00	0.00	0.00	0.00
Native	Rs	8	Ae	%	0.00	0.00	0.36	0.35	0.00	0.00	0.00	0.00	2.33	0.76	1.10	0.92	4.24	0.96	0.58	0.89	0.00	0.00	0.54	0.83
Native	Rs	8	Ca	%	0.00	0.00	0.23	0.40	0.00	0.00	0.00	0.00	2.12	0.29	0.43	0.16	1.97	0.67	0.58	0.89	0.00	0.00	0.54	0.83
Native	Rs	8	E	GPa	2.72	0.25	2.65	0.22	2.69	0.19	2.91	0.41	8.02	0.40	4.20	0.24	11.81	0.67	3.72	1.79	3.64	0.96	4.78	1.33
Native	Rs	8	Fe	%	0.00	0.00	0.13	0.23	0.00	0.00	0.00	0.00	0.21	0.33	0.26	0.08	2.27	0.41	0.00	0.00	0.00	0.00	0.00	0.00
Native	Rs	8	H	GPa	0.33	0.03	0.32	0.03	0.33	0.03	0.35	0.05	0.99	0.07	0.55	0.04	1.53	0.11	0.48	0.23	0.44	0.12	0.62	0.17
Native	Rs	8	Te	%	0.00	0.00	0.00	0.00	0.00	0.00	0.00	0.00	0.00	0.00	0.42	1.02	0.00	0.00	0.00	0.00	0.00	0.00	0.00	0.00
Native	Rs	9	Ae	%	0.20	0.35	0.00	0.00	0.00	0.00	0.00	0.00	2.13	0.43	0.51	0.07	6.36	0.73	0.00	0.00	0.21	0.33	0.89	0.91
Native	Rs	9	Ca	%	0.00	0.00	0.00	0.00	0.00	0.00	0.00	0.00	2.01	0.19	0.35	0.10	1.61	0.46	0.00	0.00	0.21	0.33	0.89	0.91
Native	Rs	9	E	GPa	2.73	0.25	2.66	0.22	2.74	0.25	2.84	0.31	9.14	0.46	4.45	0.25	13.64	0.77	3.99	1.55	3.72	0.90	5.41	0.97
Native	Rs	9	Fe	%	0.20	0.35	0.00	0.00	0.00	0.00	0.00	0.00	0.12	0.10	0.16	0.04	4.75	0.45	0.00	0.00	0.00	0.00	0.00	0.00
Native	Rs	9	H	GPa	0.33	0.03	0.32	0.03	0.34	0.03	0.34	0.04	1.13	0.08	0.58	0.04	1.77	0.12	0.52	0.20	0.45	0.11	0.70	0.13
Native	Rs	9	Te	%	0.00	0.00	0.00	0.00	0.00	0.00	0.00	0.00	0.00	0.00	0.00	0.00	0.00	0.00	0.00	0.00	0.00	0.00	0.00	0.00
Native	Rs	10	Ae	%	0.27	0.47	0.00	0.00	0.00	0.00	0.00	0.00	2.46	0.11	0.76	0.39	6.46	2.25	0.56	0.87	0.65	1.01	0.50	0.68
Native	Rs	10	Ca	%	0.27	0.47	0.00	0.00	0.00	0.00	0.00	0.00	2.36	0.41	0.58	0.39	1.42	1.02	0.56	0.87	0.65	1.01	0.50	0.68
Native	Rs	10	E	GPa	2.60	0.14	2.53	0.14	2.65	0.14	2.85	0.32	10.26	0.51	5.51	0.31	14.34	0.88	4.11	1.47	4.78	0.91	6.46	0.81
Native	Rs	10	Fe	%	0.00	0.00	0.00	0.00	0.00	0.00	0.00	0.00	0.09	0.15	0.18	0.06	5.03	1.25	0.00	0.00	0.00	0.00	0.00	0.00
Native	Rs	10	H	GPa	0.31	0.02	0.30	0.02	0.33	0.02	0.34	0.04	1.27	0.09	0.72	0.05	1.83	0.13	0.53	0.19	0.57	0.11	0.85	0.09
Native	Rs	10	Te	%	0.00	0.00	0.00	0.00	0.00	0.00	0.00	0.00	0.00	0.00	0.00	0.00	0.00	0.00	0.00	0.00	0.00	0.00	0.00	0.00
Native	Rs	11	Ae	%	0.60	0.84	0.40	0.69	0.29	0.42	0.10	0.15	2.35	0.33	1.80	1.30	8.13	1.83	0.08	0.12	0.43	0.61	0.03	0.06
Native	Rs	11	Ca	%	0.60	0.84	0.40	0.69	0.00	0.00	0.00	0.00	1.98	0.19	1.16	1.31	2.25	1.12	0.00	0.00	0.39	0.60	0.00	0.00
Native	Rs	11	E	GPa	2.72	0.04	2.61	0.13	2.71	0.19	2.79	0.29	10.95	0.55	6.34	0.36	15.76	0.89	4.37	1.36	5.41	0.47	7.16	0.82
Native	Rs	11	Fe	%	0.00	0.00	0.00	0.00	0.25	0.39	0.00	0.00	0.32	0.25	0.58	0.08	5.83	1.25	0.00	0.00	0.00	0.00	0.00	0.00
Native	Rs	11	H	GPa	0.33	0.00	0.31	0.02	0.33	0.03	0.34	0.04	1.35	0.09	0.82	0.06	2.05	0.14	0.57	0.18	0.65	0.06	0.93	0.11
Native	Rs	11	Te	%	0.00	0.00	0.00	0.00	0.04	0.07	0.10	0.15	0.05	0.08	0.06	0.10	0.04	0.07	0.08	0.12	0.04	0.06	0.03	0.06
Native	Rs	12	Ae	%	0.36	0.53	0.00	0.00	0.01	0.04	0.19	0.24	2.44	0.13	4.47	1.65	11.07	2.12	0.01	0.03	0.65	0.88	0.70	0.92
Native	Rs	12	Ca	%	0.32	0.56	0.00	0.00	0.00	0.00	0.00	0.00	2.34	0.39	3.77	1.29	3.92	1.11	0.00	0.00	0.65	0.88	0.60	0.93
Native	Rs	12	E	GPa	2.72	0.19	2.64	0.18	2.72	0.20	2.94	0.43	11.38	0.57	6.58	0.37	16.10	0.91	4.74	1.12	6.54	0.50	8.21	0.83
Native	Rs	12	Fe	%	0.00	0.00	0.00	0.00	0.00	0.00	0.13	0.20	0.10	0.16	0.54	0.17	7.04	1.58	0.00	0.00	0.00	0.00	0.00	0.00
Native	Rs	12	H	GPa	0.33	0.02	0.32	0.02	0.34	0.03	0.35	0.05	1.41	0.10	0.86	0.06	2.09	0.14	0.62	0.15	0.78	0.06	1.07	0.12
Native	Rs	12	Te	%	0.03	0.06	0.00	0.00	0.01	0.04	0.06	0.10	0.00	0.00	0.16	0.31	0.12	0.23	0.01	0.03	0.01	0.02	0.10	0.12
Native	Mz	13	Ae	%	0.66	1.15	0.16	0.28	0.00	0.00	0.01	0.02	2.34	0.37	4.03	1.50	15.27	2.90	0.02	0.02	0.83	1.28	1.34	0.91
Native	Mz	13	Ca	%	0.60	1.03	0.16	0.28	0.00	0.00	0.00	0.00	2.25	0.12	3.82	1.40	6.29	2.43	0.00	0.00	0.80	1.24	1.28	0.99
Native	Mz	13	E	GPa	2.68	0.22	2.69	0.23	2.62	0.13	2.80	0.24	11.60	0.58	7.65	0.43	17.88	1.01	5.04	0.96	7.53	0.52	9.41	0.85
Native	Mz	13	Fe	%	0.07	0.12	0.00	0.00	0.00	0.00	0.00	0.00	0.03	0.05	0.14	0.04	8.72	1.44	0.00	0.00	0.00	0.00	0.00	0.00
Native	Mz	13	H	GPa	0.32	0.03	0.32	0.03	0.32	0.02	0.34	0.03	1.43	0.10	0.99	0.07	2.32	0.16	0.65	0.13	0.90	0.06	1.22	0.12
Native	Mz	13	Te	%	0.00	0.00	0.00	0.00	0.00	0.00	0.01	0.02	0.06	0.10	0.07	0.17	0.26	0.60	0.02	0.02	0.03	0.06	0.06	0.14
Native	Mz	14	Ae	%	0.30	0.52	1.57	1.39	0.08	0.08	0.13	0.11	3.71	0.22	4.36	0.75	15.24	3.15	0.10	0.08	0.06	0.05	0.04	0.06
Native	Mz	14	Ca	%	0.00	0.00	1.57	1.39	0.00	0.00	0.00	0.00	3.31	1.01	3.59	0.90	6.20	2.93	0.00	0.00	0.00	0.00	0.00	0.00
Native	Mz	14	E	GPa	2.59	0.13	2.75	0.22	2.66	0.14	2.71	0.19	13.62	0.68	9.19	0.58	19.24	1.09	5.02	0.99	8.64	0.50	10.39	0.86
Native	Mz	14	Fe	%	0.30	0.52	0.00	0.00	0.00	0.00	0.00	0.00	0.04	0.07	0.58	0.14	8.99	0.87	0.00	0.00	0.00	0.00	0.00	0.00
Native	Mz	14	H	GPa	0.31	0.02	0.33	0.03	0.33	0.02	0.33	0.02	1.68	0.12	1.17	0.07	2.50	0.17	0.65	0.13	1.04	0.06	1.35	0.12
Native	Mz	14	Te	%	0.00	0.00	0.00	0.00	0.08	0.08	0.13	0.11	0.35	0.30	0.19	0.21	0.04	0.07	0.10	0.08	0.06	0.05	0.04	0.06
Native	Mz	15	Ae	%	0.15	0.14	0.00	0.00	1.34	0.85	0.26	0.32	3.04	1.28	4.32	1.50	16.97	1.62	0.00	0.00	0.25	0.39	1.14	0.81
Native	Mz	15	Ca	%	0.00	0.00	0.00	0.00	1.23	1.02	0.23	0.35	2.93	0.26	3.72	1.34	7.36	0.91	0.00	0.00	0.25	0.39	1.10	0.86
Native	Mz	15	E	GPa	2.60	0.13	2.67	0.14	2.65	0.13	2.73	0.19	15.40	0.77	9.70	0.58	19.77	1.12	5.15	0.36	10.04	0.57	11.37	0.66
Native	Mz	15	Fe	%	0.00	0.00	0.00	0.00	0.00	0.00	0.00	0.00	0.07	0.11	0.54	0.09	9.61	0.88	0.00	0.00	0.00	0.00	0.00	0.00
Native	Mz	15	H	GPa	0.31	0.02	0.32	0.02	0.33	0.02	0.33	0.02	1.90	0.13	1.28	0.09	2.57	0.18	0.68	0.05	1.20	0.07	1.48	0.10
Native	Mz	15	Te	%	0.15	0.14	0.00	0.00	0.12	0.18	0.04	0.06	0.03	0.05	0.06	0.08	0.00	0.00	0.00	0.00	0.00	0.00	0.03	0.05
Native	Mz	16	Ae	%	0.18	0.19	0.61	0.70	0.36	0.65	0.26	0.23	3.40	0.36	5.15	1.57	19.33	3.72	0.79	0.98	1.17	0.93	0.30	0.35
Native	Mz	16	Ca	%	0.00	0.00	0.00	0.00	0.28	0.68	0.00	0.00	2.83	0.76	3.86	1.17	6.15	2.12	0.66	1.03	1.06	0.88	0.21	0.33
Native	Mz	16	E	GPa	3.53	0.18	3.52	0.18	3.49	0.17	3.59	0.28	15.49	0.78	11.18	0.63	19.87	1.12	5.49	0.31	10.53	0.60	12.01	0.76
Native	Mz	16	Fe	%	0.00	0.00	0.40	0.69	0.00	0.00	0.00	0.00	0.44	0.30	0.84	0.23	12.59	2.75	0.00	0.00	0.00	0.00	0.00	0.00
Native	Mz	16	H	GPa	0.42	0.02	0.42	0.02	0.43	0.03	0.43	0.03	1.91	0.13	1.45	0.10	2.58	0.18	0.71	0.05	1.26	0.07	1.56	0.12

Native	Mz	16	Te	%	0.18	0.19	0.21	0.08	0.08	0.10	0.26	0.23	0.13	0.11	0.45	0.62	0.59	0.93	0.13	0.17	0.11	0.13	0.09	0.10
Native	Mz	17	Ae	%	0.46	0.38	1.14	0.57	0.31	0.76	0.07	0.14	3.13	0.83	5.26	1.59	19.77	1.50	0.05	0.08	0.27	0.32	0.26	0.23
Native	Mz	17	Ca	%	0.00	0.00	0.95	0.86	0.31	0.76	0.00	0.00	2.78	0.13	4.61	1.44	7.86	2.16	0.00	0.00	0.15	0.23	0.00	0.00
Native	Mz	17	E	GPa	3.42	0.17	4.66	0.24	3.49	0.18	4.74	0.33	16.11	0.89	13.56	0.60	20.94	1.19	6.08	0.80	10.99	0.62	12.34	0.70
Native	Mz	17	Fe	%	0.00	0.00	0.00	0.00	0.00	0.00	0.03	0.05	0.35	0.31	0.56	0.48	11.52	0.92	0.00	0.00	0.00	0.00	0.00	0.00
Native	Mz	17	H	GPa	0.41	0.02	0.56	0.03	0.43	0.03	0.57	0.04	2.00	0.15	1.77	0.20	2.72	0.19	0.79	0.11	1.32	0.07	1.60	0.11
Native	Mz	17	Te	%	0.46	0.38	0.18	0.31	0.00	0.00	0.04	0.10	0.00	0.00	0.10	0.24	0.39	0.89	0.05	0.08	0.12	0.12	0.26	0.23
Native	Mz	18	Ae	%	0.11	0.18	1.10	0.78	0.62	1.19	0.08	0.11	3.54	0.18	4.06	1.54	20.46	3.25	0.26	0.32	0.82	0.43	0.12	0.13
Native	Mz	18	Ca	%	0.00	0.00	1.10	0.78	0.51	1.24	0.00	0.00	2.85	0.28	3.44	1.29	6.42	2.32	0.00	0.00	0.80	0.42	0.00	0.00
Native	Mz	18	E	GPa	4.69	0.24	5.61	0.28	4.61	0.23	5.89	0.41	16.18	0.81	15.44	0.93	21.48	1.22	6.31	0.81	11.44	0.65	12.86	0.73
Native	Mz	18	Fe	%	0.00	0.00	0.00	0.00	0.00	0.00	0.00	0.00	0.03	0.05	0.52	0.17	13.88	1.33	0.00	0.00	0.00	0.00	0.00	0.00
Native	Mz	18	H	GPa	0.56	0.03	0.67	0.03	0.57	0.04	0.71	0.05	2.00	0.14	2.04	0.15	2.79	0.19	0.82	0.11	1.37	0.08	1.67	0.12
Native	Mz	18	Te	%	0.11	0.18	0.00	0.00	0.11	0.13	0.08	0.11	0.66	1.18	0.10	0.14	0.17	0.27	0.26	0.32	0.02	0.04	0.12	0.13
Native	Mz	19	Ae	%	0.00	0.00	0.25	0.17	0.73	1.14	0.05	0.09	3.44	1.09	7.60	2.08	18.99	1.90	0.04	0.08	0.33	0.80	0.05	0.11
Native	Mz	19	Ca	%	0.00	0.00	0.25	0.17	0.68	1.06	0.00	0.00	3.17	1.17	6.25	1.30	7.35	0.96	0.00	0.00	0.33	0.80	0.00	0.00
Native	Mz	19	E	GPa	5.28	0.27	6.71	0.34	5.39	0.27	7.04	0.49	17.33	0.81	16.66	0.94	22.13	1.36	6.15	0.37	11.90	0.67	13.38	0.76
Native	Mz	19	Fe	%	0.00	0.00	0.00	0.00	0.00	0.00	0.00	0.00	0.23	0.35	0.77	0.73	11.55	1.22	0.00	0.00	0.00	0.00	0.00	0.00
Native	Mz	19	H	GPa	0.63	0.03	0.80	0.04	0.67	0.05	0.85	0.06	2.14	0.15	2.16	0.15	2.83	0.20	0.81	0.06	1.43	0.08	1.74	0.12
Native	Mz	19	Te	%	0.00	0.00	0.00	0.00	0.05	0.12	0.05	0.09	0.04	0.07	0.58	1.32	0.09	0.19	0.04	0.08	0.00	0.00	0.05	0.11
Native	Mz	20	Ae	%	0.42	0.38	0.47	0.41	0.82	1.33	0.00	0.00	2.38	1.18	5.97	1.61	21.03	2.45	0.51	0.55	0.00	0.00	0.39	0.31
Native	Mz	20	Ca	%	0.42	0.38	0.47	0.41	0.82	1.33	0.00	0.00	2.26	0.28	4.72	1.08	10.07	1.60	0.51	0.55	0.00	0.00	0.39	0.31
Native	Mz	20	E	GPa	5.85	0.30	7.91	0.40	5.73	0.29	7.98	0.55	16.95	1.07	17.27	0.98	22.56	1.28	6.43	0.36	12.36	0.70	13.90	0.79
Native	Mz	20	Fe	%	0.00	0.00	0.00	0.00	0.00	0.00	0.00	0.00	0.04	0.06	1.04	0.32	10.68	1.57	0.00	0.00	0.00	0.00	0.00	0.00
Native	Mz	20	H	GPa	0.70	0.04	0.95	0.05	0.71	0.05	0.96	0.07	2.17	0.12	2.24	0.16	2.93	0.20	0.84	0.06	1.48	0.08	1.81	0.12
Native	Mz	20	Te	%	0.00	0.00	0.00	0.00	0.00	0.00	0.00	0.00	0.08	0.17	0.21	0.51	0.28	0.68	0.00	0.00	0.00	0.00	0.00	0.00
Native	Mz	21	Ae	%	0.14	0.24	0.26	0.45	1.11	1.28	0.00	0.00	3.09	0.27	7.63	1.28	25.22	2.27	0.02	0.04	0.33	0.51	0.00	0.00
Native	Mz	21	Ca	%	0.14	0.24	0.26	0.45	1.11	1.28	0.00	0.00	2.11	0.36	6.30	1.35	10.39	1.95	0.00	0.00	0.33	0.51	0.00	0.00
Native	Mz	21	E	GPa	6.28	0.32	7.80	0.40	6.40	0.32	8.19	0.57	17.03	0.96	18.74	1.06	24.05	1.36	6.66	0.38	12.82	0.73	14.42	0.82
Native	Mz	21	Fe	%	0.00	0.00	0.00	0.00	0.00	0.00	0.00	0.00	0.19	0.15	1.21	0.17	13.72	1.03	0.00	0.00	0.00	0.00	0.00	0.00
Native	Mz	21	H	GPa	0.75	0.04	0.94	0.05	0.79	0.05	0.98	0.07	2.21	0.15	2.43	0.17	3.12	0.22	0.87	0.06	1.54	0.09	1.87	0.13
Native	Mz	21	Te	%	0.00	0.00	0.00	0.00	0.00	0.00	0.00	0.00	0.79	1.67	0.12	0.29	1.11	1.36	0.02	0.04	0.00	0.00	0.00	0.00
Native	Mz	22	Ae	%	1.38	1.30	0.53	0.06	0.64	1.13	0.64	0.99	3.12	1.87	8.50	2.39	25.32	2.94	0.16	0.19	0.18	0.18	0.16	0.26
Native	Mz	22	Ca	%	1.38	1.30	0.53	0.06	0.64	1.13	0.64	0.99	2.00	1.15	6.37	1.41	11.21	2.92	0.00	0.00	0.00	0.00	0.00	0.00
Native	Mz	22	E	GPa	7.02	0.36	9.29	0.47	6.85	0.34	9.35	0.65	17.48	0.99	19.78	1.22	24.37	1.38	6.90	0.39	13.06	0.61	14.94	0.85
Native	Mz	22	Fe	%	0.00	0.00	0.00	0.00	0.00	0.00	0.00	0.00	0.21	0.07	2.10	1.09	13.79	1.04	0.00	0.00	0.08	0.10	0.00	0.00
Native	Mz	22	H	GPa	0.84	0.04	1.12	0.06	0.85	0.06	1.12	0.08	2.27	0.16	2.61	0.14	3.17	0.22	0.90	0.06	1.57	0.07	1.94	0.13
Native	Mz	22	Te	%	0.00	0.00	0.00	0.00	0.00	0.00	0.00	0.00	0.91	0.97	0.03	0.07	0.31	0.61	0.16	0.19	0.10	0.16	0.16	0.26
Native	Mz	23	Ae	%	0.56	0.44	0.71	0.53	0.62	0.40	0.16	0.18	2.54	1.29	9.24	1.89	24.73	3.24	1.06	0.58	0.65	0.12	0.66	0.28
Native	Mz	23	Ca	%	0.40	0.41	0.46	0.45	0.42	0.47	0.00	0.00	2.25	1.08	5.39	0.21	8.17	3.66	0.71	0.23	0.65	0.12	0.55	0.15
Native	Mz	23	E	GPa	7.81	0.40	10.00	0.51	7.97	0.40	10.68	0.52	17.56	0.99	19.91	1.13	25.04	1.42	7.13	0.40	13.73	0.78	15.46	0.88
Native	Mz	23	Fe	%	0.00	0.00	0.00	0.00	0.00	0.00	0.00	0.00	0.21	0.16	2.99	0.54	16.00	1.55	0.00	0.00	0.00	0.00	0.00	0.00
Native	Mz	23	H	GPa	0.94	0.05	1.20	0.06	0.98	0.07	1.26	0.09	2.28	0.16	2.59	0.18	3.25	0.22	0.93	0.06	1.65	0.09	2.01	0.14
Native	Mz	23	Te	%	0.16	0.14	0.25	0.09	0.20	0.22	0.16	0.18	0.09	0.10	0.86	2.11	0.56	0.98	0.34	0.46	0.00	0.00	0.11	0.17
Native	Mz	24	Ae	%	0.84	0.22	0.51	0.08	0.71	1.29	0.03	0.06	3.06	1.14	7.31	0.95	26.86	1.21	0.00	0.00	0.91	0.34	0.08	0.16
Native	Mz	24	Ca	%	0.71	0.16	0.51	0.08	0.71	1.29	0.00	0.00	2.16	1.44	3.54	1.31	8.69	0.90	0.00	0.00	0.91	0.34	0.00	0.00
Native	Mz	24	E	GPa	8.96	0.45	10.92	0.55	8.71	0.44	11.47	0.79	17.60	1.00	19.99	1.13	26.31	1.49	7.84	0.44	15.26	0.86	16.31	0.92
Native	Mz	24	Fe	%	0.13	0.23	0.00	0.00	0.00	0.00	0.00	0.00	0.45	0.08	3.21	0.48	18.05	1.47	0.00	0.00	0.00	0.00	0.00	0.00
Native	Mz	24	H	GPa	1.07	0.05	1.31	0.07	1.08	0.07	1.38	0.10	2.29	0.16	2.60	0.18	3.42	0.24	1.02	0.07	1.83	0.10	2.12	0.15
Native	Mz	24	Te	%	0.00	0.00	0.00	0.00	0.00	0.00	0.03	0.06	0.45	0.42	0.55	0.88	0.12	0.25	0.00	0.00	0.00	0.00	0.08	0.16
Native	Mz	25	Ae	%	0.04	0.07	0.47	0.29	0.14	0.34	0.00	0.00	4.54	1.66	8.70	1.47	30.58	2.20	0.00	0.00	1.16	1.34	0.00	0.00
Native	Mz	25	Ca	%	0.00	0.00	0.20	0.34	0.14	0.34	0.00	0.00	2.99	2.34	4.34	1.29	10.01	1.76	0.00	0.00	1.16	1.34	0.00	0.00
Native	Mz	25	E	GPa	8.91	0.45	11.65	0.59	9.09	0.46	11.65	0.81	17.79	1.01	20.85	1.18	27.28	1.54	7.84	0.44	14.65	0.83	17.12	0.97
Native	Mz	25	Fe	%	0.00	0.00	0.00	0.00	0.00	0.00	0.00	0.00	0.60	0.07	4.26	0.62	19.47	1.80	0.00	0.00	0.00	0.00	0.00	0.00
Native	Mz	25	H	GPa	1.07	0.05	1.40	0.07	1.12	0.08	1.40	0.10	2.31	0.16	2.71	0.19	3.54	0.25	1.02	0.07	1.76	0.10	2.22	0.15

Native	Mz	25	Te	%	0.04	0.07	0.27	0.15	0.00	0.00	0.00	0.00	0.95	0.83	0.10	0.25	1.10	1.41	0.00	0.00	0.00	0.00	0.00	0.00
Native	Mz	26	Ae	%	2.66	3.50	2.33	2.95	2.01	2.09	1.01	0.28	2.72	2.57	10.79	1.46	35.79	1.17	0.00	0.00	0.65	0.14	0.58	0.11
Native	Mz	26	Ca	%	2.62	3.53	2.33	2.95	2.01	2.09	1.01	0.28	2.20	1.31	6.09	1.30	10.39	1.59	0.00	0.00	0.65	0.14	0.58	0.11
Native	Mz	26	E	GPa	10.00	0.51	11.67	0.59	10.20	0.51	11.88	0.64	17.77	1.01	21.71	1.23	28.46	1.61	6.78	0.38	15.26	0.86	17.37	0.98
Native	Mz	26	Fe	%	0.04	0.08	0.00	0.00	0.00	0.00	0.00	0.00	0.06	0.05	4.49	0.73	24.61	2.21	0.00	0.00	0.00	0.00	0.00	0.00
Native	Mz	26	H	GPa	1.20	0.06	1.40	0.07	1.26	0.09	1.43	0.08	2.31	0.16	2.82	0.19	3.70	0.26	0.88	0.06	1.83	0.10	2.26	0.16
Native	Mz	26	Te	%	0.00	0.00	0.00	0.00	0.00	0.00	0.00	0.00	0.46	0.89	0.21	0.51	0.78	0.72	0.00	0.00	0.00	0.00	0.00	0.00
Native	Mz	27	Ae	%	0.00	0.00	0.46	0.40	0.97	1.23	0.00	0.00	2.82	1.79	9.62	1.56	33.81	4.14	0.64	1.11	0.11	0.14	0.00	0.00
Native	Mz	27	Ca	%	0.00	0.00	0.46	0.40	0.97	1.23	0.00	0.00	2.05	1.28	4.36	1.28	7.53	2.29	0.16	0.25	0.11	0.14	0.00	0.00
Native	Mz	27	E	GPa	9.45	0.48	12.19	0.62	9.64	0.48	12.80	0.88	17.81	1.01	19.86	1.12	28.73	1.63	7.39	0.42	15.02	0.70	17.37	0.98
Native	Mz	27	Fe	%	0.00	0.00	0.00	0.00	0.00	0.00	0.00	0.00	0.37	0.12	5.23	0.42	25.85	2.69	0.00	0.00	0.00	0.00	0.00	0.00
Native	Mz	27	H	GPa	1.13	0.06	1.46	0.07	1.19	0.08	1.54	0.11	2.31	0.16	2.58	0.18	3.73	0.26	0.96	0.07	1.80	0.08	2.26	0.16
Native	Mz	27	Te	%	0.00	0.00	0.00	0.00	0.00	0.00	0.00	0.00	0.40	0.48	0.04	0.06	0.43	0.82	0.47	1.16	0.00	0.00	0.00	0.00
Native	Wz	28	Ae	%	0.20	0.29	0.15	0.26	0.82	1.30	0.22	0.35	5.60	1.27	12.29	1.45	33.08	3.87	0.00	0.00	0.65	0.54	0.41	0.32
Native	Wz	28	Ca	%	0.20	0.29	0.15	0.26	0.82	1.30	0.21	0.33	2.96	0.07	4.64	1.61	7.64	1.26	0.00	0.00	0.65	0.54	0.41	0.32
Native	Wz	28	E	GPa	10.54	0.75	13.28	0.67	10.20	0.51	13.95	0.96	17.81	1.01	19.75	1.22	31.93	1.81	6.78	0.38	15.26	0.86	16.31	0.92
Native	Wz	28	Fe	%	0.00	0.00	0.00	0.00	0.00	0.00	0.00	0.00	0.36	0.16	6.29	0.56	25.07	3.46	0.00	0.00	0.00	0.00	0.00	0.00
Native	Wz	28	H	GPa	1.26	0.09	1.59	0.08	1.26	0.09	1.67	0.12	2.31	0.16	2.52	0.18	4.15	0.29	0.88	0.06	1.83	0.10	2.12	0.15
Native	Wz	28	Te	%	0.00	0.00	0.00	0.00	0.00	0.00	0.01	0.03	2.29	1.77	1.35	0.61	0.37	0.58	0.00	0.00	0.00	0.00	0.00	0.00
Native	Wz	29	Ae	%	0.00	0.00	0.00	0.00	0.33	0.81	0.00	0.00	4.18	1.74	11.65	1.64	36.30	4.24	0.00	0.00	0.78	0.14	0.55	0.11
Native	Wz	29	Ca	%	0.00	0.00	0.00	0.00	0.33	0.81	0.00	0.00	3.15	0.28	4.55	1.32	6.50	0.98	0.00	0.00	0.78	0.14	0.55	0.11
Native	Wz	29	E	GPa	11.10	0.56	15.21	0.77	11.32	0.57	15.09	1.04	17.43	0.99	19.76	1.12	31.79	1.80	7.14	0.40	15.26	0.86	17.12	0.97
Native	Wz	29	Fe	%	0.00	0.00	0.00	0.00	0.00	0.00	0.00	0.00	0.49	0.13	6.50	0.46	28.54	4.47	0.00	0.00	0.00	0.00	0.00	0.00
Native	Wz	29	H	GPa	1.33	0.07	1.83	0.09	1.40	0.10	1.81	0.13	2.26	0.16	2.57	0.18	4.13	0.29	0.93	0.06	1.83	0.10	2.22	0.15
Native	Wz	29	Te	%	0.00	0.00	0.00	0.00	0.00	0.00	0.00	0.00	0.55	0.97	0.60	0.48	1.25	1.50	0.00	0.00	0.00	0.00	0.00	0.00
Native	Wz	30	Ae	%	0.87	0.20	0.95	0.06	0.82	1.30	0.00	0.00	5.68	1.15	12.43	0.58	36.37	2.16	0.81	0.07	0.34	0.83	1.26	0.54
Native	Wz	30	Ca	%	0.87	0.20	0.95	0.06	0.82	1.30	0.00	0.00	4.00	1.47	5.60	0.35	7.17	1.13	0.81	0.07	0.34	0.83	1.26	0.54
Native	Wz	30	E	GPa	12.30	0.62	16.40	0.83	11.83	0.59	16.24	1.12	16.98	0.96	20.42	1.16	30.73	1.74	7.55	0.43	16.33	0.92	17.37	0.98
Native	Wz	30	Fe	%	0.00	0.00	0.00	0.00	0.00	0.00	0.00	0.00	0.41	0.23	6.51	0.50	28.71	2.01	0.00	0.00	0.00	0.00	0.00	0.00
Native	Wz	30	H	GPa	1.48	0.07	1.97	0.10	1.46	0.10	1.95	0.13	2.21	0.15	2.65	0.18	3.99	0.28	0.98	0.07	1.96	0.11	2.26	0.16
Native	Wz	30	Te	%	0.00	0.00	0.00	0.00	0.00	0.00	0.00	0.00	1.27	1.24	0.32	0.60	0.48	0.54	0.00	0.00	0.00	0.00	0.00	0.00
Native	Wz	31	Ae	%	0.37	0.65	0.00	0.00	1.02	1.57	0.00	0.00	4.80	2.05	11.70	1.53	31.27	2.10	0.71	1.70	2.24	1.26	0.00	0.00
Native	Wz	31	Ca	%	0.00	0.00	0.00	0.00	1.02	1.57	0.00	0.00	2.98	0.16	4.70	1.25	6.13	1.82	0.00	0.00	2.24	1.26	0.00	0.00
Native	Wz	31	E	GPa	11.80	0.60	16.43	0.83	11.33	0.57	16.25	1.12	16.51	0.93	18.43	1.04	31.16	1.76	8.13	0.46	15.46	0.87	17.37	0.98
Native	Wz	31	Fe	%	0.37	0.65	0.00	0.00	0.00	0.00	0.00	0.00	0.60	0.07	6.42	0.54	24.72	2.76	0.00	0.00	0.00	0.00	0.00	0.00
Native	Wz	31	H	GPa	1.42	0.07	1.97	0.10	1.40	0.10	1.95	0.13	2.14	0.15	2.39	0.17	4.05	0.28	1.06	0.07	1.85	0.10	2.26	0.16
Native	Wz	31	Te	%	0.00	0.00	0.00	0.00	0.00	0.00	0.00	0.00	1.21	1.86	0.58	0.64	0.42	0.93	0.71	1.70	0.00	0.00	0.00	0.00
Native	Wz	32	Ae	%	0.33	0.58	0.88	0.15	1.09	1.20	0.00	0.00	3.65	1.81	11.99	1.43	33.06	3.84	0.31	0.49	1.40	0.46	0.98	0.17
Native	Wz	32	Ca	%	0.33	0.58	0.88	0.15	1.09	1.20	0.00	0.00	2.96	0.19	4.61	1.42	5.19	1.41	0.31	0.49	1.40	0.46	0.98	0.17
Native	Wz	32	E	GPa	12.20	0.62	16.57	0.84	12.44	0.62	17.40	1.20	17.63	1.00	19.79	1.12	31.79	1.80	7.39	0.42	15.26	0.86	17.47	0.99
Native	Wz	32	Fe	%	0.00	0.00	0.00	0.00	0.00	0.00	0.00	0.00	0.54	0.17	6.36	0.92	26.66	3.07	0.00	0.00	0.00	0.00	0.00	0.00
Native	Wz	32	H	GPa	1.46	0.07	1.99	0.10	1.54	0.11	2.09	0.14	2.29	0.16	2.57	0.18	4.13	0.29	0.96	0.07	1.83	0.10	2.27	0.16
Native	Wz	32	Te	%	0.00	0.00	0.00	0.00	0.00	0.00	0.00	0.00	0.16	0.19	1.02	1.35	1.20	1.87	0.00	0.00	0.00	0.00	0.00	0.00
Native	Wz	33	Ae	%	0.00	0.00	1.66	2.88	0.65	1.01	0.00	0.00	4.05	0.20	11.39	1.29	32.15	3.27	0.00	0.00	1.19	0.92	0.60	0.51
Native	Wz	33	Ca	%	0.00	0.00	1.66	2.88	0.65	1.01	0.00	0.00	3.02	0.13	4.61	1.34	5.84	0.84	0.00	0.00	1.19	0.92	0.60	0.51
Native	Wz	33	E	GPa	12.74	0.65	17.21	0.87	13.00	0.65	17.34	0.96	16.78	1.14	19.33	1.30	29.33	2.05	6.78	0.38	15.26	0.86	16.99	0.96
Native	Wz	33	Fe	%	0.00	0.00	0.00	0.00	0.00	0.00	0.00	0.00	0.71	0.28	6.74	0.89	26.03	3.27	0.00	0.00	0.00	0.00	0.00	0.00
Native	Wz	33	H	GPa	1.53	0.08	2.07	0.10	1.61	0.11	2.08	0.12	2.13	0.15	2.46	0.17	3.72	0.26	0.88	0.06	1.83	0.10	2.21	0.15
Native	Wz	33	Te	%	0.00	0.00	0.00	0.00	0.00	0.00	0.00	0.00	0.32	0.49	0.03	0.08	0.28	0.37	0.00	0.00	0.00	0.00	0.00	0.00
Native	Wz	34	Ae	%	1.14	0.81	1.02	0.60	1.20	1.36	0.00	0.00	3.73	0.66	10.03	1.76	34.21	1.88	0.00	0.00	0.60	0.45	0.00	0.00
Native	Wz	34	Ca	%	0.97	0.52	1.02	0.60	1.20	1.36	0.00	0.00	3.04	0.14	2.91	0.16	4.86	1.75	0.00	0.00	0.60	0.45	0.00	0.00
Native	Wz	34	E	GPa	12.20	0.62	17.13	0.87	12.45	0.62	17.22	0.95	17.70	1.00	19.81	1.12	31.71	1.79	7.14	0.40	15.33	0.94	17.20	0.97
Native	Wz	34	Fe	%	0.15	0.26	0.00	0.00	0.00	0.00	0.00	0.00	0.24	0.14	6.39	0.93	26.97	1.88	0.00	0.00	0.00	0.00	0.00	0.00
Native	Wz	34	H	GPa	1.46	0.07	2.06	0.10	1.54	0.11	2.07	0.11	2.30	0.16	2.57	0.18	4.12	0.28	0.93	0.06	1.84	0.11	2.06	0.12

Native	Wz	34	Te	%	0.02	0.03	0.00	0.00	0.00	0.00	0.00	0.00	0.46	1.12	0.74	1.30	2.38	1.09	0.00	0.00	0.00	0.00	0.00	0.00
Native	Wz	35	Ae	%	2.68	3.66	0.52	0.90	1.75	1.20	2.97	1.55	4.04	1.03	10.67	0.69	33.26	2.83	1.19	1.84	0.84	2.06	0.55	0.43
Native	Wz	35	Ca	%	2.68	3.66	0.52	0.90	1.75	1.20	2.97	1.55	3.83	1.20	3.03	0.30	4.79	0.29	1.19	1.84	0.84	2.06	0.55	0.43
Native	Wz	35	E	GPa	12.20	0.62	17.78	0.59	12.47	0.58	17.29	0.83	17.21	0.97	19.46	1.31	29.58	2.06	7.55	0.43	15.26	0.86	17.12	0.97
Native	Wz	35	Fe	%	0.00	0.00	0.00	0.00	0.00	0.00	0.00	0.00	0.09	0.07	7.51	0.73	26.65	3.07	0.00	0.00	0.00	0.00	0.00	0.00
Native	Wz	35	H	GPa	1.46	0.07	2.05	0.10	1.55	0.11	2.05	0.11	2.24	0.15	2.47	0.17	3.75	0.26	0.98	0.07	1.83	0.10	2.05	0.12
Native	Wz	35	Te	%	0.00	0.00	0.00	0.00	0.00	0.00	0.00	0.00	0.12	0.29	0.14	0.22	1.82	1.68	0.00	0.00	0.00	0.00	0.00	0.00
Native	Wz	36	Ae	%	2.56	1.15	1.38	1.20	1.31	1.22	1.47	2.28	4.54	1.08	8.62	2.83	34.27	2.79	0.00	0.00	1.41	1.61	0.00	0.00
Native	Wz	36	Ca	%	2.01	0.32	1.38	1.20	1.31	1.22	1.45	2.25	3.81	1.44	3.70	1.21	5.44	1.28	0.00	0.00	1.41	1.61	0.00	0.00
Native	Wz	36	E	GPa	12.11	0.61	17.32	0.47	12.35	0.62	17.01	0.56	16.88	0.96	19.54	1.11	32.06	1.81	8.31	0.34	15.26	0.86	16.06	0.91
Native	Wz	36	Fe	%	0.55	0.95	0.00	0.00	0.00	0.00	0.00	0.00	0.10	0.08	4.62	1.63	27.29	2.29	0.00	0.00	0.00	0.00	0.00	0.00
Native	Wz	36	H	GPa	1.45	0.07	1.86	0.09	1.53	0.11	2.00	0.11	2.19	0.15	2.54	0.18	4.16	0.29	1.06	0.07	1.83	0.10	1.93	0.11
Native	Wz	36	Te	%	0.00	0.00	0.00	0.00	0.00	0.00	0.02	0.04	0.63	1.02	0.31	0.47	1.54	1.91	0.00	0.00	0.00	0.00	0.00	0.00
Native	Wz	37	Ae	%	1.02	0.87	1.49	1.36	1.69	1.99	0.00	0.00	3.94	2.15	10.59	0.92	34.66	4.38	0.04	0.10	2.44	2.79	0.00	0.00
Native	Wz	37	Ca	%	0.64	1.12	1.49	1.36	1.69	1.99	0.00	0.00	3.76	1.32	4.14	1.45	5.69	0.87	0.00	0.00	2.44	2.79	0.00	0.00
Native	Wz	37	E	GPa	13.41	0.68	16.72	0.85	12.73	0.64	17.09	0.56	16.78	0.95	20.42	1.16	30.13	1.56	7.60	0.33	15.26	0.86	16.31	0.92
Native	Wz	37	Fe	%	0.00	0.00	0.00	0.00	0.00	0.00	0.00	0.00	0.13	0.02	6.38	0.84	26.55	1.60	0.00	0.00	0.00	0.00	0.00	0.00
Native	Wz	37	H	GPa	1.61	0.08	2.01	0.10	1.57	0.11	2.01	0.11	2.18	0.15	2.65	0.18	3.75	0.29	0.87	0.06	1.83	0.10	1.96	0.11
Native	Wz	37	Te	%	0.37	0.48	0.00	0.00	0.00	0.00	0.00	0.00	0.06	0.10	0.07	0.17	2.42	3.11	0.04	0.10	0.00	0.00	0.00	0.00
Native	Wz	38	Ae	%	0.93	0.10	0.61	0.87	1.05	1.16	0.00	0.00	4.51	1.39	9.98	1.64	35.59	6.32	0.00	0.00	1.18	1.82	0.00	0.00
Native	Wz	38	Ca	%	0.93	0.10	0.61	0.87	1.05	1.16	0.00	0.00	4.42	1.31	4.07	1.16	6.08	1.29	0.00	0.00	1.18	1.82	0.00	0.00
Native	Wz	38	E	GPa	12.56	0.64	17.45	1.25	12.81	0.64	17.47	0.97	16.78	0.95	20.72	1.17	29.72	2.07	7.55	0.43	15.26	0.86	16.89	0.96
Native	Wz	38	Fe	%	0.00	0.00	0.00	0.00	0.00	0.00	0.00	0.00	0.09	0.07	5.84	0.57	25.80	1.86	0.00	0.00	0.00	0.00	0.00	0.00
Native	Wz	38	H	GPa	1.51	0.08	2.09	0.15	1.58	0.11	2.10	0.12	2.18	0.15	2.69	0.19	3.77	0.26	0.98	0.07	1.83	0.10	2.03	0.11
Native	Wz	38	Te	%	0.00	0.00	0.00	0.00	0.00	0.00	0.00	0.00	0.00	0.00	0.06	0.15	3.71	4.91	0.00	0.00	0.00	0.00	0.00	0.00
Native	Wz	39	Ae	%	1.22	2.10	1.97	1.72	2.06	1.47	1.58	2.11	4.51	1.28	11.55	1.97	39.52	2.90	1.95	1.17	1.95	1.55	0.00	0.00
Native	Wz	39	Ca	%	1.22	2.10	1.97	1.72	2.06	1.47	1.58	2.11	4.38	1.30	4.82	0.90	6.04	1.08	1.89	1.16	1.77	1.77	0.00	0.00
Native	Wz	39	E	GPa	12.67	0.64	17.18	0.87	13.11	0.51	16.79	0.63	18.12	1.03	21.31	1.21	30.69	2.12	8.27	0.47	15.33	0.94	16.31	0.92
Native	Wz	39	Fe	%	0.00	0.00	0.00	0.00	0.00	0.00	0.00	0.00	0.12	0.10	6.60	1.17	30.23	2.21	0.00	0.00	0.18	0.25	0.00	0.00
Native	Wz	39	H	GPa	1.52	0.08	2.06	0.10	1.63	0.09	2.05	0.11	2.35	0.16	2.77	0.19	3.89	0.27	1.07	0.07	1.84	0.11	1.96	0.11
Native	Wz	39	Te	%	0.00	0.00	0.00	0.00	0.00	0.00	0.00	0.00	0.00	0.00	0.13	0.20	3.25	3.80	0.06	0.15	0.00	0.00	0.00	0.00
Treated	Rs	1	Ae	%	0.00	0.00	0.00	0.00	0.00	0.00	0.00	0.00	0.00	0.00	0.00	0.00	0.00	0.00	0.00	0.00	0.00	0.00	0.41	0.63
Treated	Rs	1	Ca	%	0.00	0.00	0.00	0.00	0.00	0.00	0.00	0.00	0.00	0.00	0.00	0.00	0.00	0.00	0.00	0.00	0.00	0.00	0.00	0.00
Treated	Rs	1	E	GPa	1.75	0.09	1.92	0.10	2.26	0.17	2.61	0.32	2.57	0.13	2.05	0.38	2.31	0.13	2.26	0.28	2.48	0.46	2.54	0.34
Treated	Rs	1	Fe	%	0.00	0.00	0.00	0.00	0.00	0.00	0.00	0.00	0.00	0.00	0.00	0.00	0.00	0.00	0.00	0.00	0.00	0.00	0.41	0.63
Treated	Rs	1	H	GPa	0.21	0.01	0.23	0.01	0.28	0.02	0.33	0.05	0.27	0.02	0.27	0.04	0.32	0.02	0.30	0.04	0.32	0.06	0.24	0.05
Treated	Rs	1	Te	%	0.00	0.00	0.00	0.00	0.00	0.00	0.00	0.00	0.00	0.00	0.00	0.00	0.00	0.00	0.00	0.00	0.00	0.00	0.00	0.00
Treated	Rs	2	Ae	%	0.00	0.00	0.00	0.00	0.00	0.00	0.07	0.16	0.00	0.00	0.01	0.02	0.00	0.00	0.00	0.00	0.00	0.00	0.00	0.00
Treated	Rs	2	Ca	%	0.00	0.00	0.00	0.00	0.00	0.00	0.00	0.00	0.00	0.00	0.00	0.00	0.00	0.00	0.00	0.00	0.00	0.00	0.00	0.00
Treated	Rs	2	E	GPa	1.82	0.12	1.96	0.10	2.30	0.20	2.49	0.37	2.71	0.14	1.98	0.11	2.69	0.50	2.43	0.16	2.67	0.18	2.47	0.25
Treated	Rs	2	Fe	%	0.00	0.00	0.00	0.00	0.00	0.00	0.07	0.16	0.00	0.00	0.01	0.02	0.00	0.00	0.00	0.00	0.00	0.00	0.00	0.00
Treated	Rs	2	H	GPa	0.21	0.01	0.23	0.01	0.27	0.02	0.31	0.03	0.29	0.02	0.26	0.02	0.47	0.03	0.32	0.03	0.34	0.02	0.23	0.03
Treated	Rs	2	Te	%	0.00	0.00	0.00	0.00	0.00	0.00	0.00	0.00	0.00	0.00	0.00	0.00	0.00	0.00	0.00	0.00	0.00	0.00	0.00	0.00
Treated	Rs	3	Ae	%	0.00	0.00	0.00	0.00	0.00	0.00	0.00	0.00	0.00	0.00	0.00	0.00	0.00	0.00	0.00	0.00	0.28	0.44	0.00	0.00
Treated	Rs	3	Ca	%	0.00	0.00	0.00	0.00	0.00	0.00	0.00	0.00	0.00	0.00	0.00	0.00	0.00	0.00	0.00	0.00	0.00	0.00	0.00	0.00
Treated	Rs	3	E	GPa	1.87	0.16	2.00	0.13	2.27	0.13	2.36	0.19	3.80	0.19	2.02	0.11	4.00	0.89	2.60	0.34	2.83	0.23	2.70	0.57
Treated	Rs	3	Fe	%	0.00	0.00	0.00	0.00	0.00	0.00	0.00	0.00	0.00	0.00	0.00	0.00	0.00	0.00	0.00	0.00	0.28	0.44	0.00	0.00
Treated	Rs	3	H	GPa	0.22	0.02	0.24	0.02	0.25	0.02	0.32	0.05	0.40	0.03	0.26	0.02	0.53	0.08	0.34	0.05	0.36	0.03	0.26	0.08
Treated	Rs	3	Te	%	0.00	0.00	0.00	0.00	0.00	0.00	0.00	0.00	0.00	0.00	0.00	0.00	0.00	0.00	0.00	0.00	0.00	0.00	0.00	0.00
Treated	Rs	4	Ae	%	0.48	0.52	0.20	0.28	0.00	0.00	0.00	0.00	0.00	0.00	0.00	0.00	0.03	0.05	0.00	0.00	0.00	0.00	0.00	0.00
Treated	Rs	4	Ca	%	0.00	0.00	0.00	0.00	0.00	0.00	0.00	0.00	0.00	0.00	0.00	0.00	0.03	0.05	0.00	0.00	0.00	0.00	0.00	0.00
Treated	Rs	4	E	GPa	1.88	0.15	2.16	0.13	2.30	0.15	2.45	0.35	3.72	0.19	2.09	0.12	3.68	0.20	3.07	0.49	2.96	0.68	2.65	0.58
Treated	Rs	4	Fe	%	0.13	0.23	0.20	0.28	0.00	0.00	0.00	0.00	0.00	0.00	0.00	0.00	0.00	0.00	0.00	0.00	0.00	0.00	0.00	0.00
Treated	Rs	4	H	GPa	0.22	0.02	0.26	0.02	0.26	0.02	0.32	0.03	0.39	0.03	0.27	0.02	0.51	0.04	0.40	0.07	0.38	0.09	0.25	0.08

Treated	Rs	4	Te	%	0.35	0.60	0.00	0.00	0.00	0.00	0.00	0.00	0.00	0.00	0.00	0.00	0.00	0.00	0.00	0.00	0.00	0.00	0.00	0.00
Treated	Rs	5	Ae	%	0.00	0.00	0.00	0.00	0.00	0.00	0.05	0.12	0.00	0.00	0.01	0.03	0.10	0.08	0.00	0.00	0.30	0.43	0.00	0.00
Treated	Rs	5	Ca	%	0.00	0.00	0.00	0.00	0.00	0.00	0.00	0.00	0.00	0.00	0.00	0.00	0.08	0.09	0.00	0.00	0.00	0.00	0.00	0.00
Treated	Rs	5	E	GPa	1.79	0.09	2.06	0.19	2.34	0.18	2.45	0.26	4.04	0.20	1.36	0.08	4.71	0.26	3.05	0.60	2.84	0.83	2.72	0.54
Treated	Rs	5	Fe	%	0.00	0.00	0.00	0.00	0.00	0.00	0.05	0.12	0.00	0.00	0.00	0.00	0.00	0.00	0.00	0.00	0.28	0.44	0.00	0.00
Treated	Rs	5	H	GPa	0.21	0.01	0.25	0.02	0.26	0.02	0.34	0.06	0.43	0.03	0.18	0.01	0.65	0.05	0.40	0.08	0.37	0.11	0.26	0.07
Treated	Rs	5	Te	%	0.00	0.00	0.00	0.00	0.00	0.00	0.00	0.00	0.00	0.00	0.01	0.03	0.02	0.05	0.00	0.00	0.02	0.04	0.00	0.00
Treated	Rs	6	Ae	%	0.00	0.00	0.01	0.02	0.00	0.00	0.00	0.00	0.00	0.00	0.07	0.16	0.71	0.77	0.03	0.07	0.04	0.11	0.00	0.00
Treated	Rs	6	Ca	%	0.00	0.00	0.01	0.02	0.00	0.00	0.00	0.00	0.00	0.00	0.07	0.16	0.31	0.34	0.03	0.07	0.04	0.11	0.00	0.00
Treated	Rs	6	E	GPa	1.84	0.09	1.99	0.10	2.28	0.12	2.59	0.42	5.03	0.25	1.82	0.10	6.82	0.38	3.52	0.99	2.84	0.82	3.13	0.84
Treated	Rs	6	Fe	%	0.00	0.00	0.00	0.00	0.00	0.00	0.00	0.00	0.00	0.00	0.00	0.00	0.40	0.63	0.00	0.00	0.00	0.00	0.00	0.00
Treated	Rs	6	H	GPa	0.22	0.01	0.24	0.01	0.26	0.02	0.32	0.03	0.53	0.04	0.24	0.02	0.94	0.07	0.46	0.13	0.36	0.11	0.31	0.11
Treated	Rs	6	Te	%	0.00	0.00	0.00	0.00	0.00	0.00	0.00	0.00	0.00	0.00	0.00	0.00	0.00	0.00	0.00	0.00	0.00	0.00	0.00	0.00
Treated	Rs	7	Ae	%	0.00	0.00	0.09	0.15	0.00	0.00	0.00	0.00	0.13	0.12	0.25	0.12	0.69	0.36	0.00	0.00	0.00	0.00	0.00	0.00
Treated	Rs	7	Ca	%	0.00	0.00	0.09	0.15	0.00	0.00	0.00	0.00	0.00	0.00	0.09	0.11	0.14	0.34	0.00	0.00	0.00	0.00	0.00	0.00
Treated	Rs	7	E	GPa	1.86	0.12	2.07	0.14	2.34	0.15	2.43	0.21	6.10	0.31	2.02	0.11	7.94	0.44	3.29	1.28	3.51	1.20	3.42	0.79
Treated	Rs	7	Fe	%	0.00	0.00	0.00	0.00	0.00	0.00	0.00	0.00	0.13	0.12	0.16	0.05	0.55	0.41	0.00	0.00	0.00	0.00	0.00	0.00
Treated	Rs	7	H	GPa	0.22	0.01	0.25	0.02	0.26	0.02	0.32	0.04	0.64	0.05	0.26	0.02	0.97	0.07	0.43	0.17	0.45	0.15	0.35	0.11
Treated	Rs	7	Te	%	0.00	0.00	0.00	0.00	0.00	0.00	0.00	0.00	0.00	0.00	0.00	0.00	0.00	0.00	0.00	0.00	0.00	0.00	0.00	0.00
Treated	Rs	8	Ae	%	0.00	0.00	0.09	0.16	0.13	0.33	0.00	0.00	0.36	0.42	0.32	0.16	2.41	0.48	0.00	0.00	0.00	0.00	0.00	0.00
Treated	Rs	8	Ca	%	0.00	0.00	0.09	0.16	0.00	0.00	0.00	0.00	0.16	0.40	0.05	0.08	0.00	0.00	0.00	0.00	0.00	0.00	0.00	0.00
Treated	Rs	8	E	GPa	1.94	0.18	2.08	0.17	2.37	0.17	2.47	0.27	7.05	0.36	2.52	0.14	8.62	0.48	3.69	1.77	3.52	0.80	3.52	1.06
Treated	Rs	8	Fe	%	0.00	0.00	0.00	0.00	0.13	0.33	0.00	0.00	0.20	0.31	0.27	0.09	2.41	0.48	0.00	0.00	0.00	0.00	0.00	0.00
Treated	Rs	8	H	GPa	0.23	0.02	0.25	0.02	0.27	0.02	0.32	0.03	0.75	0.05	0.33	0.02	1.06	0.07	0.48	0.23	0.45	0.10	0.45	0.12
Treated	Rs	8	Te	%	0.00	0.00	0.00	0.00	0.00	0.00	0.00	0.00	0.00	0.00	0.00	0.00	0.00	0.00	0.00	0.00	0.00	0.00	0.00	0.00
Treated	Rs	9	Ae	%	0.00	0.00	0.00	0.00	0.00	0.00	0.02	0.04	0.43	0.47	0.17	0.04	5.05	0.64	0.00	0.00	0.03	0.07	0.00	0.00
Treated	Rs	9	Ca	%	0.00	0.00	0.00	0.00	0.00	0.00	0.00	0.00	0.30	0.41	0.00	0.00	0.00	0.00	0.00	0.00	0.03	0.07	0.00	0.00
Treated	Rs	9	E	GPa	1.96	0.18	2.09	0.17	2.43	0.22	2.53	0.28	7.60	0.97	2.68	0.15	10.02	0.55	3.98	1.54	3.93	0.95	3.86	0.69
Treated	Rs	9	Fe	%	0.00	0.00	0.00	0.00	0.00	0.00	0.00	0.00	0.13	0.09	0.17	0.04	5.05	0.64	0.00	0.00	0.00	0.00	0.00	0.00
Treated	Rs	9	H	GPa	0.23	0.02	0.25	0.02	0.27	0.03	0.31	0.05	0.87	0.05	0.35	0.02	1.23	0.09	0.52	0.20	0.51	0.12	0.51	0.09
Treated	Rs	9	Te	%	0.00	0.00	0.00	0.00	0.00	0.00	0.02	0.04	0.00	0.00	0.00	0.00	0.00	0.00	0.00	0.00	0.00	0.00	0.00	0.00
Treated	Rs	10	Ae	%	0.00	0.00	0.00	0.00	0.00	0.00	0.07	0.16	0.09	0.14	0.18	0.06	5.48	1.31	0.00	0.00	0.00	0.00	0.00	0.00
Treated	Rs	10	Ca	%	0.00	0.00	0.00	0.00	0.00	0.00	0.00	0.00	0.00	0.00	0.00	0.00	0.00	0.00	0.00	0.00	0.00	0.00	0.00	0.00
Treated	Rs	10	E	GPa	1.87	0.10	2.00	0.11	2.36	0.13	2.46	0.41	6.11	0.68	3.34	0.18	10.54	0.58	3.81	1.35	4.23	0.77	4.51	0.60
Treated	Rs	10	Fe	%	0.00	0.00	0.00	0.00	0.00	0.00	0.07	0.16	0.09	0.14	0.18	0.06	5.48	1.31	0.00	0.00	0.00	0.00	0.00	0.00
Treated	Rs	10	H	GPa	0.22	0.01	0.24	0.01	0.26	0.02	0.24	0.02	0.96	0.07	0.44	0.03	1.29	0.09	0.50	0.16	0.54	0.10	0.59	0.08
Treated	Rs	10	Te	%	0.00	0.00	0.00	0.00	0.00	0.00	0.00	0.00	0.00	0.00	0.00	0.00	0.00	0.00	0.00	0.00	0.00	0.00	0.00	0.00
Treated	Rs	11	Ae	%	0.11	0.18	0.00	0.00	0.10	0.25	0.00	0.00	0.45	0.47	0.60	0.09	6.19	1.43	0.00	0.00	0.00	0.00	0.00	0.00
Treated	Rs	11	Ca	%	0.11	0.18	0.00	0.00	0.00	0.00	0.00	0.00	0.13	0.32	0.00	0.00	0.00	0.00	0.00	0.00	0.00	0.00	0.00	0.00
Treated	Rs	11	E	GPa	1.89	0.12	2.07	0.10	2.42	0.17	1.96	0.19	6.11	0.41	3.86	0.21	11.69	0.65	2.81	0.76	4.35	0.60	5.31	0.52
Treated	Rs	11	Fe	%	0.00	0.00	0.00	0.00	0.10	0.25	0.00	0.00	0.29	0.24	0.60	0.09	6.19	1.43	0.00	0.00	0.00	0.00	0.00	0.00
Treated	Rs	11	H	GPa	0.22	0.01	0.25	0.01	0.27	0.02	0.26	0.04	1.03	0.07	0.51	0.04	1.43	0.10	0.37	0.10	0.56	0.08	0.69	0.09
Treated	Rs	11	Te	%	0.00	0.00	0.00	0.00	0.00	0.00	0.00	0.00	0.03	0.07	0.00	0.00	0.00	0.00	0.00	0.00	0.00	0.00	0.00	0.00
Treated	Rs	12	Ae	%	0.12	0.13	0.00	0.00	0.00	0.00	0.06	0.14	0.57	0.53	0.55	0.18	7.48	1.80	0.00	0.00	0.07	0.18	0.00	0.00
Treated	Rs	12	Ca	%	0.09	0.15	0.00	0.00	0.00	0.00	0.00	0.00	0.48	0.53	0.00	0.00	0.00	0.00	0.00	0.00	0.07	0.18	0.00	0.00
Treated	Rs	12	E	GPa	1.96	0.14	2.09	0.14	2.45	0.18	2.10	0.32	5.71	0.99	4.03	0.22	12.01	0.67	2.97	0.70	4.45	0.40	5.94	0.60
Treated	Rs	12	Fe	%	0.00	0.00	0.00	0.00	0.00	0.00	0.06	0.14	0.10	0.15	0.55	0.18	7.48	1.80	0.00	0.00	0.00	0.00	0.00	0.00
Treated	Rs	12	H	GPa	0.23	0.02	0.25	0.02	0.27	0.02	0.25	0.02	1.08	0.08	0.53	0.04	1.47	0.10	0.39	0.09	0.57	0.05	0.78	0.09
Treated	Rs	12	Te	%	0.03	0.06	0.00	0.00	0.00	0.00	0.00	0.00	0.00	0.00	0.00	0.00	0.00	0.00	0.00	0.00	0.00	0.00	0.00	0.00
Treated	Mz	13	Ae	%	0.16	0.28	0.04	0.08	0.00	0.00	0.00	0.00	0.03	0.05	0.15	0.04	9.61	1.82	0.00	0.00	0.11	0.26	0.25	0.28
Treated	Mz	13	Ca	%	0.16	0.28	0.04	0.08	0.00	0.00	0.00	0.00	0.00	0.00	0.00	0.00	0.34	0.59	0.00	0.00	0.11	0.26	0.25	0.28
Treated	Mz	13	E	GPa	1.94	0.16	2.14	0.18	2.37	0.12	1.97	0.17	5.96	0.55	4.68	0.28	13.41	0.74	3.18	0.60	5.30	0.36	6.84	0.61
Treated	Mz	13	Fe	%	0.00	0.00	0.00	0.00	0.00	0.00	0.00	0.00	0.03	0.05	0.15	0.04	9.27	1.72	0.00	0.00	0.00	0.00	0.00	0.00
Treated	Mz	13	H	GPa	0.23	0.02	0.25	0.02	0.27	0.02	0.24	0.02	1.28	0.18	0.62	0.05	1.64	0.11	0.42	0.08	0.68	0.05	0.90	0.09

Treated	Mz	13	Te	%	0.00	0.00	0.00	0.00	0.00	0.04	0.00	0.00	0.00	0.00	0.00	0.00	0.00	0.00	0.00	0.00	0.00	0.00	0.00	0.00
Treated	Mz	14	Ae	%	0.00	0.00	0.00	0.00	0.02	0.04	0.00	0.00	0.04	0.06	0.58	0.14	10.15	0.66	0.00	0.00	0.01	0.03	0.00	0.00
Treated	Mz	14	Ca	%	0.00	0.00	0.00	0.00	0.00	0.00	0.00	0.00	0.00	0.00	0.00	0.00	0.60	0.73	0.00	0.00	0.00	0.00	0.00	0.00
Treated	Mz	14	E	GPa	1.88	0.09	2.20	0.16	2.42	0.13	1.92	0.13	6.18	0.56	5.71	0.32	14.51	0.80	3.18	0.63	6.14	0.35	7.59	0.62
Treated	Mz	14	Fe	%	0.00	0.00	0.00	0.00	0.00	0.00	0.00	0.00	0.04	0.06	0.56	0.16	9.55	1.22	0.00	0.00	0.00	0.00	0.00	0.00
Treated	Mz	14	H	GPa	0.22	0.01	0.26	0.02	0.27	0.02	0.24	0.02	1.61	0.11	0.75	0.05	1.78	0.12	0.42	0.08	0.79	0.04	1.02	0.11
Treated	Mz	14	Te	%	0.00	0.00	0.00	0.00	0.02	0.18	0.00	0.00	0.00	0.00	0.02	0.04	0.00	0.00	0.00	0.00	0.01	0.03	0.00	0.00
Treated	Mz	15	Ae	%	0.00	0.00	0.00	0.00	0.28	0.24	0.08	0.09	0.07	0.10	1.14	0.50	10.49	0.93	0.00	0.00	0.03	0.08	0.16	0.22
Treated	Mz	15	Ca	%	0.00	0.00	0.00	0.00	0.16	0.26	0.06	0.09	0.00	0.00	0.57	0.59	0.28	0.62	0.00	0.00	0.03	0.08	0.15	0.23
Treated	Mz	15	E	GPa	1.89	0.09	2.16	0.11	2.42	0.12	1.94	0.14	7.03	1.08	6.08	0.34	14.99	0.83	3.32	0.20	7.21	0.39	8.35	0.48
Treated	Mz	15	Fe	%	0.00	0.00	0.00	0.00	0.00	0.00	0.00	0.00	0.07	0.10	0.54	0.09	10.21	1.26	0.00	0.00	0.00	0.00	0.00	0.00
Treated	Mz	15	H	GPa	0.22	0.01	0.25	0.01	0.27	0.02	0.32	0.02	1.83	0.13	0.80	0.06	1.83	0.13	0.43	0.04	0.93	0.05	1.16	0.08
Treated	Mz	15	Te	%	0.00	0.00	0.00	0.00	0.12	0.00	0.02	0.04	0.00	0.00	0.02	0.06	0.00	0.00	0.00	0.00	0.00	0.00	0.02	0.04
Treated	Mz	16	Ae	%	0.07	0.12	0.00	0.00	0.00	0.00	0.00	0.00	0.44	0.30	2.26	0.44	13.37	3.14	0.09	0.22	0.14	0.23	0.03	0.07
Treated	Mz	16	Ca	%	0.00	0.00	0.00	0.00	0.00	0.00	0.00	0.00	0.00	0.00	1.39	0.39	0.00	0.00	0.09	0.22	0.14	0.23	0.03	0.07
Treated	Mz	16	E	GPa	2.58	0.13	2.86	0.14	3.18	0.17	2.57	0.18	6.28	1.09	7.00	0.39	16.66	0.92	3.52	0.19	7.64	0.41	8.86	0.55
Treated	Mz	16	Fe	%	0.07	0.12	0.00	0.00	0.00	0.00	0.00	0.00	0.44	0.30	0.86	0.24	13.37	3.14	0.00	0.00	0.00	0.00	0.00	0.00
Treated	Mz	16	H	GPa	0.30	0.02	0.34	0.02	0.35	0.03	0.43	0.03	1.86	0.14	0.92	0.06	2.04	0.14	0.46	0.03	0.98	0.05	1.23	0.09
Treated	Mz	16	Te	%	0.00	0.00	0.00	0.00	0.00	0.00	0.00	0.00	0.00	0.00	0.01	0.01	0.00	0.00	0.00	0.00	0.00	0.00	0.00	0.00
Treated	Mz	17	Ae	%	0.00	0.00	0.05	0.09	0.00	0.00	0.01	0.03	0.27	0.29	0.64	0.53	12.35	1.55	0.00	0.00	0.02	0.05	0.00	0.00
Treated	Mz	17	Ca	%	0.00	0.00	0.00	0.00	0.00	0.00	0.00	0.00	0.00	0.00	0.07	0.11	0.00	0.00	0.00	0.00	0.02	0.05	0.00	0.00
Treated	Mz	17	E	GPa	2.50	0.13	3.79	0.19	3.21	0.16	3.41	0.24	5.78	0.52	7.78	0.43	17.73	1.07	3.92	0.52	8.05	0.44	9.14	0.51
Treated	Mz	17	Fe	%	0.00	0.00	0.00	0.00	0.00	0.00	0.01	0.03	0.27	0.29	0.57	0.49	12.35	1.55	0.00	0.00	0.00	0.00	0.00	0.00
Treated	Mz	17	H	GPa	0.29	0.01	0.45	0.02	0.36	0.03	0.53	0.04	1.93	0.13	1.02	0.07	2.14	0.16	0.51	0.07	1.04	0.06	1.27	0.09
Treated	Mz	17	Te	%	0.00	0.00	0.05	0.09	0.00	0.00	0.00	0.00	0.00	0.00	0.00	0.00	0.00	0.00	0.00	0.00	0.00	0.00	0.00	0.00
Treated	Mz	18	Ae	%	0.00	0.00	0.00	0.00	0.00	0.00	0.00	0.00	0.05	0.05	0.59	0.23	14.74	1.87	0.00	0.00	0.11	0.14	0.00	0.00
Treated	Mz	18	Ca	%	0.00	0.00	0.00	0.00	0.00	0.00	0.00	0.00	0.00	0.00	0.00	0.00	0.00	0.00	0.00	0.00	0.11	0.14	0.00	0.00
Treated	Mz	18	E	GPa	3.44	0.17	4.57	0.23	4.26	0.21	4.27	0.30	6.79	0.42	8.56	0.47	18.19	1.01	4.10	0.53	8.47	0.46	9.57	0.53
Treated	Mz	18	Fe	%	0.00	0.00	0.00	0.00	0.00	0.00	0.00	0.00	0.03	0.05	0.59	0.23	14.74	1.87	0.00	0.00	0.00	0.00	0.00	0.00
Treated	Mz	18	H	GPa	0.40	0.02	0.54	0.03	0.48	0.03	0.64	0.04	1.95	0.14	1.12	0.08	2.23	0.16	0.54	0.07	1.09	0.06	1.33	0.09
Treated	Mz	18	Te	%	0.00	0.00	0.00	0.00	0.00	0.34	0.00	0.00	0.02	0.00	0.00	0.00	0.00	0.00	0.00	0.00	0.00	0.00	0.00	0.00
Treated	Mz	19	Ae	%	0.00	0.00	0.00	0.00	0.14	0.34	0.00	0.00	0.21	0.33	0.78	0.74	12.19	1.56	0.00	0.00	0.00	0.00	0.00	0.00
Treated	Mz	19	Ca	%	0.00	0.00	0.00	0.00	0.00	0.00	0.00	0.00	0.00	0.00	0.00	0.00	0.00	0.00	0.00	0.00	0.00	0.00	0.00	0.00
Treated	Mz	19	E	GPa	3.89	0.20	5.48	0.27	5.01	0.25	5.14	0.36	7.14	0.60	9.22	0.51	18.75	1.04	4.04	0.22	8.89	0.48	10.01	0.56
Treated	Mz	19	Fe	%	0.00	0.00	0.00	0.00	0.00	0.00	0.00	0.00	0.21	0.33	0.78	0.74	12.19	1.56	0.00	0.00	0.00	0.00	0.00	0.00
Treated	Mz	19	H	GPa	0.46	0.02	0.65	0.03	0.56	0.04	0.73	0.05	2.09	0.15	1.21	0.08	2.29	0.16	0.53	0.04	1.14	0.06	1.39	0.10
Treated	Mz	19	Te	%	0.00	0.00	0.00	0.00	0.14	0.00	0.00	0.00	0.00	0.04	0.00	0.00	0.00	0.00	0.00	0.00	0.00	0.00	0.00	0.00
Treated	Mz	20	Ae	%	0.00	0.00	0.06	0.11	0.00	0.00	0.00	0.00	0.33	0.49	1.06	0.33	11.35	1.92	0.14	0.15	0.00	0.00	0.05	0.08
Treated	Mz	20	Ca	%	0.00	0.00	0.06	0.11	0.00	0.00	0.00	0.00	0.14	0.34	0.00	0.00	0.00	0.00	0.14	0.15	0.00	0.00	0.05	0.08
Treated	Mz	20	E	GPa	4.31	0.22	6.48	0.32	4.94	0.61	5.85	0.41	7.40	0.66	9.61	0.53	19.30	1.07	4.22	0.23	7.21	0.73	10.45	0.58
Treated	Mz	20	Fe	%	0.00	0.00	0.00	0.00	0.00	0.00	0.00	0.00	0.03	0.05	1.06	0.33	11.35	1.92	0.00	0.00	0.00	0.00	0.00	0.00
Treated	Mz	20	H	GPa	0.51	0.03	0.76	0.04	0.55	0.06	0.75	0.05	2.08	0.15	1.26	0.09	2.36	0.17	0.55	0.04	1.20	0.07	1.46	0.10
Treated	Mz	20	Te	%	0.00	0.00	0.00	0.00	0.00	0.00	0.00	0.00	0.16	0.00	0.00	0.00	0.00	0.00	0.00	0.00	0.00	0.00	0.00	0.00
Treated	Mz	21	Ae	%	0.00	0.00	0.00	0.00	0.00	0.00	0.02	0.04	0.19	0.15	1.23	0.19	14.57	1.63	0.00	0.00	0.00	0.00	0.00	0.00
Treated	Mz	21	Ca	%	0.00	0.00	0.00	0.00	0.00	0.00	0.00	0.00	0.00	0.00	0.00	0.00	0.00	0.00	0.00	0.00	0.00	0.00	0.00	0.00
Treated	Mz	21	E	GPa	4.64	0.23	6.41	0.32	4.65	0.23	5.98	0.44	7.67	0.83	10.48	0.58	20.68	1.15	4.40	0.24	7.42	0.81	8.43	0.47
Treated	Mz	21	Fe	%	0.00	0.00	0.00	0.00	0.00	0.00	0.02	0.04	0.19	0.15	1.23	0.19	14.57	1.63	0.00	0.00	0.00	0.00	0.00	0.00
Treated	Mz	21	H	GPa	0.55	0.03	0.75	0.04	0.52	0.04	0.87	0.06	2.09	0.15	1.38	0.10	2.53	0.18	0.58	0.04	1.26	0.07	1.17	0.08
Treated	Mz	21	Te	%	0.00	0.00	0.00	0.00	0.00	0.00	0.00	0.00	0.00	0.31	0.00	0.00	0.00	0.00	0.00	0.00	0.00	0.00	0.00	0.00
Treated	Mz	22	Ae	%	0.00	0.00	0.00	0.00	0.00	0.00	0.24	0.34	0.21	0.07	2.57	1.50	14.65	1.64	0.00	0.00	0.06	0.10	0.00	0.00
Treated	Mz	22	Ca	%	0.00	0.00	0.00	0.00	0.00	0.00	0.17	0.27	0.00	0.00	0.00	0.00	0.00	0.00	0.00	0.00	0.00	0.00	0.00	0.00
Treated	Mz	22	E	GPa	5.18	0.37	7.66	0.38	5.01	0.25	6.93	0.49	7.15	0.35	11.25	0.48	21.07	1.17	4.61	0.24	7.04	0.53	8.79	0.49
Treated	Mz	22	Fe	%	0.00	0.00	0.00	0.00	0.00	0.00	0.00	0.00	0.21	0.07	2.57	1.50	14.65	1.64	0.00	0.00	0.06	0.10	0.00	0.00
Treated	Mz	22	H	GPa	0.61	0.04	0.90	0.05	0.56	0.04	0.98	0.07	2.16	0.15	1.45	0.11	2.58	0.18	0.60	0.04	1.31	0.07	1.22	0.09

Treated	Mz	22	Te	%	0.00	0.00	0.00	0.00	0.00	0.10	0.07	0.12	0.00	0.00	0.00	0.00	0.00	0.00	0.00	0.00	0.00	0.00	0.00	0.00
Treated	Mz	23	Ae	%	0.00	0.00	0.00	0.00	0.04	0.10	0.00	0.00	0.21	0.16	6.26	2.13	17.00	2.17	0.17	0.10	0.11	0.10	0.07	0.09
Treated	Mz	23	Ca	%	0.00	0.00	0.00	0.00	0.00	0.00	0.00	0.00	0.00	0.00	0.00	0.00	0.00	0.00	0.17	0.10	0.11	0.10	0.07	0.09
Treated	Mz	23	E	GPa	5.80	0.29	8.26	0.41	5.86	0.30	7.96	0.40	7.96	0.72	11.26	0.62	21.75	1.21	4.84	0.26	7.12	0.42	9.15	0.51
Treated	Mz	23	Fe	%	0.00	0.00	0.00	0.00	0.00	0.00	0.00	0.00	0.21	0.16	6.26	2.13	17.00	2.17	0.00	0.00	0.00	0.00	0.00	0.00
Treated	Mz	23	H	GPa	0.68	0.03	0.97	0.05	0.66	0.05	1.08	0.08	2.18	0.15	1.48	0.10	2.66	0.19	0.63	0.04	1.35	0.06	1.27	0.09
Treated	Mz	23	Te	%	0.00	0.00	0.00	0.00	0.04	0.00	0.00	0.00	0.00	0.00	0.00	0.00	0.00	0.00	0.00	0.00	0.00	0.00	0.00	0.00
Treated	Mz	24	Ae	%	0.37	0.65	0.00	0.00	0.00	0.00	0.00	0.00	0.44	0.08	6.48	2.39	19.17	2.23	0.00	0.00	0.05	0.12	0.00	0.00
Treated	Mz	24	Ca	%	0.00	0.00	0.00	0.00	0.00	0.00	0.00	0.00	0.00	0.00	0.00	0.00	0.00	0.00	0.00	0.00	0.05	0.12	0.00	0.00
Treated	Mz	24	E	GPa	6.67	0.33	9.34	0.21	6.44	0.32	8.60	0.60	7.96	0.34	11.36	0.63	22.98	1.27	5.31	0.34	7.39	0.11	9.71	0.54
Treated	Mz	24	Fe	%	0.37	0.65	0.00	0.00	0.00	0.00	0.00	0.00	0.44	0.08	6.48	2.39	19.17	2.23	0.00	0.00	0.00	0.00	0.00	0.00
Treated	Mz	24	H	GPa	0.78	0.04	1.07	0.05	0.72	0.05	1.10	0.08	2.17	0.16	1.49	0.10	2.81	0.20	0.69	0.05	1.54	0.08	1.35	0.09
Treated	Mz	24	Te	%	0.00	0.00	0.00	0.00	0.00	0.00	0.00	0.00	0.00	0.00	0.00	0.00	0.00	0.00	0.00	0.00	0.00	0.00	0.00	0.00
Treated	Mz	25	Ae	%	0.00	0.00	0.00	0.00	0.00	0.00	0.00	0.00	0.67	0.13	7.37	1.25	20.69	2.57	0.00	0.00	0.00	0.00	0.00	0.00
Treated	Mz	25	Ca	%	0.00	0.00	0.00	0.00	0.00	0.00	0.00	0.00	0.07	0.11	0.00	0.00	0.00	0.00	0.00	0.00	0.00	0.00	0.00	0.00
Treated	Mz	25	E	GPa	6.65	0.33	9.68	0.49	6.76	0.34	8.78	0.61	8.33	0.74	11.92	0.66	23.94	1.33	5.22	0.29	7.25	0.62	10.24	0.57
Treated	Mz	25	Fe	%	0.00	0.00	0.00	0.00	0.00	0.00	0.00	0.00	0.60	0.07	7.37	1.25	20.69	2.57	0.00	0.00	0.00	0.00	0.00	0.00
Treated	Mz	25	H	GPa	0.78	0.04	1.14	0.06	0.76	0.05	1.11	0.08	2.23	0.16	1.56	0.11	2.93	0.20	0.69	0.05	1.49	0.08	1.43	0.10
Treated	Mz	25	Te	%	0.00	0.00	0.00	0.00	0.00	0.00	0.00	0.00	0.00	0.00	0.00	0.00	0.00	0.00	0.00	0.00	0.00	0.00	0.00	0.00
Treated	Mz	26	Ae	%	0.60	1.04	0.00	0.00	0.00	0.00	0.13	0.15	0.51	0.64	5.07	1.23	26.15	3.20	0.00	0.00	0.00	0.00	0.08	0.09
Treated	Mz	26	Ca	%	0.60	1.04	0.00	0.00	0.00	0.00	0.13	0.15	0.45	0.62	0.00	0.00	0.00	0.00	0.00	0.00	0.00	0.00	0.08	0.09
Treated	Mz	26	E	GPa	7.75	0.19	10.02	0.21	7.77	0.43	8.98	0.70	8.39	0.74	12.47	0.69	25.10	1.39	4.53	0.25	7.50	0.26	10.46	0.58
Treated	Mz	26	Fe	%	0.00	0.00	0.00	0.00	0.00	0.00	0.00	0.00	0.06	0.05	5.07	1.23	26.15	3.20	0.00	0.00	0.00	0.00	0.00	0.00
Treated	Mz	26	H	GPa	0.88	0.04	1.15	0.06	0.86	0.06	1.22	0.09	2.23	0.16	1.64	0.11	3.07	0.21	0.60	0.04	1.57	0.09	1.46	0.10
Treated	Mz	26	Te	%	0.00	0.00	0.00	0.00	0.00	0.00	0.00	0.00	0.00	0.00	0.00	0.00	0.00	0.00	0.00	0.00	0.00	0.00	0.00	0.00
Treated	Mz	27	Ae	%	0.00	0.00	0.00	0.00	0.00	0.00	0.00	0.00	0.36	0.11	10.22	2.74	27.60	4.08	0.00	0.00	0.00	0.00	0.00	0.00
Treated	Mz	27	Ca	%	0.00	0.00	0.00	0.00	0.00	0.00	0.00	0.00	0.00	0.00	0.00	0.00	0.00	0.00	0.00	0.00	0.00	0.00	0.00	0.00
Treated	Mz	27	E	GPa	7.88	0.36	10.48	0.21	7.53	0.45	9.91	0.50	8.00	0.53	11.47	0.64	25.50	1.60	4.93	0.30	7.14	0.51	10.52	0.58
Treated	Mz	27	Fe	%	0.00	0.00	0.00	0.00	0.00	0.00	0.00	0.00	0.36	0.11	10.22	2.74	27.60	4.08	0.00	0.00	0.00	0.00	0.00	0.00
Treated	Mz	27	H	GPa	0.83	0.04	1.20	0.06	0.81	0.06	1.32	0.10	2.25	0.16	1.51	0.11	3.08	0.22	0.67	0.05	1.58	0.09	1.47	0.10
Treated	Mz	27	Te	%	0.00	0.00	0.00	0.00	0.00	0.00	0.00	0.00	0.00	0.00	0.00	0.00	0.00	0.00	0.00	0.00	0.00	0.00	0.00	0.00
Treated	Wz	28	Ae	%	0.04	0.06	0.00	0.00	0.00	0.00	0.00	0.00	0.36	0.16	9.37	2.01	26.91	4.31	0.00	0.00	0.00	0.00	0.11	0.09
Treated	Wz	28	Ca	%	0.04	0.06	0.00	0.00	0.00	0.00	0.00	0.00	0.00	0.00	0.00	0.00	0.25	0.62	0.00	0.00	0.00	0.00	0.11	0.09
Treated	Wz	28	E	GPa	8.22	0.19	11.13	0.56	7.72	0.39	10.57	0.77	9.25	0.71	11.41	0.63	28.31	1.58	4.58	0.26	7.14	0.44	9.94	0.55
Treated	Wz	28	Fe	%	0.00	0.00	0.00	0.00	0.00	0.00	0.00	0.00	0.36	0.16	9.37	2.01	26.64	4.30	0.00	0.00	0.00	0.00	0.00	0.00
Treated	Wz	28	H	GPa	0.94	0.05	1.31	0.07	0.87	0.06	1.31	0.09	2.26	0.16	1.50	0.10	3.48	0.24	0.61	0.04	1.60	0.09	1.38	0.10
Treated	Wz	28	Te	%	0.00	0.00	0.00	0.00	0.00	0.00	0.00	0.00	0.00	0.00	0.00	0.00	0.02	0.04	0.00	0.00	0.00	0.00	0.00	0.00
Treated	Wz	29	Ae	%	0.00	0.00	0.00	0.00	0.00	0.00	0.00	0.00	0.53	0.20	10.21	0.95	30.77	6.15	0.00	0.00	0.00	0.00	0.15	0.03
Treated	Wz	29	Ca	%	0.00	0.00	0.00	0.00	0.00	0.00	0.00	0.00	0.00	0.00	0.22	0.47	0.45	0.89	0.00	0.00	0.00	0.00	0.15	0.03
Treated	Wz	29	E	GPa	7.77	0.59	11.50	0.58	7.75	0.39	10.47	0.73	10.51	0.67	10.38	0.58	28.93	3.92	4.37	0.25	7.06	0.31	9.44	0.52
Treated	Wz	29	Fe	%	0.00	0.00	0.00	0.00	0.00	0.00	0.00	0.00	0.48	0.13	9.99	1.07	30.32	5.39	0.00	0.00	0.00	0.00	0.00	0.00
Treated	Wz	29	H	GPa	0.89	0.04	1.35	0.07	0.87	0.06	1.42	0.10	2.00	0.14	1.36	0.10	3.55	0.38	0.58	0.04	1.45	0.08	1.31	0.09
Treated	Wz	29	Te	%	0.00	0.00	0.00	0.00	0.00	0.00	0.00	0.00	0.04	0.00	0.00	0.00	0.00	0.00	0.00	0.00	0.00	0.00	0.00	0.00
Treated	Wz	30	Ae	%	0.13	0.23	0.08	0.14	0.00	0.00	0.00	0.00	0.41	0.23	9.25	2.25	30.60	3.43	0.00	0.00	0.00	0.00	0.34	0.15
Treated	Wz	30	Ca	%	0.00	0.00	0.08	0.14	0.00	0.00	0.00	0.00	0.00	0.00	1.38	0.78	0.10	0.13	0.00	0.00	0.00	0.00	0.34	0.15
Treated	Wz	30	E	GPa	8.37	0.42	12.44	0.62	8.15	0.41	11.41	0.81	9.79	1.09	10.78	0.60	31.50	1.76	4.85	0.17	7.85	0.36	9.63	0.53
Treated	Wz	30	Fe	%	0.13	0.23	0.00	0.00	0.00	0.00	0.00	0.00	0.41	0.23	7.87	1.61	30.50	3.32	0.00	0.00	0.00	0.00	0.00	0.00
Treated	Wz	30	H	GPa	0.99	0.05	1.46	0.07	0.91	0.06	1.43	0.10	1.73	0.17	1.41	0.10	3.88	0.27	0.62	0.04	1.51	0.22	1.34	0.09
Treated	Wz	30	Te	%	0.00	0.00	0.00	0.00	0.00	0.00	0.00	0.00	0.00	0.10	0.00	0.00	0.00	0.00	0.00	0.00	0.00	0.00	0.00	0.00
Treated	Wz	31	Ae	%	0.00	0.00	0.00	0.00	0.00	0.00	0.00	0.00	0.74	0.35	9.32	1.44	26.54	3.79	0.00	0.00	0.00	0.00	0.00	0.00
Treated	Wz	31	Ca	%	0.00	0.00	0.00	0.00	0.00	0.00	0.00	0.00	0.00	0.00	0.44	0.58	0.29	0.57	0.00	0.00	0.00	0.00	0.00	0.00
Treated	Wz	31	E	GPa	8.05	0.40	12.50	0.63	7.85	0.40	11.88	0.83	9.37	1.18	9.78	0.54	31.01	1.79	5.36	0.30	8.73	0.47	9.66	0.59
Treated	Wz	31	Fe	%	0.00	0.00	0.00	0.00	0.00	0.00	0.00	0.00	0.60	0.07	8.88	1.40	26.26	3.66	0.00	0.00	0.00	0.00	0.00	0.00
Treated	Wz	31	H	GPa	0.95	0.05	1.47	0.07	0.88	0.06	1.53	0.11	1.48	0.10	1.28	0.09	3.95	0.28	0.67	0.05	1.12	0.06	1.32	0.08

Treated	Wz	31	Te	%	0.00	0.00	0.00	0.00	0.00	0.00	0.00	0.00	0.13	0.00	0.00	0.00	0.00	0.00	0.00	0.00	0.00	0.00	0.00	0.00
Treated	Wz	32	Ae	%	0.00	0.00	0.00	0.00	0.00	0.00	0.00	0.00	0.58	0.20	10.83	2.42	28.32	4.03	0.00	0.00	0.00	0.00	0.22	0.12
Treated	Wz	32	Ca	%	0.00	0.00	0.00	0.00	0.00	0.00	0.00	0.00	0.00	0.00	1.10	1.10	0.00	0.00	0.00	0.00	0.00	0.00	0.22	0.12
Treated	Wz	32	E	GPa	8.34	0.42	12.64	0.63	8.67	0.44	12.79	0.89	10.73	1.69	10.72	0.46	30.55	1.84	4.90	0.27	8.47	1.02	9.80	0.54
Treated	Wz	32	Fe	%	0.00	0.00	0.00	0.00	0.00	0.00	0.00	0.00	0.56	0.18	9.73	1.87	28.32	4.03	0.00	0.00	0.00	0.00	0.00	0.00
Treated	Wz	32	H	GPa	0.98	0.05	1.49	0.07	0.97	0.07	1.54	0.09	1.57	0.11	1.39	0.11	4.05	0.28	0.61	0.04	1.11	0.07	1.36	0.10
Treated	Wz	32	Te	%	0.00	0.00	0.00	0.00	0.00	0.00	0.00	0.00	0.02	0.33	0.00	0.00	0.00	0.00	0.00	0.00	0.00	0.00	0.00	0.00
Treated	Wz	33	Ae	%	0.00	0.00	0.00	0.00	0.00	0.00	0.00	0.00	0.71	0.28	10.72	1.90	27.65	4.17	0.00	0.00	0.00	0.00	0.08	0.13
Treated	Wz	33	Ca	%	0.00	0.00	0.00	0.00	0.00	0.00	0.00	0.00	0.00	0.00	0.75	0.88	0.00	0.00	0.00	0.00	0.00	0.00	0.08	0.13
Treated	Wz	33	E	GPa	8.97	0.18	13.16	0.66	9.48	0.55	12.81	0.73	9.35	0.99	10.36	0.68	28.13	2.11	4.52	0.25	8.81	0.88	9.58	0.53
Treated	Wz	33	Fe	%	0.00	0.00	0.00	0.00	0.00	0.00	0.00	0.00	0.71	0.28	9.97	1.55	27.65	4.17	0.00	0.00	0.00	0.00	0.00	0.00
Treated	Wz	33	H	GPa	1.03	0.05	1.55	0.08	1.02	0.07	1.54	0.09	1.49	0.10	1.33	0.09	3.66	0.26	0.55	0.03	1.11	0.06	1.33	0.09
Treated	Wz	33	Te	%	0.00	0.00	0.00	0.00	0.00	0.00	0.00	0.00	0.00	0.04	0.00	0.00	0.00	0.00	0.00	0.00	0.00	0.00	0.00	0.00
Treated	Wz	34	Ae	%	0.17	0.29	0.00	0.00	0.00	0.00	0.00	0.00	0.24	0.13	8.14	1.13	28.65	3.11	0.00	0.00	0.00	0.00	0.00	0.00
Treated	Wz	34	Ca	%	0.00	0.00	0.00	0.00	0.00	0.00	0.00	0.00	0.00	0.00	0.58	0.64	0.00	0.00	0.00	0.00	0.00	0.00	0.00	0.00
Treated	Wz	34	E	GPa	8.86	0.26	13.13	0.66	9.36	0.50	12.79	0.73	10.19	1.07	10.67	0.59	30.77	1.85	5.25	0.25	9.44	0.55	9.75	0.54
Treated	Wz	34	Fe	%	0.15	0.26	0.00	0.00	0.00	0.00	0.00	0.00	0.24	0.13	7.56	0.95	28.65	3.11	0.00	0.00	0.00	0.00	0.00	0.00
Treated	Wz	34	H	GPa	0.99	0.05	1.55	0.08	0.98	0.08	1.51	0.08	1.62	0.11	1.40	0.10	4.08	0.28	0.60	0.04	1.13	0.07	1.25	0.07
Treated	Wz	34	Te	%	0.02	0.03	0.00	0.00	0.00	0.00	0.00	0.00	0.00	0.00	0.00	0.00	0.00	0.00	0.00	0.00	0.00	0.00	0.00	0.00
Treated	Wz	35	Ae	%	0.04	0.08	0.00	0.00	0.00	0.00	0.00	0.00	0.08	0.07	10.31	1.72	28.31	4.03	0.00	0.00	0.00	0.00	0.07	0.11
Treated	Wz	35	Ca	%	0.00	0.00	0.00	0.00	0.00	0.00	0.00	0.00	0.00	0.00	0.61	0.67	0.00	0.00	0.00	0.00	0.00	0.00	0.07	0.11
Treated	Wz	35	E	GPa	9.13	0.31	13.67	0.46	9.41	0.41	12.75	0.56	10.04	0.64	10.53	0.69	30.09	2.14	5.54	0.27	9.50	0.51	9.76	0.54
Treated	Wz	35	Fe	%	0.04	0.08	0.00	0.00	0.00	0.00	0.00	0.00	0.08	0.07	9.71	1.55	28.31	4.03	0.00	0.00	0.00	0.00	0.00	0.00
Treated	Wz	35	H	GPa	0.99	0.05	1.54	0.07	0.99	0.06	1.50	0.08	1.58	0.11	1.35	0.09	3.73	0.26	0.64	0.04	1.14	0.06	1.26	0.07
Treated	Wz	35	Te	%	0.00	0.00	0.00	0.00	0.00	0.00	0.00	0.00	0.00	0.00	0.00	0.00	0.00	0.00	0.00	0.00	0.00	0.00	0.00	0.00
Treated	Wz	36	Ae	%	0.52	0.11	0.37	0.32	0.00	0.00	0.00	0.00	0.10	0.08	6.03	2.25	28.99	3.43	0.00	0.00	0.07	0.11	0.00	0.00
Treated	Wz	36	Ca	%	0.52	0.11	0.37	0.32	0.00	0.00	0.00	0.00	0.00	0.00	0.41	0.59	0.00	0.00	0.00	0.00	0.07	0.11	0.00	0.00
Treated	Wz	36	E	GPa	9.06	0.43	13.35	0.35	9.34	0.47	12.77	0.44	9.88	0.65	10.63	0.59	32.87	1.89	5.39	1.00	9.61	0.52	9.20	0.51
Treated	Wz	36	Fe	%	0.00	0.00	0.00	0.00	0.00	0.00	0.00	0.00	0.10	0.08	5.62	2.47	28.99	3.43	0.00	0.00	0.00	0.00	0.00	0.00
Treated	Wz	36	H	GPa	0.98	0.07	1.41	0.06	0.99	0.07	1.51	0.09	1.56	0.11	1.39	0.10	4.16	0.29	0.67	0.04	1.15	0.06	1.18	0.07
Treated	Wz	36	Te	%	0.00	0.00	0.00	0.00	0.00	0.00	0.00	0.00	0.00	0.00	0.00	0.00	0.00	0.00	0.00	0.00	0.00	0.00	0.00	0.00
Treated	Wz	37	Ae	%	0.24	0.26	0.50	0.46	0.00	0.00	0.00	0.00	0.14	0.04	7.07	0.73	28.21	2.49	0.04	0.10	0.35	0.62	0.00	0.00
Treated	Wz	37	Ca	%	0.17	0.30	0.50	0.46	0.00	0.00	0.00	0.00	0.00	0.00	0.07	0.16	0.00	0.00	0.00	0.00	0.35	0.62	0.00	0.00
Treated	Wz	37	E	GPa	9.67	0.19	12.62	0.54	9.67	0.49	12.90	0.44	9.88	0.65	11.16	0.62	29.29	2.83	5.63	0.21	9.72	0.52	9.40	0.52
Treated	Wz	37	Fe	%	0.00	0.00	0.00	0.00	0.00	0.00	0.00	0.00	0.13	0.02	7.00	0.81	28.21	2.49	0.00	0.00	0.00	0.00	0.00	0.00
Treated	Wz	37	H	GPa	1.09	0.05	1.49	0.08	1.02	0.07	1.59	0.09	1.56	0.11	1.46	0.10	3.76	0.26	0.57	0.04	1.17	0.06	1.21	0.07
Treated	Wz	37	Te	%	0.07	0.12	0.00	0.00	0.00	0.00	0.00	0.00	0.01	0.00	0.00	0.00	0.00	0.00	0.04	0.10	0.00	0.00	0.00	0.00
Treated	Wz	38	Ae	%	0.66	0.69	0.00	0.00	0.00	0.00	0.00	0.00	0.27	0.47	7.36	1.73	27.25	3.10	0.02	0.05	0.00	0.00	0.00	0.00
Treated	Wz	38	Ca	%	0.25	0.03	0.00	0.00	0.00	0.00	0.00	0.00	0.18	0.44	0.00	0.00	0.00	0.00	0.00	0.00	0.00	0.00	0.00	0.00
Treated	Wz	38	E	GPa	9.01	0.23	13.57	0.68	9.79	0.49	13.25	0.75	10.78	0.46	11.38	0.63	28.03	1.81	5.63	0.27	9.83	0.53	9.79	0.54
Treated	Wz	38	Fe	%	0.35	0.60	0.00	0.00	0.00	0.00	0.00	0.00	0.09	0.07	7.33	1.74	27.25	3.10	0.00	0.00	0.00	0.00	0.00	0.00
Treated	Wz	38	H	GPa	1.03	0.05	1.60	0.08	1.04	0.07	1.57	0.09	1.57	0.11	1.49	0.10	3.83	0.28	0.64	0.05	1.18	0.06	1.26	0.07
Treated	Wz	38	Te	%	0.07	0.12	0.00	0.00	0.00	0.00	0.00	0.00	0.00	0.03	0.03	0.07	0.00	0.00	0.02	0.05	0.00	0.00	0.00	0.00
Treated	Wz	39	Ae	%	0.00	0.00	0.00	0.00	0.00	0.00	0.00	0.00	0.66	0.94	7.25	1.20	32.08	2.77	0.26	0.36	0.15	0.24	0.00	0.00
Treated	Wz	39	Ca	%	0.00	0.00	0.00	0.00	0.00	0.00	0.00	0.00	0.54	0.89	0.00	0.00	0.03	0.07	0.26	0.36	0.00	0.00	0.00	0.00
Treated	Wz	39	E	GPa	9.16	0.27	13.35	0.67	9.93	0.50	12.80	0.49	9.76	0.63	11.76	0.65	28.77	1.55	5.12	0.85	9.95	0.54	9.51	0.53
Treated	Wz	39	Fe	%	0.00	0.00	0.00	0.00	0.00	0.00	0.00	0.00	0.12	0.10	7.25	1.20	32.04	2.70	0.00	0.00	0.15	0.24	0.00	0.00
Treated	Wz	39	H	GPa	1.04	0.05	1.57	0.08	1.05	0.07	0.33	0.05	1.70	0.12	1.54	0.11	3.95	0.28	0.71	0.05	1.19	0.06	1.21	0.07
Treated	Wz	39	Te	%	0.00	0.00	0.00	0.00	0.00	0.00	0.00	0.00	0.00	0.00	0.00	0.00	0.01	0.03	0.00	0.00	0.00	0.00	0.00	0.00

Supplementary Table 12. Gain or loss from one tooth row (Tr) to the adjacent tooth row for each tooth part (P), given in %, (the highest values for each parameter was set as 100%) for E, H, Fe, Ca, Ae (all elements), and Te (all elements without Ca and Fe) are shown.

Condition	P	Tr	Gain or loss of, %					
			E, %	Fe, %	Ca,	Ae	H	A-CaFe
Native	Central tooth Cusp	1	0.62	0.00	0.00	0.00	0.67	0.00
Native	Central tooth Stylus	1	0.67	0.00	4.48	4.48	0.68	0.00
Native	Lateral tooth I Cusp	1	0.69	0.00	0.00	0.00	0.69	0.00
Native	Lateral tooth I Stylus	1	0.23	0.00	0.00	0.00	0.25	0.00
Native	Lateral tooth II Basis	1	0.61	0.00	0.00	0.00	0.60	0.00
Native	Lateral tooth II Cusp	1	3.31	0.00	0.00	0.05	3.31	0.54
Native	Lateral tooth II Stylus	1	0.60	0.13	0.00	-1.61	0.60	-15.56
Native	Marginal tooth Basis	1	1.93	0.00	0.00	2.56	1.97	7.04
Native	Marginal tooth Cusp	1	-0.69	-100.00	0.00	-28.36	-0.71	0.00
Native	Marginal tooth Stylus	1	1.04	0.00	0.00	0.00	1.06	0.00
Native	Central tooth Cusp	2	-0.11	0.00	1.29	1.29	-0.14	0.00
Native	Central tooth Stylus	2	0.45	0.00	-4.48	-4.48	0.45	0.00
Native	Lateral tooth I Cusp	2	-0.92	0.00	0.00	0.00	-0.95	0.00
Native	Lateral tooth I Stylus	2	-0.53	0.00	0.00	0.00	-0.57	0.00
Native	Lateral tooth II Basis	2	5.74	0.00	0.00	0.00	5.33	0.00
Native	Lateral tooth II Cusp	2	3.31	0.00	5.98	1.90	3.32	2.16
Native	Lateral tooth II Stylus	2	0.09	-0.13	0.00	1.45	0.12	14.07
Native	Marginal tooth Basis	2	0.84	0.00	0.00	3.08	1.40	8.45
Native	Marginal tooth Cusp	2	1.77	0.00	0.00	0.00	1.79	0.00
Native	Marginal tooth Stylus	2	0.80	100.00	0.00	11.48	0.80	0.00
Native	Central tooth Cusp	3	0.45	0.00	-1.29	-1.29	0.46	0.00
Native	Central tooth Stylus	3	0.07	0.00	0.00	0.00	0.07	0.00
Native	Lateral tooth I Cusp	3	0.74	0.00	16.84	16.84	0.79	0.00
Native	Lateral tooth I Stylus	3	0.31	0.00	0.00	0.00	0.32	0.00
Native	Lateral tooth II Basis	3	4.08	0.00	51.81	40.32	4.01	0.00
Native	Lateral tooth II Cusp	3	2.28	0.00	-3.93	-1.16	2.26	-0.54
Native	Lateral tooth II Stylus	3	0.32	0.00	4.71	0.88	0.30	-14.07
Native	Marginal tooth Basis	3	6.74	0.00	0.00	-8.72	6.25	-23.94
Native	Marginal tooth Cusp	3	-0.52	0.00	0.00	0.00	-0.53	0.00
Native	Marginal tooth Stylus	3	0.67	-100.00	8.61	-2.87	0.62	0.00
Native	Central tooth Cusp	4	-0.11	0.00	0.00	0.00	-0.08	0.00
Native	Central tooth Stylus	4	-1.04	0.00	6.34	6.34	-1.03	0.00
Native	Lateral tooth I Cusp	4	-0.29	100.00	-16.84	-12.46	-0.32	0.00
Native	Lateral tooth I Stylus	4	0.23	0.00	0.00	0.00	0.25	0.00
Native	Lateral tooth II Basis	4	1.93	0.00	-0.90	-0.70	1.82	0.00
Native	Lateral tooth II Cusp	4	4.37	0.00	1.34	0.18	4.37	-2.16
Native	Lateral tooth II Stylus	4	0.00	0.00	-2.20	-0.48	0.00	5.93
Native	Marginal tooth Basis	4	-0.36	0.00	0.00	10.77	-0.38	29.58
Native	Marginal tooth Cusp	4	0.52	0.00	3.13	2.99	0.49	0.00
Native	Marginal tooth Stylus	4	-0.92	100.00	-8.61	2.87	-0.92	0.00
Native	Central tooth Cusp	5	-0.96	0.00	2.15	2.15	-1.01	0.00
Native	Central tooth Stylus	5	0.52	0.00	-6.34	-6.34	0.54	0.00
Native	Lateral tooth I Cusp	5	0.80	-100.00	8.75	4.38	0.82	0.00
Native	Lateral tooth I Stylus	5	-0.53	0.00	0.00	0.00	-0.58	0.00
Native	Lateral tooth II Basis	5	6.13	0.00	10.63	8.27	5.86	0.00
Native	Lateral tooth II Cusp	5	8.98	1.26	10.26	3.87	8.98	0.00
Native	Lateral tooth II Stylus	5	3.45	0.00	6.12	5.55	3.22	22.22
Native	Marginal tooth Basis	5	7.70	0.00	6.35	-4.62	7.18	-29.58
Native	Marginal tooth Cusp	5	3.21	0.00	13.28	12.69	3.23	0.00
Native	Marginal tooth Stylus	5	-0.24	-100.00	13.52	2.05	-0.22	0.00
Native	Central tooth Cusp	6	0.96	0.00	6.87	6.87	0.98	0.00
Native	Central tooth Stylus	6	0.15	0.00	0.00	0.00	0.18	0.00
Native	Lateral tooth I Cusp	6	-1.09	0.00	-8.75	-8.75	-1.10	0.00
Native	Lateral tooth I Stylus	6	0.38	0.00	0.00	0.00	0.38	0.00
Native	Lateral tooth II Basis	6	6.57	19.72	7.69	8.27	6.23	0.00
Native	Lateral tooth II Cusp	6	4.68	0.46	-2.59	-0.40	4.68	0.00
Native	Lateral tooth II Stylus	6	1.47	2.00	-1.88	3.14	1.70	26.67
Native	Marginal tooth Basis	6	-5.17	0.00	1.59	1.54	-4.68	0.00
Native	Marginal tooth Cusp	6	2.18	0.00	-9.38	-8.96	2.19	0.00
Native	Marginal tooth Stylus	6	3.86	0.00	-13.52	-13.52	3.82	0.00
Native	Central tooth Cusp	7	0.00	32.50	0.86	6.44	0.03	0.00
Native	Central tooth Stylus	7	0.82	0.00	0.00	0.00	0.77	0.00
Native	Lateral tooth I Cusp	7	0.86	0.00	0.00	0.00	0.86	0.00
Native	Lateral tooth I Stylus	7	0.15	0.00	0.00	0.00	0.18	0.00
Native	Lateral tooth II Basis	7	5.79	9.86	-21.27	-15.14	5.50	0.00
Native	Lateral tooth II Cusp	7	2.71	5.79	6.51	6.30	2.72	0.00
Native	Lateral tooth II Stylus	7	3.73	1.46	0.00	-1.77	3.75	-23.70
Native	Marginal tooth Basis	7	4.69	0.00	22.75	22.05	4.67	0.00
Native	Marginal tooth Cusp	7	5.90	0.00	35.16	33.58	5.89	0.00
Native	Marginal tooth Stylus	7	1.47	0.00	0.00	0.00	1.53	0.00
Native	Central tooth Cusp	8	0.06	-32.50	-9.87	-15.45	0.02	0.00
Native	Central tooth Stylus	8	0.07	36.36	0.00	7.46	0.09	0.00
Native	Lateral tooth I Cusp	8	-0.40	0.00	0.00	0.00	-0.39	0.00
Native	Lateral tooth I Stylus	8	0.38	0.00	0.00	0.00	0.39	0.00

Native	Lateral tooth II Basis	8	6.18	-12.68	-2.49	-3.52	5.87	0.00
Native	Lateral tooth II Cusp	8	5.71	8.20	-3.21	5.36	5.72	0.00
Native	Lateral tooth II Stylus	8	1.15	-1.33	-1.26	-4.75	1.15	-31.11
Native	Marginal tooth Basis	8	3.25	0.00	-30.69	-29.74	3.35	0.00
Native	Marginal tooth Cusp	8	3.61	0.00	27.34	26.12	3.61	0.00
Native	Marginal tooth Stylus	8	0.49	0.00	8.61	8.61	0.45	0.00
Native	Central tooth Cusp	9	-0.73	0.00	0.00	0.00	-0.71	0.00
Native	Central tooth Stylus	9	-0.97	-36.36	10.07	2.61	-0.98	0.00
Native	Lateral tooth I Cusp	9	0.06	0.00	0.00	0.00	0.08	0.00
Native	Lateral tooth I Stylus	9	-0.69	0.00	0.00	0.00	-0.72	0.00
Native	Lateral tooth II Basis	9	6.18	-4.23	7.92	5.81	5.87	0.00
Native	Lateral tooth II Cusp	9	2.18	0.93	-1.69	0.25	1.48	0.00
Native	Lateral tooth II Stylus	9	4.88	0.27	3.61	2.01	4.90	0.00
Native	Marginal tooth Basis	9	1.44	0.00	29.63	28.72	1.48	0.00
Native	Marginal tooth Cusp	9	6.01	0.00	-30.47	-29.10	6.47	0.00
Native	Marginal tooth Stylus	9	6.49	0.00	18.03	18.03	6.50	0.00
Native	Central tooth Cusp	10	0.45	0.00	17.17	17.17	0.46	0.00
Native	Central tooth Stylus	10	0.89	0.00	12.31	12.31	0.94	0.00
Native	Lateral tooth I Cusp	10	-0.34	0.00	0.00	3.37	-0.35	38.46
Native	Lateral tooth I Stylus	10	0.46	100.00	0.00	14.08	0.47	20.00
Native	Lateral tooth II Basis	10	3.81	32.39	-8.60	-1.94	3.64	2.18
Native	Lateral tooth II Cusp	10	4.43	2.65	7.40	4.23	5.12	1.08
Native	Lateral tooth II Stylus	10	3.82	5.33	9.11	8.37	3.79	4.44
Native	Marginal tooth Basis	10	3.13	0.00	-29.63	-24.62	3.05	11.27
Native	Marginal tooth Cusp	10	4.01	0.00	-39.06	-35.07	3.54	11.54
Native	Marginal tooth Stylus	10	3.86	0.00	-10.66	-9.02	3.85	33.33
Native	Central tooth Cusp	11	0.17	0.00	-17.17	-17.17	0.16	0.00
Native	Central tooth Stylus	11	0.00	0.00	-10.45	-8.96	-0.05	6.52
Native	Lateral tooth I Cusp	11	0.86	100.00	0.00	3.03	0.86	-15.38
Native	Lateral tooth I Stylus	11	0.08	-100.00	0.00	-13.59	0.11	-15.00
Native	Lateral tooth II Basis	11	2.37	-30.99	8.14	1.58	2.26	-2.18
Native	Lateral tooth II Cusp	11	1.06	4.00	14.90	7.44	1.06	2.16
Native	Lateral tooth II Stylus	11	1.11	-0.53	40.97	21.48	1.13	7.41
Native	Marginal tooth Basis	11	4.45	0.00	0.00	-3.59	4.57	-9.86
Native	Marginal tooth Cusp	11	6.01	0.00	46.88	50.00	6.00	26.92
Native	Marginal tooth Stylus	11	6.92	0.00	10.66	9.02	6.93	-25.00
Native	Central tooth Cusp	12	0.28	0.00	6.87	6.87	0.28	0.00
Native	Central tooth Stylus	12	-0.30	12.73	10.45	11.19	-0.26	-6.52
Native	Lateral tooth I Cusp	12	-0.80	-100.00	0.00	-6.06	-0.85	-19.23
Native	Lateral tooth I Stylus	12	-0.76	0.00	0.00	-0.49	-0.78	-5.00
Native	Lateral tooth II Basis	12	1.21	-9.86	-2.04	-1.76	1.13	2.62
Native	Lateral tooth II Cusp	12	5.55	5.56	21.14	10.63	5.55	3.77
Native	Lateral tooth II Stylus	12	4.93	-5.33	0.78	-3.54	4.89	-6.67
Native	Marginal tooth Basis	12	3.61	0.00	0.00	0.51	3.54	1.41
Native	Marginal tooth Cusp	12	6.87	0.00	53.13	47.76	6.87	-15.38
Native	Marginal tooth Stylus	12	6.06	0.00	6.15	7.38	6.03	16.67
Native	Central tooth Cusp	13	0.34	0.00	60.52	60.52	0.36	0.00
Native	Central tooth Stylus	13	-0.67	41.82	-22.39	-13.43	-0.68	0.00
Native	Lateral tooth I Cusp	13	-0.52	0.00	0.00	4.04	-0.49	46.15
Native	Lateral tooth I Stylus	13	0.31	0.00	0.00	3.88	0.31	40.00
Native	Lateral tooth II Basis	13	11.15	1.41	23.98	24.12	10.60	12.66
Native	Lateral tooth II Cusp	13	4.24	0.89	-0.80	-0.08	4.24	-5.93
Native	Lateral tooth II Stylus	13	7.09	5.86	-3.61	2.65	6.44	8.89
Native	Marginal tooth Basis	13	-0.24	0.00	0.00	4.10	-0.20	11.27
Native	Marginal tooth Cusp	13	5.61	0.00	-100.00	-97.01	5.62	-7.69
Native	Marginal tooth Stylus	13	6.80	0.00	-32.79	-31.56	6.82	25.00
Native	Central tooth Cusp	14	-0.45	0.00	-67.38	-67.38	-0.45	0.00
Native	Central tooth Stylus	14	0.07	-54.55	0.00	-5.60	0.04	32.61
Native	Lateral tooth I Cusp	14	0.11	0.00	7.74	4.38	0.08	-34.62
Native	Lateral tooth I Stylus	14	-0.08	0.00	59.71	61.17	-0.09	20.00
Native	Lateral tooth II Basis	14	9.82	4.23	-8.60	-11.80	9.37	-13.97
Native	Lateral tooth II Cusp	14	1.65	2.05	10.35	4.38	1.68	-1.08
Native	Lateral tooth II Stylus	14	2.35	-0.53	2.04	-0.32	3.72	-9.63
Native	Marginal tooth Basis	14	1.56	0.00	0.00	-5.13	2.53	-14.08
Native	Marginal tooth Cusp	14	5.61	0.00	85.94	82.09	5.58	-3.85
Native	Marginal tooth Stylus	14	8.57	0.00	10.25	7.79	8.55	-50.00
Native	Central tooth Cusp	15	4.78	100.00	0.00	26.18	4.88	77.78
Native	Central tooth Stylus	15	6.94	0.00	0.00	1.12	6.95	6.52
Native	Lateral tooth I Cusp	15	4.92	0.00	-7.74	0.00	4.96	84.62
Native	Lateral tooth I Stylus	15	6.41	0.00	-46.12	-47.57	6.34	-20.00
Native	Lateral tooth II Basis	15	0.50	52.11	-2.26	6.34	0.46	4.37
Native	Lateral tooth II Cusp	15	0.31	9.86	-10.79	5.97	0.30	15.90
Native	Lateral tooth II Stylus	15	6.82	3.99	2.20	6.68	6.13	28.89
Native	Marginal tooth Basis	15	4.09	0.00	34.92	40.51	3.13	18.31
Native	Marginal tooth Cusp	15	3.66	0.00	-69.53	-62.69	3.68	23.08
Native	Marginal tooth Stylus	15	3.00	0.00	33.20	37.70	3.01	91.67
Native	Central tooth Cusp	16	6.41	-100.00	40.77	22.75	6.53	-11.11
Native	Central tooth Stylus	16	-0.82	0.00	0.00	10.45	-0.81	60.87
Native	Lateral tooth I Cusp	16	6.58	23.08	0.00	-6.40	6.55	-84.62
Native	Lateral tooth I Stylus	16	0.00	0.00	1.46	-2.43	0.04	-40.00
Native	Lateral tooth II Basis	16	3.42	-12.68	-1.13	-4.75	3.70	-5.68
Native	Lateral tooth II Cusp	16	3.34	-3.54	15.25	1.11	3.34	-5.39

Native	Lateral tooth II Stylus	16	10.96	-3.73	11.77	0.88	11.16	-25.93
Native	Marginal tooth Basis	16	7.10	0.00	-34.92	-37.95	7.15	-11.27
Native	Marginal tooth Cusp	16	1.89	0.00	-16.41	-2.99	1.85	65.38
Native	Marginal tooth Stylus	16	2.82	0.00	-37.30	-36.89	2.80	8.33
Native	Central tooth Cusp	17	5.34	0.00	6.44	-1.72	5.41	-66.67
Native	Central tooth Stylus	17	9.47	0.00	0.00	-13.06	9.43	-76.09
Native	Lateral tooth I Cusp	17	6.58	-23.08	0.00	0.34	6.59	15.38
Native	Lateral tooth I Stylus	17	8.54	0.00	9.71	15.05	8.49	55.00
Native	Lateral tooth II Basis	17	0.39	-45.07	1.58	7.22	-0.09	28.82
Native	Lateral tooth II Cusp	17	1.68	7.81	-12.85	1.75	1.67	-5.93
Native	Lateral tooth II Stylus	17	8.66	-0.53	-18.37	-9.65	9.57	0.00
Native	Marginal tooth Basis	17	2.77	0.00	0.00	10.77	2.85	29.58
Native	Marginal tooth Cusp	17	2.98	0.00	0.00	-10.45	2.98	-53.85
Native	Marginal tooth Stylus	17	2.76	0.00	26.64	22.54	2.80	-83.33
Native	Central tooth Cusp	18	6.19	0.00	-36.48	-36.48	6.30	0.00
Native	Central tooth Stylus	18	4.40	0.00	0.00	-4.10	4.46	-23.91
Native	Lateral tooth I Cusp	18	6.58	0.00	0.00	-1.01	6.60	-11.54
Native	Lateral tooth I Stylus	18	5.95	0.00	8.25	5.34	5.92	-30.00
Native	Lateral tooth II Basis	18	6.35	28.17	7.24	-1.76	6.06	-27.07
Native	Lateral tooth II Cusp	18	2.03	-7.71	8.30	-3.72	0.94	-2.16
Native	Lateral tooth II Stylus	18	5.62	3.33	44.11	28.48	4.51	35.56
Native	Marginal tooth Basis	18	-1.93	0.00	0.00	-11.28	-0.84	-30.99
Native	Marginal tooth Cusp	18	2.98	0.00	0.00	-5.22	2.98	-26.92
Native	Marginal tooth Stylus	18	2.82	0.00	-19.26	-20.08	2.80	-16.67
Native	Central tooth Cusp	19	6.75	0.00	9.44	9.44	6.87	0.00
Native	Central tooth Stylus	19	4.25	0.00	15.67	15.67	4.19	0.00
Native	Lateral tooth I Cusp	19	5.38	0.00	0.00	-1.68	5.38	-19.23
Native	Lateral tooth I Stylus	19	2.59	0.00	6.80	4.37	2.57	-25.00
Native	Lateral tooth II Basis	19	-2.10	-26.76	-20.59	-18.66	1.02	1.75
Native	Lateral tooth II Cusp	19	1.34	-2.88	24.26	5.16	2.43	5.12
Native	Lateral tooth II Stylus	19	2.81	3.60	-24.02	-13.11	2.83	-27.41
Native	Marginal tooth Basis	19	3.37	0.00	26.98	24.10	2.23	-5.63
Native	Marginal tooth Cusp	19	2.98	0.00	30.47	25.37	2.98	-19.23
Native	Marginal tooth Stylus	19	2.82	0.00	-13.52	-13.52	2.80	0.00
Native	Central tooth Cusp	20	-0.62	0.00	-9.01	-9.01	-0.58	0.00
Native	Central tooth Stylus	20	3.21	0.00	-10.45	-10.45	3.20	0.00
Native	Lateral tooth I Cusp	20	1.20	0.00	0.00	0.00	1.21	0.00
Native	Lateral tooth I Stylus	20	5.11	0.00	14.08	14.08	5.10	0.00
Native	Lateral tooth II Basis	20	0.44	21.13	-3.39	12.50	1.99	31.00
Native	Lateral tooth II Cusp	20	4.65	10.06	2.85	10.60	4.64	22.37
Native	Lateral tooth II Stylus	20	6.77	2.26	24.80	13.35	6.74	-6.67
Native	Marginal tooth Basis	20	2.77	0.00	-26.98	-25.13	2.85	2.82
Native	Marginal tooth Cusp	20	2.98	0.00	-30.47	-29.10	2.98	0.00
Native	Marginal tooth Stylus	20	2.82	0.00	13.52	13.52	2.80	0.00
Native	Central tooth Cusp	21	8.38	0.00	11.59	11.59	8.54	0.00
Native	Central tooth Stylus	21	5.52	0.00	46.27	46.27	5.54	0.00
Native	Lateral tooth I Cusp	21	6.64	0.00	21.55	21.55	6.60	0.00
Native	Lateral tooth I Stylus	21	3.43	0.00	-22.82	-22.82	3.41	0.00
Native	Lateral tooth II Basis	21	2.48	2.82	-2.49	0.53	2.47	5.24
Native	Lateral tooth II Cusp	21	1.00	0.23	7.31	0.25	1.01	-21.56
Native	Lateral tooth II Stylus	21	4.79	11.85	1.10	7.00	6.14	-6.67
Native	Marginal tooth Basis	21	2.89	0.00	0.00	7.18	2.85	19.72
Native	Marginal tooth Cusp	21	2.98	0.00	0.00	11.94	2.98	61.54
Native	Marginal tooth Stylus	21	1.47	28.57	-13.52	-6.15	1.51	83.33
Native	Central tooth Cusp	22	3.99	0.00	-3.00	7.73	4.03	92.59
Native	Central tooth Stylus	22	5.89	0.00	-36.57	-30.60	5.91	34.78
Native	Lateral tooth I Cusp	22	7.61	0.00	-21.55	-16.16	6.58	61.54
Native	Lateral tooth I Stylus	22	8.54	0.00	-10.68	-0.97	8.47	100.00
Native	Lateral tooth II Basis	22	0.44	0.00	5.66	-10.21	0.43	-35.81
Native	Lateral tooth II Cusp	22	2.09	7.31	-27.12	-1.49	2.08	6.74
Native	Lateral tooth II Stylus	22	0.60	11.85	-15.38	5.95	-0.72	61.48
Native	Marginal tooth Basis	22	2.77	0.00	37.57	46.15	2.85	25.35
Native	Marginal tooth Cusp	22	2.98	0.00	42.97	37.31	2.98	-19.23
Native	Marginal tooth Stylus	22	4.10	-28.57	26.64	19.26	4.09	-83.33
Native	Central tooth Cusp	23	5.17	0.00	2.15	-8.58	5.30	-92.59
Native	Central tooth Stylus	23	8.58	23.64	11.57	10.45	8.53	-34.78
Native	Lateral tooth I Cusp	23	4.52	0.00	0.00	-4.38	5.56	-50.00
Native	Lateral tooth I Stylus	23	5.64	0.00	14.08	4.37	5.62	-100.00
Native	Lateral tooth II Basis	23	0.22	33.80	-2.04	9.15	0.26	15.72
Native	Lateral tooth II Cusp	23	3.96	6.78	4.64	5.39	3.99	-11.86
Native	Lateral tooth II Stylus	23	0.37	2.93	-29.04	-15.53	0.33	-22.96
Native	Marginal tooth Basis	23	8.54	0.00	-37.57	-54.36	8.54	-47.89
Native	Marginal tooth Cusp	23	4.87	0.00	-42.97	-43.28	4.87	-11.54
Native	Marginal tooth Stylus	23	9.37	0.00	10.66	10.66	9.34	0.00
Native	Central tooth Cusp	24	4.11	0.00	-13.30	-1.72	4.17	100.00
Native	Central tooth Stylus	24	-0.37	-23.64	-26.49	-29.85	-0.35	8.70
Native	Lateral tooth I Cusp	24	1.03	0.00	0.00	-1.01	1.04	-11.54
Native	Lateral tooth I Stylus	24	2.90	0.00	-27.67	-27.67	2.88	0.00
Native	Lateral tooth II Basis	24	1.05	21.13	18.78	26.06	1.04	21.83
Native	Lateral tooth II Cusp	24	3.03	4.70	11.78	9.41	3.02	26.42
Native	Lateral tooth II Stylus	24	3.96	13.98	12.56	11.18	3.98	-33.33
Native	Marginal tooth Basis	24	0.00	0.00	0.00	0.00	0.00	0.00

Native	Marginal tooth Cusp	24	4.64	0.00	0.00	-5.97	4.58	-30.77
Native	Marginal tooth Stylus	24	-3.74	0.00	10.25	10.25	-3.74	0.00
Native	Central tooth Cusp	25	0.11	0.00	91.42	79.83	0.13	-100.00
Native	Central tooth Stylus	25	8.13	7.27	97.76	97.76	8.15	-8.70
Native	Lateral tooth I Cusp	25	1.32	0.00	34.01	34.01	1.32	0.00
Native	Lateral tooth I Stylus	25	8.47	0.00	90.78	90.78	8.46	0.00
Native	Lateral tooth II Basis	25	-0.11	-76.06	-17.87	-32.04	-0.14	-21.40
Native	Lateral tooth II Cusp	25	3.68	17.00	3.39	13.18	3.67	-8.63
Native	Lateral tooth II Stylus	25	3.96	3.06	27.47	16.81	3.95	8.15
Native	Marginal tooth Basis	25	-12.76	0.00	0.00	0.00	-12.82	0.00
Native	Marginal tooth Cusp	25	1.43	0.00	45.31	43.28	1.48	0.00
Native	Marginal tooth Stylus	25	3.74	0.00	-20.90	-20.90	3.74	0.00
Native	Central tooth Cusp	26	2.92	0.00	-80.26	-80.26	2.96	0.00
Native	Central tooth Stylus	26	-4.10	-7.27	-97.76	-99.25	-4.12	0.00
Native	Lateral tooth I Cusp	26	5.27	0.00	-34.01	-34.01	5.25	0.00
Native	Lateral tooth I Stylus	26	-4.27	0.00	-50.49	-50.49	-4.28	0.00
Native	Lateral tooth II Basis	26	0.22	43.66	-3.39	1.76	0.22	-2.62
Native	Lateral tooth II Cusp	26	0.84	4.10	-25.51	-5.01	0.84	-9.43
Native	Lateral tooth II Stylus	26	-8.52	9.85	-27.16	-9.41	-8.51	-12.59
Native	Marginal tooth Basis	26	7.34	0.00	8.47	32.82	7.39	66.20
Native	Marginal tooth Cusp	26	0.00	0.00	-45.31	-43.28	0.00	0.00
Native	Marginal tooth Stylus	26	-1.47	0.00	-22.13	-22.13	-1.48	0.00
Native	Central tooth Cusp	27	6.13	0.00	-13.30	-13.30	6.27	0.00
Native	Central tooth Stylus	27	8.13	0.00	7.46	7.46	8.13	0.00
Native	Lateral tooth I Cusp	27	6.58	0.00	7.07	7.41	6.58	3.85
Native	Lateral tooth I Stylus	27	4.27	0.00	-7.28	-7.28	4.30	0.00
Native	Lateral tooth II Basis	27	0.00	-1.41	20.59	48.94	0.03	82.53
Native	Lateral tooth II Cusp	27	9.98	-2.58	0.98	-1.85	10.00	-1.62
Native	Lateral tooth II Stylus	27	-0.51	14.11	4.40	21.48	-1.96	97.04
Native	Marginal tooth Basis	27	-7.34	0.00	-8.47	-32.82	-7.34	-66.20
Native	Marginal tooth Cusp	27	-6.07	0.00	32.03	30.60	-6.07	0.00
Native	Marginal tooth Stylus	27	1.47	0.00	22.13	22.13	1.48	0.00
Native	Central tooth Cusp	28	10.85	0.00	-6.44	-6.44	11.05	0.00
Native	Central tooth Stylus	28	4.18	0.00	-7.46	-7.46	4.18	0.00
Native	Lateral tooth I Cusp	28	6.53	0.00	-7.07	-7.41	6.57	-3.85
Native	Lateral tooth I Stylus	28	8.54	0.00	-23.79	-23.79	8.47	0.00
Native	Lateral tooth II Basis	28	-2.10	18.31	4.30	-25.00	-2.09	-75.98
Native	Lateral tooth II Cusp	28	-0.44	11.48	-10.17	8.15	-0.44	23.72
Native	Lateral tooth II Stylus	28	0.05	2.80	-1.41	-5.15	1.49	-55.56
Native	Marginal tooth Basis	28	4.33	0.00	0.00	0.00	4.34	0.00
Native	Marginal tooth Cusp	28	4.64	0.00	10.94	10.45	4.58	0.00
Native	Marginal tooth Stylus	28	0.00	0.00	5.33	5.33	0.00	0.00
Native	Central tooth Cusp	29	6.69	0.00	40.77	40.77	6.82	0.00
Native	Central tooth Stylus	29	8.95	0.00	32.46	32.46	8.98	0.00
Native	Lateral tooth I Cusp	29	6.58	0.00	0.00	0.00	6.58	0.00
Native	Lateral tooth I Stylus	29	3.89	0.00	23.79	23.79	3.85	0.00
Native	Lateral tooth II Basis	29	-2.48	-11.27	19.23	26.41	-2.49	31.44
Native	Lateral tooth II Cusp	29	-3.31	0.56	5.98	0.18	-3.31	-20.75
Native	Lateral tooth II Stylus	29	3.04	0.13	16.48	6.28	3.05	-20.74
Native	Marginal tooth Basis	29	4.93	0.00	42.86	41.54	4.97	0.00
Native	Marginal tooth Cusp	29	1.43	0.00	55.47	52.99	1.48	0.00
Native	Marginal tooth Stylus	29	6.55	0.00	-18.03	-18.03	6.58	0.00
Native	Central tooth Cusp	30	0.17	0.00	-40.77	-40.77	0.20	0.00
Native	Central tooth Stylus	30	-3.73	67.27	-32.46	-18.66	-3.73	0.00
Native	Lateral tooth I Cusp	30	0.06	0.00	0.00	0.00	0.02	0.00
Native	Lateral tooth I Stylus	30	-3.81	0.00	9.71	9.71	-3.81	0.00
Native	Lateral tooth II Basis	30	-2.59	26.76	-23.08	-15.49	-2.61	-2.62
Native	Lateral tooth II Cusp	30	1.34	-13.20	-9.28	-12.90	1.35	-1.62
Native	Lateral tooth II Stylus	30	-9.17	-1.20	-14.13	-5.87	-9.18	19.26
Native	Marginal tooth Basis	30	6.98	0.00	-42.86	-5.13	6.93	100.00
Native	Marginal tooth Cusp	30	0.00	0.00	-98.44	-94.03	0.00	0.00
Native	Marginal tooth Stylus	30	-5.33	0.00	77.87	77.87	-5.36	0.00
Native	Central tooth Cusp	31	0.79	0.00	37.77	37.77	0.77	0.00
Native	Central tooth Stylus	31	2.98	-67.27	12.31	-1.49	2.94	0.00
Native	Lateral tooth I Cusp	31	6.58	0.00	0.00	0.00	6.58	0.00
Native	Lateral tooth I Stylus	31	8.47	0.00	3.40	3.40	8.46	0.00
Native	Lateral tooth II Basis	31	6.18	-8.45	-0.45	-20.25	6.17	-45.85
Native	Lateral tooth II Cusp	31	1.97	6.42	-8.39	4.53	1.94	21.02
Native	Lateral tooth II Stylus	31	6.26	-0.80	-1.41	2.33	6.26	32.59
Native	Marginal tooth Basis	31	-8.90	0.00	16.40	-20.51	-8.91	-100.00
Native	Marginal tooth Cusp	31	0.57	0.00	76.56	73.13	0.57	0.00
Native	Marginal tooth Stylus	31	-1.22	0.00	-34.43	-34.43	-1.22	0.00
Native	Central tooth Cusp	32	3.60	0.00	33.48	33.48	3.70	0.00
Native	Central tooth Stylus	32	4.03	0.00	-12.31	-12.31	4.07	0.00
Native	Lateral tooth I Cusp	32	-0.34	0.00	0.00	0.00	-0.32	0.00
Native	Lateral tooth I Stylus	32	4.27	0.00	-21.36	-21.36	4.23	0.00
Native	Lateral tooth II Basis	32	-4.69	23.94	1.36	7.04	-6.71	6.99
Native	Lateral tooth II Cusp	32	-7.67	-2.08	5.80	-2.30	-9.88	-24.80
Native	Lateral tooth II Stylus	32	-2.12	5.06	0.00	-4.83	-4.00	-73.33
Native	Marginal tooth Basis	32	-7.34	0.00	-16.40	-15.90	-7.34	0.00
Native	Marginal tooth Cusp	32	-2.75	0.00	-29.69	-28.36	-2.76	0.00
Native	Marginal tooth Stylus	32	0.00	0.00	-8.61	-8.61	0.00	0.00

Native	Central tooth Cusp	33	-0.45	0.00	-27.47	-27.47	-0.50	0.00
Native	Central tooth Stylus	33	-4.03	27.27	36.19	42.54	-4.04	4.35
Native	Lateral tooth I Cusp	33	-0.69	0.00	0.00	0.00	-0.67	0.00
Native	Lateral tooth I Stylus	33	-4.20	0.00	26.70	26.70	-4.19	0.00
Native	Lateral tooth II Basis	33	5.08	-66.20	0.45	-5.63	7.13	6.11
Native	Lateral tooth II Cusp	33	7.42	3.11	-8.74	5.21	9.65	56.60
Native	Lateral tooth II Stylus	33	2.21	-4.66	-26.69	-10.94	4.08	52.59
Native	Marginal tooth Basis	33	4.33	0.00	0.00	0.00	4.34	0.00
Native	Marginal tooth Cusp	33	1.20	0.00	-46.88	-44.78	-6.31	0.00
Native	Marginal tooth Stylus	33	0.43	0.00	-24.18	-24.18	0.46	0.00
Native	Central tooth Cusp	34	3.66	0.00	-21.46	-21.46	-0.48	0.00
Native	Central tooth Stylus	34	0.00	-27.27	63.81	57.46	0.01	-4.35
Native	Lateral tooth I Cusp	34	0.40	0.00	100.00	100.00	-0.65	0.00
Native	Lateral tooth I Stylus	34	0.15	0.00	26.70	26.70	0.73	0.00
Native	Lateral tooth II Basis	34	-2.70	-21.13	17.87	5.46	-2.73	-14.85
Native	Lateral tooth II Cusp	34	-6.64	-1.06	-0.62	-2.40	-8.89	-15.09
Native	Lateral tooth II Stylus	34	-1.61	14.91	1.88	5.15	-3.48	-44.44
Native	Marginal tooth Basis	34	4.93	0.00	62.96	61.03	4.97	0.00
Native	Marginal tooth Cusp	34	-0.46	0.00	42.97	41.04	-0.45	0.00
Native	Marginal tooth Stylus	34	-0.43	0.00	9.84	9.84	-0.46	0.00
Native	Central tooth Cusp	35	-2.59	0.00	36.91	36.91	-8.94	0.00
Native	Central tooth Stylus	35	-0.67	100.00	-25.00	-4.48	-0.69	0.00
Native	Lateral tooth I Cusp	35	-1.60	0.00	-51.18	-50.51	-2.67	7.69
Native	Lateral tooth I Stylus	35	-0.92	0.00	-21.36	-21.36	-1.42	0.00
Native	Lateral tooth II Basis	35	-1.82	1.41	-0.45	8.80	-1.82	22.27
Native	Lateral tooth II Cusp	35	7.74	2.12	5.80	2.56	9.96	-7.55
Native	Lateral tooth II Stylus	35	0.37	-38.48	10.52	-16.49	2.23	12.59
Native	Marginal tooth Basis	35	9.15	0.00	-62.96	-61.03	6.91	0.00
Native	Marginal tooth Cusp	35	-6.07	0.00	-42.97	-41.04	-5.60	0.00
Native	Marginal tooth Stylus	35	0.00	0.00	23.36	23.36	0.00	0.00
Native	Central tooth Cusp	36	-3.37	0.00	4.72	4.72	7.07	0.00
Native	Central tooth Stylus	36	9.69	-100.00	-51.12	-57.46	9.70	80.43
Native	Lateral tooth I Cusp	36	0.46	0.00	-48.82	-49.49	0.49	-7.69
Native	Lateral tooth I Stylus	36	2.90	0.00	18.45	18.45	2.83	0.00
Native	Lateral tooth II Basis	36	-0.55	4.23	-1.13	-10.56	-0.55	-24.89
Native	Lateral tooth II Cusp	36	-6.02	-2.45	2.23	0.99	-9.99	23.72
Native	Lateral tooth II Stylus	36	4.05	23.44	6.91	15.85	4.06	-17.78
Native	Marginal tooth Basis	36	-8.54	0.00	0.00	2.05	-17.51	5.63
Native	Marginal tooth Stylus	36	0.00	0.00	42.21	42.21	0.00	0.00
Native	Central tooth Cusp	37	4.11	0.00	-37.77	-37.77	4.20	0.00
Native	Central tooth Stylus	37	-6.34	0.00	10.82	-3.36	-6.34	-80.43
Native	Lateral tooth I Cusp	37	2.18	0.00	0.00	0.00	4.25	0.00
Native	Lateral tooth I Stylus	37	0.61	0.00	-31.07	-31.07	0.66	0.00
Native	Lateral tooth II Basis	37	0.00	-5.63	14.93	10.04	0.02	-2.62
Native	Lateral tooth II Cusp	37	-1.28	-2.48	3.48	2.35	0.49	34.77
Native	Lateral tooth II Stylus	37	1.38	-7.19	-1.10	-4.91	1.40	-0.74
Native	Marginal tooth Basis	37	-0.60	0.00	0.00	-2.05	10.60	-5.63
Native	Marginal tooth Cusp	37	1.43	0.00	0.00	0.00	1.37	0.00
Native	Marginal tooth Cusp	37	3.32	0.00	0.00	0.00	3.02	0.00
Native	Marginal tooth Stylus	37	0.00	0.00	-51.64	-51.64	0.00	0.00
Native	Central tooth Cusp	38	-1.52	0.00	58.37	58.37	-1.54	0.00
Native	Central tooth Stylus	38	0.82	0.00	10.82	10.82	0.83	0.00
Native	Lateral tooth I Cusp	38	-3.89	0.00	53.20	53.20	-2.01	0.00
Native	Lateral tooth I Stylus	38	2.29	0.00	49.03	49.03	2.80	0.00
Native	Lateral tooth II Basis	38	7.40	4.23	-0.90	0.00	7.41	0.00
Native	Lateral tooth II Cusp	38	3.03	14.65	-0.36	9.94	3.02	-12.40
Native	Lateral tooth II Stylus	38	2.72	10.12	11.77	12.63	2.71	5.19
Native	Marginal tooth Basis	38	8.66	0.00	100.00	100.00	8.61	8.45
Native	Marginal tooth Cusp	38	-3.32	0.00	0.00	0.00	-3.02	0.00
Native	Marginal tooth Stylus	38	0.43	64.29	24.18	31.56	0.46	0.00
Treated	Central tooth Cusp	1	0.29	0.00	0.00	0.00	0.30	0.00
Treated	Central tooth Stylus	1	0.72	0.00	0.00	0.00	0.74	0.00
Treated	Lateral tooth I Cusp	1	-0.91	100.00	0.00	29.17	-1.09	0.00
Treated	Lateral tooth I Stylus	1	0.40	0.00	0.00	0.00	-0.84	0.00
Treated	Lateral tooth II Basis	1	1.30	0.00	0.00	0.00	0.64	0.00
Treated	Lateral tooth II Cusp	1	3.53	0.00	0.00	0.00	6.59	0.00
Treated	Lateral tooth II Stylus	1	-0.56	0.10	0.00	0.09	-0.40	0.00
Treated	Marginal tooth Basis	1	3.02	0.00	0.00	0.00	3.10	0.00
Treated	Marginal tooth Cusp	1	-0.67	-100.00	0.00	-100.00	-0.68	0.00
Treated	Marginal tooth Stylus	1	1.91	0.00	0.00	0.00	1.58	0.00
Treated	Central tooth Cusp	2	0.29	0.00	0.00	0.00	0.31	0.00
Treated	Central tooth Stylus	2	0.52	0.00	0.00	0.00	0.51	0.00
Treated	Lateral tooth I Cusp	2	-0.98	-100.00	0.00	-29.17	0.71	0.00
Treated	Lateral tooth I Stylus	2	-0.30	0.00	0.00	0.00	-1.65	0.00
Treated	Lateral tooth II Basis	2	10.11	0.00	0.00	0.00	5.12	0.00
Treated	Lateral tooth II Cusp	2	3.99	0.00	0.00	0.00	1.60	0.00
Treated	Lateral tooth II Stylus	2	0.32	-0.10	0.00	-0.09	0.28	0.00
Treated	Marginal tooth Basis	2	3.02	0.00	0.00	0.00	2.30	0.00
Treated	Marginal tooth Cusp	2	2.19	0.00	0.00	0.00	2.01	0.00
Treated	Marginal tooth Stylus	2	1.61	100.00	0.00	80.00	1.28	0.00
Treated	Central tooth Cusp	3	1.17	100.00	0.00	40.00	1.22	0.00
Treated	Central tooth Stylus	3	0.10	37.14	0.00	57.14	0.12	100.00

Treated	Lateral tooth I Cusp	3	0.68	0.00	0.00	0.00	0.06	0.00
Treated	Lateral tooth I Stylus	3	0.30	0.00	0.00	0.00	0.42	0.00
Treated	Lateral tooth II Basis	3	-0.74	0.00	0.00	0.00	-0.38	0.00
Treated	Lateral tooth II Cusp	3	-0.97	0.00	5.00	0.09	-0.66	0.00
Treated	Lateral tooth II Stylus	3	0.56	0.00	0.00	0.00	0.56	0.00
Treated	Marginal tooth Basis	3	8.35	0.00	0.00	0.00	9.52	0.00
Treated	Marginal tooth Cusp	3	-0.48	0.00	0.00	0.00	-0.49	0.00
Treated	Marginal tooth Stylus	3	1.31	-100.00	0.00	-80.00	1.06	0.00
Treated	Central tooth Cusp	4	-0.73	-100.00	0.00	-40.00	-0.75	0.00
Treated	Central tooth Stylus	4	-0.93	-37.14	0.00	-57.14	-1.00	-100.00
Treated	Lateral tooth I Cusp	4	0.00	71.43	0.00	20.83	1.14	0.00
Treated	Lateral tooth I Stylus	4	0.40	0.00	0.00	0.00	0.42	0.00
Treated	Lateral tooth II Basis	4	2.97	0.00	0.00	0.00	1.50	0.00
Treated	Lateral tooth II Cusp	4	3.13	0.00	8.33	0.22	3.39	100.00
Treated	Lateral tooth II Stylus	4	-5.85	0.00	0.00	0.09	-5.79	33.33
Treated	Marginal tooth Basis	4	-0.36	0.00	0.00	0.00	-0.30	0.00
Treated	Marginal tooth Cusp	4	0.67	0.00	0.00	0.00	0.62	0.00
Treated	Marginal tooth Stylus	4	-1.21	100.00	0.00	85.71	-0.96	100.00
Treated	Central tooth Cusp	5	-0.51	0.00	2.00	2.00	-0.55	0.00
Treated	Central tooth Stylus	5	0.52	0.00	0.00	0.00	0.61	0.00
Treated	Lateral tooth I Cusp	5	1.06	-71.43	0.00	-20.83	-1.29	0.00
Treated	Lateral tooth I Stylus	5	-0.60	0.00	0.00	0.00	-0.58	0.00
Treated	Lateral tooth II Basis	5	9.18	0.00	0.00	0.00	4.64	0.00
Treated	Lateral tooth II Cusp	5	6.42	1.25	38.33	1.90	6.95	-100.00
Treated	Lateral tooth II Stylus	5	3.69	0.00	5.04	0.55	3.67	-33.33
Treated	Marginal tooth Basis	5	8.35	0.00	11.54	11.54	8.55	0.00
Treated	Marginal tooth Cusp	5	3.90	0.00	0.00	0.00	3.64	0.00
Treated	Marginal tooth Stylus	5	0.00	-100.00	11.43	-74.29	-0.05	-100.00
Treated	Central tooth Cusp	6	0.59	0.00	16.00	16.00	0.61	0.00
Treated	Central tooth Stylus	6	0.21	0.00	0.00	0.00	0.24	0.00
Treated	Lateral tooth I Cusp	6	-1.21	0.00	0.00	0.00	-0.30	0.00
Treated	Lateral tooth I Stylus	6	0.60	0.00	0.00	0.00	0.60	0.00
Treated	Lateral tooth II Basis	6	9.93	18.31	0.00	17.57	4.98	0.00
Treated	Lateral tooth II Cusp	6	3.41	0.47	-28.33	-0.06	0.86	0.00
Treated	Lateral tooth II Stylus	6	1.60	1.57	1.44	1.66	1.56	0.00
Treated	Marginal tooth Basis	6	-4.09	0.00	-11.54	-11.54	-4.24	0.00
Treated	Marginal tooth Cusp	6	2.76	0.00	0.00	0.00	2.53	0.00
Treated	Marginal tooth Stylus	6	6.73	0.00	-11.43	-11.43	5.44	0.00
Treated	Central tooth Cusp	7	0.07	0.00	0.00	0.00	0.07	0.00
Treated	Central tooth Stylus	7	0.83	0.00	0.00	0.00	0.85	0.00
Treated	Lateral tooth I Cusp	7	0.30	0.00	0.00	0.00	0.07	0.00
Treated	Lateral tooth I Stylus	7	0.30	100.00	0.00	46.43	0.35	0.00
Treated	Lateral tooth II Basis	7	8.81	9.86	29.63	31.08	4.46	0.00
Treated	Lateral tooth II Cusp	7	2.07	5.81	-23.33	5.36	2.00	0.00
Treated	Lateral tooth II Stylus	7	4.01	1.08	-2.88	0.65	4.00	0.00
Treated	Marginal tooth Basis	7	7.10	0.00	0.00	0.00	7.36	0.00
Treated	Marginal tooth Cusp	7	0.95	0.00	0.00	0.00	6.66	0.00
Treated	Marginal tooth Stylus	7	0.10	0.00	0.00	0.00	0.10	0.00
Treated	Central tooth Cusp	8	0.00	0.00	0.00	0.00	0.06	0.00
Treated	Central tooth Cusp	8	0.07	0.00	-18.00	-18.00	-0.68	0.00
Treated	Central tooth Stylus	8	0.21	0.00	0.00	0.00	0.15	0.00
Treated	Lateral tooth I Cusp	8	0.45	0.00	0.00	8.33	-0.51	28.57
Treated	Lateral tooth I Stylus	8	0.60	-100.00	0.00	-46.43	0.61	0.00
Treated	Lateral tooth II Basis	8	5.10	-9.86	25.93	9.46	5.54	0.00
Treated	Lateral tooth II Cusp	8	4.26	8.24	0.00	8.23	4.10	0.00
Treated	Lateral tooth II Stylus	8	1.28	-0.98	-3.60	-1.39	1.32	0.00
Treated	Marginal tooth Basis	8	5.15	0.00	0.00	0.00	5.41	0.00
Treated	Marginal tooth Cusp	8	3.23	0.00	0.00	0.00	4.18	0.00
Treated	Marginal tooth Stylus	8	4.12	0.00	8.57	8.57	3.23	0.00
Treated	Central tooth Cusp	9	-0.66	0.00	0.00	0.00	0.51	0.00
Treated	Central tooth Stylus	9	-0.93	0.00	0.00	0.00	-0.97	0.00
Treated	Lateral tooth I Cusp	9	-0.53	100.00	0.00	20.83	-3.98	-28.57
Treated	Lateral tooth I Stylus	9	-0.70	0.00	0.00	0.00	-0.78	0.00
Treated	Lateral tooth II Basis	9	-13.82	-5.63	-55.56	-45.95	4.09	0.00
Treated	Lateral tooth II Cusp	9	1.58	1.34	0.00	1.34	1.53	0.00
Treated	Lateral tooth II Stylus	9	5.29	0.10	0.00	0.09	5.29	0.00
Treated	Marginal tooth Basis	9	-3.02	0.00	0.00	0.00	-3.56	0.00
Treated	Marginal tooth Cusp	9	6.18	0.00	0.00	0.00	5.79	0.00
Treated	Marginal tooth Stylus	9	3.02	0.00	-8.57	-8.57	2.47	0.00
Treated	Central tooth Cusp	10	0.51	0.00	0.00	0.00	0.20	0.00
Treated	Central tooth Stylus	10	0.21	0.00	15.49	13.10	0.29	0.00
Treated	Lateral tooth I Cusp	10	-3.77	-100.00	0.00	-29.17	1.09	0.00
Treated	Lateral tooth I Stylus	10	0.60	76.92	0.00	35.71	0.71	0.00
Treated	Lateral tooth II Basis	10	0.00	28.17	24.07	48.65	3.11	18.75
Treated	Lateral tooth II Cusp	10	3.50	2.22	0.00	2.21	3.41	0.00
Treated	Lateral tooth II Stylus	10	4.17	4.11	0.00	3.88	4.17	0.00
Treated	Marginal tooth Basis	10	-17.76	0.00	0.00	0.00	-18.13	0.00
Treated	Marginal tooth Cusp	10	7.60	0.00	0.00	0.00	6.50	0.00
Treated	Marginal tooth Stylus	10	1.21	0.00	0.00	0.00	0.95	0.00
Treated	Central tooth Cusp	11	0.15	0.00	0.00	0.00	0.33	0.00
Treated	Central tooth Stylus	11	0.72	0.00	-2.82	1.19	0.74	8.57
Treated	Lateral tooth I Cusp	11	1.06	85.71	0.00	25.00	-1.01	0.00

Treated	Lateral tooth I Stylus	11	0.30	-76.92	0.00	-35.71	0.26	0.00
Treated	Lateral tooth II Basis	11	-3.71	-26.76	64.81	16.22	2.04	-18.75
Treated	Lateral tooth II Cusp	11	0.97	4.03	0.00	4.02	0.93	0.00
Treated	Lateral tooth II Stylus	11	1.36	-0.49	0.00	-0.46	1.38	0.00
Treated	Marginal tooth Basis	11	2.84	0.00	0.00	0.00	3.10	0.00
Treated	Marginal tooth Cusp	11	5.99	0.00	0.00	0.00	6.35	0.00
Treated	Marginal tooth Stylus	11	1.01	0.00	20.00	20.00	0.82	0.00
Treated	Central tooth Cusp	12	0.37	0.00	8.00	8.00	0.42	0.00
Treated	Central tooth Stylus	12	-0.21	0.00	9.86	4.76	-0.21	-8.57
Treated	Lateral tooth I Cusp	12	-0.98	-85.71	0.00	-25.00	-0.38	0.00
Treated	Lateral tooth I Stylus	12	-0.81	0.00	0.00	0.00	-0.86	0.00
Treated	Lateral tooth II Basis	12	2.32	-9.86	-88.89	-72.97	8.94	0.00
Treated	Lateral tooth II Cusp	12	4.26	5.59	56.67	6.64	4.12	0.00
Treated	Lateral tooth II Stylus	12	5.21	-3.91	0.00	-3.69	5.76	0.00
Treated	Marginal tooth Basis	12	3.73	0.00	0.00	0.00	3.73	0.00
Treated	Marginal tooth Cusp	12	8.56	0.00	73.53	60.98	8.07	0.00
Treated	Marginal tooth Stylus	12	8.54	0.00	11.43	11.43	6.80	0.00
Treated	Central tooth Cusp	13	0.44	0.00	-8.00	-8.00	-0.42	0.00
Treated	Central tooth Stylus	13	-0.62	0.00	-22.54	-19.05	-0.65	0.00
Treated	Lateral tooth I Cusp	13	-0.38	0.00	0.00	0.00	0.17	0.00
Treated	Lateral tooth I Stylus	13	0.50	0.00	0.00	7.14	0.51	14.29
Treated	Lateral tooth II Basis	13	2.04	1.41	0.00	1.35	14.64	0.00
Treated	Lateral tooth II Cusp	13	3.35	0.87	43.33	1.68	3.22	0.00
Treated	Lateral tooth II Stylus	13	8.26	4.01	0.00	3.97	7.71	66.67
Treated	Marginal tooth Basis	13	0.00	0.00	0.00	0.00	0.15	0.00
Treated	Marginal tooth Cusp	13	7.13	0.00	-73.53	-60.98	8.19	0.00
Treated	Marginal tooth Stylus	13	8.44	0.00	-31.43	-28.57	6.80	50.00
Treated	Central tooth Cusp	14	-0.29	0.00	0.00	0.00	5.13	0.00
Treated	Central tooth Stylus	14	0.10	0.00	0.00	0.00	0.09	0.00
Treated	Lateral tooth I Cusp	14	0.15	0.00	35.29	33.33	4.95	28.57
Treated	Lateral tooth I Stylus	14	0.00	0.00	100.00	92.86	0.00	71.43
Treated	Lateral tooth II Basis	14	7.88	4.23	0.00	4.05	9.76	0.00
Treated	Lateral tooth II Cusp	14	1.46	2.06	-53.33	1.06	1.42	0.00
Treated	Lateral tooth II Stylus	14	2.97	-0.20	41.01	5.17	3.00	0.00
Treated	Marginal tooth Basis	14	2.49	0.00	0.00	0.00	1.69	0.00
Treated	Marginal tooth Cusp	14	7.22	0.00	44.12	39.02	9.85	100.00
Treated	Marginal tooth Stylus	14	10.75	0.00	8.57	5.71	8.57	-50.00
Treated	Central tooth Cusp	15	5.12	0.00	0.00	0.00	6.88	100.00
Treated	Central tooth Stylus	15	7.14	20.00	0.00	8.33	7.37	0.00
Treated	Lateral tooth I Cusp	15	4.75	0.00	-35.29	-33.33	6.64	-28.57
Treated	Lateral tooth I Stylus	15	7.65	0.00	-100.00	-100.00	7.93	-85.71
Treated	Lateral tooth II Basis	15	-6.96	52.11	0.00	50.00	0.98	0.00
Treated	Lateral tooth II Cusp	15	5.08	9.86	-46.67	8.98	4.90	0.00
Treated	Lateral tooth II Stylus	15	7.38	3.13	58.99	10.34	7.36	-33.33
Treated	Marginal tooth Basis	15	3.55	0.00	34.62	34.62	4.54	0.00
Treated	Marginal tooth Cusp	15	4.85	0.00	-35.29	-31.71	4.89	-100.00
Treated	Marginal tooth Stylus	15	4.32	0.00	31.43	31.43	3.45	0.00
Treated	Central tooth Cusp	16	6.80	0.00	0.00	10.00	5.75	-100.00
Treated	Central tooth Stylus	16	-0.83	-20.00	0.00	-8.33	-0.78	0.00
Treated	Lateral tooth I Cusp	16	6.34	14.29	0.00	4.17	6.73	0.00
Treated	Lateral tooth I Stylus	16	0.30	0.00	0.00	0.00	0.58	0.00
Treated	Lateral tooth II Basis	16	-4.64	-23.94	0.00	-22.97	3.35	0.00
Treated	Lateral tooth II Cusp	16	3.26	-3.18	0.00	-3.18	2.34	0.00
Treated	Lateral tooth II Stylus	16	6.26	-2.84	-94.96	-14.96	6.23	-33.33
Treated	Marginal tooth Basis	16	7.10	0.00	-34.62	-34.62	7.42	0.00
Treated	Marginal tooth Cusp	16	2.66	0.00	-8.82	-7.32	2.68	0.00
Treated	Marginal tooth Stylus	16	4.12	0.00	-34.29	-34.29	3.31	0.00
Treated	Central tooth Cusp	17	5.71	0.00	0.00	-10.00	6.71	0.00
Treated	Central tooth Stylus	17	9.72	0.00	0.00	0.00	10.06	0.00
Treated	Lateral tooth I Cusp	17	6.49	-14.29	0.00	-4.17	6.81	0.00
Treated	Lateral tooth I Stylus	17	10.57	0.00	0.00	0.00	11.23	0.00
Treated	Lateral tooth II Basis	17	9.37	-33.80	0.00	-29.73	1.04	12.50
Treated	Lateral tooth II Cusp	17	1.40	7.46	0.00	7.45	2.18	0.00
Treated	Lateral tooth II Stylus	17	6.26	0.20	-5.04	-0.46	6.28	0.00
Treated	Marginal tooth Basis	17	3.20	0.00	0.00	0.00	3.24	0.00
Treated	Marginal tooth Cusp	17	4.09	0.00	0.00	0.00	4.10	0.00
Treated	Marginal tooth Stylus	17	4.22	0.00	25.71	25.71	3.37	0.00
Treated	Central tooth Cusp	18	6.66	0.00	0.00	0.00	7.35	0.00
Treated	Central tooth Stylus	18	4.65	0.00	0.00	0.00	4.83	0.00
Treated	Lateral tooth I Cusp	18	6.57	0.00	0.00	0.00	5.65	0.00
Treated	Lateral tooth I Stylus	18	7.55	0.00	0.00	50.00	7.96	100.00
Treated	Lateral tooth II Basis	18	3.25	25.35	0.00	21.62	5.86	-12.50
Treated	Lateral tooth II Cusp	18	1.70	-7.96	0.00	-7.95	1.64	0.00
Treated	Lateral tooth II Stylus	18	5.29	1.86	0.00	1.75	5.28	0.00
Treated	Marginal tooth Basis	18	-1.07	0.00	0.00	0.00	-1.02	0.00
Treated	Marginal tooth Cusp	18	4.18	0.00	0.00	0.00	4.13	0.00
Treated	Marginal tooth Stylus	18	4.22	0.00	-31.43	-31.43	3.42	0.00
Treated	Central tooth Cusp	19	7.32	0.00	12.00	12.00	-0.48	0.00
Treated	Central tooth Stylus	19	4.34	0.00	0.00	0.00	4.57	0.00
Treated	Lateral tooth I Cusp	19	5.36	0.00	0.00	0.00	0.97	0.00
Treated	Lateral tooth I Stylus	19	-0.70	0.00	0.00	-50.00	-0.88	-100.00
Treated	Lateral tooth II Basis	19	2.41	-25.35	25.93	16.22	-0.46	100.00

Treated	Lateral tooth II Cusp	19	1.67	-2.62	0.00	-2.62	1.64	0.00
Treated	Lateral tooth II Stylus	19	3.13	2.74	0.00	2.59	3.14	0.00
Treated	Marginal tooth Basis	19	3.20	0.00	53.85	53.85	3.28	0.00
Treated	Marginal tooth Cusp	19	4.18	0.00	14.71	12.20	4.17	0.00
Treated	Marginal tooth Stylus	19	-16.88	0.00	0.00	0.00	3.48	0.00
Treated	Central tooth Cusp	20	-0.51	0.00	-12.00	-12.00	9.18	0.00
Treated	Central tooth Stylus	20	3.41	0.00	0.00	0.00	3.53	0.00
Treated	Lateral tooth I Cusp	20	0.98	28.57	0.00	8.33	7.52	0.00
Treated	Lateral tooth I Stylus	20	-2.92	0.00	0.00	0.00	-2.96	0.00
Treated	Lateral tooth II Basis	20	2.50	22.54	-25.93	-18.92	0.62	-100.00
Treated	Lateral tooth II Cusp	20	4.20	10.05	0.00	10.04	4.05	0.00
Treated	Lateral tooth II Stylus	20	6.98	1.66	0.00	1.57	6.98	0.00
Treated	Marginal tooth Basis	20	3.20	0.00	-53.85	-53.85	3.31	0.00
Treated	Marginal tooth Cusp	20	-19.20	0.00	-14.71	-12.20	-19.17	0.00
Treated	Marginal tooth Stylus	20	2.11	0.00	0.00	0.00	3.54	0.00
Treated	Central tooth Cusp	21	9.14	0.00	0.00	0.00	4.44	0.00
Treated	Central tooth Stylus	21	5.58	0.00	0.00	0.00	5.89	0.00
Treated	Lateral tooth I Cusp	21	7.17	-28.57	100.00	91.67	7.05	100.00
Treated	Lateral tooth I Stylus	21	3.63	0.00	0.00	0.00	3.80	0.00
Treated	Lateral tooth II Basis	21	-4.82	2.82	0.00	2.70	2.89	0.00
Treated	Lateral tooth II Cusp	21	1.19	0.25	0.00	0.25	1.14	0.00
Treated	Lateral tooth II Stylus	21	6.17	13.11	0.00	12.37	4.82	0.00
Treated	Marginal tooth Basis	21	3.73	0.00	0.00	0.00	3.34	0.00
Treated	Marginal tooth Cusp	21	3.42	0.00	0.00	0.00	3.39	0.00
Treated	Marginal tooth Stylus	21	-3.82	21.43	0.00	17.14	3.60	0.00
Treated	Central tooth Cusp	22	4.39	0.00	0.00	0.00	5.82	0.00
Treated	Central tooth Stylus	22	6.41	0.00	0.00	0.00	6.65	0.00
Treated	Lateral tooth I Cusp	22	7.77	0.00	-100.00	-100.00	6.08	-100.00
Treated	Lateral tooth I Stylus	22	8.56	0.00	0.00	14.29	9.09	28.57
Treated	Lateral tooth II Basis	22	7.51	0.00	0.00	0.00	0.88	0.00
Treated	Lateral tooth II Cusp	22	2.07	7.33	0.00	7.33	2.01	0.00
Treated	Lateral tooth II Stylus	22	0.08	36.11	0.00	34.07	1.41	0.00
Treated	Marginal tooth Basis	22	4.09	0.00	65.38	65.38	3.38	0.00
Treated	Marginal tooth Cusp	22	3.42	0.00	20.59	17.07	3.43	0.00
Treated	Marginal tooth Stylus	22	0.80	-21.43	31.43	14.29	2.34	0.00
Treated	Central tooth Cusp	23	10.39	0.00	0.00	0.00	4.64	0.00
Treated	Central tooth Stylus	23	32.68	37.14	100.00	100.00	9.33	0.00
Treated	Lateral tooth I Cusp	23	4.83	0.00	0.00	0.00	1.45	0.00
Treated	Lateral tooth I Stylus	23	5.84	0.00	0.00	-14.29	6.22	-28.57
Treated	Lateral tooth II Basis	23	0.00	32.39	0.00	31.08	-0.35	0.00
Treated	Lateral tooth II Cusp	23	3.74	6.77	0.00	6.76	3.61	0.00
Treated	Lateral tooth II Stylus	23	0.80	2.15	0.00	2.03	0.81	0.00
Treated	Marginal tooth Basis	23	8.35	0.00	-65.38	-65.38	9.25	0.00
Treated	Marginal tooth Cusp	23	5.32	0.00	-20.59	-17.07	5.33	0.00
Treated	Marginal tooth Stylus	23	2.71	0.00	-17.14	-17.14	11.77	0.00
Treated	Central tooth Stylus	24	-23.89	-37.14	-100.00	-100.00	-0.19	0.00
Treated	Lateral tooth I Cusp	24	1.36	0.00	0.00	0.00	0.39	0.00
Treated	Lateral tooth I Stylus	24	3.22	0.00	0.00	0.00	3.42	0.00
Treated	Lateral tooth II Basis	24	3.43	22.54	12.96	31.08	2.58	0.00
Treated	Lateral tooth II Cusp	24	2.92	4.74	0.00	4.74	2.84	0.00
Treated	Lateral tooth II Stylus	24	4.49	8.71	0.00	8.22	4.44	0.00
Treated	Marginal tooth Basis	24	-1.60	0.00	0.00	0.00	0.54	0.00
Treated	Marginal tooth Cusp	24	5.04	0.00	0.00	0.00	5.10	0.00
Treated	Marginal tooth Stylus	24	-1.41	0.00	-14.29	-14.29	-2.97	0.00
Treated	Central tooth Cusp	25	2.49	0.00	0.00	0.00	0.34	0.00
Treated	Central tooth Stylus	25	11.38	0.00	84.51	71.43	8.99	0.00
Treated	Lateral tooth I Cusp	25	1.51	0.00	76.47	54.17	7.27	0.00
Treated	Lateral tooth I Stylus	25	10.17	0.00	0.00	0.00	9.34	0.00
Treated	Lateral tooth II Basis	25	0.56	-76.06	70.37	-21.62	0.32	0.00
Treated	Lateral tooth II Cusp	25	3.53	17.04	0.00	17.02	3.40	0.00
Treated	Lateral tooth II Stylus	25	4.41	-22.50	0.00	-21.24	4.45	0.00
Treated	Marginal tooth Basis	25	-12.26	0.00	0.00	0.00	-12.76	0.00
Treated	Marginal tooth Cusp	25	2.09	0.00	23.53	19.51	2.05	0.00
Treated	Marginal tooth Stylus	25	2.51	0.00	0.00	0.00	4.81	0.00
Treated	Central tooth Cusp	26	3.37	0.00	0.00	0.00	3.38	0.00
Treated	Central tooth Stylus	26	1.34	0.00	-84.51	-71.43	-4.27	0.00
Treated	Lateral tooth I Cusp	26	7.02	0.00	-76.47	-54.17	6.42	0.00
Treated	Lateral tooth I Stylus	26	-2.42	0.00	0.00	0.00	-4.06	0.00
Treated	Lateral tooth II Basis	26	-3.62	42.25	-83.33	-20.27	0.69	0.00
Treated	Lateral tooth II Cusp	26	1.22	4.53	0.00	4.52	0.24	0.00
Treated	Lateral tooth II Stylus	26	-8.02	50.39	0.00	47.55	-8.03	0.00
Treated	Marginal tooth Basis	26	7.10	0.00	0.00	0.00	8.99	0.00
Treated	Marginal tooth Cusp	26	0.57	0.00	-23.53	-19.51	0.58	0.00
Treated	Marginal tooth Stylus	26	-3.62	0.00	0.00	0.00	0.93	0.00
Treated	Central tooth Cusp	27	4.75	0.00	0.00	0.00	6.97	0.00
Treated	Central tooth Stylus	27	3.52	0.00	5.63	4.76	9.31	0.00
Treated	Lateral tooth I Cusp	27	4.98	0.00	0.00	0.00	-0.81	0.00
Treated	Lateral tooth I Stylus	27	1.91	0.00	0.00	0.00	5.03	0.00
Treated	Lateral tooth II Basis	27	11.60	0.00	0.00	0.00	0.50	0.00
Treated	Lateral tooth II Cusp	27	8.55	-3.00	41.67	-2.15	9.59	100.00
Treated	Lateral tooth II Stylus	27	-0.48	-8.32	0.00	-7.85	-0.51	0.00
Treated	Marginal tooth Basis	27	-6.22	0.00	0.00	0.00	-8.00	0.00

Treated	Marginal tooth Cusp	27	-5.51	0.00	32.35	26.83	-5.56	0.00
Treated	Marginal tooth Stylus	27	0.00	0.00	0.00	0.00	0.93	0.00
Treated	Central tooth Cusp	28	2.71	0.00	0.00	0.00	2.75	0.00
Treated	Central tooth Stylus	28	-4.65	0.00	-5.63	-4.76	-4.54	0.00
Treated	Lateral tooth I Cusp	28	-0.75	0.00	0.00	0.00	6.76	0.00
Treated	Lateral tooth I Stylus	28	0.30	0.00	0.00	0.00	0.35	0.00
Treated	Lateral tooth II Basis	28	11.69	16.90	0.00	22.97	-11.50	25.00
Treated	Lateral tooth II Cusp	28	1.89	11.49	33.33	12.03	1.56	-100.00
Treated	Lateral tooth II Stylus	28	-8.26	6.07	15.83	7.76	-8.26	0.00
Treated	Marginal tooth Basis	28	-3.73	0.00	0.00	0.00	-4.06	0.00
Treated	Marginal tooth Cusp	28	-4.75	0.00	11.76	9.76	-4.76	0.00
Treated	Marginal tooth Stylus	28	-0.80	0.00	0.00	0.00	-9.15	0.00
Treated	Central tooth Cusp	29	6.88	0.00	16.00	16.00	6.88	0.00
Treated	Central tooth Stylus	29	6.20	37.14	0.00	15.48	9.03	0.00
Treated	Lateral tooth I Cusp	29	7.09	0.00	0.00	0.00	0.50	0.00
Treated	Lateral tooth I Stylus	29	4.03	0.00	0.00	0.00	4.20	0.00
Treated	Lateral tooth II Basis	29	-6.68	-9.86	0.00	-16.22	-12.16	-25.00
Treated	Lateral tooth II Cusp	29	7.82	0.56	-58.33	-0.53	7.91	0.00
Treated	Lateral tooth II Stylus	29	3.21	-20.74	83.45	-8.86	3.23	0.00
Treated	Marginal tooth Basis	29	8.53	0.00	0.00	0.00	5.19	0.00
Treated	Marginal tooth Cusp	29	1.81	0.00	55.88	46.34	1.88	0.00
Treated	Marginal tooth Stylus	29	7.94	0.00	0.00	0.00	3.52	0.00
Treated	Central tooth Cusp	30	0.44	0.00	-16.00	-16.00	0.45	0.00
Treated	Central tooth Stylus	30	-3.31	-37.14	0.00	-15.48	-3.45	0.00
Treated	Lateral tooth I Cusp	30	3.55	0.00	0.00	0.00	6.85	0.00
Treated	Lateral tooth I Stylus	30	-3.02	0.00	0.00	0.00	-3.23	0.00
Treated	Lateral tooth II Basis	30	-3.90	26.76	0.00	44.59	-10.81	81.25
Treated	Lateral tooth II Cusp	30	-1.49	-13.23	31.67	-12.66	1.77	0.00
Treated	Lateral tooth II Stylus	30	-8.02	9.88	-67.63	0.65	-8.04	0.00
Treated	Marginal tooth Basis	30	9.06	0.00	0.00	0.00	7.12	0.00
Treated	Marginal tooth Cusp	30	0.29	0.00	-100.00	-82.93	-1.29	0.00
Treated	Marginal tooth Stylus	30	8.84	0.00	0.00	0.00	-24.09	0.00
Treated	Central tooth Cusp	31	1.02	0.00	0.00	0.00	1.01	0.00
Treated	Central tooth Stylus	31	3.00	0.00	0.00	0.00	3.12	0.00
Treated	Lateral tooth I Cusp	31	6.87	0.00	0.00	0.00	0.20	0.00
Treated	Lateral tooth I Stylus	31	8.26	0.00	0.00	0.00	8.77	0.00
Treated	Lateral tooth II Basis	31	12.62	-5.63	0.00	-21.62	4.08	-68.75
Treated	Lateral tooth II Cusp	31	-1.40	6.43	-48.33	5.55	2.37	0.00
Treated	Lateral tooth II Stylus	31	7.54	8.32	47.48	13.94	6.32	0.00
Treated	Marginal tooth Basis	31	-8.17	0.00	0.00	0.00	-8.08	0.00
Treated	Marginal tooth Cusp	31	1.33	0.00	64.71	53.66	2.86	0.00
Treated	Marginal tooth Stylus	31	-2.61	0.00	0.00	0.00	-0.73	0.00
Treated	Central tooth Cusp	32	3.80	0.00	0.00	0.00	3.89	0.00
Treated	Central tooth Stylus	32	6.51	0.00	0.00	0.00	4.26	0.00
Treated	Lateral tooth I Cusp	32	0.15	0.00	0.00	0.00	-0.15	0.00
Treated	Lateral tooth I Stylus	32	8.16	0.00	0.00	0.00	4.69	0.00
Treated	Lateral tooth II Basis	32	-12.80	21.13	0.00	17.57	-3.69	-12.50
Treated	Lateral tooth II Cusp	32	-7.36	-2.09	0.00	-2.09	-9.28	0.00
Treated	Lateral tooth II Stylus	32	-2.89	2.35	-25.18	-1.02	-3.42	0.00
Treated	Marginal tooth Basis	32	-6.75	0.00	0.00	0.00	-8.22	0.00
Treated	Marginal tooth Cusp	32	-2.09	0.00	-41.18	-34.15	-2.06	0.00
Treated	Marginal tooth Stylus	32	3.42	0.00	0.00	0.00	0.13	0.00
Treated	Central tooth Cusp	33	-0.22	0.00	0.00	0.00	-0.23	0.00
Treated	Central tooth Stylus	33	-1.14	42.86	0.00	20.24	-3.78	5.71
Treated	Lateral tooth I Cusp	33	-0.15	0.00	0.00	0.00	-1.55	0.00
Treated	Lateral tooth I Stylus	33	-1.21	0.00	0.00	0.00	-3.50	0.00
Treated	Lateral tooth II Basis	33	7.79	-66.20	0.00	-63.51	5.61	0.00
Treated	Lateral tooth II Cusp	33	8.03	3.12	0.00	3.12	9.98	0.00
Treated	Lateral tooth II Stylus	33	2.49	-23.58	-12.23	-23.82	4.24	0.00
Treated	Marginal tooth Basis	33	12.97	0.00	0.00	0.00	6.22	0.00
Treated	Marginal tooth Cusp	33	1.62	0.00	-23.53	-19.51	-5.44	0.00
Treated	Marginal tooth Stylus	33	6.33	0.00	0.00	0.00	1.18	0.00
Treated	Central tooth Cusp	34	3.95	0.00	0.00	0.00	-0.21	0.00
Treated	Central tooth Stylus	34	2.79	-31.43	0.00	-15.48	0.24	-5.71
Treated	Lateral tooth I Cusp	34	-0.30	0.00	0.00	0.00	-0.72	0.00
Treated	Lateral tooth I Stylus	34	0.50	0.00	0.00	0.00	0.96	0.00
Treated	Lateral tooth II Basis	34	-1.39	-22.54	0.00	-21.62	-1.59	0.00
Treated	Lateral tooth II Cusp	34	-2.07	-1.06	0.00	-1.06	-8.38	0.00
Treated	Lateral tooth II Stylus	34	-1.12	21.04	2.16	20.04	-2.85	0.00
Treated	Marginal tooth Basis	34	5.15	0.00	0.00	0.00	5.31	0.00
Treated	Marginal tooth Cusp	34	0.10	0.00	20.59	17.07	0.05	0.00
Treated	Marginal tooth Stylus	34	0.60	0.00	0.00	0.00	0.47	0.00
Treated	Central tooth Cusp	35	-2.34	0.00	74.00	74.00	-8.60	0.00
Treated	Central tooth Stylus	35	-0.72	-11.43	73.24	57.14	-0.73	0.00
Treated	Lateral tooth I Cusp	35	0.15	0.00	0.00	0.00	0.97	0.00
Treated	Lateral tooth I Stylus	35	-0.70	0.00	0.00	0.00	-0.74	0.00
Treated	Lateral tooth II Basis	35	-1.48	2.82	0.00	2.70	-0.94	0.00
Treated	Lateral tooth II Cusp	35	8.46	2.12	0.00	2.12	10.38	0.00
Treated	Lateral tooth II Stylus	35	0.80	-40.02	-14.39	-39.52	2.52	0.00
Treated	Marginal tooth Basis	35	-2.66	0.00	0.00	0.00	5.37	0.00
Treated	Marginal tooth Cusp	35	-5.32	0.00	-20.59	-17.07	-4.86	0.00
Treated	Marginal tooth Stylus	35	1.11	0.00	20.00	20.00	0.83	0.00

Treated	Central tooth Cusp	36	-5.34	0.00	26.00	26.00	5.02	0.00
Treated	Central tooth Stylus	36	6.31	0.00	-49.30	-33.33	10.19	20.00
Treated	Lateral tooth I Cusp	36	0.98	0.00	0.00	0.00	4.74	0.00
Treated	Lateral tooth I Stylus	36	3.32	0.00	0.00	0.00	3.37	0.00
Treated	Lateral tooth II Basis	36	0.00	4.23	0.00	5.41	-0.01	6.25
Treated	Lateral tooth II Cusp	36	-10.89	-2.43	0.00	-2.43	-9.72	0.00
Treated	Lateral tooth II Stylus	36	4.25	13.50	-24.46	9.60	4.29	0.00
Treated	Marginal tooth Basis	36	4.26	0.00	0.00	15.38	-14.92	100.00
Treated	Marginal tooth Cusp	36	1.90	0.00	0.00	0.00	1.75	0.00
Treated	Marginal tooth Stylus	36	1.11	0.00	80.00	80.00	0.84	0.00
Treated	Central tooth Cusp	37	6.95	0.00	-100.00	-100.00	7.02	0.00
Treated	Central tooth Stylus	37	-6.83	100.00	11.27	50.00	-6.12	0.00
Treated	Lateral tooth I Cusp	37	2.64	0.00	0.00	0.00	-1.51	0.00
Treated	Lateral tooth I Stylus	37	1.21	0.00	0.00	0.00	1.20	0.00
Treated	Lateral tooth II Basis	37	8.35	-5.63	33.33	17.57	0.41	-6.25
Treated	Lateral tooth II Cusp	37	-3.83	-3.00	0.00	-2.99	1.86	0.00
Treated	Lateral tooth II Stylus	37	1.76	3.23	-5.04	2.68	1.78	100.00
Treated	Marginal tooth Basis	37	0.00	0.00	0.00	-7.69	9.96	-50.00
Treated	Marginal tooth Cusp	37	3.71	0.00	0.00	0.00	3.35	0.00
Treated	Marginal tooth Stylus	37	1.11	0.00	-100.00	-100.00	0.84	0.00
Treated	Central tooth Cusp	38	-1.61	0.00	0.00	0.00	-1.61	0.00
Treated	Central tooth Stylus	38	1.55	-100.00	-35.21	-78.57	1.06	-20.00
Treated	Lateral tooth I Cusp	38	-3.40	0.00	0.00	0.00	-77.88	0.00
Treated	Lateral tooth I Stylus	38	1.41	0.00	0.00	0.00	1.41	0.00
Treated	Lateral tooth II Basis	38	-9.46	4.23	66.67	52.70	5.98	0.00
Treated	Lateral tooth II Cusp	38	2.25	14.95	5.00	15.06	2.71	50.00
Treated	Lateral tooth II Stylus	38	3.05	-0.78	0.00	-1.02	3.05	-100.00
Treated	Marginal tooth Basis	38	-9.06	0.00	100.00	92.31	10.08	-50.00
Treated	Marginal tooth Cusp	38	-2.66	0.00	0.00	0.00	-3.05	0.00
Treated	Marginal tooth Stylus	38	1.21	53.57	0.00	42.86	0.85	0.00

3. Correlation coefficients:

Supplementary Table 13. For all tooth types together (treated and native), correlations, estimated by row-wise method, between the parameters hardness, Young's modulus, Na, Mg, Si, P, S, Cl, K, Ca, and Fe are shown.

	Hardness, GPa	Young's modulus, GPa	Na, %	Mg, %	Si, %	P, %	S, %	Cl, %	K, %	Ca, %	Fe, %
Hardness, GPa	1.0000	0.9912	0.1242	0.2038	0.0651	0.2476	0.0273	-0.0231	0.0695	0.6841	0.7575
Young's modulus, GPa	0.9912	1.0000	0.1346	0.2100	0.0687	0.2552	0.0369	-0.0206	0.0703	0.6973	0.7013
Na, %	0.1242	0.1346	1.0000	0.2753	0.1849	0.2991	-0.0013	-0.0085	0.1174	0.1381	0.1531
Mg, %	0.2038	0.2100	0.2753	1.0000	0.1053	0.3242	0.0044	-0.0107	0.1086	0.2329	0.1773
Si, %	0.0651	0.0687	0.1849	0.1053	1.0000	0.1437	-0.0044	-0.0057	0.1159	0.0883	0.0727
P, %	0.2476	0.2552	0.2991	0.3242	0.1437	1.0000	0.0592	-0.0108	0.1048	0.3006	0.2543
S, %	0.0273	0.0369	-0.0013	0.0044	-0.0044	0.0592	1.0000	0.0042	-0.0045	0.0541	-0.0092
Cl, %	-0.0231	-0.0206	-0.0085	-0.0107	-0.0057	-0.0108	0.0042	1.0000	-0.0063	0.0453	-0.0268
K, %	0.0695	0.0703	0.1174	0.1086	0.1159	0.1048	-0.0045	-0.0063	1.0000	0.0628	0.0526
Ca, %	0.6841	0.6973	0.1381	0.2329	0.0883	0.3006	0.0541	0.0453	0.0628	1.0000	0.4221
Fe, %	0.7575	0.7013	0.1531	0.1773	0.0727	0.2543	-0.0092	-0.0268	0.0526	0.4221	1.0000

Supplementary Table 14. For all native tooth types together, correlations, estimated by row-wise method, between the parameters hardness, Young's modulus, Na, Mg, Si, P, S, Cl, K, Ca, and Fe are shown.

	Hardness, GPa	Young's modulus, GPa	Na, %	Mg, %	Si, %	P, %	S, %	Cl, %	K, %	Ca, %	Fe, %
Hardness, GPa	1.0000	0.9909	0.1428	0.2517	0.0817	0.3005	-0.0045	-0.0385	0.0806	0.7076	0.6953
Young's modulus, GPa	0.9909	1.0000	0.1509	0.2516	0.0828	0.3021	-0.0015	-0.0385	0.0805	0.6889	0.6862
Na, %	0.1428	0.1509	1.0000	0.2728	0.1910	0.2929	-0.0160	-0.0150	0.1215	0.1139	0.2280
Mg, %	0.2517	0.2516	0.2728	1.0000	0.1072	0.3199	-0.0135	-0.0187	0.1128	0.2127	0.2694
Si, %	0.0817	0.0828	0.1910	0.1072	1.0000	0.1460	-0.0135	-0.0097	0.1269	0.0789	0.1161
P, %	0.3005	0.3021	0.2929	0.3199	0.1460	1.0000	0.0403	-0.0198	0.1069	0.2783	0.3807
S, %	-0.0045	-0.0015	-0.0160	-0.0135	-0.0135	0.0403	1.0000	-0.0062	-0.0115	-0.0137	-0.0068
Cl, %	-0.0385	-0.0385	-0.0150	-0.0187	-0.0097	-0.0198	-0.0062	1.0000	-0.0093	0.0235	-0.0317
K, %	0.0806	0.0805	0.1215	0.1128	0.1269	0.1069	-0.0115	-0.0093	1.0000	0.0555	0.0792
Ca, %	0.7076	0.6889	0.1139	0.2127	0.0789	0.2783	-0.0137	0.0235	0.0555	1.0000	0.6794
Fe, %	0.6953	0.6862	0.2280	0.2694	0.1161	0.3807	-0.0068	-0.0317	0.0792	0.6794	1.0000

Supplementary Table 15. For all treated tooth types together, correlations, estimated by row-wise method, between the parameters hardness, Young's modulus, Na, Mg, Si, P, S, Cl, K, Ca, and Fe are shown.

	Hardness, GPa	Young's modulus, GPa	Na, %	Mg, %	Si, %	P, %	S, %	Cl, %	K, %	Ca, %	Fe, %
Hardness, GPa	1.0000	0.9356	0.0170	-0.0224	-0.0157	0.0110	-0.0202	-0.0361	0.0238	0.0863	0.7718
Young's modulus, GPa	0.9356	1.0000	0.0107	-0.0202	-0.0150	-0.0075	-0.0223	-0.0383	0.0183	0.0955	0.8511
Na, %	0.0170	0.0107	1.0000	-0.0006	-0.0005	-0.0008	-0.0014	-0.0016	-0.0015	-0.0055	0.0075
Mg, %	-0.0224	-0.0202	-0.0006	1.0000	-0.0006	-0.0008	-0.0015	-0.0017	-0.0015	-0.0019	-0.0082
Si, %	-0.0157	-0.0150	-0.0005	-0.0006	1.0000	-0.0007	-0.0013	-0.0014	-0.0013	-0.0050	-0.0076
P, %	0.0110	-0.0075	-0.0008	-0.0008	-0.0007	1.0000	-0.0018	-0.0020	-0.0019	-0.0070	-0.0107
S, %	-0.0202	-0.0223	-0.0014	-0.0015	-0.0013	-0.0018	1.0000	-0.0038	-0.0035	0.0391	-0.0196
Cl, %	-0.0361	-0.0383	-0.0016	-0.0017	-0.0014	-0.0020	-0.0038	1.0000	-0.0039	0.0251	-0.0212
K, %	0.0238	0.0183	-0.0015	-0.0015	-0.0013	-0.0019	-0.0035	-0.0039	1.0000	-0.0111	0.0099
Ca, %	0.0863	0.0955	-0.0055	-0.0019	-0.0050	-0.0070	0.0391	0.0251	-0.0111	1.0000	0.1100
Fe, %	0.7718	0.8511	0.0075	-0.0082	-0.0076	-0.0107	-0.0196	-0.0212	0.0099	0.1100	1.0000

Supplementary Table 16. For all native central teeth, correlations, estimated by row-wise method, between the parameters hardness, Young's modulus, Na, Mg, Si, P, S, Cl, K, Ca, and Fe are shown.

	Hardness, GPa	Young's modulus, GPa	Na, %	Mg, %	Si, %	P, %	S, %	Cl, %	K, %	Ca, %	Fe, %
Hardness, GPa	1.0000	0.9988	0.0000	0.0000	0.0000	0.0000	-0.0781	-0.0789	0.0713	0.3244	-0.0043
Young's modulus, GPa	0.9988	1.0000	0.0000	0.0000	0.0000	0.0000	-0.0789	-0.0787	0.0700	0.3235	-0.0054
Na, %	0.0000	0.0000	1.0000	0.0000	0.0000	0.0000	0.0000	0.0000	0.0000	0.0000	0.0000
Mg, %	0.0000	0.0000	0.0000	1.0000	0.0000	0.0000	0.0000	0.0000	0.0000	0.0000	0.0000
Si, %	0.0000	0.0000	0.0000	0.0000	1.0000	0.0000	0.0000	0.0000	0.0000	0.0000	0.0000
P, %	0.0000	0.0000	0.0000	0.0000	0.0000	1.0000	0.0000	0.0000	0.0000	0.0000	0.0000
S, %	-0.0781	-0.0789	0.0000	0.0000	0.0000	0.0000	1.0000	-0.0262	-0.0218	-0.0928	0.0204
Cl, %	-0.0789	-0.0787	0.0000	0.0000	0.0000	0.0000	-0.0262	1.0000	-0.0088	-0.0324	0.0125
K, %	0.0713	0.0700	0.0000	0.0000	0.0000	0.0000	-0.0218	-0.0088	1.0000	-0.0422	-0.0151
Ca, %	0.3244	0.3235	0.0000	0.0000	0.0000	0.0000	-0.0928	-0.0324	-0.0422	1.0000	0.0376
Fe, %	-0.0043	-0.0054	0.0000	0.0000	0.0000	0.0000	0.0204	0.0125	-0.0151	0.0376	1.0000

Supplementary Table 17. For all native lateral teeth I, correlations, estimated by row-wise method, between the parameters hardness, Young's modulus, Na, Mg, Si, P, S, Cl, K, Ca, and Fe are shown.

	Hardness, GPa	Young's modulus, GPa	Na, %	Mg, %	Si, %	P, %	S, %	Cl, %	K, %	Ca, %	Fe, %
Hardness, GPa	1.0000	0.9988	0.0000	0.0000	0.0892	0.0000	-0.1028	-0.1500	0.0000	0.2784	-0.1056
Young's modulus, GPa	0.9988	1.0000	0.0000	0.0000	0.0988	0.0000	-0.0989	-0.1491	0.0000	0.2716	-0.1049
Na, %	0.0000	0.0000	1.0000	0.0000	0.0000	0.0000	0.0000	0.0000	0.0000	0.0000	0.0000
Mg, %	0.0000	0.0000	0.0000	1.0000	0.0000	0.0000	0.0000	0.0000	0.0000	0.0000	0.0000
Si, %	0.0892	0.0988	0.0000	0.0000	1.0000	0.0000	-0.0156	-0.0099	0.0000	0.1612	-0.0074
P, %	0.0000	0.0000	0.0000	0.0000	0.0000	1.0000	0.0000	0.0000	0.0000	0.0000	0.0000
S, %	-0.1028	-0.0989	0.0000	0.0000	-0.0156	0.0000	1.0000	0.0298	0.0000	-0.0729	0.0887
Cl, %	-0.1500	-0.1491	0.0000	0.0000	-0.0099	0.0000	0.0298	1.0000	0.0000	-0.0643	-0.0174
K, %	0.0000	0.0000	0.0000	0.0000	0.0000	0.0000	0.0000	0.0000	1.0000	0.0000	0.0000
Ca, %	0.2784	0.2716	0.0000	0.0000	0.1612	0.0000	-0.0729	-0.0643	0.0000	1.0000	-0.0481
Fe, %	-0.1056	-0.1049	0.0000	0.0000	-0.0074	0.0000	0.0887	-0.0174	0.0000	-0.0481	1.0000

Supplementary Table 18. For all native lateral teeth II, correlations, estimated by row-wise method, between the parameters hardness, Young's modulus, Na, Mg, Si, P, S, Cl, K, Ca, and Fe are shown.

	Hardness, GPa	Young's modulus, GPa	Na, %	Mg, %	Si, %	P, %	S, %	Cl, %	K, %	Ca, %	Fe, %
Hardness, GPa	1.0000	0.9853	0.0982	0.2167	0.1402	0.2936	0.0558	-0.0528	0.0989	0.7312	0.7867
Young's modulus, GPa	0.9853	1.0000	0.1191	0.2274	0.1433	0.3091	0.0693	-0.0481	0.0997	0.7261	0.7988
Na, %	0.0982	0.1191	1.0000	0.2475	0.2741	0.2758	-0.0251	-0.0265	0.1622	0.0064	0.1739
Mg, %	0.2167	0.2274	0.2475	1.0000	0.1492	0.2914	-0.0209	-0.0349	0.1454	0.0967	0.1912
Si, %	0.1402	0.1433	0.2741	0.1492	1.0000	0.2136	-0.0065	-0.0121	0.1221	0.1029	0.1697
P, %	0.2936	0.3091	0.2758	0.2914	0.2136	1.0000	0.1050	-0.0373	0.1412	0.1916	0.3248
S, %	0.0558	0.0693	-0.0251	-0.0209	-0.0065	0.1050	1.0000	-0.0045	-0.0112	0.0396	0.0037
Cl, %	-0.0528	-0.0481	-0.0265	-0.0349	-0.0121	-0.0373	-0.0045	1.0000	-0.0157	0.0105	-0.0708
K, %	0.0989	0.0997	0.1622	0.1454	0.1221	0.1412	-0.0112	-0.0157	1.0000	0.0430	0.0857
Ca, %	0.7312	0.7261	0.0064	0.0967	0.1029	0.1916	0.0396	0.0105	0.0430	1.0000	0.6077
Fe, %	0.7867	0.7988	0.1739	0.1912	0.1697	0.3248	0.0037	-0.0708	0.0857	0.6077	1.0000

Supplementary Table 19. For all native marginal teeth, correlations, estimated by row-wise method, between the parameters hardness, Young's modulus, Na, Mg, Si, P, S, Cl, K, Ca, and Fe are shown.

	Hardness, GPa	Young's modulus, GPa	Na, %	Mg, %	Si, %	P, %	S, %	Cl, %	K, %	Ca, %	Fe, %
Hardness, GPa	1.0000	0.9897	-0.1001	0.0000	-0.0371	-0.0161	0.0203	-0.0296	-0.0162	0.2251	-0.0968
Young's modulus, GPa	0.9897	1.0000	-0.1046	0.0000	-0.0386	-0.0171	0.0182	-0.0320	-0.0127	0.2370	-0.0928
Na, %	-0.1001	-0.1046	1.0000	0.0000	-0.0073	-0.0044	-0.0198	-0.0107	-0.0045	-0.0401	-0.0091
Mg, %	0.0000	0.0000	0.0000	1.0000	0.0000	0.0000	0.0000	0.0000	0.0000	0.0000	0.0000
Si, %	-0.0371	-0.0386	-0.0073	0.0000	1.0000	-0.0047	-0.0210	-0.0114	0.4424	0.0159	-0.0096
P, %	-0.0161	-0.0171	-0.0044	0.0000	-0.0047	1.0000	-0.0126	-0.0068	-0.0028	-0.0255	-0.0058
S, %	0.0203	0.0182	-0.0198	0.0000	-0.0210	-0.0126	1.0000	-0.0308	-0.0128	-0.0281	-0.0208
Cl, %	-0.0296	-0.0320	-0.0107	0.0000	-0.0114	-0.0068	-0.0308	1.0000	-0.0070	-0.0622	-0.0141
K, %	-0.0162	-0.0127	-0.0045	0.0000	0.4424	-0.0028	-0.0128	-0.0070	1.0000	0.0383	-0.0059
Ca, %	0.2251	0.2370	-0.0401	0.0000	0.0159	-0.0255	-0.0281	-0.0622	0.0383	1.0000	-0.0524
Fe, %	-0.0968	-0.0928	-0.0091	0.0000	-0.0096	-0.0058	-0.0208	-0.0141	-0.0059	-0.0524	1.0000

Supplementary Table 20. For all treated central teeth, correlations, estimated by row-wise method, between the parameters hardness, Young's modulus, Na, Mg, Si, P, S, Cl, K, Ca, and Fe are shown.

	Hardness, GPa	Young's modulus, GPa	Na, %	Mg, %	Si, %	P, %	S, %	Cl, %	K, %	Ca, %	Fe, %
Hardness, GPa	1.0000	0.9974	0.0000	-0.0549	0.0000	0.0000	-0.0374	0.0438	0.0284	0.1179	0.0256
Young's modulus, GPa	0.9974	1.0000	0.0000	-0.0536	0.0000	0.0000	-0.0380	0.0530	0.0278	0.1357	0.0309
Na, %	0.0000	0.0000	1.0000	0.0000	0.0000	0.0000	0.0000	0.0000	0.0000	0.0000	0.0000
Mg, %	-0.0549	-0.0536	0.0000	1.0000	0.0000	0.0000	-0.0051	-0.0051	-0.0069	-0.0007	0.1015
Si, %	0.0000	0.0000	0.0000	0.0000	1.0000	0.0000	0.0000	0.0000	0.0000	0.0000	0.0000
P, %	0.0000	0.0000	0.0000	0.0000	0.0000	1.0000	0.0000	0.0000	0.0000	0.0000	0.0000
S, %	-0.0374	-0.0380	0.0000	-0.0051	0.0000	0.0000	1.0000	-0.0044	-0.0059	-0.0152	-0.0104
Cl, %	0.0438	0.0530	0.0000	-0.0051	0.0000	0.0000	-0.0044	1.0000	-0.0059	-0.0152	0.2499
K, %	0.0284	0.0278	0.0000	-0.0069	0.0000	0.0000	-0.0059	-0.0059	1.0000	-0.0205	-0.0140
Ca, %	0.1179	0.1357	0.0000	-0.0007	0.0000	0.0000	-0.0152	-0.0152	-0.0205	1.0000	0.0179
Fe, %	0.0256	0.0309	0.0000	0.1015	0.0000	0.0000	-0.0104	0.2499	-0.0140	0.0179	1.0000

Supplementary Table 21. For all treated lateral teeth I, correlations, estimated by row-wise method, between the parameters hardness, Young's modulus, Na, Mg, Si, P, S, Cl, K, Ca, and Fe are shown.

	Hardness, GPa	Young's modulus, GPa	Na, %	Mg, %	Si, %	P, %	S, %	Cl, %	K, %	Ca, %	Fe, %
Hardness, GPa	1.0000	0.9932	0.0000	0.0000	0.0000	-0.0174	0.0055	-0.0923	0.0000	-0.0063	-0.0986
Young's modulus, GPa	0.9932	1.0000	0.0000	0.0000	0.0000	-0.0132	0.0024	-0.0918	0.0000	-0.0143	-0.1015
Na, %	0.0000	0.0000	1.0000	0.0000	0.0000	0.0000	0.0000	0.0000	0.0000	0.0000	0.0000
Mg, %	0.0000	0.0000	0.0000	1.0000	0.0000	0.0000	0.0000	0.0000	0.0000	0.0000	0.0000
Si, %	0.0000	0.0000	0.0000	0.0000	1.0000	0.0000	0.0000	0.0000	0.0000	0.0000	0.0000
P, %	-0.0174	-0.0132	0.0000	0.0000	0.0000	1.0000	-0.0037	-0.0044	0.0000	-0.0060	-0.0053
S, %	0.0055	0.0024	0.0000	0.0000	0.0000	-0.0037	1.0000	-0.0074	0.0000	0.3068	-0.0089
Cl, %	-0.0923	-0.0918	0.0000	0.0000	0.0000	-0.0044	-0.0074	1.0000	0.0000	-0.0121	-0.0107
K, %	0.0000	0.0000	0.0000	0.0000	0.0000	0.0000	0.0000	0.0000	1.0000	0.0000	0.0000
Ca, %	-0.0063	-0.0143	0.0000	0.0000	0.0000	-0.0060	0.3068	-0.0121	0.0000	1.0000	-0.0146
Fe, %	-0.0986	-0.1015	0.0000	0.0000	0.0000	-0.0053	-0.0089	-0.0107	0.0000	-0.0146	1.0000

Supplementary Table 22. For all treated lateral teeth II, correlations, estimated by row-wise method, between the parameters hardness, Young's modulus, Na, Mg, Si, P, S, Cl, K, Ca, and Fe are given.

	Hardness, GPa	Young's modulus, GPa	Na, %	Mg, %	Si, %	P, %	S, %	Cl, %	K, %	Ca, %	Fe, %
Hardness, GPa	1.0000	0.9171	-0.0004	0.0000	0.0000	0.0213	-0.0475	-0.0319	0.0301	-0.0305	0.8184
Young's modulus, GPa	0.9171	1.0000	-0.0047	0.0000	0.0000	-0.0160	-0.0543	-0.0415	0.0140	0.0038	0.9436
Na, %	-0.0004	-0.0047	1.0000	0.0000	0.0000	-0.0016	-0.0031	-0.0032	-0.0032	-0.0129	-0.0107
Mg, %	0.0000	0.0000	0.0000	1.0000	0.0000	0.0000	0.0000	0.0000	0.0000	0.0000	0.0000
Si, %	0.0000	0.0000	0.0000	0.0000	1.0000	0.0000	0.0000	0.0000	0.0000	0.0000	0.0000
P, %	0.0213	-0.0160	-0.0016	0.0000	0.0000	1.0000	-0.0028	-0.0029	-0.0029	-0.0116	-0.0260
S, %	-0.0475	-0.0543	-0.0031	0.0000	0.0000	-0.0028	1.0000	-0.0055	-0.0055	0.0337	-0.0488
Cl, %	-0.0319	-0.0415	-0.0032	0.0000	0.0000	-0.0029	-0.0055	1.0000	-0.0057	0.0853	-0.0479
K, %	0.0301	0.0140	-0.0032	0.0000	0.0000	-0.0029	-0.0055	-0.0057	1.0000	-0.0190	-0.0006
Ca, %	-0.0305	0.0038	-0.0129	0.0000	0.0000	-0.0116	0.0337	0.0853	-0.0190	1.0000	0.0293
Fe, %	0.8184	0.9436	-0.0107	0.0000	0.0000	-0.0260	-0.0488	-0.0479	-0.0006	0.0293	1.0000

Supplementary Table 23. For all treated marginal teeth, correlations, estimated by row-wise method, between the parameters hardness, Young's modulus, Na, Mg, Si, P, S, Cl, K, Ca, and Fe are given.

	Hardness, GPa	Young's modulus, GPa	Na, %	Mg, %	Si, %	P, %	S, %	Cl, %	K, %	Ca, %	Fe, %
Hardness, GPa	1.0000	0.9152	0.0000	0.0000	-0.0327	0.0000	0.0000	0.0179	-0.0264	0.1325	-0.0928
Young's modulus, GPa	0.9152	1.0000	0.0000	0.0000	-0.0337	0.0000	0.0000	0.0300	-0.0057	0.1671	-0.0886
Na, %	0.0000	0.0000	1.0000	0.0000	0.0000	0.0000	0.0000	0.0000	0.0000	0.0000	0.0000
Mg, %	0.0000	0.0000	0.0000	1.0000	0.0000	0.0000	0.0000	0.0000	0.0000	0.0000	0.0000
Si, %	-0.0327	-0.0337	0.0000	0.0000	1.0000	0.0000	0.0000	-0.0020	-0.0019	-0.0102	-0.0041
P, %	0.0000	0.0000	0.0000	0.0000	0.0000	1.0000	0.0000	0.0000	0.0000	0.0000	0.0000
S, %	0.0000	0.0000	0.0000	0.0000	0.0000	0.0000	1.0000	0.0000	0.0000	0.0000	0.0000
Cl, %	0.0179	0.0300	0.0000	0.0000	-0.0020	0.0000	0.0000	1.0000	-0.0027	-0.0143	-0.0058
K, %	-0.0264	-0.0057	0.0000	0.0000	-0.0019	0.0000	0.0000	-0.0027	1.0000	-0.0136	-0.0055
Ca, %	0.1325	0.1671	0.0000	0.0000	-0.0102	0.0000	0.0000	-0.0143	-0.0136	1.0000	-0.0289
Fe, %	-0.0928	-0.0886	0.0000	0.0000	-0.0041	0.0000	0.0000	-0.0058	-0.0055	-0.0289	1.0000

Supplementary Table 24. For all tooth types together (treated and native), correlations, estimated by row-wise method, between the parameters hardness, Young's modulus, Fe, Ca, dry breaking force (Bf), wet bf, dry breaking tress (Bs), and wet Bs are given.

	Hardness, GPa	Young's modulus, GPa	Fe, %	Ca, %	Dry Bf, mN	Wet Bf, mN	Dry Bs, MPa	Wet Bs, MPa
Hardness, GPa	1.0000	0.9912	0.7575	0.6841	0.9022	0.7934	0.1620	0.5206
Young's modulus, GPa	0.9912	1.0000	0.7013	0.6973	0.9001	0.7600	0.0741	0.4301
Fe, %	0.7575	0.7013	1.0000	0.5465	0.8039	0.9372	0.3448	0.8254
Ca, %	0.6841	0.6973	0.5465	1.0000	0.7866	0.4695	0.0512	0.2845
Dry Bf, mN	0.9022	0.9001	0.8039	0.7866	1.0000	0.7803	0.1361	0.5038
Wet Bf, mN	0.7934	0.7600	0.9372	0.4695	0.7803	1.0000	0.0934	0.6900
Dry Bs, MPa	0.1620	0.0741	0.3448	0.0512	0.1361	0.0934	1.0000	0.7419
Wet Bs, MPa	0.5206	0.4301	0.8254	0.2845	0.5038	0.6900	0.7419	1.0000

Supplementary Table 25. For central teeth (native condition), correlations, estimated by row-wise method, between the parameters hardness, Young's modulus, Fe, Ca, dry and wet breaking force (Bf) are given.

	Hardness, GPa	Young's modulus, GPa	Fe, %	Ca, %	Dry Bf, mN	Wet Bf, mN
Hardness, GPa	1.0000	1.0000	0.9853	0.9750	0.9991	0.9946
Young's modulus, GPa	1.0000	1.0000	0.9838	0.9730	0.9995	0.9936
Fe, %	0.9853	0.9838	1.0000	0.9986	0.9773	0.9977
Ca, %	0.9750	0.9730	0.9986	1.0000	0.9649	0.9928
Dry Bf, mN	0.9991	0.9995	0.9773	0.9649	1.0000	0.9894
Wet Bf, mN	0.9946	0.9936	0.9977	0.9928	0.9894	1.0000

Supplementary Table 26. For lateral teeth I (native condition), correlations, estimated by row-wise method, between the parameters hardness, Young's modulus, Fe, Ca, dry and wet breaking force (Bf) are given.

	Hardness, GPa	Young's modulus, GPa	Fe, %	Ca, %	Dry Bf, mN	Wet Bf, mN
Hardness, GPa	1.0000	1.0000	0.4931	0.9914	0.9994	0.9999
Young's modulus, GPa	1.0000	1.0000	0.4911	0.9917	0.9995	0.9999
Fe, %	0.4931	0.4911	1.0000	0.3752	0.4639	0.4783
Ca, %	0.9914	0.9917	0.3752	1.0000	0.9952	0.9935
Dry Bf, mN	0.9994	0.9995	0.4639	0.9952	1.0000	0.9999
Wet Bf, mN	0.9999	0.9999	0.4783	0.9935	0.9999	1.0000

Supplementary Table 27. For lateral teeth II (native condition), correlations, estimated by row-wise method, between the parameters hardness, Young's modulus, Fe, Ca, dry breaking force (Bf), wet bf, dry breaking tress (Bs), and wet Bs are given.

	Hardness, GPa	Young's modulus, GPa	Fe, %	Ca, %	Dry Bf, mN	Wet Bf, mN	Dry Bs, MPa	Wet Bs, MPa
Hardness, GPa	1.0000	1.0000	0.9496	0.9270	0.9683	0.9482	0.9866	0.9726
Young's modulus, GPa	1.0000	1.0000	0.9509	0.9255	0.9693	0.9495	0.9872	0.9735
Fe, %	0.9496	0.9509	1.0000	0.7628	0.9978	1.0000	0.9880	0.9965
Ca, %	0.9270	0.9255	0.7628	1.0000	0.8039	0.7599	0.8533	0.8144
Dry Bf, mN	0.9683	0.9693	0.9978	0.8039	1.0000	0.9975	0.9961	0.9998
Wet Bf, mN	0.9482	0.9495	1.0000	0.7599	0.9975	1.0000	0.9874	0.9961
Dry Bs, MPa	0.9866	0.9872	0.9880	0.8533	0.9961	0.9874	1.0000	0.9975
Wet Bs, MPa	0.9726	0.9735	0.9965	0.8144	0.9998	0.9961	0.9975	1.0000

Supplementary Table 28. For marginal teeth (native condition), correlations, estimated by row-wise method, between the parameters hardness, Young's modulus, Fe, Ca, dry breaking force (Bf), wet bf, dry breaking tress (Bs), and wet Bs are given.

	Hardness, GPa	Young's modulus, GPa	Fe, %	Ca, %	Dry Bf, mN	Wet Bf, mN	Dry Bs, MPa	Wet Bs, MPa
Hardness, GPa	1.0000	0.9998	-0.9745	0.8405	0.7391	0.9770	0.9979	0.7160
Young's modulus, GPa	0.9998	1.0000	-0.9700	0.8508	0.7520	0.9809	0.9990	0.7293
Fe, %	-0.9745	-0.9700	1.0000	-0.6975	-0.5691	-0.9042	-0.9579	-0.5410
Ca, %	0.8405	0.8508	-0.6975	1.0000	0.9862	0.9367	0.8738	0.9800
Dry Bf, mN	0.7391	0.7520	-0.5691	0.9862	1.0000	0.8658	0.7812	0.9994
Wet Bf, mN	0.9770	0.9809	-0.9042	0.9367	0.8658	1.0000	0.9887	0.8484
Dry Bs, MPa	0.9979	0.9990	-0.9579	0.8738	0.7812	0.9887	1.0000	0.7597
Wet Bs, MPa	0.7160	0.7293	-0.5410	0.9800	0.9994	0.8484	0.7597	1.0000

Supplementary Table 29. For central teeth (treated condition), correlations, estimated by row-wise method, between the parameters hardness, Young's modulus, Fe, Ca, dry and wet breaking force (Bf) are given.

	Hardness, GPa	Young's modulus, GPa	Fe, %	Ca, %	Dry Bf, mN	Wet Bf, mN
Hardness, GPa	1.0000	1.0000	-0.9281	0.9817	0.9983	0.9549
Young's modulus, GPa	1.0000	1.0000	-0.9263	0.9808	0.9980	0.9563
Fe, %	-0.9281	-0.9263	1.0000	-0.9820	-0.9481	-0.7756
Ca, %	0.9817	0.9808	-0.9820	1.0000	0.9911	0.8809
Dry Bf, mN	0.9983	0.9980	-0.9481	0.9911	1.0000	0.9360
Wet Bf, mN	0.9549	0.9563	-0.7756	0.8809	0.9360	1.0000

Supplementary Table 30. For lateral teeth I (treated condition), correlations, estimated by row-wise method, between the parameters hardness, Young's modulus, Fe, Ca, dry and wet breaking force (Bf) are given.

	Hardness, GPa	Young's modulus, GPa	Fe, %	Ca, %	Dry Bf, mN	Wet Bf, mN
Hardness, GPa	1.0000	0.9999	0.4790	-0.1653	0.9992	0.9988
Young's modulus, GPa	0.9999	1.0000	0.4890	-0.1765	0.9996	0.9993
Fe, %	0.4790	0.4890	1.0000	-0.9449	0.5128	0.5207
Ca, %	-0.1653	-0.1765	-0.9449	1.0000	-0.2035	-0.2125
Dry Bf, mN	0.9992	0.9996	0.5128	-0.2035	1.0000	1.0000
Wet Bf, mN	0.9988	0.9993	0.5207	-0.2125	1.0000	1.0000

Supplementary Table 31. For lateral teeth II (treated condition), correlations, estimated by row-wise method, between the parameters hardness, Young's modulus, Fe, Ca, dry breaking force (Bf), wet bf, dry breaking tress (Bs), and wet Bs are given.

	Hardness, GPa	Young's modulus, GPa	Fe, %	Ca, %	Dry Bf, mN	Wet Bf, mN	Dry Bs, MPa	Wet Bs, MPa
Hardness, GPa	1.0000	0.9874	0.9516	0.8612	0.9816	0.9486	0.9995	0.9524
Young's modulus, GPa	0.9874	1.0000	0.9883	0.9308	0.9995	0.9867	0.9918	0.9886
Fe, %	0.9516	0.9883	1.0000	0.9757	0.9928	1.0000	0.9606	1.0000
Ca, %	0.8612	0.9308	0.9757	1.0000	0.9424	0.9778	0.8765	0.9752
Dry Bf, mN	0.9816	0.9995	0.9928	0.9424	1.0000	0.9916	0.9870	0.9931
Wet Bf, mN	0.9486	0.9867	1.0000	0.9778	0.9916	1.0000	0.9579	0.9999
Dry Bs, MPa	0.9995	0.9918	0.9606	0.8765	0.9870	0.9579	1.0000	0.9613
Wet Bs, MPa	0.9524	0.9886	1.0000	0.9752	0.9931	0.9999	0.9613	1.0000

Supplementary Table 32. For marginal teeth (treated condition), correlations, estimated by row-wise method, between the parameters hardness, Young's modulus, Fe, Ca, dry breaking force (Bf), wet bf, dry breaking tress (Bs), and wet Bs are given.

	Hardness, GPa	Young's modulus, GPa	Fe, %	Ca, %	Dry Bf, mN	Wet Bf, mN	Dry Bs, MPa	Wet Bs, MPa
Hardness, GPa	1.0000	0.9911	-0.9984	0.9911	0.9096	0.9911	0.9156	0.9788
Young's modulus, GPa	0.9911	1.0000	-0.9819	1.0000	0.9569	1.0000	0.9611	0.9426
Fe, %	-0.9984	-0.9819	1.0000	-0.9820	-0.8846	-0.9820	-0.8913	-0.9888
Ca, %	0.9911	1.0000	-0.9820	1.0000	0.9568	1.0000	0.9610	0.9428
Dry Bf, mN	0.9096	0.9569	-0.8846	0.9568	1.0000	0.9568	0.9999	0.8052
Wet Bf, mN	0.9911	1.0000	-0.9820	1.0000	0.9568	1.0000	0.9610	0.9428
Dry Bs, MPa	0.9156	0.9611	-0.8913	0.9610	0.9999	0.9610	1.0000	0.8137
Wet Bs, MPa	0.9788	0.9426	-0.9888	0.9428	0.8052	0.9428	0.8137	1.0000

4. Relationships between parameters including results from ANOVA and PCA:

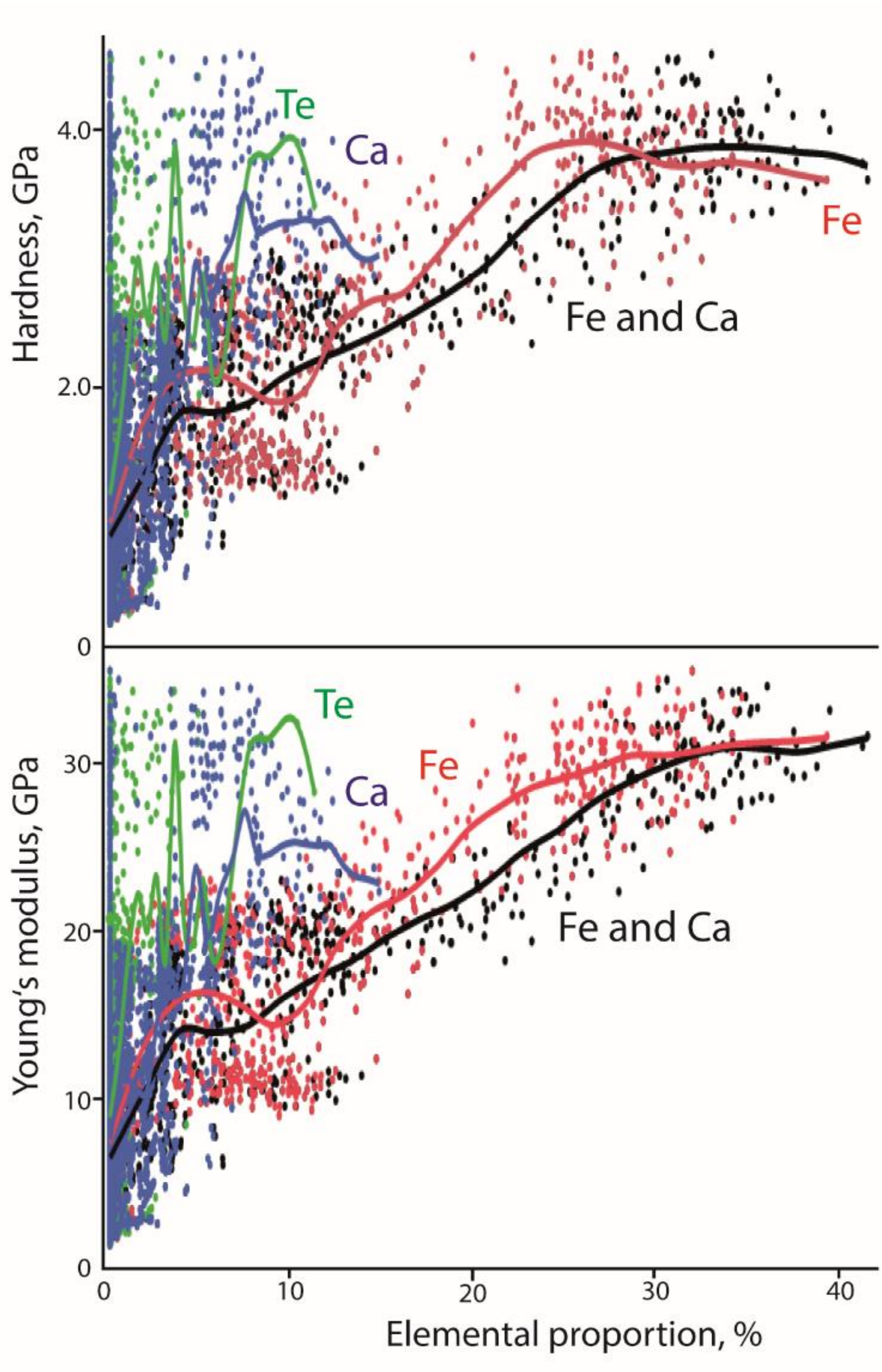

Supplementary Figure 8. Relationship between E, H, and the measured elemental proportions for all tooth types (treated and native).

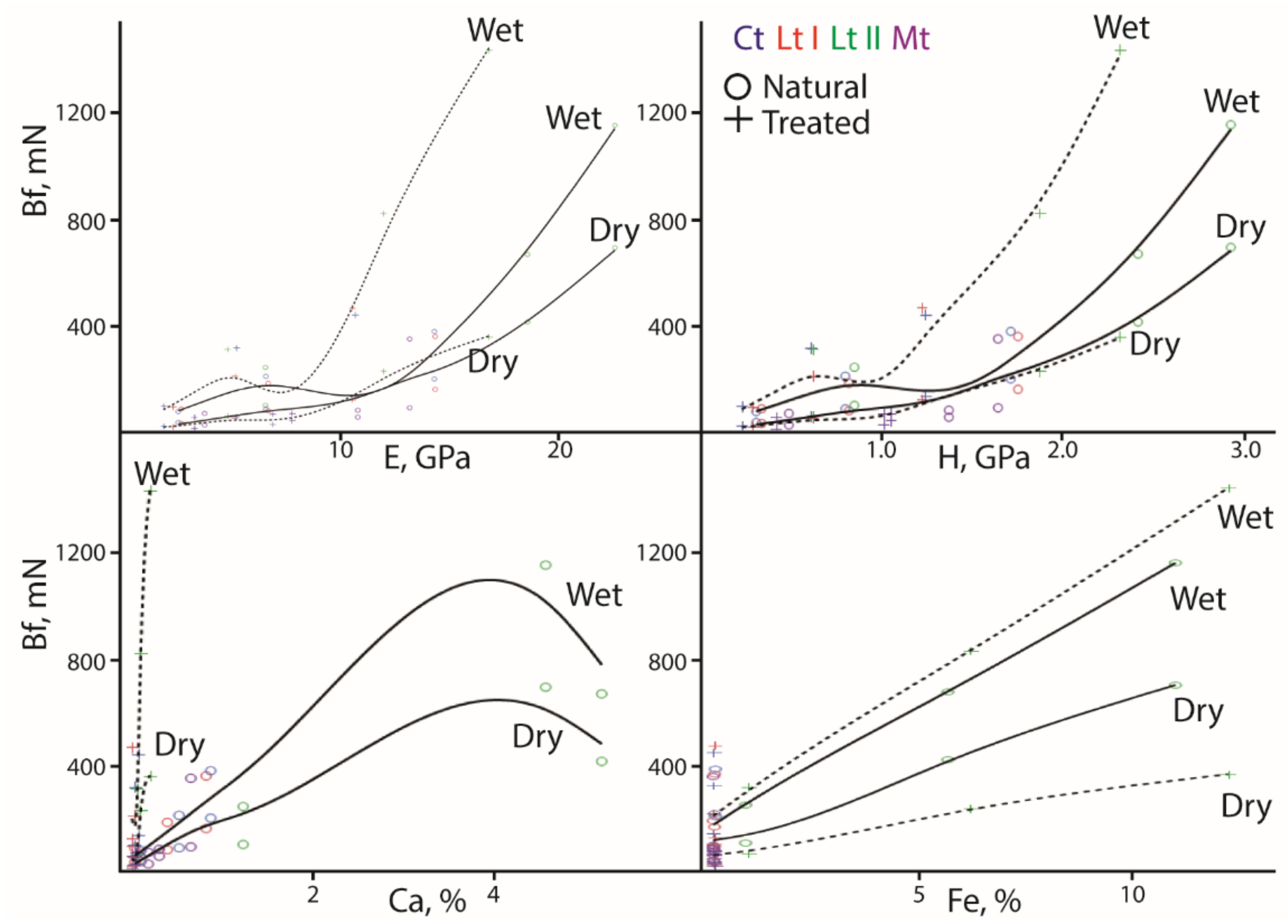

Supplementary Figure 9. Relationship between breaking force (tested under wet and dry condition) and Young's modulus (E), hardness (H), Ca, and Fe for treated and native teeth.

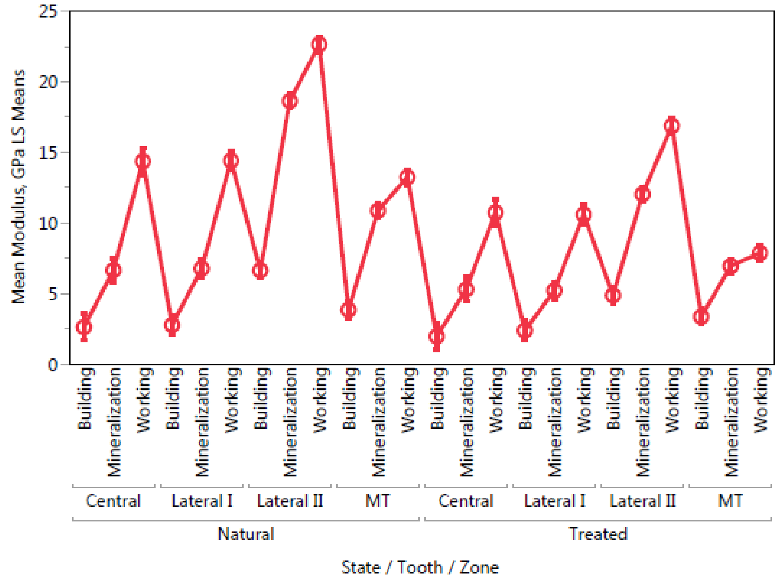

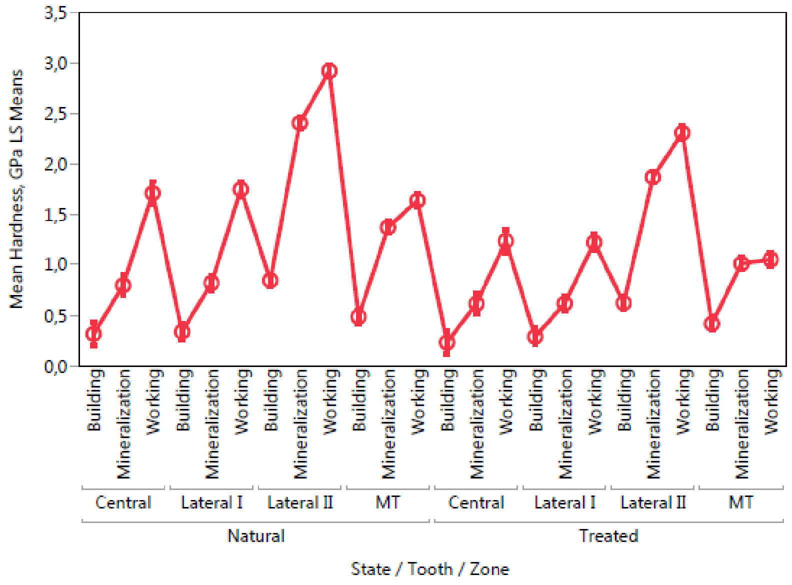

Supplementary Figure 10. Least square mean plots of 3-way ANOVA for E (*above*) and H (*below*).

Supplementary Table 33. 3-way ANOVA table and the effect tests with the interaction terms for Young's modulus.

Analysis of Variance for Modulus					
**Source**	**DF**	**Sum of Squares**	**Mean Square**	**F Ratio**	**Prob > F**
Model	23	132445.77	5758.51	363.9159	<.0001*
Error	4120	65193.82	15.82		
C. Total	4143	197639.59			
**Effect Tests for Modulus**					
**Source**	**Nparm**	**DF**	**Sum of Squares**	**F Ratio**	**Prob > F**
State	1	1	7262.744	458.9776	<.0001*
Tooth	3	3	36235.024	763.3049	<.0001*
Zone	2	2	55229.683	1745.152	<.0001*
State*Tooth	3	3	1348.904	28.4152	<.0001*
State*Zone	2	2	1980.911	62.5930	<.0001*
Tooth*Zone	6	6	8447.405	88.9739	<.0001*
State*Tooth*Zone	6	6	568.054	5.9831.6	<.0001*

Supplementary Table 34. 3-way ANOVA table and the effect tests with the interaction terms for hardness.

Analysis of Variance for Hardness					
Source	DF	Sum of Squares	Mean Square	F Ratio	Prob > F
Model	23	2331.0694	101.351	424.0671	<.0001*
Error	4120	984.6685	0.239		
C. Total	4143	3315.7380			

Effect Tests for Hardness					
Source	Nparm	DF	Sum of Squares	F Ratio	Prob > F
State	1	1	88.80607	371.5778	<.0001*
Tooth	3	3	781.86988	1090.487	<.0001*
Zone	2	2	871.22416	1822.666	<.0001*
State*Tooth	3	3	7.11882	9.9287	<.0001*
State*Zone	2	2	25.55181	53.4563	<.0001*
Tooth*Zone	6	6	181.48954	126.5632	<.0001*
State*Tooth*Zone	6	6	2.28510	1.5935	<.0001*

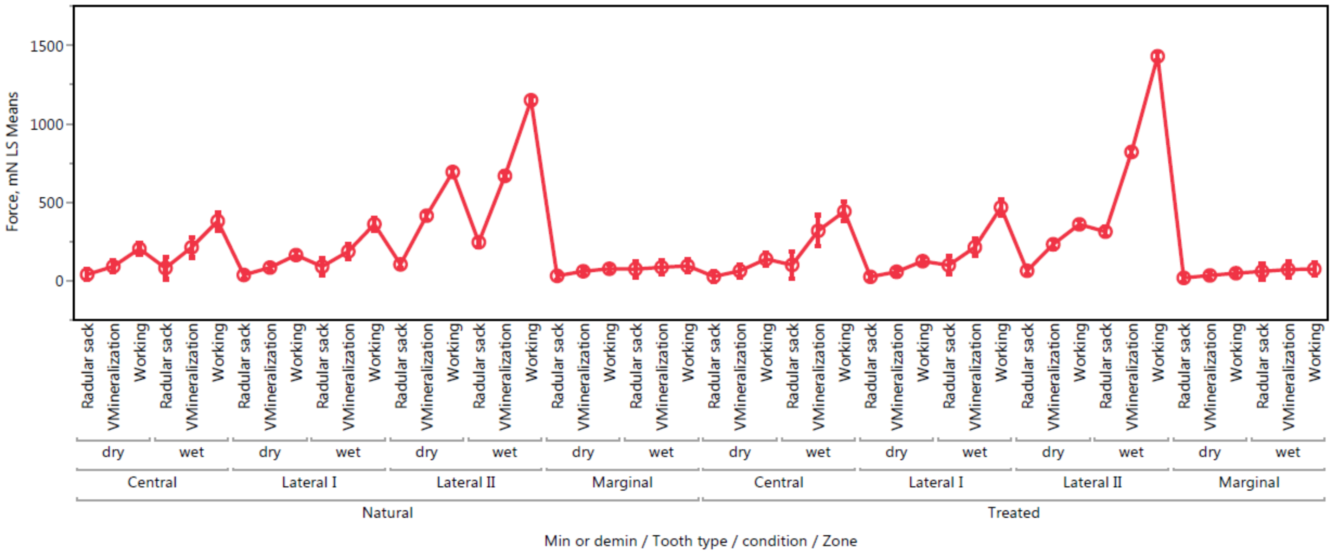

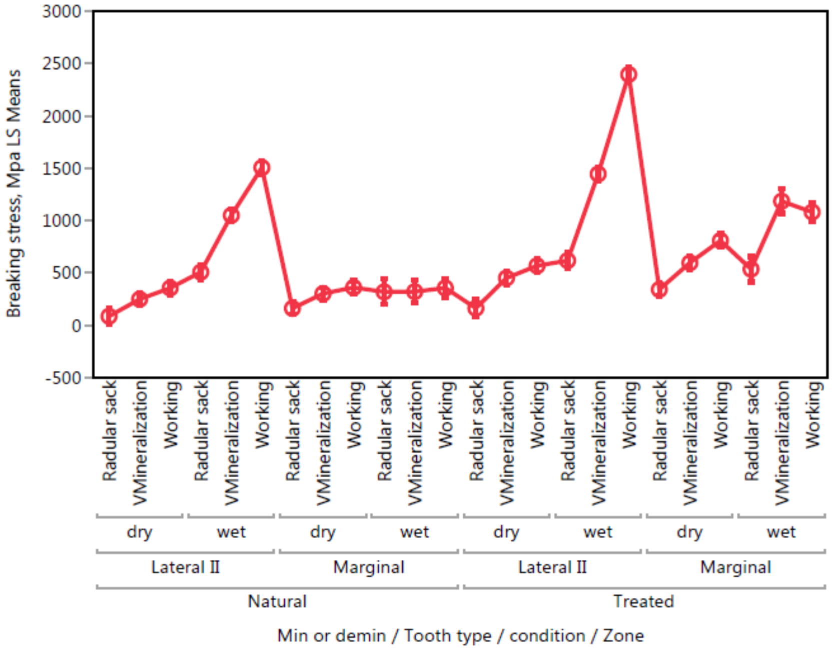

Supplementary Figure 11. Least square mean plots of 4-way ANOVA for breaking force (*above*) and stress (*below*).

Supplementary Table 35. 4-way ANOVA table and the effect tests with the interaction terms for breaking force.

Analysis of Variance for breaking force					
**Source**	**DF**	**Sum of Squares**	**Mean Square**	**F Ratio**	**Prob > F**
Model	47	145932684	3104951	396.8448	<.0001*
Error	1253	9803588	7824		
C. Total	1300	155736273			
**Effect Tests for breaking force**					
**Source**	**Nparm**	**DF**	**Sum of Squares**	**F Ratio**	**Prob > F**
State (native or treated)	1	1	265	0.0339	<.0001*
Tooth type	3	3	47066355	2005.189	<.0001*
Condition (wet or dry)	1	1	7955540	1016.800	<.0001*
Zone	2	2	12487644	798.0250	<.0001*
State*Tooth type	3	3	37628	1.6031	<.0001*
State*Condition	1	1	854514	109.2157	<.0001*
State*Zone	2	2	1364	0.0872	<.0001*
Tooth type*Condition	3	3	8706140	370.9116	<.0001*
Tooth type*Zone	6	6	15759889	335.7128	<.0001*
Condition*Zone	2	2	1920481	122.7287	<.0001*
State*Tooth type*Condition	3	3	1522538	64.8653	<.0001*
State*Tooth type*Zone	6	6	48186	1.0265	<.0001*
State*Condition*Zone	2	2	290605	18.5712	<.0001*
Tooth type*Condition*Zone	6	6	2680047	57.0896	<.0001*
State*Tooth type*Condition*Zone	6	6	537724	11.4544	<.0001*

Supplementary Table 36. 4-way ANOVA table and the effect tests with the interaction terms for breaking stress.

Analysis of Variance for breaking stress					
**Source**	**DF**	**Sum of Squares**	**Mean Square**	**F Ratio**	**Prob > F**
Model	23	241531786	10501382	248.8659	<.0001*
Error	740	31225748	42196.957		
C. Total	763	272757535			
**Effect Tests for breaking stress**					
**Source**	**Nparm**	**DF**	**Sum of Squares**	**F Ratio**	**Prob > F**
State (native or treated)	1	1	22739261	**538.8839**	<.0001*
Tooth type	1	1	9769365	**231.5182**	<.0001*
Condition (wet or dry)	1	1	50475041	**1196.177**	<.0001*
Zone	2	2	34700390	**411.1717**	<.0001*
State*Tooth type	1	1	755927	**17.9143**	<.0001*
State*Condition	1	1	3407958	**80.7631**	<.0001*
State*Zone	2	2	4544316	**53.8465**	<.0001*
Tooth type*Condition	1	1	20894659	**495.1698**	<.0001*
Tooth type*Zone	2	2	8250334	**97.7598**	<.0001*
Condition*Zone	2	2	6393987	**75.7636**	<.0001*
State*Tooth type*Condition	1	1	425	**0.0101**	<.0001*
State*Tooth type*Zone	2	2	447224	**5.2992**	0.0052*
State*Condition*Zone	2	2	1288471	**15.2673**	<.0001*
Tooth type*Condition*Zone	2	2	8384834	**99.3535**	<.0001*
State*Tooth type*Condition*Zone	2	2	1087002	**12.8801**	<.0001*

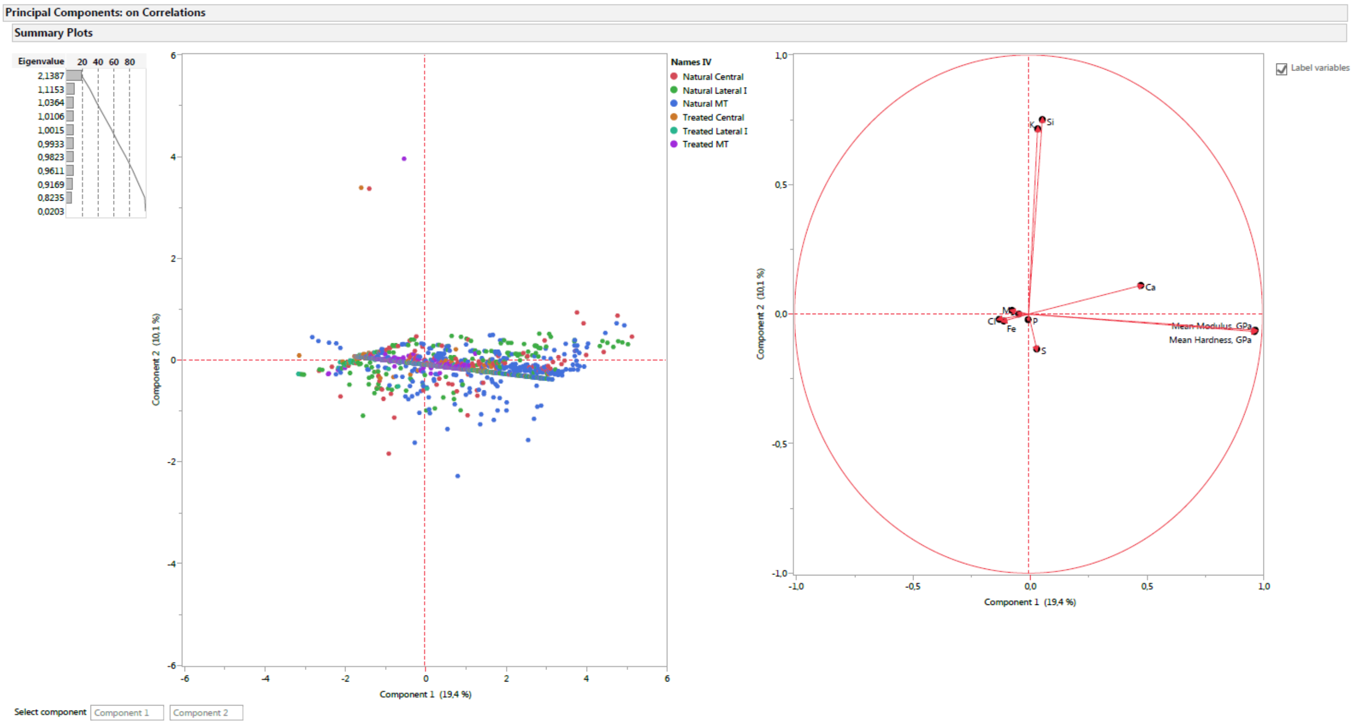

Supplementary Figure 12. PCA of all tooth types, except lateral tooth II. No clustering can be detected.
